# Oral Presentation

**DOI:** 10.1111/ene.70622

**Published:** 2026-06-26

## Saturday, June 27 2026

## Autonomic Nervous System Diseases 1

## OPR‐001

### Development of autonomic dysreflexia card

#### E. Hagen

##### Department of Clinical Medicine, Aarhus University, Aarhus, Denmark

**Background and Aims:** Autonomic dysreflexia (AD) is a potentially life‐threatening medical emergency, most commonly occurring in individuals with spinal cord injury (SCI) at or above the T6 neurological level. AD is characterized by a sudden increase in systolic blood pressure of >20 mmHg above a person's baseline, which is the result of an unopposed sympathetic response to noxious or non‐noxious stimuli below the injury. To facilitate prompt recognition and targeted intervention of AD by clinicians, rapid access to essential information, including patient‐specific information, is critical.

**Methods:** Through joint online surveys and meetings, the International Spinal Cord Society (ISCoS) and the European Society of Physical and Rehabilitation Medicine (ESPRM) have systematically developed an International AD Medical Emergency Card highlighting causes, management, and prevention of AD.

**Results:** The International AD Medical Emergency Card has been uploaded on ESPRM and ISCoS platforms. It contains Personal information, General information concerning AD, Management of AD and QR codes linking to international standards, guidelines and a review. It is a pocket size‐Instructions for completing and editing the Medical Emergency Card has been developed, as well as a translation process. The card is bilingual, in English and a national language.

**Figure:**
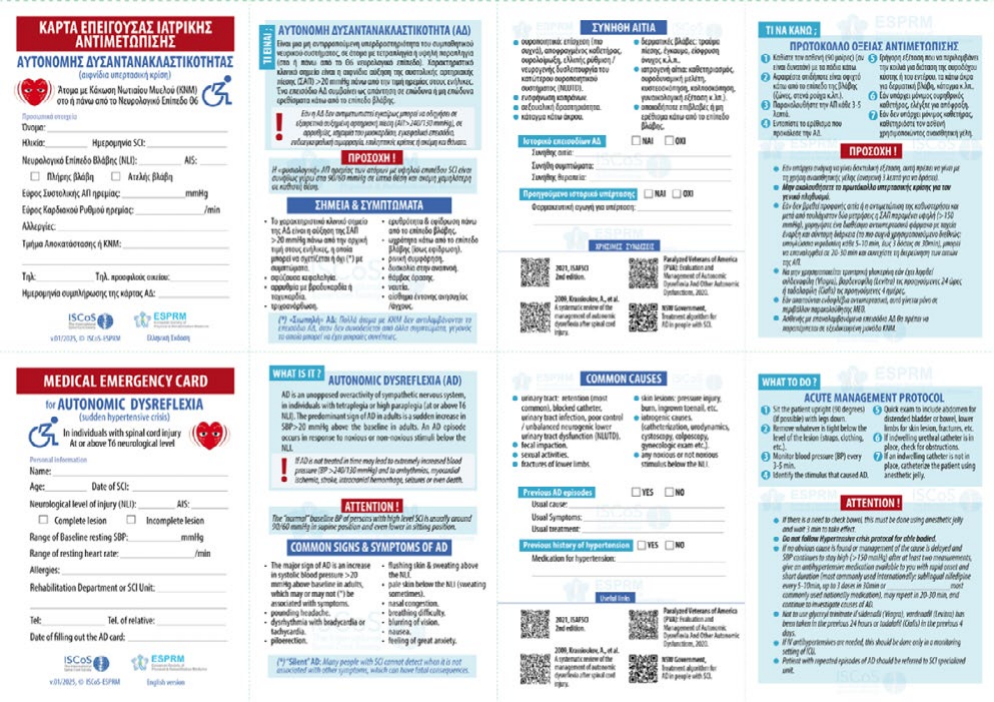
**FIGURE 1** Colour version of the card in full size for printing on a colour printer. There are 8 sections. Four in Greek and four in English.

**Conclusion:** The International AD Medical Emergency Card has been developed and will be updated regularly. It is presently in English and Greek and translations to other languages is underway.

**Disclosure:** Nothing to disclose.

## OPR‐002

### Diagnosing prodromal synucleinopathies: What is the role of cardiac metaiodobenzylguanidine scintigraphy?

#### G. Chiaro ^1^; Z. Jiang^1^; A. Rogeau^2^; J. Bomanji^3^; V. Iodice^4^

##### ^*1*^ *National Autonomic Centre, The National Hospital for Neurology and Neurosurgery, London, UK;* ^*2*^ *Department of Nuclear Medicine, Pitié‐Salpêtrière Hospital, APHP Sorbonne Université, Paris, France;* ^*3*^ *Institute of Nuclear Medicine, University College London Hospital NHS Foundation Trust, London, UK;* ^*4*^ *NIHR University College London Hospitals Biomedical Research Centre, London, UK*

**Background and Aims:** Cardiac metaiodobenzylguanidine (MIBG) scintigraphy differentiates Lewy body disorders (LBD), such as Parkinson's disease and dementia with Lewy bodies, from multiple system atrophy (MSA). Reduced cardiac MIBG uptake reflects postganglionic sympathetic denervation typical of LBD, while uptake is usually preserved in MSA. Abnormal uptake has also been reported in pure autonomic failure (PAF). We aimed to characterize cardiac MIBG uptake in patients with an initial diagnosis of PAF (iPAF) and evaluate its role as a prognostic tool in stratifying risk of phenoconversion.

**Methods:** As part of our longitudinal natural history study, consecutive patients with iPAF underwent prospective clinical and autonomic evaluations, including cardiac MIBG scintigraphy. Patients were followed until conversion to LBD or MSA, or until last follow‐up in 2025.

**Results:** Among 106 patients with iPAF, 63 (59%) remained clinically stable for ≥3 years; 62 (98%) had abnormal MIBG uptake. The single patient with normal uptake was under 50 years old with severe urogenital dysfunction. Thirty‐four patients (32%) converted to LBD; 33 (97%) showed abnormal MIBG uptake up to 9 years before phenoconversion. Eight patients (8%) converted to MSA, all with normal uptake up to 1 year before phenoconversion. One patient with parkinsonism of uncertain diagnosis also had normal uptake.

**Conclusion:** Cardiac MIBG scintigraphy was highly sensitive for detecting cardiac sympathetic denervation in a large cohort of iPAF and accurately predicted disease trajectories up to 9 years before phenoconversion, supporting its value for risk stratification and counseling.

**Disclosure:** Nothing to disclose.

## OPR‐003

### Cardiovascular reflex tests as early indicators of disease‐modifying treatments eligibility in asymptomatic hereditary transthyretin amyloidosis carriers: Introducing the “Two or more rule”

#### G. Carrozzo ^1^; M. Gianoli^2^; S. Longhi^3^; I. Cani^2^; I. Ruotolo^3^; M. Schiavo^3^; C. Fasano^4^; R. D'Angelo^2^; R. Rinaldi^2^; S. Domenichini^2^; A. Cerere^2^; G. Calandra‐Buonaura^5^; P. Guaraldi^2^

##### ^*1*^ *Department of Biomedical and NeuroMotor Sciences (DIBINEM), University of Bologna, Bologna, Italy;* ^*2*^ *IRCCS Istituto delle Scienze Neurologiche di Bologna, Bologna, Italy;* ^*3*^ *Department of Medical and Surgical Sciences (DIMEC), University of Bologna, Bologna, Italy; European Reference Network for Rare, Low Prevalence and Complex Diseases of the Heart – ERN GUARD‐Heart;* ^*4*^ *AOU Careggi and Department of Neurosciences, Drug and Child Health, University of Florence, Florence, Italy;* ^*5*^ *Department of Biomedical and NeuroMotor Sciences (DIBINEM), University of Bologna, Bologna, Italy; IRCCS Istituto delle Scienze Neurologiche di Bologna, Bologna, Italy*

**Background and Aims:** Hereditary transthyretin amyloidosis (ATTRv) is a progressive multisystem disorder in which autonomic dysfunction may precede both neuropathy and cardiomyopathy. Early identification of autonomic involvement is crucial, as disease‐modifying treatments (DMTs) are most effective in the earliest disease stages.

**Methods:** In this monocentric, cross‐sectional study, 47 asymptomatic ATTRv carriers and 47 healthy controls underwent cardiovascular reflex testing (CRTs), including head‐up tilt test (HUTT), Valsalva manoeuvre (VM), deep breathing (DB), cold face (CF), and handgrip (HG). Forty carriers also underwent ^99m^Tc‐DPD scintigraphy.

**Results:** ATTRv carriers exhibited a significantly reduced overshoot during VM (*p* < 0.001) and attenuated blood pressure responses during HUTT, despite the absence of overt orthostatic hypotension. Overall, 21 of 47 carriers (44.7%) demonstrated at least one abnormal CRT and 7 of 21 were initiated on DMTs. All treated individuals presented with two or more abnormal CRTs, most frequently involving overshoot and Valsalva ratio (VR), or the combination of overshoot, VR, and DB abnormalities.

**Conclusion:** CRTs therefore appear to be sensitive markers of early autonomic dysfunction in ATTRv carriers. The presence of two or more abnormal CRTs (e.g., overshoot <15 mmHg, VR <1.24, ΔI–E in DB <10 bpm) identifies a subgroup at higher risk and may represent a practical threshold for advanced evaluation and early therapeutic intervention. Integrating this “two‐or‐more rule” into routine monitoring may enable timely treatment initiation and improve long‐term outcomes in presymptomatic carriers of ATTRv.

**Disclosure:** Nothing to disclose.

## OPR‐004

### Autonomic involvement in small fiber neuropathy

#### M. Nolano ^1^; V. Provitera^2^; I. Borreca^2^; A. Areniello^2^; G. Caporaso^2^; D. Dell'Aversana^3^; F. Masciarelli^3^; G. Ciccarelli^3^; F. Vitale^3^; R. Lombardi^4^; S. Mazzetti^5^; A. Elia^5^; D. Cazzato^6^; R. Eleopra^5^; S. Tozza^3^; F. Manganelli^3^; G. Lauria^7^; G. Devigili^5^

##### ^*1*^ *Neurology Department, Skin Biopsy Laboratory, Istituti Clinici Scientifici Maugeri IRCCS, Telese Terme, Italy. 2. Department of Neurosciences, Reproductive Sciences and Odontostomatology, University Federico II of Naples, Naples, Italy;* ^*2*^ *Neurology Department, Skin Biopsy Laboratory, Istituti Clinici Scientifici Maugeri IRCCS, Telese Terme, Italy;* ^*3*^ *Department of Neurosciences, Reproductive Sciences and Odontostomatology, University Federico II of Naples, Naples, Italy;* ^*4*^ *Neuroalgology Unit, Fondazione IRCCS Istituto Neurologico “Carlo Besta,” Milan, Italy;* ^*5*^ *Movement Disorders Unit, Fondazione IRCCS Istituto Neurologico “Carlo Besta,” Milan, Italy;* ^*6*^ *Clinical Neurophysiology Unit, Fondazione IRCCS Istituto Neurologico “Carlo Besta,” Milan, Italy;* ^*7*^ *1. Neuroalgology Unit, Fondazione IRCCS Istituto Neurologico “Carlo Besta,” Milan, Italy; 2. Medical Genetics and Neurogenetics Unit, Fondazione IRCCS Istituto Neurologico Carlo Besta, Milan, Italy*

**Background and Aims:** The diagnosis of small fiber neuropathy (SFN) is based on sensory symptoms and signs, supported by sensory dysfunction assessment using quantitative sensory testing and/or evidence of intraepidermal nerve fiber (IENF) loss. However, autonomic impairment is frequently part of the clinical picture.

**Methods:** We assessed sensory and autonomic symptoms, as well as cardiovascular and sudomotor dysfunction, using the SFN‐SIQ and COMPASS‐31 questionnaires, cardiovascular reflex tests (Ewing score), sympathetic skin response (SSR), dynamic sweat test (DST), and IENF density at the distal leg. A total of 337 patients with SFN (age 49.0 ± 14.27 years; 237 women) from three centers were included.

**Results:** According to etiology, patients were classified into five groups: dysimmune (*n* = 103), metabolic (*n* = 39), toxic (*n* = 5), genetic (*n* = 22), and idiopathic (*n* = 168). No significant differences in functional or morphological assessments were observed among groups, except for higher COMPASS‐31 scores in patients with dysimmune etiology. In the overall population, a COMPASS‐31 total score >30 was found in 59.3% of patients, while an Ewing score ≥2 was observed in 26.9%. SSR was absent in 7 patients and showed reduced amplitude in 10.6%. DST at the distal leg demonstrated hypohidrosis in 79.1% of patients compared with cut‐off values derived from 210 healthy controls. IENF loss was detected in 81% of patients. Overall, sensory and autonomic symptom severity (SFN‐SIQ and COMPASS‐31 total scores) was higher in women, who also exhibited greater sudomotor impairment.

**Conclusion:** These findings indicate that autonomic involvement is common in SFN and support the inclusion of postganglionic sudomotor function assessment in the diagnostic criteria for SFN.

**Disclosure:** Nothing to disclose.

## OPR‐005

### To tilt or to stand? Comparing the accuracy of current diagnostic tests for orthostatic hypotension in Parkinson's disease

#### N. Campese ^1^; A. Van Der Stam^2^; G. Goebel^3^; F. Leys^1^; J. Ndayisaba^1^; R. Granata^1^; S. Kiechl^1^; W. Poewe^1^; R. Thijs^4^; A. Fanciulli^1^

##### ^*1*^ *Department of Neurology, Medical University of Innsbruck, Innsbruck, Austria;* ^*2*^ *Radboud University Medical Center, Donders Institute for Brain, Cognition and Behavior, Department of Neurology, Center of Expertise for Parkinson & Movement Disorders, Nijmegen;* ^*3*^ *Institute of Clinical Epidemiology, Public Health, Health Economics, Medical Statistics and Informatics, Medical University of Innsbruck, Innsbruck (Austria);* ^*4*^ *Department of Neurology, Leiden University Medical Centre, Leiden*

**Background and Aims:** Classic orthostatic hypotension (cOH) affects approximately 30% of persons with Parkinson's disease (pwPD), being associated with functional read‐outs like orthostatic intolerance, syncope and falls. cOH is defined by a systolic and/or diastolic blood pressure drop ≥20/10 mmHg within 3 min of an orthostatic challenge, be it an active standing test (AST) or a passive head‐up tilt‐test (HUT). However, the diagnostic accuracy of these tests in pwPD remains unclear.

**Methods:** In a retrospective cohort of 173 pwPD, who underwent both the AST and the HUT at the Medical University of Innsbruck between 2007 and 2020, we compared the diagnostic accuracy for cOH of HUT and AST.

**Results:** Both AST and HUT showed acceptable sensitivity for cOH in pwPD [63% (confidence interval, CI 48%–77%) vs. 73% (CI 59%–85%)] with moderate agreement between the two tests (Cohen's *k* 0.43, CI 0.26–0.60). Compared to AST, the HUT warranted a 10% sensitivity gain for cOH. We found no differences in the frequency of orthostatic intolerance, syncope and falls across persons with cOH at both the AST and the HUT and those with cOH at either the AST or the HUT only (*p* = 1, *p* = 0.26 and *p* = 0.69 respectively).

**Conclusion:** Identifying and managing cOH improves the quality of life and prevents adverse outcomes in pwPD. The HUT shows higher sensitivity for cOH compared to AST but a proportion of pwPD shows cOH only at either the AST or the HUT. In clinical practice, AST and HUT represent therefore complimentary tests, with similar sensitivity for cOH‐related functional read‐outs.

**Disclosure:** Academic study without external financial support. The authors have no conflict of interest to declare.

## Cognitive Neurology/Neuropsychology

## OPR‐006

### Substrates of consolidated resilience and impact on cognitive outcomes in multiple sclerosis

#### D. Mistri ^1^; M. Margoni^2^; P. Valsasina^1^; A. Meani^1^; N. Tedone^1^; M. Rubin^3^; G. Guido^3^; F. Esposito^4^; M. Rocca^3^; M. Filippi^5^

##### ^*1*^ *Neuroimaging Research Unit, Division of Neuroscience, IRCCS San Raffaele Scientific Institute, Milan, Italy;* ^*2*^ *Neuroimaging Research Unit, Division of Neuroscience, Neurology Unit, and Neurorehabilitation Unit, IRCCS San Raffaele Scientific Institute, Milan, Italy;* ^*3*^ *Neuroimaging Research Unit, Division of Neuroscience, and Neurology Unit, IRCCS San Raffaele Scientific Institute, Milan, Italy; and Vita‐Salute San Raffaele University, Milan, Italy;* ^*4*^ *Neurology Unit, IRCCS San Raffaele Scientific Institute, Milan, Italy;* ^*5*^ *Neuroimaging Research Unit, Division of Neuroscience, Neurology Unit, Neurorehabilitation Unit, and Neurophysiology Service, IRCCS San Raffaele Scientific Institute, Milan, Italy; and Vita‐Salute San Raffaele University, Milan, Italy*

**Background and Aims:** Consolidated resilience integrates cognitive reserve and cognitive resilience. Its relevance in multiple sclerosis (MS) and the underlying functional mechanisms remain unexplored.

**Methods:** Structural and functional 3.0 T MRI scans were acquired from 522 MS patients and 304 healthy controls (HCs). Consolidated resilience scores were estimated in patients using a partial least‐squares path model, combining proxies of cognitive reserve (education and intelligence quotient) and cognitive resilience (residuals from the regression of cognitive scores on T2‐hyperintense white matter lesion volumes and normalized brain volumes standardized to HCs). After a median follow‐up of 2.0 years (interquartile range = 1.1; 2.9 years), 179 patients underwent cognitive and MRI reassessment. Resting state (RS) functional connectivity (FC) was estimated using independent component analysis. Linear mixed models tested the interaction between consolidated resilience and RS FC changes in cognitive networks in predicting variations in cognitive scores.

**Results:** At follow‐up, higher consolidated resilience was associated with better verbal and visuospatial memory, processing speed and verbal fluency (all *p* < 0.001). Significant three‐way interactions (consolidated resilience × RS FC changes × time) were observed in verbal memory (executive control network [β = −1.36, *p* = 0.029], left frontoparietal network [β = −0.79, *p* = 0.034] and default mode network [β = −0.71, *p* = 0.035]) and processing speed changes (salience network [β = −1.43, *p* < 0.001], executive control network [β = −0.92, *p* = 0.030], left frontoparietal network [β = −0.66, *p* = 0.034] and default mode network [β = −0.83, *p* = 0.003]).

**Conclusion:** Consolidated resilience moderated the association between cognitive functioning and FC changes. In highly resilient patients, better cognitive performance was associated with stable or decreased FC, whereas in patients with lower resilience, better cognitive outcomes correlated with increased FC.

**Figure:**
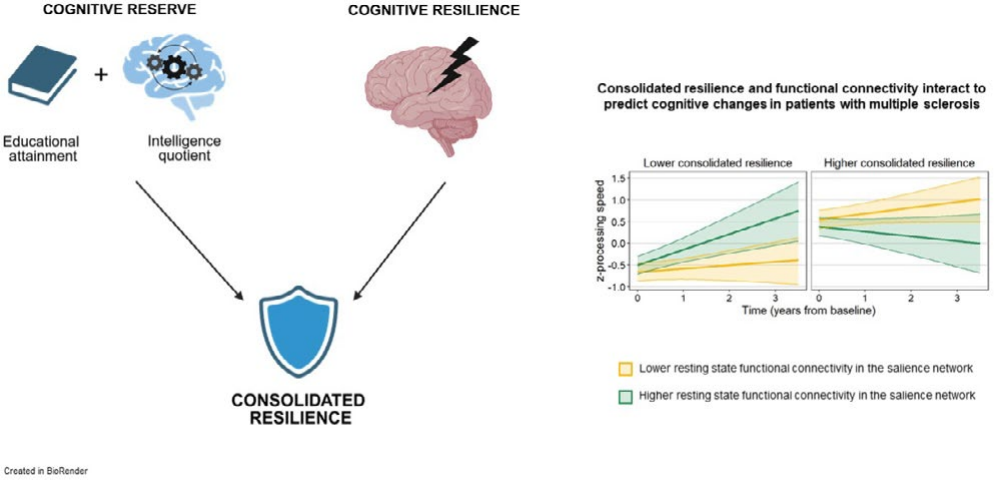
**FIGURE 1** Consolidated resilience integrates cognitive reserve and cognitive resilience.

**Disclosure:** D. Mistri, P. Valsasina, A. Meani, N. Tedone, M. Rubin, G. Guido and F. Esposito report no competing interests. M. Margoni reports grants and personal fees from Sanofi Genzyme, Merck Serono, Roche, Biogen, Amgen and Novartis. Filippi is Editor‐in‐Chief of the Journal of Neurology, Associate Editor of Human Brain Mapping, Neurological Sciences, and Radiology; received compensation for consulting services from Almirall, Biogen, Bristol‐Myers Squibb, Eli Lilly, Merck, Novartis, Roche, Sanofi; speaking activities from Amgen, Bayer, Biogen, Bristol‐Myers Squibb, Celgene, Chiesi Italia SpA, Eisai, Eli Lilly, Fujirebio, Genzyme, Janssen, Merck, Neopharmed Gentili, Neuraxpharm, Novartis, Novo Nordisk, Roche, Sanofi, Takeda; participation in Advisory Boards for Alexion, Biogen, Bristol‐Myers Squibb, Eli Lilly, GE Healthcare Ltd, Merck, Neuraxpharm, Novartis, Roche, Sandoz, Sanofi, Takeda; scientific direction of educational events for Biogen, Merck, Roche, Celgene, Bristol‐Myers Squibb, Lilly, Novartis, Sanofi‐Genzyme; he receives research support from Biogen Idec, Merck‐Serono, Novartis, Roche, the Italian Ministry of Health, the Italian Ministry of University and Research, and Fondazione Italiana Sclerosi Multipla. M.A. Rocca received consulting fees from Biogen, Bristol Myers Squibb, Roche; and speaker honoraria from Alexion, Biogen, Bristol Myers Squibb, Celgene, Horizon Therapeutics Italy, Merck Serono SpA, Mitsubishi‐Tanabe Pharma, Neuraxpharm, Novartis, Roche, Sandoz, and Sanofi. She receives research support from the MS Society of Canada, the Italian Ministry of Health, the Italian Ministry of University and Research, and Fondazione Italiana Sclerosi Multipla. She is Associate Editor for Multiple Sclerosis and Related Disorders; and Associate Co‐Editor for Europe and Africa for Multiple Sclerosis Journal.

## OPR‐007

### Glymphatic dysfunction by DTI‐ALPS links to GFAP, brain atrophy, white matter hyperintensity progression, and processing speed decline in ONDRI cohort

#### D. Andriuta ^1^; J. Ramirez^2^; L. Woods^2^; E. Gibson^2^; M. Kang^2^; S. Berberian^2^; F. Gao^2^; M. Wood Alexander^2^; J. Rabin^2^; C. Scott^2^; D. Broberg^3^; R. Bartha^3^; R. Swartz^4^; M. Masellis^4^; S. Black^4^

##### ^*1*^ *Department of Neurology, Amiens University Medical Center, Amiens, France;* ^*2*^ *Dr. Sandra Black Centre for Brain Resilience & Recovery, Sunnybrook Research Institute, Toronto, Canada;* ^*3*^ *Western University/Robarts Research Institute, London, Canada;* ^*4*^ *Division of Neurology, Sunnybrook Health Sciences Centre, Toronto, Canada*

**Background and Aims:** Glymphatic clearance is implicated in neurocognitive disorders, but its role in human is unclear. The diffusion tensor image analysis along the perivascular space (DTI‐ALPS) index provides a noninvasive MRI marker of glymphatic function. We examined the association between baseline DTI‐ALPS and 1‐year changes in gray matter (GM) and white matter hyperintensity (WMH) volumes. Secondary aims were to assess whether plasma GFAP mediates DTI‐ALPS effects on GM atrophy and the effect of DTI‐ALPS on processing speed decline via GM volume.

**Methods:** We studied 304 participants from the Ontario Neurodegenerative Disease Research Initiative (ONDRI): AD/MCI (*n* = 120), cerebrovascular disease (CVD, *n* = 139), and frontotemporal dementia (FTD, *n* = 45). MRI‐derived GM, WMH, perivascular space, DTI‐ALPS indices, total intracranial volumes were measured. Plasma GFAP was quantified. Processing speed was assessed with a composite *z*‐score from the Digit Symbol Modalities Test and Trail Making Test A. Linear regression and mediation analyses were performed.

**Results:** Baseline DTI‐ALPS was significantly associated with 1‐year GM loss (β = 0.111, *p* = 0.048) and WMH increase (β = −0.148, *p* = 0.013). Baseline plasma GFAP mediated the effect of DTI‐ALPS on GM atrophy (indirect effect: 845.94; 95% CI [73.89, 3370.62]). Baseline left DTI‐ALPS influenced 1‐year processing speed decline via GM volume (indirect effect: 0.1385; 95% CI [0.042, 0.3016]).

**Conclusion:** Glymphatic dysfunction, measured by DTI‐ALPS, is linked to structural brain changes and cognitive decline in mixed neurodegenerative and cerebrovascular disorders. Plasma GFAP mediates GM atrophy, highlighting astroglial involvement. DTI‐ALPS, combined with plasma GFAP, may serve as a noninvasive biomarker to guide mechanistic studies and therapeutic strategies targeting glymphatic pathways.

**Disclosure:** The authors report no disclosures of relevance to the manuscript. The ONDRI study was conducted in part with the support of the Ontario Brain Institute, an independent non‐profit corporation, funded partially by the Ontario government (GRANT# ONDRI/OBI‐34739).

## OPR‐008

### Structural and functional brain correlates of fatigue in neuromyelitis optica spectrum disorder

#### M. Grosselle^1^; N. Tedone ^1^; P. Valsasina^1^; P. Preziosa^1,2,3^; M. Rubin^1,2,3^; L. Moiola^2^; M. Filippi^1,2,3,4,5^; M. Rocca^1,2,3^

##### ^*1*^ *Neuroimaging Research Unit, Division of Neuroscience, IRCCS San Raffaele Scientific Institute, Milan, Italy;* ^*2*^ *Neurology Unit, IRCCS San Raffaele Scientific Institute, Milan, Italy;* ^*3*^ *Vita‐Salute San Raffaele University, Milan, Italy;* ^*4*^ *Neurorehabilitation Unit, IRCCS San Raffaele Scientific Institute, Milan, Italy;* ^*5*^ *Neurophysiology Service, IRCCS San Raffaele Scientific Institute, Milan, Italy*

**Background and Aims:** Fatigue in NMOSD remains poorly understood. We investigated structural and functional MRI correlates of fatigue in NMOSD patients.

**Methods:** Twenty‐five NMOSD patients and 60 healthy controls (HC) underwent 3T brain structural and functional MRI scans, and fatigue assessment using the Modified Fatigue Impact Scale (MFIS). In patients, associations were evaluated between MFIS and regional distribution of T2‐hyperintense lesions, microstructural white matter (WM) damage, regional grey matter (GM) atrophy, and seed‐based resting‐state (RS) functional connectivity (FC) abnormalities within main brain networks.

**Results:** Higher MFIS scores were associated with lower GM volume in the right superior frontal gyrus, right posterior cingulate cortex, left inferior frontal gyrus, and right parahippocampal gyrus (*r* range = −0.37/−0.40, *p* < 0.001 uncorrected). Higher MFIS was also associated with increased RS FC within the two primary motor cortices (BA4), as well as between the right BA4 and the left postcentral gyrus, and between the left BA4 and the right temporal cortex (*r* range = 0.36/0.47, *p* < 0.001 uncorrected). Finally, higher MFIS scores were associated with: (1) increased RS FC between the right insula and the right inferior frontal gyrus (*r* = 0.46, *p* < 0.001), (2) decreased RS FC within the left posterior cingulate cortex of the default‐mode network, and (3) decreased RS FC between the left caudate and bilateral thalami (*r* = range −0.45/−0.48, *p* < 0.001). No associations were found between fatigue and distribution of T2‐hyperintense lesions or WM microstructural abnormalities.

**Conclusion:** Fatigue in NMOSD is primarily related to large‐scale brain network dysfunction rather than focal WM damage, supporting a network‐based MRI biomarker framework for this symptom.

**Disclosure:** M. Grosselle, P. Valsasina, and M. Rubin have nothing to disclose. P. Preziosa received speaker honoraria from Roche, Biogen, Novartis, Merck, Bristol Myers Squibb, Genzyme, Horizon and Sanofi. L. Moiola received speaker honoraria from Roche. M. Filippi is Editor‐in‐Chief of the Journal of Neurology, Associate Editor of Human Brain Mapping, Neurological Sciences, and Radiology; received compensation for consulting services from Almirall, Biogen, Bristol‐Myers Squibb, Eli Lilly, Merck, Novartis, Roche, Sanofi; speaking activities from Amgen, Bayer, Biogen, Bristol‐Myers Squibb, Celgene, Chiesi Italia SpA, Eisai, Eli Lilly, Fujirebio, Genzyme, Janssen, Merck, Neopharmed Gentili, Neuraxpharm, Novartis, Novo Nordisk, Roche, Sanofi, Takeda; participation in Advisory Boards for Alexion, Biogen, Bristol‐Myers Squibb, Eli Lilly, GE Healthcare Ltd, Merck, Neuraxpharm, Novartis, Roche, Sandoz, Sanofi, Takeda; scientific direction of educational events for Biogen, Merck, Roche, Celgene, Bristol‐Myers Squibb, Lilly, Novartis, Sanofi‐Genzyme; he receives research support from Biogen Idec, Merck‐Serono, Novartis, Roche, the Italian Ministry of Health, the Italian Ministry of University and Research, and Fondazione Italiana Sclerosi Multipla. M.A. Rocca received consulting fees from Biogen, Bristol Myers Squibb, Roche; and speaker honoraria from Alexion, Biogen, Bristol Myers Squibb, Celgene, Horizon Therapeutics Italy, Merck Serono SpA, Mitsubishi‐Tanabe Pharma, Neuraxpharm, Novartis, Roche, Sandoz, and Sanofi. She receives research support from the MS Society of Canada, the Italian Ministry of Health, the Italian Ministry of University and Research, and Fondazione Italiana Sclerosi Multipla. She is Associate Editor for Multiple Sclerosis and Related Disorders; and Associate Co‐Editor for Europe and Africa for Multiple Sclerosis Journal.

## OPR‐009

### Blood biomarkers of Alzheimer's disease and progression of brain atrophy and cerebrovascular burden in cognitively healthy older adults

#### M. Valletta ^1^; D. Liborio^1^; E. Laukka^1^; G. Kalpouzos^1^; J. Oltra^1^; D. Rizzuto^1^; M. Canevelli^2^; C. Fredolini^3^; C. Qiu^1^; B. Winblad^4^; L. Fratiglioni^1^; G. Grande^1^

##### ^*1*^ *Aging Research Center, Department of Neurobiology, Care Sciences and Society, Karolinska Institutet, Stockholm, Sweden;* ^*2*^ *Department of Human Neuroscience, Sapienza University, Rome, Italy;* ^*3*^ *Affinity Proteomics Stockholm, Science for Life Laboratory, Royal Institute of Technology (KTH), Solna, Sweden;* ^*4*^ *Division of Neurogeriatrics, Department of Neurobiology, Care Sciences and Society, Karolinska Institutet, Solna, Sweden*

**Background and Aims:** Blood biomarkers of Alzheimer's disease (AD) have been linked to structural brain alterations in clinical cohorts, but evidence in cognitively healthy populations remains limited. We investigated the associations between AD blood biomarkers and changes in brain volumes and cerebrovascular burden in cognitively healthy community‐dwelling older adults.

**Methods:** In a cohort of 372 dementia‐free older adults from Sweden, baseline blood levels of amyloid‐β42/40 (Aβ42/40), phosphorylated tau (p‐tau)181 and 217, total tau (t‐tau), neurofilament light chain (NfL), and glial fibrillary acidic protein (GFAP) were measured and their *z*‐scores derived. Brain MRI was performed at baseline and after 3 and/or 6 years. Associations between AD blood biomarkers and changes in volumes of total brain tissue (TBTV), ventricles, hippocampus, amygdala and white matter hyperintensities (WMH) were examined using linear mixed models.

**Results:** Higher p‐tau181 and p‐tau217 were associated with faster hippocampal volume loss (beta * year −0.036, 95% CI −0.063, −0.009; and beta * year −0.051, 95% CI −0.080, −0.021, respectively), and p‐tau217 was additionally associated with amygdala volume decline. Elevated GFAP was linked to faster TBTV decline, ventricular enlargement, and hippocampal shrinkage. Higher NfL levels were associated with steeper TBTV decline, ventricular enlargement, hippocampal and amygdala shrinkage, and faster WMH accumulation.

**Figure:**
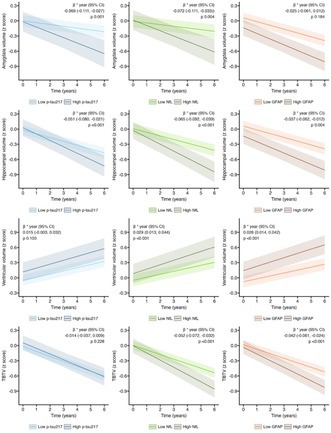
**FIGURE 1** Annual rate of change in total brain tissue (TBTV), ventricular and hippocampus and amygdala volumes, by baseline levels of p‐tau217, NfL and GFAP.

**Figure:**
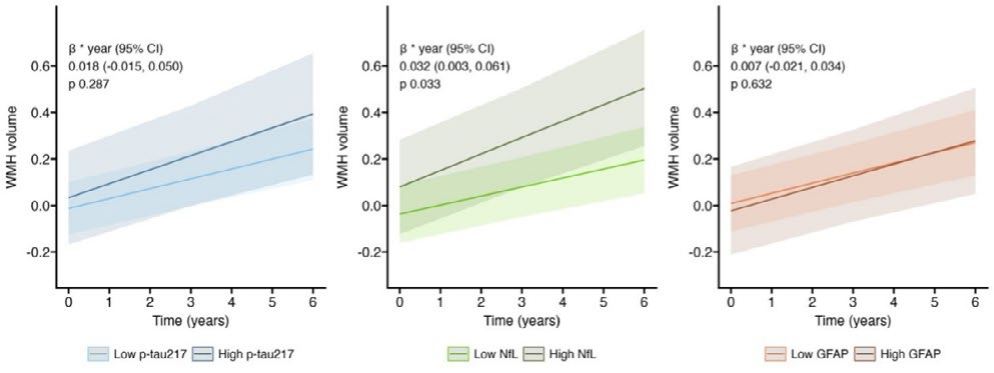
**FIGURE 2** Annual rate of change in white matter hyperintensities (WMH) volume by baseline levels of p‐tau217, NfL and GFAP.

**Conclusion:** In cognitively healthy older adults, elevated blood levels of p‐tau isoforms were linked to decline in AD‐specific regions, whereas NfL and GFAP were associated with widespread atrophy and NfL also with WMH accumulation. These findings suggest that altered AD blood biomarkers may capture the earliest stages of dementia‐related brain changes in the general population.

**Disclosure:** Nothing to disclose.

## OPR‐010

### Precuneus and posterior cingulate functional connectivity in multiple sclerosis: Age, onset timing and cognitive implications

#### N. Tedone ^1^; P. Valsasina^2^; A. Gallo^3^; P. Pantano^4^; M. Margoni^5^; P. Preziosa^6^; M. Filippi^7^; M. Rocca^6^

##### ^*1*^ *Neuroimaging Research Unit, Division of Neuroscience, IRCCS San Raffaele Scientific Institute, Milan, Italy; and Vita‐Salute San Raffaele University, Milan, Italy;* ^*2*^ *Neuroimaging Research Unit, Division of Neuroscience, IRCCS San Raffaele Scientific Institute, Milan, Italy;* ^*3*^ *University of Campania “Luigi Vanvitelli”, Department of Advanced Medical and Surgical Sciences, and 3T MRI‐Center, Naples, Italy;* ^*4*^ *Sapienza University of Rome, Department of Human Neurosciences, Rome, Italy; and IRCCS NEUROMED, Pozzilli, Italy;* ^*5*^ *Neuroimaging Research Unit, Division of Neuroscience, Neurology Unit, and Neurorehabilitation Unit, IRCCS San Raffaele Scientific Institute, Milan, Italy;* ^*6*^ *Neuroimaging Research Unit, Division of Neuroscience, and Neurology Unit, IRCCS San Raffaele Scientific Institute, Milan, Italy; and Vita‐Salute San Raffaele University, Milan, Italy;* ^*7*^ *Neuroimaging Research Unit, Division of Neuroscience, Neurology Unit, Neurorehabilitation Unit, and Neurophysiology Service, IRCCS San Raffaele Scientific Institute, Milan, Italy; and Vita‐Salute San Raffaele University, Milan, Italy*

**Background and Aims:** Precuneus and posterior cingulate cortex (PCC) are cognitive hubs frequently involved in multiple sclerosis (MS). We explored the effects of age and age at onset on static (sFC) and time‐varying functional connectivity (TVFC) of precuneus/PCC and their association with cognition.

**Methods:** We enrolled 279 right‐handed MS patients and 130 matched healthy controls (HC) from three Italian sites. All subjects underwent Rao's battery and 3T resting‐state fMRI assessment. sFC was computed with seed‐to‐voxel correlation; TVFC with sliding‐window correlation using bilateral precuneus and PCC as seeds.

**Results:** Compared with HC, MS patients showed limited sFC increase between the precuneus/PCC and parieto‐frontal regions, and widespread sFC decrease within the default‐mode network. Time‐varying analysis revealed localized TVFC increase of the precuneus/PCC with lingual and cerebellar regions. In MS, a modulation of age on connectivity (significant at disease‐by‐age interaction analysis) was found: older age was associated with decreased sFC between the precuneus and cerebellum, and between PCC and anterior cingulate cortex. Similarly, older age was associated with decreased TVFC between the precuneus and left caudate (associated with better visual memory performance [rho = −0.19, *p* = 0.002]) and between PCC and right superior frontal gyrus. Older age at disease onset was associated with decreased TVFC between the PCC and right thalamus, right angular gyrus, right middle frontal and left postcentral gyri, these latter two were associated with worse fluency performance (rho = 0.13–0.15, *p* = 0.02–0.03).

**Conclusion:** In MS, age and age at disease onset significantly influenced sFC and TVFC of the precuneus and PCC, contributing to explain cognitive performances.

**Disclosure:** N. Tedone, P. Valsasina have nothing to disclose. A. Gallo received speaker and consulting fees from Biogen, Genzyme, Merck Serono, Mylan, Novartis, Roche, and Teva, and receives research support from Fondazione Italiana Sclerosi Multipla. P. Pantano has received funding for travel from Novartis, Genzyme, and Bracco and a speaking honorarium from Biogen. She received research support from Italian Ministry of Foreign Affairs and Fondazione Italiana Sclerosi Multipla. M. Margoni reports grants and personal fees from Sanofi Genzyme, Merck Serono, Roche, Biogen, Amgen and Novartis. P. Preziosa received speaker honoraria from Roche, Biogen, Novartis, Merck, Bristol Myers Squibb, Genzyme, Horizon and Sanofi. M. Filippi is Editor‐in‐Chief of the Journal of Neurology, Associate Editor of Human Brain Mapping, Neurological Sciences, and Radiology; received compensation for consulting services from Almirall, Biogen, Bristol‐Myers Squibb, Eli Lilly, Merck, Novartis, Roche, Sanofi; speaking activities from Amgen, Bayer, Biogen, Bristol‐Myers Squibb, Celgene, Chiesi Italia SpA, Eisai, Eli Lilly, Fujirebio, Genzyme, Janssen, Merck, Neopharmed Gentili, Neuraxpharm, Novartis, Novo Nordisk, Roche, Sanofi, Takeda; participation in Advisory Boards for Alexion, Biogen, Bristol‐Myers Squibb, Eli Lilly, GE Healthcare Ltd, Merck, Neuraxpharm, Novartis, Roche, Sandoz, Sanofi, Takeda; scientific direction of educational events for Biogen, Merck, Roche, Celgene, Bristol‐Myers Squibb, Lilly, Novartis, Sanofi‐Genzyme; he receives research support from Biogen Idec, Merck‐Serono, Novartis, Roche, the Italian Ministry of Health, the Italian Ministry of University and Research, and Fondazione Italiana Sclerosi Multipla. M.A. Rocca received consulting fees from Biogen, Bristol Myers Squibb, Roche; and speaker honoraria from Alexion, Biogen, Bristol Myers Squibb, Celgene, Horizon Therapeutics Italy, Merck Serono SpA, Mitsubishi‐Tanabe Pharma, Neuraxpharm, Novartis, Roche, Sandoz, and Sanofi. She receives research support from the MS Society of Canada, the Italian Ministry of Health, the Italian Ministry of University and Research, and Fondazione Italiana Sclerosi Multipla. She is Associate Editor for Multiple Sclerosis and Related Disorders; and Associate Co‐Editor for Europe and Africa for Multiple Sclerosis Journal.

## Neuro‐Oncology

## OPR‐011

### Leptomeningeal metastases from primary brain tumors: Still a neglected complication

#### E. Pronello ^1^; F. Bruno^1^; A. Pellerino^1^; L. Bertero^2^; E. Marchesani^1^; B. Raschio^1^; M. Borgognone^1^; A. Gastino^3^; D. Garbossa^4^; R. Soffietti^5^; R. Rudà^1^

##### ^*1*^ *Division of Neuro‐Oncology, Department of Neuroscience “Rita Levi Montalcini”, University of Turin, Turin, Italy;* ^*2*^ *Pathology Unit, Department of Medical Sciences, University of Turin, Italy;* ^*3*^ *Division of Radiotherapy, Department of Oncology, University and City of Health and Science, Turin, Italy, Turin, Italy;* ^*4*^ *Division of Neurosurgery, Dept. of Neuroscience “Rita Levi Montalcini”, University and City of Health and Science Hospital, Turin, Italy;* ^*5*^ *Candiolo Cancer Institute, FPO‐IRCCS, Candiolo, Turin, Italy*

**Background and Aims:** Leptomeningeal metastases (LM) from primary brain tumors are rare and aggressive with information about optimal management limited to small retrospective series. We report the characteristics and outcomes of a large cohort of patients with LM from primary brain tumors treated at our Institution.

**Methods:** Clinico‐therapeutic and outcome data of adult patients with LM from primary brain tumors were retrospectively collected. LM were classified according to EANO–ESMO criteria. Time‐to‐LM was defined as the interval from first surgery to LM diagnosis, and overall survival from LM (OS‐LM) as the interval from LM diagnosis to death or censoring. Statistical analyses included chi‐square tests and Cox regression models.

**Results:** 111 patients diagnosed between 1994 and 2025 were included (median age 50 years). The most frequent histologies were glioblastoma IDH‐wildtype (33.7%), astrocytoma IDH‐mutant (16.8%), medulloblastoma (15.8%), and ependymoma (15.8%). LM were synchronous with the primary tumor in 15.8% of cases and occurred mainly at local tumor progression. Diagnosis was based on clinico‐radiological findings in 72.6%. Median time‐to‐LM was 17.0 months and significantly differed by histology (*p* < 0.001), with the shortest interval in glioblastoma. Median OS‐LM was 4.0 months overall and was strongly influenced by histology (*p* < 0.001). On multivariable analysis, Karnofsky Performance Status ≥70 and LM from ependymomas were independently associated with longer OS‐LM.

**Conclusion:** LM represent a dismal complication of primary brain tumors. In this cohort, tumor histology and performance status were the main determinants of time‐to‐LM and survival, whereas EANO–ESMO class and treatment modalities did not significantly impact OS‐LM. Molecular analysis in the different subgroups are ongoing.

**Disclosure:** Nothing to disclose.

## OPR‐012

### IDH‐mutant gliomas treated with vorasidenib: Early seizure control correlates with [18F]‐DOPA PET response and D‐2‐hydroxyglutarate plasma reduction

#### F. Bruno ^1^; E. Pronello^1^; A. Pellerino^1^; P. Botta^1^; F. Scalvini^1^; F. Ponzetto^2^; L. Leoni^2^; A. Melis^2^; M. Zotta^3^; G. Mengozzi^2^; S. Morbelli^3^; R. Rudà^1^

##### ^*1*^ *Division of Neuro‐Oncology, Department of Neuroscience “Rita Levi Montalcini”, University and City of Health and Science Hospital, Turin, Italy;* ^*2*^ *Clinical Biochemistry Laboratory, Department of Medical Sciences, City of Health and Science Hospital and University of Turin, University of Turin, Italy, Turin, Italy;* ^*3*^ *Division of Nuclear Medicine, Department of Medical Sciences, City of Health and Science Hospital and University of Turin, University of Turin, Italy, Turin, Italy*

**Background and Aims:** Given the role of IDH mutations and D‐2‐hydroxyglutarate (D2HG) in epileptogenesis, the impact of IDH inhibitors on seizure control is appealing. This prospective study assessed patterns and timing of seizure response in grade 2 IDH‐mutant glioma patients receiving vorasidenib after surgery.

**Methods:** Seizures were recorded at the end of each 28‐day cycle. MRI, [18F] fluorodopa (F‐DOPA) PET, and D2HG plasma levels were performed at baseline and every 3 cycles.

**Results:** Of 56 patients, 12 (7 astrocytomas, 5 oligodendrogliomas) had persistent seizures prior to vorasidenib (mean frequency: 4/month); 9/12 had measurable F‐DOPA PET uptake; median D2HG plasma concentration was 39.7 ng/mL. Over a median treatment duration of 11.6 months, 6/12 achieved seizure freedom within 1 cycle, whereas 5/12 improved gradually. One patient, after initial improvement, worsened by cycle 4. All patients showed stable disease (SD) per RANO criteria on MRI after 3 and 6 cycles. 8/9 patients with seizure reduction showed reduced PET uptake by cycle 3 (mean changes: SUVmax −6.3%; TBRmax −7.5%; MTV −4.7 cm^3^) and 6 (SUVmax −20.9%; TBRmax −10.9%; MTV −35.9 cm^3^), translating into 3 partial responses and 5 SD by PET RANO. All 11 patients with durable seizure response showed reduced D2HG at cycle 3 (mean change: −51%) and cycle 6 (−60%). In contrast, the only patient, who experienced seizure worsening over time, showed D2HG reduction at cycle 3 (−53%), but no further reduction, and PET progression disease, at cycle 6.

**Conclusion:** These findings suggest that F‐DOPA PET and D2HG plasma levels may correlate with seizure improvement after vorasidenib.

**Disclosure:** F.B., A.P, R.R.: Servier (Honoraria for Preceptorships) All other Authors: nothing to disclose.

## OPR‐013

### Phase I trial of a single intratumoral injection of the oncolytic adenovirus DNX‐2440 in recurrent glioblastoma

#### J. Gallego Perez‐Larraya ^1^; N. Casares^2^; M. Giraldez^1^; M. Serrano^1^; B. Bejarano^1^; P. Dominguez^1^; C. Mbongo^1^; M. Gonzalez‐Huarriz^2^; C. Honorato^1^; A. Martinez‐Simon^1^; J. Fueyo^3^; C. Gomez‐Manzano^3^; I. Ausejo‐Mauelon^2^; A. Patiño^1^; M. Alonso^2^

##### ^*1*^ *Department of Neurology, Cancer Center Clínica Universidad de Navarra, Pamplona, Spain;* ^*2*^ *Program in Solid Tumors, Center for Applied Medical Research, Pamplona, Navarra, Spain;* ^*3*^ *Department of Neuro‐Oncology, The University of Texas MD Anderson Cancer Center, Houston, USA*

**Background and Aims:** Oncolytic immunovirotherapy is an emerging therapeutic approach in neuro‐oncology. DNX‐2401, a tumour‐selective oncolytic adenovirus with enhanced infectivity, has demonstrated clinical activity in adults with recurrent glioblastoma. DNX‐2440 is a next‐generation construct incorporating the OX40‐ligand to enhance antitumour immune responses through T cell co‐stimulation and modulation of the immunosuppressive tumour microenvironment. The primary objective was to evaluate the safety and feasibility of DNX‐2440, with secondary exploration of preliminary clinical activity.

**Methods:** This open‐label, single‐centre, phase I dose‐escalation study used a 3 + 3 design in patients with first or second glioblastoma recurrence. Participants underwent stereotactic biopsy followed by single intratumoral DNX‐2440 administration. Two dose levels were evaluated: 4 × 10^9^ and 4 × 10^10^ viral particles. Eligibility required a single measurable lesion of at least 10 mm in two perpendicular diameters and a maximum tumour volume of 25 cc.

**Results:** Sixteen patients were enrolled, including 6 women and 10 men, with a median age of 55 years (range 39–80) and a median KPS of 80 (range 70–90). No dose‐limiting toxicities occurred during escalation, with 3 patients treated per dose level. Remaining patients received 4 × 10^10^ vp in the expansion cohort. DNX‐2440 was well tolerated, with no treatment‐related serious adverse events. Clinical benefit, including tumour reduction and prolonged survival, was observed in 3 patients. Correlative studies assessed systemic immune responses and characterised the tumour immune microenvironment.

**Conclusion:** Single intratumoral DNX‐2440 administration is feasible, with a favourable safety profile and hints of antitumour activity, supporting further investigation of oncolytic virotherapy as an immunomodulatory strategy in glioblastoma.

**Disclosure:** Funded by Instituto de Salud Carlos III (FIS), grant PI23/01155.

## OPR‐014

### Progressive encephalomyelitis with rigidity and myoclonus with glycine receptor antibodies

#### M. Guasp Verdaguer^1^; M. Ruiz‐Vives ^2^; A. Saiz Hinarejos^1^; M. Almendrote Muñoz^3^; J. Bruna Escuer^4^; J. González‐Menacho^5^; J. Kaneko^6^; L. Martín‐Aguilar^7^; F. Martínez‐García^8^; K. Noda^9^; A. Ruiz Molina^10^; S. Sequeiros Fernández^11^; M. Mistieri Simabukuro^12^; M. Takenaka^13^; M. Zurdo Hernández^14^; J. Dalmau Obrador^15^; T. Iizuka^6^; F. Graus Ribas^16^

##### ^*1*^ *Neurology, Hospital Clínic i Provincial de Barcelona, Barcelona, Spain. Neuroimmunology Program, Institut d'Investigació Biomèdica August Pi i Sunyer (IDIBAPS), Barcelona, Spain;* ^*2*^ *Neurology, Hospital Clínic i Provincial de Barcelona, Barcelona, Spain;* ^*3*^ *Neuroscience, Hospital Universitari Germans Trias i Pujol, Barcelona, Spain;* ^*4*^ *Neuro‐Oncology, Hospital Universitari de Bellvitge‐ICO l'Hospitalet, IDIBELL (Oncobell Program), Barcelona, Spain;* ^*5*^ *Neurology, Hospital Universitari de Sant Joan, Institut d'Investigació Sanitària Pere Virgili, Reus, Spain;* ^*6*^ *Neurology, Kitasato University School of Medicine, Sagamihara, Japan;* ^*7*^ *Neurology, Hospital de la Santa Creu i Sant Pau, Barcelona, Spain. Neurological Diseases, Institut de Recerca Sant Pau, Barcelona, Spain;* ^*8*^ *Neurology, Hospital Clínico Universitario Virgen de la Arrixaca, Murcia, Spain;* ^*9*^ *Neurology, Juntendo University Shizuoka Hospital, Izunokuni, Japan;* ^*10*^ *Neurology, Hospital Universitario Puerta de Hierro Majadahonda, Madrid, Spain;* ^*11*^ *Neurology, Hospital Alvaro Cunqueiro, Vigo, Spain;* ^*12*^ *Neurology, Hospital das Clinicas, Faculty of Medicine, University of São Paulo, São Paulo, Brazil;* ^*13*^ *Neurology, Chugoku Rosai Hospital, Kure, Hiroshima, Japan;* ^*14*^ *Neurology, Hospital Virgen del Puerto, Plasencia, Spain;* ^*15*^ *Neurology, Hospital Clínic i Provincial de Barcelona, Barcelona, Spain. Neuroimmunology Program, Institut d'Investigació Biomèdica August Pi i Sunyer (IDIBAPS), Barcelona, Spain, Neurology, University of Pennsylvania, Philadelphia, USA;* ^*16*^ *Neuroimmunology Program, Institut d'Investigació Biomèdica August Pi i Sunyer (IDIBAPS), Barcelona, Spain*

**Background and Aims:** This study aimed to describe clinical features and long‐term outcomes of patients with glycine receptor (GlyR) antibody–mediated progressive encephalomyelitis with rigidity and myoclonus (PERM).

**Methods:** We retrospectively analyzed 41 patients with PERM and GlyR antibodies (22 diagnosed in our laboratory, 19 previously reported patients identified through a systematic literature review) with sufficient clinical data and ≥12 months of follow‐up. Neurologic disability was assessed using the modified Rankin Scale (mRS). Relapses were defined as events occurring >6 months after onset requiring immunotherapy.

**Results:** Median age was 58 years (IQR: 43–66 years), and 36 (88%) were male. Median time from symptom onset to admission was 2 weeks (IQR: 1–4 weeks). Predominant presentations included brainstem symptoms, mainly dysphagia and trismus, in 23 patients (56%); muscle stiffness and myoclonus in 9 (22%); dysesthesias or pruritus in 7 (17%); and cacosmia with dysgeusia in 2 (5%). Five patients (12%) never developed stiffness. All received immunotherapy. Eleven patients died, 8 (20%) from complications of PERM. Ten patients relapsed but responded well to immunotherapy. At last follow‐up [median time 24 months (IQR: 18–72 months)] 23 (70%) of the 33 patients who did not die from PERM presented good functional status (mRs score <3). Age and admission to the intensive care unit independently predicted poor outcomes (mRS score ≥3).

**Conclusion:** GlyR antibody–mediated PERM is a rapidly progressive and severe disease that predominantly affects men and frequently presents with brainstem involvement. Its distinct demographic and clinical features support considering it separately from stiff‐person spectrum disorders.

**Disclosure:** J. Dalmau receives royalties from Athena Diagnostics for the use of Ma2 as an autoantibody test and from Euroimmun for the use of NMDA, GABAB receptor, GABAA receptor, DPPX, and IgLON5 asautoantibody testsand has received an unrestricted research grant from Euroimmun. F. Graus holds a patent licensed to Euroimmun for the use of IgLON5 in an autoantibody test, for which he receives royalties and receives honoraria from MedLink Neurology for his role as associate editor. The other authors report no relevant disclosures.

## OPR‐015

### Reinforcing peritumoral GABAergic inhibition modulates glioma progression and enhances immunotherapy efficacy

#### M. Scalera ^1^; E. De Santis^2^; F. Rossi^1^; N. Meneghetti^3^; A. Mazzoni^3^; A. Flori^4^; F. Raimondi^2^; P. Miglionico^2^; M. Pasqualetti^5^; M. Costa^1^; E. Vannini^1^

##### ^*1*^ *Italian National Research Council (CNR), Neuroscience Institute, Pisa, Italy;* ^*2*^ *Scuola Normale Superiore, Pisa, Italy;* ^*3*^ *Scuola Superiore Sant'Anna, Pisa, Italy;* ^*4*^ *Bioengineering Unit, Fondazione Gabriele Monasterio Regione Toscana CNR;* ^*5*^ *University of Pisa, Biology Department, Pisa, Italy*

**Background and Aims:** Gliomas disrupt cortical excitatory–inhibitory balance, contributing to neurological dysfunction and tumor progression. Parvalbumin (PV)‐expressing GABAergic interneurons are key regulators of network stability within the peritumoral microenvironment. This study aimed to determine whether chemogenetic modulation of peritumoral PV‐interneurons and pharmacological enhancement of GABAergic signaling influence glioma growth, cortical function, and survival in murine models.

**Methods:** PV‐Cre mice were implanted with GL261 glioma cells. To selectively modulate peritumoral PV‐interneurons, AAV‐hSyn‐DIO‐hM4D(Gi)‐mCherry or AAV‐hSyn‐DIO‐hM3D(Gq)‐mCherry were bilaterally injected into the motor cortex adjacent to the tumor site. From day 12 to day 22 post–tumor induction, mice received clozapine‐N‐oxide (CNO) or vehicle twice daily. As readouts, we evaluated tumor volume by magnetic resonance imaging, longitudinal motor performance, and animal survival. To evaluate a pharmacological strategy, GL261‐ and CT‐2A–bearing mice were treated with the GABAB receptor agonist baclofen, alone or combined with anti–PD‐L1 immunotherapy. In vivo and in vitro assays were performed to assess direct drug effects.

**Results:** PV‐interneuron inactivation exacerbated neurological symptoms and epileptiform activity without affecting tumor size or survival. Conversely, PV‐interneuron activation reduced tumor proliferation, preserved motor and network function, and partially maintained visual acuity and contrast sensitivity. Baclofen modestly reduced tumor volume and motor decline in GL261 but not CT‐2A gliomas, consistent with indirect microenvironment‐mediated effects. Notably, baclofen combined with anti–PD‐L1 therapy synergistically reduced tumor burden, achieved complete tumor eradication in 66% of mice, and significantly extended survival.

**Conclusion:** Enhancing peritumoral GABAergic inhibition stabilizes cortical networks, limits glioma progression, and potentiates immunotherapy, representing a promising adjuvant strategy for glioma treatment.

**Disclosure:** Nothing to disclose.

## OPR‐016

### Vascular complications in IDH‐wildtype glioblastoma: Incidence, timing, and clinical impact

#### T. Urbanic Purkart; M. Lochmann; T. Gattringer

##### Neurology, Medical University of Graz, Graz, Austria

**Background and Aims:** Patients with high‐grade glioma (HGG) are at elevated risk for venous thromboembolism (VTE) and cerebrovascular events; however, the frequency, timing, and underlying mechanisms remain insufficiently characterized. This study aimed to determine the incidence and potential risk factors for vascular complications in a well‐defined glioblastoma cohort.

**Methods:** We retrospectively analyzed 337 consecutive patients with histopathologically confirmed IDH‐wildtype glioblastoma (WHO 2021) treated at the neuro‐oncological outpatient clinic of University Hospital Graz. Demographic data, treatment characteristics, and vascular events were systematically collected using the electronic hospital documentation system, covering all acute vascular events within the regional catchment area.

**Results:** Vascular complications occurred in 25.2% of patients (*n* = 85), including VTE (18.4%), ischemic stroke (4.2%), intracerebral hemorrhage (3.6%), cerebral sinus venous thrombosis (0.6%), and myocardial infarction (0.6%). Most events occurred early: 31.8% within 1 month and 74.1% within 6 months of diagnosis; 22.4% occurred during first‐line therapy. Vascular events were associated with a case fatality rate of 12.9%. Complications were treated according to current guidelines; VTE was mainly managed with low‐molecular‐weight heparins and, less frequently, with direct oral anticoagulants, without major intracerebral bleeding. Overall survival did not differ significantly between patients with and without vascular events, and no analyzed risk factor was significantly associated with outcome. TP53 mutations were less frequent in patients with vascular events.

**Conclusion:** Incidence rates were consistent with previous retrospective studies. The temporal clustering of events in the postoperative period and during radiochemotherapy confirms known high‐risk phases. Vascular complications may affect treatment continuity and increase case fatality rates, warranting prospective risk stratification studies.

**Disclosure:** Nothing to disclose.

## MS and Related Disorders 1

## OPR‐017

### Comparative effectiveness of IV pulses cyclophosphamide versus siponimod on disability accrual in secondary progressive multiple sclerosis

#### A. Mazzeo ^1^; A. Di Sapio^2^; A. Nuti^3^; E. Torrini^3^; F. Granella^4^; M. Ulivelli^5^; M. Salvetti^6^; M. Piscaglia^7^; L. Pasquali^8^; G. Lus^9^; M. Falcini^10^; A. Lugaresi^11^; C. Tortorella^12^; F. Meatti^13^; A. Mattei^14^; M. Di Cristinzi^1^; A. Repice^1^; A. Mariottini^1^; L. Massacesi^1^

##### ^*1*^ *Department of Neurology 2 and Tuscan Region MS Referral Centre, Careggi University Hospital, Florence, Italy;* ^*2*^ *Regional Multiple Sclerosis Referral Centre (CRESM), Neurology Department, San Luigi Gonzaga University Hospital, Orbassano, Italy;* ^*3*^ *Department of Statistics, Computer Science, Applications “Giuseppe Parenti”, University of Florence, Florence, Italy;* ^*4*^ *Multiple Sclerosis Centre, Neurology Operative Unit, Department of General and Specialized Medicine, University Hospital of Parma;* ^*5*^ *Department of Medical Surgical Sciences and Neurosciences, University of Siena;* ^*6*^ *Scientific Institute for Research, Hospitalization and Healthcare (IRCCS), Mediterranean Neurological Institute Neuromed, Pozzilli, Italy;* ^*7*^ *Department of Neurosciences, Multiple Sclerosis Centre, Neurology Operative Unit, S. Maria delle Croci Hospital, AUSL Romagna, Italy;* ^*8*^ *Demyelinating Diseases Centre, Neurology Operative Unit, Department of Clinical and Experimental Medicine, University of Pisa, Neuroscience Department, Pisa University Hospital;* ^*9*^ *Clinical Centre for Multiple Sclerosis, II Neurology Clinic, University of Campania “L. Vanvitelli”;* ^*10*^ *Neurology Unit, Department of Medical Specialties, Azienda USL Toscana Centro, Prato, Italy;* ^*11*^ *IRCCS Institute of Neurological Sciences of Bologna, Multiple Sclerosis Rehabilitation Operative Unit, Bologna, Italy;* ^*12*^ *Department of Neurosciences, San Camillo Forlanini Hospital, Rome, Italy;* ^*13*^ *Department of Economics, European University Institute,* ^*14*^ *Florence Centre for Data Science, Florence, Italy*

**Background and Aims:** Effectiveness of available disease‐modifying treatments (DMTs) on disability progression in multiple sclerosis (MS) is marginal, hence therapeutic options for secondary progressive (SP) MS are limited to Siponimod (SIP) in Europe. Cyclophosphamide (CY), a small highly immunosuppressive molecule with high blood‐brain‐barrier penetration, is a potential optimal treatment, although yet never compared to SIP.

**Methods:** SPMS patients recruited at the Tuscany region MS Referral Centre of the Careggi University‐Hospital (Florence, Italy) treated with intravenous CY‐pulses according to a standardized protocol were included as index‐cases and compared to SIP‐treated controls from the same MS Centre or extracted from the Italian Multiple Sclerosis Register. To correct for unbalances between the two groups, an overlap weighting approach based on propensity scores was used, censoring results at month 36. The primary endpoint was disability Worsening‐Free Survival (WFS). Key secondary endpoints were Progression‐Free Survival (PFS), No‐Evidence of Disease Activity‐2 (NEDA‐2) and Time To First‐Relapse (TTFR). Safety outcomes included mortality for any cause and incidence of neoplasms. Disability outcomes were assessed through Cox regression models.

**Results:** 123 CY‐treated and 81 SIP‐treated SPMS patients were included thus far. WFS was remarkably higher in the CY‐treated compared to the SIP‐treated cohort (Survival Probability Causal Effect, SPCE, close to significance, Figure 1); NEDA‐2 (Figure 2) and disability improvement were significantly more frequently obtained and TTFR delayed (Figure 3) in the CY‐cohort. No difference was observed in terms of safety.

**Figure:**
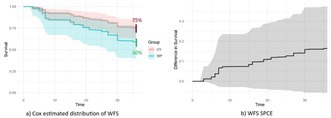
**FIGURE 1** (a) Cox estimated distribution of WFS following treatment with Cyclophosphamide (CY; red) or Siponimod (SIP; green). WFS at Year 3 was 75% in CY and 60% in SIP group. (b) WFS SPCE was positive and close to significance.

**Figure:**
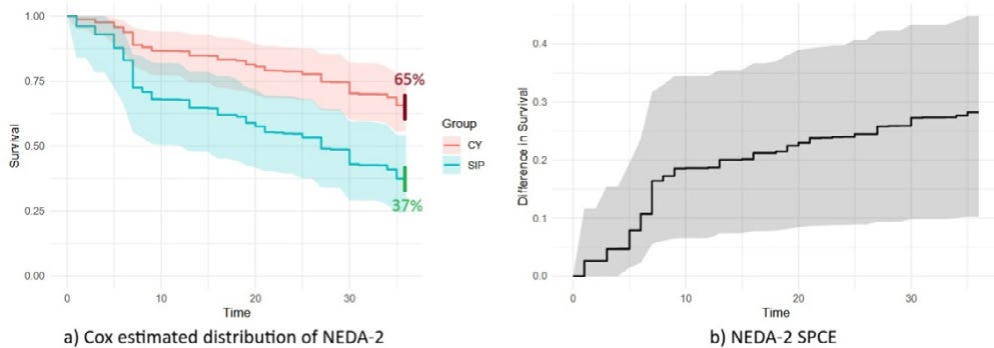
**FIGURE 2** (a) Cox estimated distribution of NEDA‐2 following treatment with Cyclophosphamide (CY; red) or Siponimod (SIP; green). NEDA‐2 at Year 3 was 65% in CY and 37% in SIP group. (b) NEDA‐2 SPCE was positive and statistically significant

**Figure:**
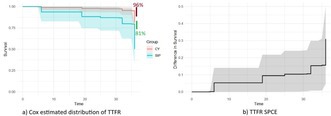
**FIGURE 3** (a) Cox estimated distribution of TTFR following treatment with Cyclophosphamide (CY; red) or Siponimod (SIP; green). TTFR at Year 3 was 96% in CY and 81% in SIP group. (b) TTFR SPCE was positive and statistically significant.

**Conclusion:** The preliminary analysis of the first three years of this study in SPMS suggests a superior effectiveness of CY vs. SIP on disability progression and NEDA‐2.

**Disclosure:** Nothing to disclose.

## OPR‐018

### Radiological progression in pre‐symptomatic multiple sclerosis: The role of paramagnetic rim lesions beyond 2024 McDonald diagnostic criteria

#### E. Barbuti ^1^; S. Borrelli^2^; F. Guisset^3^; F. London^4^; S. El Sankari^5^; E. Lommers^6^; V. Van Pesch^7^; P. Maggi^8^

##### ^*1*^ *Neuroinflammation Imaging Lab (NIL), Institute of NeuroScience, Université catholique de Louvain, Brussels, Belgium; Department of Human Neurosciences, Sapienza University, Rome, Italy;* ^*2*^ *Neuroinflammation Imaging Lab (NIL), Institute of NeuroScience, Université catholique de Louvain, Brussels, Belgium; Department of Neurology, Hôpital Erasme, Hôpital Universitaire de Bruxelles, Université Libre de Brussels, Brussels, Belgium;* ^*3*^ *Neuroinflammation Imaging Lab (NIL), Institute of NeuroScience, Université catholique de Louvain, Brussels, Belgium;* ^*4*^ *Department of Neurology, CHU UCL Namur site Godinne, Université catholique de Louvain, Yvoir, Belgium;* ^*5*^ *Department of Neurology, Cliniques Universitaires Saint‐Luc, Université catholique de Louvain, Brussels, Belgium;* ^*6*^ *GIGA‐CRC In vivo Imaging, University of Liège, Liège, Belgium; Department of Neurology, Centre Hospitalier Universitaire de Liège, Liège, Belgium;* ^*7*^ *Laboratory of Neurochemistry, Institute of Neuroscience, Université Catholique de Louvain, Brussels, Belgium; Department of Neurology, Cliniques Universitaires Saint‐Luc, Université catholique de Louvain, Brussels, Belgium;* ^*8*^ *Neuroinflammation Imaging Lab (NIL), Institute of NeuroScience, Université catholique de Louvain, Brussels, Belgium; Department of Neurology, Cliniques Universitaires Saint‐Luc, Université catholique de Louvain, Brussels, Belgium*

**Background and Aims:** The 2024 McDonald criteria allow multiple sclerosis (MS) diagnosis in pre‐symptomatic individuals, though its clinical significance remains uncertain. We assessed whether paramagnetic rim lesions (PRL), and other conventional biomarkers of disease progression improve radiological progression prediction beyond diagnostic criteria.

**Methods:** We retrospectively evaluated patients with incidental MRI findings suggestive of MS and at least two MRIs. Demographic, clinical, baseline MRI features, PRL presence, central vein sign (CVS) Select6 and CSF positivity, optic nerve involvement, and treatment were collected. Time to first radiological progression (new T2 or gadolinium‐enhancing lesions) was analyzed using multivariable Cox model, including variables significant in univariable analyses. A random forest model assessed feature importance, considering variables with univariable logistic regression *p* ≤ 0.2. Logistic regressions and ROC analyses evaluated whether MS classification according to 2024 McDonald criteria, alone or combined with PRL presence, predicted radiological progression. McNemar's test compared differences in binary classifications.

**Results:** Sixty patients were included (mean age 38.3 (10.4) years; 78% female; mean radiological follow‐up 50 months). Eighteen patients showed radiological progression (median 83 months). Among variables associated with time to first radiological progression (Table 1), only PRL presence remained significant in the multivariable Cox analysis. The most important radiological progression predictors were PRL presence, longer radiological follow‐up, CVS Select6 and CSF positivity (AUC = 0.91, specificity = 0.9, sensitivity = 0.72) (Figure 1). Adding PRL presence to McDonald criteria positivity improved the prediction (*R* ^2^ = 0.3, AUC = 0.8 vs. *R* ^2^ = 0.2, AUC = 0.75; *p* < 0.001) (Figure 2).

**TABLE 1** Univariable and multivariable analyses for time to first radiological progression.

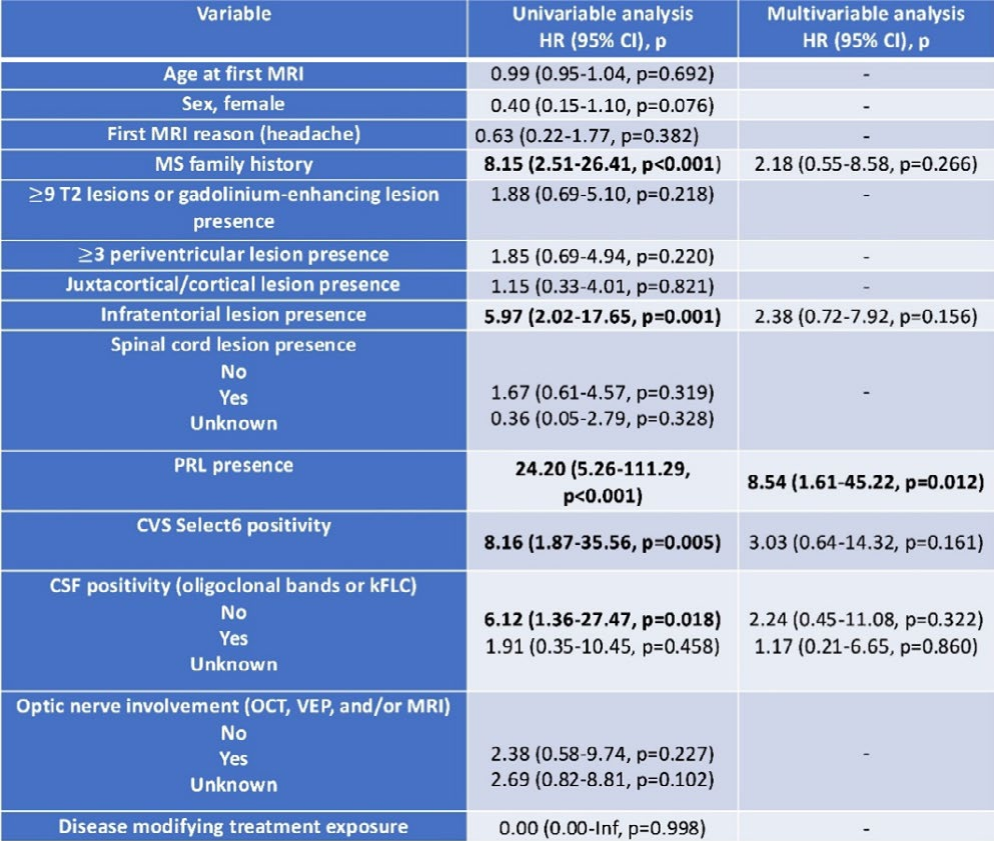

**Figure:**
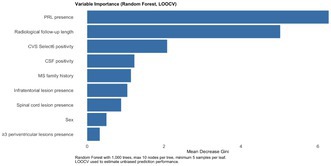
**FIGURE 1** Variable importance for radiological progression prediction assessed with a Leave‐one‐out cross validation (LOOCV) Random Forest Model.

**Figure:**
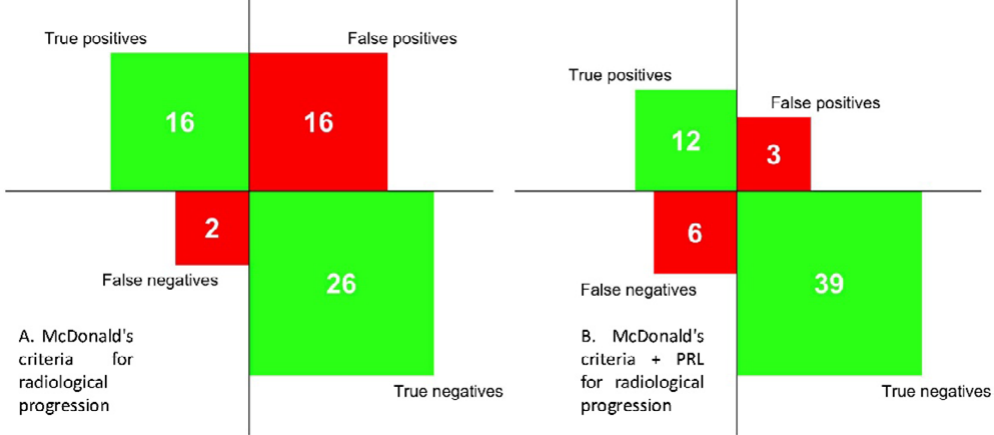
**FIGURE 2** Confusion matrix testing whether MS diagnosis according to McDonald criteria (A) or MS diagnosis according to McDonald criteria + paramagnetic rim lesions (PRL) (B) predicts radiological progression in patients with pre‐symptomatic MS.

**Conclusion:** PRL are associated with radiological progression, providing complementary information beyond biomarkers currently included in 2024 McDonald criteria for pre‐symptomatic MS.

**Disclosure:** Disclosures: E.B. was awarded an ECTRIMS clinical fellowship 2025. S.B. is supported by the Funds Claire Fauconnier, Ginette Kryksztein & José and Marie Philippart‐Hoffelt, managed by the King Baudouin Foundation and received speaker/consulting honoraria and/or travel grants from Sanofi, Roche, Janssen, Merck, Novartis, Alexion and Amgen, and research grants from Roche, Sanofi, and Brugmann Foundation. S.E.S received grants and/or travel and/or consultancy fees from Biogen, Merck Serono, Sanofi, Roche, Novartis, Teva Pharma, and Bayer Schering. E.L. received travel and consultancy fees from Sanofi, Biogen, Merck Serono and Roche and is supported by National Fund for Scientific Research (FRS‐FNRS), Belgium and received research funding from the Belgian Charcot Foundation, the King Baudouin Foundation as well as from the MS Belgian National League. V.V.P. received consulting honoraria and research grants from Biogen, Merck, Sanofi, BMS, Novartis, Janssen, Almirall, Roche, Alexion, Medtronic, and Teva Pharmaceuticals. P.M. research activity is supported by the Fondation Charcot Stichting Research Fund 2023, the Fund for Scientific Research (F.R.S, FNRS; grant #40,008,331), Cliniques universitaires Saint‐Luc “Fonds de Recherche Clinique” and Biogen and received consulting honoraria from Sanofi, Biogen and Merck. F.G. and F.L. have nothing to disclose.

## OPR‐019

### Abnormal B‐cell exosome pro‐ and anti‐apoptotic cargo in multiple sclerosis: Implication in progressive disease biology

#### G. Breville ^1^; A. Rezk^1^; S. Weissman^1^; D. Espinoza^1^; S. Thebault^1^; L. Yamashita^1^; Y. Kim^1^; M. Kakara^2^; L. Nedelkoska^3^; N. Doyon‐Reale^3^; C. Delucinge‐Vivier^4^; L. Zuroff^5^; H. Touil^6^; P. Stemmer^3^; J. Benjamins^3^; R. Lisak^3^; A. Bar‐Or^1^

##### ^*1*^ *Center for Neuroinflammation and Experimental Therapeutics, Perelman School of Medicine, University of Pennsylvania, Philadelphia, USA;* ^*2*^ *Department of Neurology, NYU, NYU Langone Health, Grossman School of Medicine, New York, NY;* ^*3*^ *Department of Neurology, Wayne State University School of Medicine, Detroit, USA;* ^*4*^ *iGE3 Genomics Platform, University of Geneva, 1 Rue Michel‐Servet, Geneva, Switzerland;* ^*5*^ *UCSF Division of Neuroimmunology & Glial Biology;* ^*6*^ *Center for Translational & Computational Neuroimmunology, Department of Neurology, Columbia University Irving Medical Center, New York, USA*

**Background and Aims:** Compartmentalized central nervous system (CNS) inflammation involving B cells is implicated in gray matter injury and disease progression in multiple sclerosis (MS). Products secreted by B cells of MS patients can kill oligodendrocytes and neurons, a cytotoxicity conferred by their exosome‐enriched extracellular vesicle (Ex‐En) fraction.

**Methods:** To explore the potential molecular mediators of this cytotoxicity, we profiled proteomic and transcriptomic cargo of Ex‐En isolated from B cells of treatment‐naive MS patients and matched healthy controls.

**Results:** MS B cell‐derived Ex‐En appeared enriched in cell‐death associated proteins (including fibrinogen, complement C9, APP, and SPARC) and deficient in cell‐survival associated proteins (such as galectin‐3). Abnormal enrichment for cell‐death proteins was supported by Gene Set Enrichment Analysis. Protein pathway analysis revealed densely connected pro‐death modules in the MS B cell‐derived EVs, contrasting with homeostatic signatures in controls. Transcriptomic analysis further revealed that Ex‐En of MS B cells appeared to carry reduced levels of miRNAs (miR‐182, miR‐212, miR‐1270) known to inhibit apoptosis.

**Figure:**
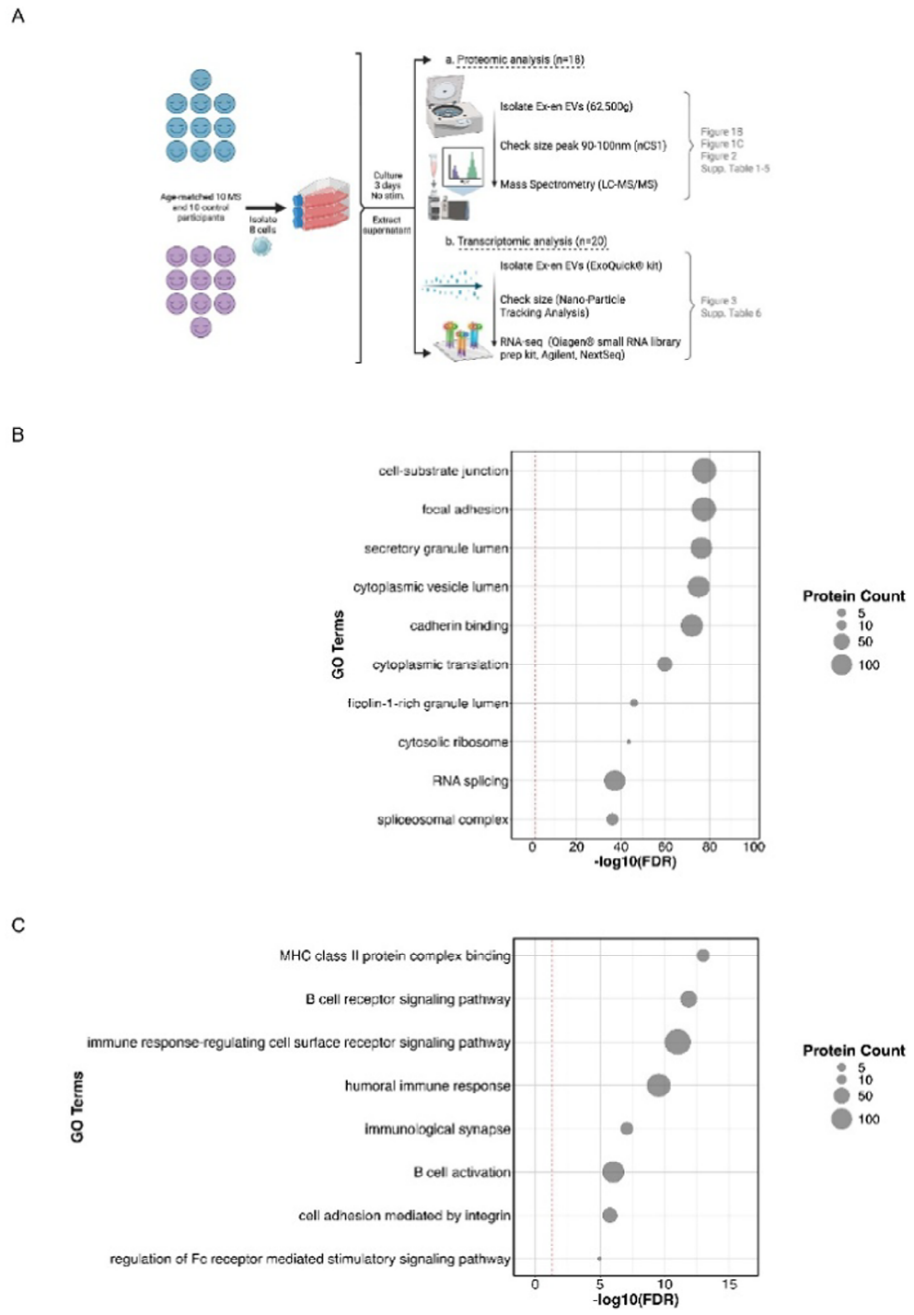
**FIGURE 1** Proteomic Gene Ontology analysis of normal human B cell‐derived exosomes identifies enrichment of vesicular elements, immune pathways and complexes implicated in inter‐cellular communication.

**Figure:**
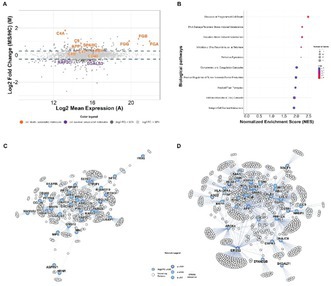
**FIGURE 2** Abnormal enrichment of cell‐death pathways in B cell‐derived exosomes of MS patients.

**Figure:**
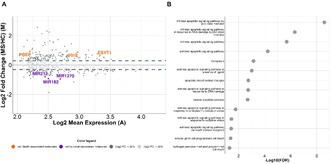
**FIGURE 3** Deficiency of anti‐apoptotic miR‐182, miR‐212, and miR‐1270 in B cell‐derived exosomes of MS patients.

**Conclusion:** Our findings indicate that B cell‐derived exosomes of MS patients, previously shown to impair neuronal and glial survival, harbor an abnormal cytotoxic molecular profile that may contribute to CNS‐compartmentalized injury and progressive MS biology.

**Disclosure:** Nothing to disclose.

## OPR‐020

### Disentangling age‐related and disease‐specific upper cervical cord atrophy in multiple sclerosis

#### K. Jain ^1^; L. Storelli^1^; P. Valsasina^1^; P. Preziosa^2^; A. Meani^1^; P. Pantano^3^; C. Piervincenzi^4^; A. Gallo^5^; A. d'Ambrosio^5^; N. De Stefano^6^; R. Cortese^6^; M. Filippi^7^; M. Rocca^2^

##### ^*1*^ *Neuroimaging Research Unit, Division of Neuroscience, IRCCS San Raffaele Scientific Institute, Milan, Italy;* ^*2*^ *Neuroimaging Research Unit, Division of Neuroscience, and Neurology Unit, IRCCS San Raffaele Scientific Institute, Milan, Italy; and Vita‐Salute San Raffaele University, Milan, Italy;* ^*3*^ *Department of Human Neuroscience, Sapienza University, Rome, Italy; and IRCCS Neuromed, Pozzilli (IS), Italy;* ^*4*^ *Department of Human Neuroscience, Sapienza University, Rome, Italy;* ^*5*^ *Department of Advanced Medical and Surgical Sciences, University of Campania Luigi Vanvitelli, Naples, Italy;* ^*6*^ *Department of Medicine, Surgery and Neuroscience, University of Siena, Siena, Italy;* ^*7*^ *Neuroimaging Research Unit, Division of Neuroscience, Neurology Unit, Neurorehabilitation Unit, and Neurophysiology Service, IRCCS San Raffaele Scientific Institute, Milan, Italy; and Vita‐Salute San Raffaele University, Milan, Italy*

**Background and Aims:** This study aimed to model lifespan trajectories of upper cervical cord atrophy in healthy controls (HC) and identify multiple sclerosis (MS)‐specific patterns of cord atrophy in a large multicenter cohort.

**Methods:** We analyzed 3D T1‐weighted brain MRIs and clinical data from 480 HC and 1295 MS patients. Mean upper cervical cord cross‐sectional area (MUCCA) was measured between C1 and C2/3 intervertebral disk using the active surface method and normalized for head size (nMUCCA). nMUCCA was modelled across the lifespan in HC using linear regression accounting for age, age^2^, sex, scanner and interactions, and thereafter, applied to the MS cohort to derive nMUCCA *Z*‐scores. Analyses were stratified by sex and age at onset (pediatric‐onset [POMS], adult‐onset [AOMS], late onset [LOMS]).

**Results:** In HC, nMUCCA showed a non‐linear relationship with age, increasing until the late 30s (*p* ≤ 0.033) and declining after age 50 (*p* ≤ 0.008). In MS, nMUCCA *Z*‐scores decreased up to late 40s (*p* ≤ 0.001). Sex did not significantly influence atrophy patterns in either group. POMS patients exhibited significantly lower *Z*‐scores compared to AOMS and LOMS (*p* < 0.001), although age‐related MS‐driven decline did not differ among onset groups. Lower nMUCCA *Z*‐scores were associated with longer disease duration and greater clinical disability (all *p* < 0.001).

**Conclusion:** In MS, upper cervical cord atrophy exceeded effects of normal ageing, especially during midlife, independent of the effect of sex and age at disease onset, and was associated with longer disease duration and disability. Upper cord atrophy holds promise as a sensitive biomarker for tracking MS progression beyond ageing effects.

**Disclosure:** Supported by FISM—Fondazione Italiana Sclerosi Multipla—cod. 2025/S/02 and financed or co‐financed with the ‘5 per mille’ public funding. KJ was funded by the Multiple Sclerosis International Federation (MSIF) and ECTRIMS in the form of MSIF‐ECTRIMS McDonald Fellowship. PV, AM, AdA, CP have nothing to disclose. LS received speakers' honoraria from Biogen. PP received speaker honoraria from Roche, Biogen, Novartis, Merck, Bristol Myers Squibb, Genzyme, Horizon and Sanofi. PPa has received funding for travel from Novartis, Genzyme, and Bracco and a speaking honorarium from Biogen. She received research support from Italian Ministry of Foreign Affairs and FISM. AG received speaker and consulting fees from Biogen, Genzyme, Merck Serono, Mylan, Novartis, Roche, and Teva, and receives research support from FISM. NDS has received honoraria from Biogen‐Idec, Bristol Myers Squibb, Celgene, Genzyme, Immunic, Merck Serono, Novartis, Roche and Teva for consulting services, speaking, and travel support. He serves on advisory boards for Merck, Novartis, Biogen‐Idec, Roche, and Genzyme, Immunic and he has received research grant support from FISM. RC was awarded a MAGNIMS‐ECTRIMS fellowship in 2019; she received speaker honoraria from Roche, Merck Serono and Sanofi and travel support for conferences by Novartis. MF is Editor‐in‐Chief of the Journal of Neurology, Associate Editor of Human Brain Mapping, Neurological Sciences, and Radiology; received fees for consultancies from Almirall, Biogen, Bristol‐Myers Squibb, Eli Lilly, Merck, Novartis, Roche, Sanofi; speaking activities from Amgen, Bayer, Biogen, Bristol‐Myers Squibb, Celgene, Chiesi Italia SpA, Eisai, Eli Lilly, Fujirebio, Genzyme, Janssen, Merck, Neopharmed Gentili, Neuraxpharm, Novartis, Novo Nordisk, Roche, Sanofi, Takeda; participation in Advisory Boards for Alexion, Biogen, Bristol‐Myers Squibb, Eli Lilly, GE Healthcare Ltd, Merck, Neuraxpharm, Novartis, Roche, Sandoz, Sanofi, Takeda; scientific direction of educational events for Biogen, Merck, Roche, Celgene, Bristol‐Myers Squibb, Lilly, Novartis, Sanofi‐Genzyme; research support from Biogen Idec, Merck‐Serono, Novartis, Roche, the Italian Ministry of Health, the Italian Ministry of University and Research, and FISM. MAR received consulting fees from Biogen, Bristol Myers Squibb, Roche; and speaker honoraria from Alexion, Biogen, Bristol Myers Squibb, Celgene, Horizon Therapeutics Italy, Merck Serono SpA, Mitsubishi‐Tanabe Pharma, Neuraxpharm, Novartis, Roche, Sandoz, and Sanofi. She receives research support from the MS Society of Canada, the Italian Ministry of Health, the Italian Ministry of University and Research, and FISM. She is Associate Editor for Multiple Sclerosis and Related Disorders, and Associate Co‐Editor for Europe and Africa for Multiple Sclerosis Journal.

## OPR‐021

### Assessing the value of NfL and GFAP for individualized treatment decisions in two independent large observational cohorts

#### M. Einsiedler ^1^; S. Sandgren^1^; S. Schaedelin^1^; K. Ning^2^; A. Maleska Maceski^1^; L. Hofer^1^; M. Hughes^2^; J. Oechtering^1^; C. Cordano^2^; L. Melie‐Garcia^1^; J. Gelfand^2^; A. Cagol^1^; J. Müller^1^; R. Henry^2^; S. Finkener^3^; P. Lalive^4^; G. Breville^4^; S. Müller^5^; C. Pot^6^; A. Mathias^6^; R. Du Pasquier^6^; R. Hoepner^7^; A. Chan^7^; G. Disanto^8^; C. Zecca^8^; L. Hemkens^1^; Ö. Yaldizli^1^; B. Fischer‐Barnicol^1^; T. Derfuss^1^; P. Roth^9^; V. Kana^9^; C. Gobbi^8^; D. Brassat^10^; B. Tackenberg^11^; R. Pedotti^11^; H. Wiendl^12^; G. Arrambide^13^; F. Piehl^14^; H. Zetterberg^15^; B. Cree^2^; M. Sormani^16^; L. Kappos^1^; S. Hauser^2^; M. Khalil^17^; C. Granziera^1^; A. Green^2^; D. Leppert^1^; P. Benkert^1^; A. Abdelhak^2^; J. Kuhle^1^

##### ^*1*^ *Multiple Sclerosis Centre and Research Center for Clinical Neuroimmunology and Neuroscience (RC2NB), Departments of Neurology, Biomedicine and Clinical Research, University Hospital and University of Basel, Basel, Switzerland;* ^*2*^ *Department of Neurology and Weill Institute for Neurosciences, University of California, San Francisco (UCSF), USA;* ^*3*^ *Department of Neurology, Cantonal Hospital Aarau, Aarau, Switzerland;* ^*4*^ *Department of Clinical Neurosciences, Division of Neurology, and Department of Medicine, Translational Biomarker Group, Geneva, Switzerland;* ^*5*^ *Department of Neurology, Cantonal Hospital St. Gallen, St. Gallen, Switzerland;* ^*6*^ *Service of Neurology and Laboratories of Neuroimmunology, Department of Clinical Neurosciences, Lausanne University Hospital (CHUV) and University of Lausanne, Lausanne, Switzerland;* ^*7*^ *Department of Neurology, Inselspital, Bern University Hospital, University of Bern, Bern, Switzerland;* ^*8*^ *Multiple Sclerosis Center (MSC), Department of Neurology, Neurocenter of Southern Switzerland, EOC, 6900 Lugano, Switzerland;* ^*9*^ *Department of Neurology and Clinical Neuroscience Center, University Hospital Zurich and University of Zurich, Zurich, Switzerland;* ^*10*^ *Novartis Pharma AG, Basel, Switzerland;* ^*11*^ *F. Hoffmann‐La Roche Ltd, Basel, Switzerland;* ^*12*^ *Department of Neurology and Neurophysiology, University Medical Center, Freiburg, Germany;* ^*13*^ *Neurology‐Neuroimmunology Department, Multiple Sclerosis Centre of Catalonia (Cemcat), Vall d'Hebron Barcelona Hospital Campus, Barcelona, Spain;* ^*14*^ *Department of Clinical Neuroscience, Karolinska Institutet, Center for Molecular Medicine, Karolinska University Hospital, Stockholm, Sweden;* ^*15*^ *Department of Psychiatry and Neurochemistry, Institute of Neuroscience and Physiology, The Sahlgrenska Academy at the University of Gothenburg, Mölndal, Sweden;* ^*16*^ *Dipartimento di Scienze della Salute, Università degli Studi di Genova, Italy;* ^*17*^ *Department of Neurology, Medical University of Graz, Graz, Austria*

**Background and Aims:** Prognosticating disease progression is difficult in MS. We investigated how serum neurofilament light chain (NfL) and glial fibrillary acidic protein levels (GFAP) can support assessment of progression‐risk and reflect protective effects of disease‐modifying therapy (DMT) against long‐term ‘progression independent of relapse activity’ (PIRA).

**Methods:** Patients from two large real‐world MS cohorts, the Swiss MS Cohort (SMSC) and the EPIC study (University of California, San Francisco), were included. Clinical data and biomarker *Z* scores were collected from 18629 visits at 6/12‐month intervals.

**Results:** SMSC and EPIC consisted of 1709 and 620 patients, yielding 13375 and 5254 samples (Table 1). Elevated NfL (*Z* score >1.5; 93rd percentile) was associated with a 2‐fold risk of relapse within the next year in SMSC (OR 2.00, 95% CI 1.63–2.45; *p* < 0.0001) and a 1.69‐fold risk in EPIC (1.69, 1.24–2.32; *p* = 0.00094, Figure 1), while GFAP was not. Elevated GFAP (*Z* score >1.0; 84th percentile) prognosticated a 45% higher risk of PIRA at next visit in SMSC (1.45, 1.21–1.75; *p* < 0.0001). This was confirmed in EPIC (1.36, 1.07–1.71; *p* = 0.011, Figure 1). In SMSC, every yearly GFAP Z score unit reduction during the first 2 years of fingolimod or B‐cell depleting therapy was associated with a 54% (HR 0.46, 95% CI 0.26–0.84; *p* = 0.012) and 67% (0.33, 0.18–0.61; *p* = 0.00036) lower risk of PIRA (Figure 2), respectively. No such associations were observed for NfL *Z* scores.

**TABLE 1** Characteristics of included patients.

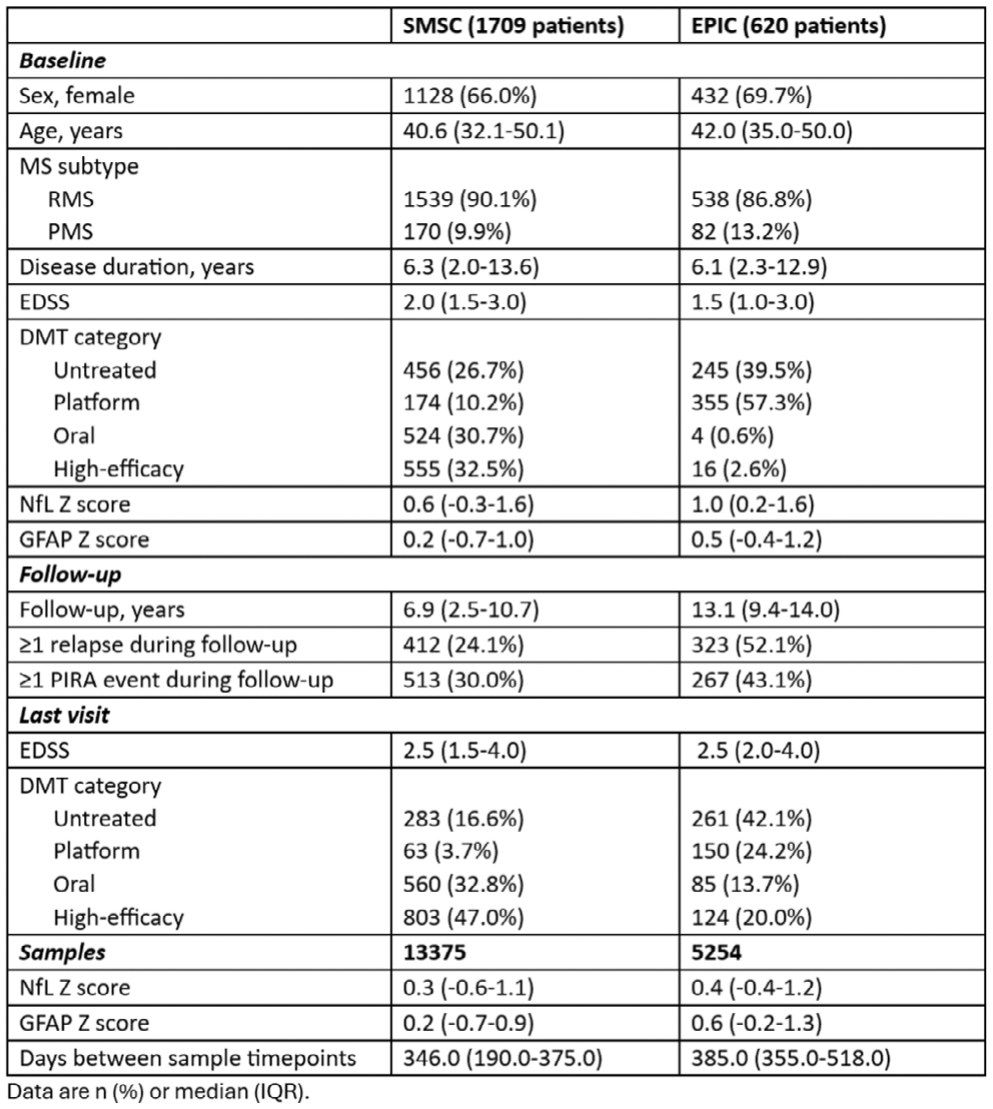

**Figure:**
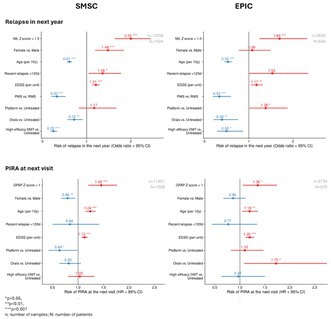
**FIGURE 1** Serum NfL prognosticated subsequent relapses (logistic mixed models, 2.0‐ and 1.69‐fold risk), while serum GFAP was independently associated with progression independent of relapse activity (PIRA; 1.45‐ and 1.36‐fold risk) in both cohorts.

**Figure:**
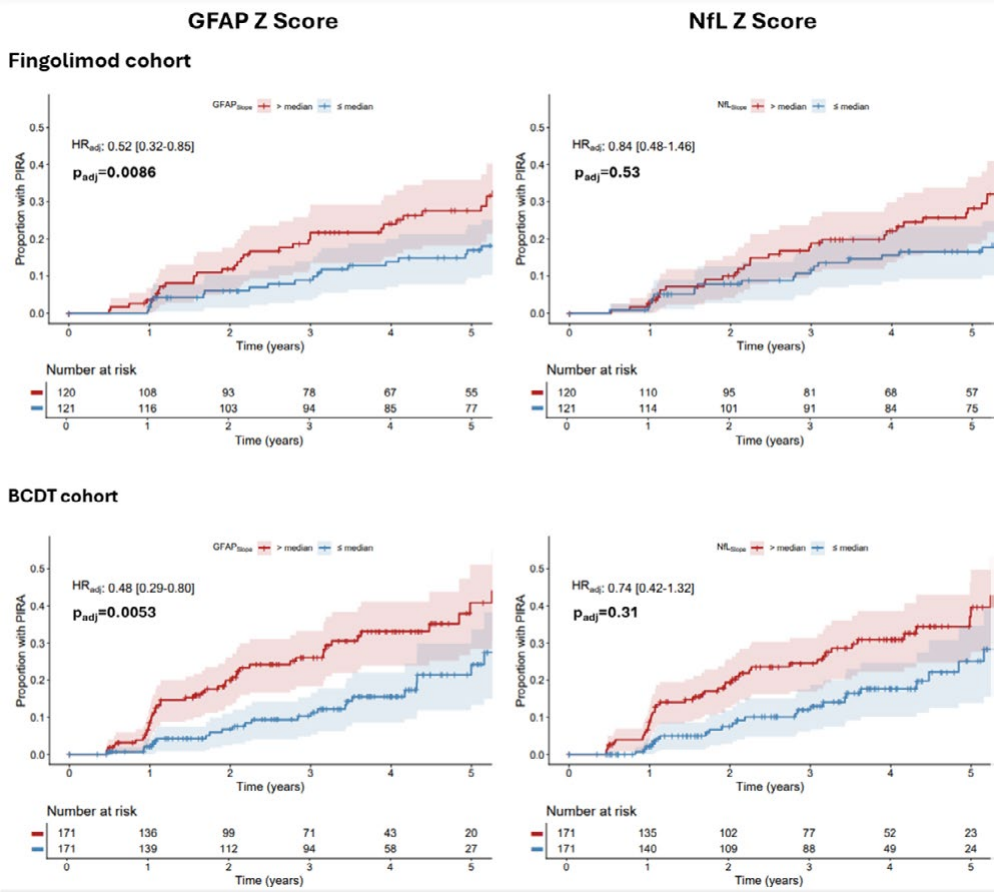
**FIGURE 2** Patients with GFAP *Z* score reduction after DMT start had lower risk of future PIRA than those without. Kaplan‐Meier curves for subsequent PIRA stratified by biomarker slope within 2 years of DMT start. HR/*p* values from multivariable Cox models.

**Conclusion:** NfL and GFAP are powerful complementary tools for prognostication of relapse activity and PIRA in MS. DMT‐associated GFAP‐reduction predicted lower PIRA‐risk. Together, these biomarkers may improve prognostic accuracy and personalized treatment.

**Disclosure:** This investigation was supported by Swiss National Science Foundation (grant 320030_189140), award from Progressive MS Alliance (PA‐2007‐36872), and grant funding from Novartis and Roche. The Swiss MS Cohort study received funding from the Swiss MS Society and grant funding from Biogen, Celgene, Merck, Novartis, Roche, and Sanofi. The EPIC cohort and biomarker assessment was funded through grants R35NS111644 from the National Institutes of Health/National Institute of Neurological Disorders and Stroke, Valhalla Foundation, Westridge Foundation and grant funding from Roche. The UCSF MS biorepository was supported by grant Si‐2001‐35701 from the US National Multiple Sclerosis Society. EPIC biomarker measurements research/study was in part funded by F. Hoffmann‐La Roche Ltd as part of Integrative Neuroscience Collaborations Network.

## Neuroepidemiology

## OPR‐022

### Genetic and early‐life vulnerability in functional neurological disorder: A large‐scale retrospective cohort study

#### E. Ipavic ^1^; T. Pollak^2^; T. Nicholson^2^; M. Edwards^2^; R. Berlot^1^

##### ^*1*^ *Department of Neurology, University Medical Centre Ljubljana, Slovenia;* ^*2*^ *Institute of Psychiatry, Psychology & Neuroscience, King's College London, London, UK*

**Background and Aims:** Contemporary models of functional neurological disorder (FND) emphasise biological vulnerability affecting motor, sensory, and integrative processing as a predisposing factor. While early‐life influences were traditionally viewed within psychological frameworks, little is known about early‐life biological vulnerabilities that may predispose to FND or shape its clinical presentation.

**Methods:** Using TriNetX, a large electronic health records dataset, we assessed rates of genetic, congenital, perinatal, and neurodevelopmental diagnoses, selected to capture a broad range of early‐life conditions, including those that may lead to sensory and motor system vulnerability. Comparisons were performed between 188,868 individuals with FND and demographically matched migraine and depression cohorts, and between matched cohorts of 42,015 individuals with motor FND and functional seizures.

**Results:** Compared with migraine and depression, FND was associated with increased odds of heritable connective tissue disorders, heritable neuromuscular disorders, and congenital malformations, including those of the musculoskeletal system and sensory organs (Table 1). Apart from attention‐deficit/hyperactivity disorder, which was more prevalent in the depression cohort, neurodevelopmental disorders were consistently enriched in FND, alongside increased rates of perinatal adversity related to short gestation, low birth weight, and newborn respiratory distress. Motor FND showed stronger associations with connective tissue, neuromuscular, and musculoskeletal conditions, whereas functional seizures were more enriched for chromosomal abnormalities and neurodevelopmental disorders (Figure 1).

**TABLE 1** Genetic, congenital, perinatal, and neurodevelopmental factors in functional neurological disorder (FND) compared to matched cohorts with migraine and depression.

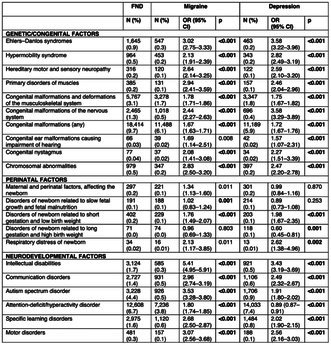

**Figure:**
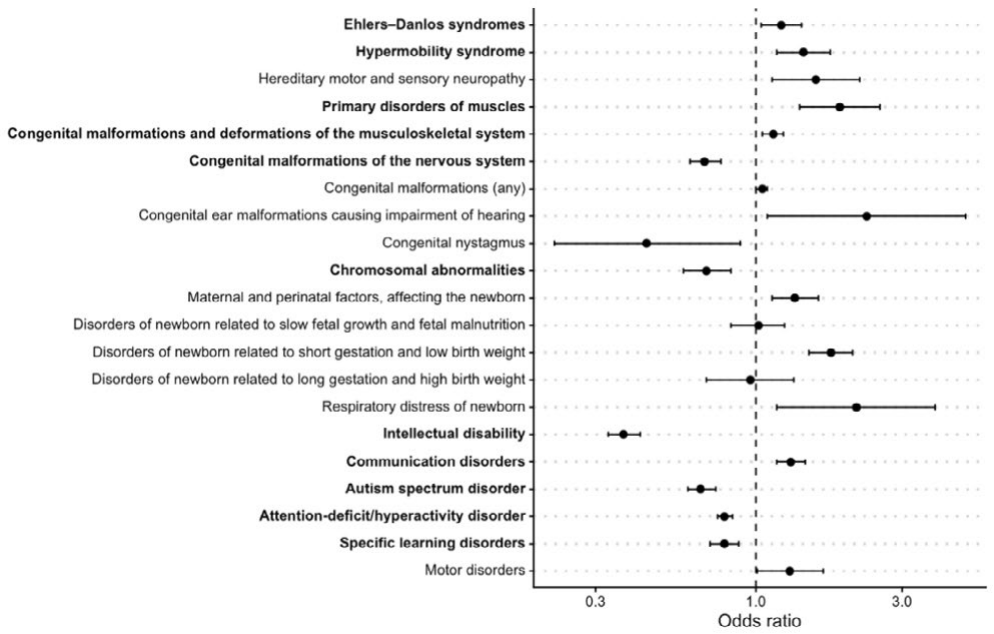
**FIGURE 1** Congenital and developmental factors in functional motor disorder versus functional seizures. OR >1 indicate higher odds in the motor subtype, whereas OR.

**Conclusion:** Genetic and early‐life biological factors may predispose to functional symptoms. Distinct associations were observed for functional motor and seizure presentations. Our findings support a more biologically informed understanding of FND.

**Disclosure:** E.I. has no disclosures. R.B. is supported by the Slovenian Research and Innovation Agency as a member of the research programme Medical Physics (P1‐0389). T.A.P. does medical expert reporting in personal injury and clinical negligence cases, including in cases of FND. He has received consultancy fees from Arialys Therapeutics. T.R.N. does medical expert reporting in personal injury and clinical negligence cases, including in cases of FND. He has received financial support for lectures from the FND Society (FNDS). He receives royalties from CRC Press for The Pocket Prescriber textbook series. He also has received grant funding, including for studies related to FND, from the UK National Institute for Health and Care Research (NIHR) and the Medical Research Council (MRC). He is co‐chair of the FNDS patient liaison committee and on the medical advisory boards of the charities FND Hope UK and FND Action, and a trustee of FND Action. M.J.E. does medical expert reporting in personal injury and clinical negligence cases, has shares in Brain & Mind, which provides neuropsychiatric and neurological rehabilitation in the independent medical sector, has received financial support for lectures from the International Parkinson's and Movement Disorders Society and the FND Society (FNDS), receives royalties from Oxford University Press for his book, The Oxford Specialist Handbook of Parkinson's Disease and Other Movement Disorder, has received honoraria for medical advice to Teva Pharmaceuticals and educational events, receives grant funding from the National Institute for Health and Care Research (NIHR), is the deputy editor of the European Journal of Neurology, and is on the medical advisory boards of the charities FND Hope and the British Association of Performing Arts Medicine.

## OPR‐023

### Risk of epilepsy in people with adult‐onset hydrocephalus: Insights from the UK Biobank

#### J. Buonocore ^1^; F. Fortunato^2^; E. Fratto^1^; I. Sammarra^2^; A. Gambardella^2^; A. Quattrone^1^; A. Quattrone^1^

##### ^*1*^ *Neuroscience Research Center, University “Magna Graecia”, Catanzaro, Italy;* ^*2*^ *Institute of Neurology, Department of Medical and Surgical Sciences, University “Magna Graecia”, Catanzaro, Italy*

**Background and Aims:** Epileptic seizures frequently occur in Alzheimer's disease (AD) and other neurodegenerative dementia; conversely, little is known about epilepsy risk in idiopathic adult‐onset hydrocephalus.

**Methods:** We analyzed data from the UK Biobank cohort. Diagnoses of “hydrocephalus” and “epilepsy” were identified through ICD‐10‐coded health records, excluding congenital and secondary cases. The association between these two diseases was investigated using logistic regression (adjusted for age and sex); subsequently, we performed a nested case‐control study to evaluate the hazard ratio (HR) for incident epilepsy in people with adult‐onset hydrocephalus using Cox proportional hazards models with a dedicated sensitivity analysis including different sets of covariates such as demographic, lifestyle, vascular, and genetic factors.

**Results:** Our cohort included 483,790 controls, 5028 individuals with epilepsy, and 320 with adult‐onset hydrocephalus. Hydrocephalus was strongly associated with epilepsy (OR: 9.6, 95% CI: 6.4–13.8, *p* < 0.001). Adjusted Cox models demonstrated a markedly increased risk of incident epilepsy in people with adult‐onset hydrocephalus, with HR values ranging from 14.62 (95% CI: 7.91–27.00, *p* < 0.001) to 23.80 (95% CI: 12.87–44.03, *p* < 0.001) across models, after adjusting for multiple covariates. Results were also consistent after excluding people with comorbid neurodegenerative dementias such as Alzheimer's disease.

**Conclusion:** We demonstrated a markedly increased risk of epilepsy in adult‐onset hydrocephalus, independent of vascular and neurodegenerative comorbidities and not attributable to shunt‐related complications. These findings underscore the importance of increased diagnostic vigilance, as seizures may be under‐recognized in this population, and support further research to clarify mechanisms and optimize management strategies.

**Disclosure:** Nothing to disclose.

## OPR‐024

### Mental health outcomes among refugees and internally displaced people in Africa: A systematic review

#### O. Uwishema

##### Department of Research and Education, Oli Health Magazine Organization, Kigali, Rwanda

**Background and Aims:** Refugees and internally displaced people in Africa are exposed to conflict, violence, and displacement‐related stressors that substantially increase the risk of mental health disorders. However, a comprehensive continent‐wide synthesis of mental health outcomes in these populations is lacking. This study aimed to systematically review the prevalence, risk factors, and interventions related to mental health among displaced populations in Africa.

**Methods:** This systematic review followed Preferred Reporting Items for Systematic Reviews and Meta‐Analyses 2020 guidelines and was registered with the International Prospective Register of Systematic Reviews. Five databases and grey literature were searched up to January 20, 2025. Eligible studies assessed mental health outcomes among refugees, internally displaced persons, or asylum seekers in African countries. Risk of bias was evaluated using Joanna Briggs Institute tools and the revised Cochrane Risk of Bias tool.

**Results:** Seventy‐eight studies from over 20 African countries were included. Depression, post‐traumatic stress disorder, anxiety, and general psychological distress were the most frequently reported conditions, with prevalence estimates ranging from 6.8 percent to over 80 percent. Common risk factors included exposure to violence, gender‐based violence, prolonged displacement, and socioeconomic hardship. Protective factors included social support, community cohesion, and access to basic services. Intervention studies were limited but suggested potential benefits of group therapy, task‐shifting approaches, and culturally adapted psychosocial interventions.

**Conclusion:** Displaced populations in Africa experience a high burden of mental health disorders, while access to evidence‐based interventions remains limited. Strengthening culturally appropriate mental health services and integrating them into humanitarian and national health systems is urgently needed.

**Disclosure:** Nothing to disclose.

## OPR‐025

### Changing incidence patterns for multiple sclerosis in a large population‐based study in the province of Palermo, southern Italy

#### P. Ragonese ^1^; S. Iacono^2^; G. Schirò^2^; W. Mazzucco^3^; C. Norrito^3^; S. Mazzola^3^; M. Zarcone^3^; G. Sorbello^4^; M. Andolina^4^; G. Callari^2^; S. Realmuto^5^; G. Vazzoler^6^; S. Cottone^6^; L. Baiamonte^4^; P. Aridon^4^; G. Salemi^4^; L. Grimaldi^2^

##### ^*1*^ *Departmental School of Medicine and Health Sciences, Saint Camillus International Medical University, Rome, Italy; Neurology and Multiple Sclerosis Center, Foundation Institute “G. Giglio”, Cefalù, PA, Italy;* ^*2*^ *Neurology and Multiple Sclerosis Center, Foundation Institute “G. Giglio”, Cefalù, PA, Italy;* ^*3*^ *Department of Health promotion, Maternal and Infant care, Internal Medicine, and Medical Specialties (PROMISE), University of Palermo, Italy;* ^*4*^ *Department of Biomedicine, Neurosciences, and Advanced Diagnostics (BIND), University of Palermo, Italy;* ^*5*^ *Neurology Unit, Azienda Ospedaliera Ospedali Riuniti Villa Sofia Cervello, Palermo, Italy;* ^*6*^ *Neurology Unit, ARNAS Civico, Palermo, Italy*

**Background and Aims:** The global epidemiology of multiple sclerosis (MS) is changing over time and the understanding of its epidemiological features in low‐prevalence areas is crucial for tailoring healthcare strategies and optimizing therapeutic interventions (1).

**Methods:** We collected demographic and clinical information of people with MS (pwMS) starting from 01st January 2000 to 31st December 2024 living in the Province of Palermo, southern Italy (pop. 1,268,000 inhabitants). The observational time was divided into five‐year intervals. We calculated crude and sex and age‐adjusted incidence rates in the analysed time frame. MS prevalence was estimated on the prevalence day, December 31st, 2024.

**Results:** A total of 2423 pwMS were diagnosed as of 31st December 2024, giving a prevalence of 201/100.000 person (95% CIs: 193–209). The annual incidence rate increased from 4.1/100.000 person/year to 7.6/100.000 person/year over time (*p* = 0.007; Figure 1). Incidence rate was higher in women, reaching the peak of 9.7/100.000 person/year after 2020 (Figure 2A). While the incidence of early‐onset MS was almost similar across eras (*p* = 0.9), we observed an increasing trend of incidence rate for adult‐onset (*p* = 0.011) and late‐onset MS (*p* = 0.001; Figure 2B).

**Figure:**
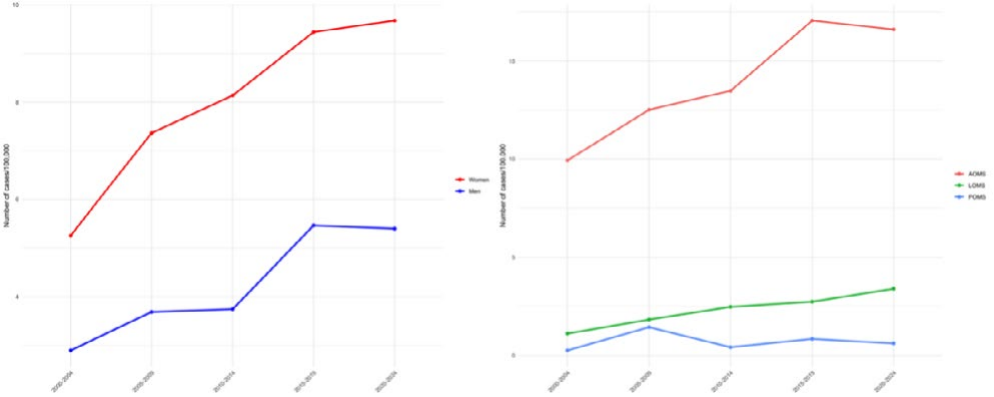
**FIGURE 1** Crude and age and sex adjusted incidence rates for MS in the Province of Palermo

**Conclusion:** Incidence and prevalence of MS in Palermo Province are higher than previously reported, making this province a high‐risk area. Our population‐based study confirms increasing incidence rates of late‐onset MS over time, confirming the impact that cases with late‐onset MS are achievion in the clinical and pharmaco‐economical management of this disease.

**Disclosure:** Nothing to disclose.

## OPR‐026

### Drivers of rising prevalence in major motor neurodegenerative diseases: Temporal trends in Sweden and France (2003–2021)

#### O. Guinebretière^1^; F. Yang^2^; F. Fang^2^; T. Nedelec ^1^

##### ^*1*^ *Sorbonne University, Paris Brain Institute – ICM, CNRS, Inria, Inserm, AP‐HP, Hôpital de la Pitié Salpêtrière, Paris, France;* ^*2*^ *Institute of Environmental Medicine, Karolinska Institutet, Stockholm, Sweden*

**Background and Aims:** The prevalence of Parkinson's disease (PD), multiple sclerosis (MS), and motor neuron diseases (MND) is rising globally. However, it is unclear to what degree this reflects increased incidence versus improved survival after diagnosis.

**Methods:** We performed two nationwide, population‐based cohort studies including all individuals in Sweden (2001–2016) and France (2009–2022). Pooled mixed‐effects models determined temporal trends in prevalence, crude and age‐sex‐standardized incidence, and life expectancy at diagnosis.

**Results:** Prevalence of PD, MS, and MND increased significantly between 2003 and 2021. While crude incidence of PD and MS remained stable (PD: IRR = 0.998; MS: IRR = 0.992), standardized incidence decreased for PD (IRR = 0.986) but not MS (IRR = 0.995). For MND, both crude and standardized incidence increased (IRR = 1.018 and 1.008, respectively). Life expectancy at diagnosis increased for PD between 2003 and 2013 (+0.95 months/year) then decreased 2013–2022 (−1.20 months/year), while increasing throughout for MS (+2.35 months/year) and MND (+0.34 months/year).

**Figure:**
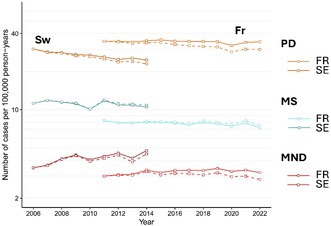
**FIGURE 1** Country‐specific of secular trends in incidence rates of Amyotrophic Lateral Sclerosis, Mulitple Sclerosis and Parkinson's disease assuming a linear relationship with time in France and Sweden (2003–2022).

**Figure:**
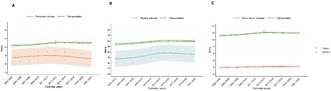
**FIGURE 2** Secular trends in life expectancy at diagnosis of patients with (A) PD, (B) MS, and (C) MND compared with the general population.

**Conclusion:** These findings reveal fundamentally different prevalence drivers with critical implications for disease etiology and healthcare planning. MS prevalence is survival‐driven through therapeutic advances, with no evidence of changing population‐level risk factors. MND shows true incidence increases, suggesting emerging or intensifying environmental or lifestyle risk factors. PD demonstrates declining age‐adjusted incidence, with prevalence growth historically driven by improved survival. These distinct mechanisms demand disease‐specific strategies.

**Figure:**
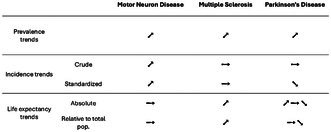
**FIGURE 3** Varying dynamics explaining increases in prevalence across diseases.

**Disclosure:** Nothing to disclose.

## Neurogenetics

## OPR‐027

### Global SPG11 genotype‐phenotype landscape: Integrated analysis of 560 patients

#### A. Daponte ^1^; M. Lima^2^; G. Koutsis^1^; G. Karadima^1^; C. Kartanou^1^; A. Gabas^3^; R. Lee^3^; K. Gunanayagam^3^; R. Kaiyrzhanov^3^; A. Cortese^3^; N. W. Wood^3^; E. Kara^3^; H. Houlden^3^; R. Maroofian^3^

##### ^*1*^ *1st Department of Neurology, Eginitio Hospital, Medical School, National and Kapodistrian University of Athens, Athens, Greece;* ^*2*^ *Second Department of Neurology, Aristotle University of Thessaloniki, Thessaloniki, Greece;* ^*3*^ *Department of Neuromuscular Diseases, UCL Queen Square Institute of Neurology, London, UK*

**Background and Aims:** SPG11 is the most frequent cause of autosomal recessive hereditary spastic paraplegia (HSP). However, its clinical spectrum and genotype‐phenotype correlations remain incompletely defined.

**Methods:** We assembled a multicenter international cohort of 75 genetically confirmed SPG11 patients with detailed epidemiological and clinical data and performed a literature review of published SPG11 cases. We analyzed clinical, radiological and genetic features to investigate genotype‐phenotype associations.

**Results:** The merged dataset comprised 560 SPG11 patients (75 in‐house; 485 literature) with >200 unique variants, without mutational hotspot. Mean age at onset was ~16 years. In addition to spastic paraparesis, the most frequent feature was cognitive impairment/intellectual disability (91%), with the vast majority presenting with complicated HSP (96%), while only 4% exhibited a pure HSP phenotype. Distinct SPG11‐associated phenotypic subtypes overlapping with other neurodegenerative diseases, included a predominately ataxic phenotype (30%), a parkinsonism/dystonia subtype (10%) and an amyotrophic lateral sclerosis (ALS)‐like phenotype (18%). Additional manifestations included dysarthria (43%), neuropathy (17%), and epilepsy, optic atrophy and psychiatric symptoms (~5% each). Brain MRI was abnormal in 95%, most commonly showing thin corpus callosum (89%), white‐matter hyperintensities (63%) and cortical atrophy (31%). Ten recurrent variants were identified; some with specific clinical patterns. The most frequent worldwide, c.733_734del (*n* = 50), was associated with increased cerebellar/extrapyramidal manifestations; whereas, the recurrent m issense variant c.5381T>C was linked to strikingly later‐onset (mean 52 years) and milder phenotype.

**TABLE 1** Epidemiological, clinical and radiological characteristics of the international SPG11 cohort compared with the combined dataset (75 in‐house patients and 485 cases extracted from the literature). Values are presented as percentages.

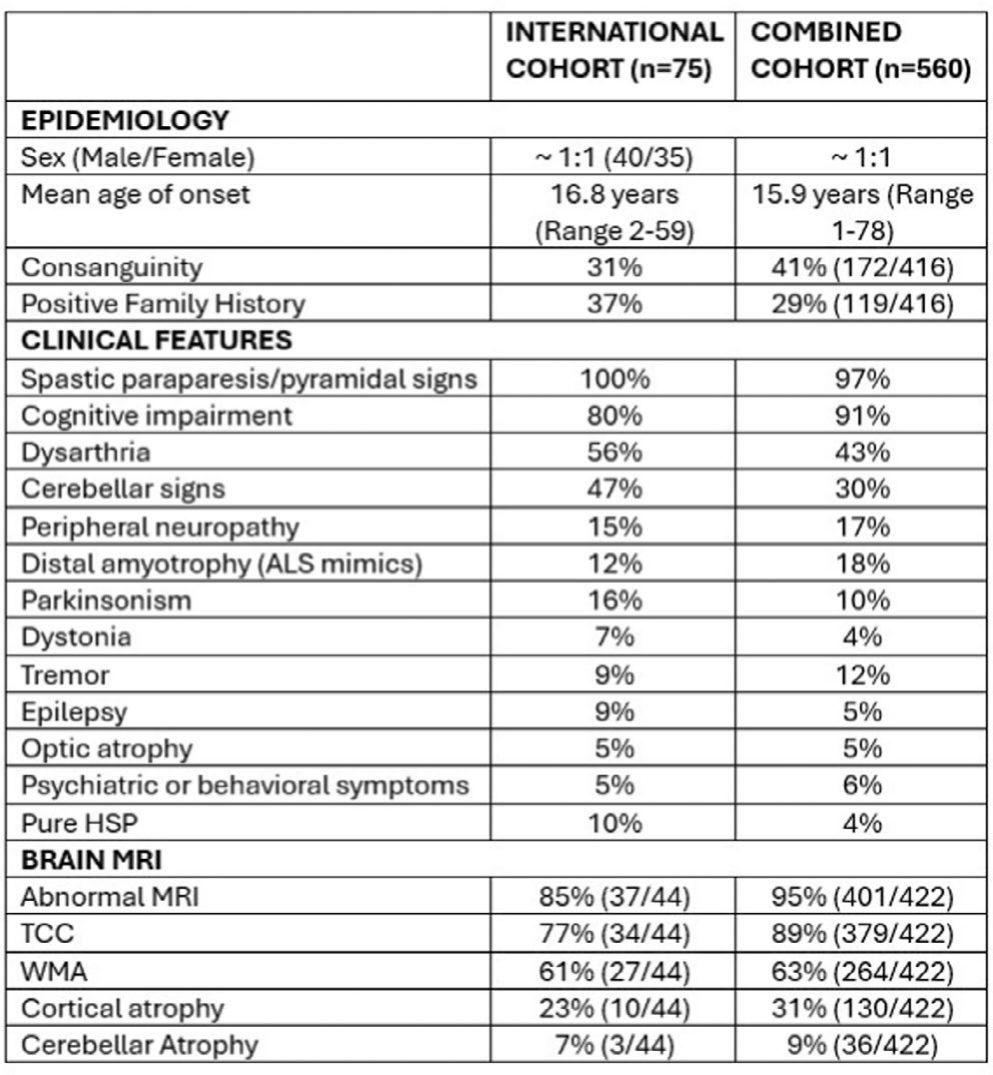

**Figure:**
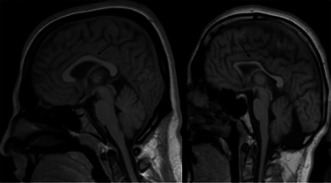
**FIGURE 1** Sagittal T1‐weighted brain MRI from two SPG11 patients showing marked thinning of the corpus callosum (TCC), as representative examples of the most common radiological finding in SPG11 (arrows).

**Conclusion:** This integrated analysis provides the largest SPG11 genotype‐phenotype dataset to date, refining the SPG11 landscape. Despite extensive allelic diversity, recurrent variants are associated with distinct clinical features, potentially facilitating diagnosis and prognosis in SPG11‐HSP.

**Disclosure:** Nothing to disclose.

## OPR‐028

### Genome sequencing as a diagnostic tool for common hereditary spinocerebellar ataxias with repeat expansions

#### E. Perraki; M. Bieg; A. Kaune; K. Kandaswamy; K. Bruesehafer; J. Pinto‐Basto; P. Bauer

##### Centogene, Rostock, Germany

**Background and Aims:** Spinocerebellar ataxias (SCAs) is a group of autosomal dominant neurodegenerative disorders. Several common SCAs are caused by CAG repeat expansions, a type of variant that is a challenge for short read next‐generation sequencing. We used genome sequencing (GS) with the application of ExpansionHunter (EH) for CAG repeat detection in SCA1, 2, 3, 6, 7, and 17.

**Methods:** The study cohort comprised 301 adult ataxia patients. PCR‐free GS was performed on an Illumina platform. CAG repeat loci associated with SCA1, 2, 3, 6, 7, and 17 were sized using EH. Expanded CAG alleles of all loci were reviewed, and orthogonal confirmation was performed using PCR‐based fragment length analyses.

**Results:** EH detected expanded CAG alleles in all SCA loci (Table 1). The presence of all expanded alleles was confirmed orthogonally. 4/5 SCA1 calls had repeat size deviation of 2‐18 repeats between methods and one expanded allele was reclassified to “normal expanded” due to repeat interruptions. 4/8 SCA2 and 3/4 SCA6 expanded calls resulted in the same repeat size between methods, while 4/8 SCA2 and 1/4 SCA6 calls deviated by 1–2 CAGs. SCA3, SCA7 and SCA17 calls had repeat size deviation of 7–15, 1–27 and 1–4 repeats between methods, respectively, with the PCR‐based method showing higher repeat size.

**TABLE 1** The GS results via EH for the different SCA loci and their orthogonal confirmation results. One of the SCA1 calls was reclassified to normal due to the presence of interruptions in the CAG repeats (*).

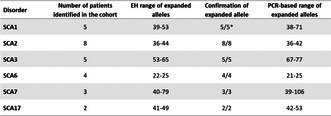

**Conclusion:** Our data show that EH application for SCA repeat expansion detection can increase the diagnostic utility of GS. Orthogonal confirmation remains relevant for positive EH calls: whilst shorter range CAG expansions show the same or ±1 CAG size between GS and conventional fragment length analysis, larger expanded alleles require orthogonal method for accurate repeat sizing.

**Disclosure:** All authors are employees of CENTOGENE GmbH.

## OPR‐029

### Parkinsonian manifestations in primary mitochondrial diseases: Evidence from a large Italian multicenter cohort

#### I. Arena ^1^; C. La Morgia^2^; C. Lamperti^3^; G. Primiano^4^; P. Lopriore^5^; G. Urbano^6^; F. Magri^7^; D. Ronchi^7^; M. Percetti^7^; A. Di Fonzo^7^; G. Cecchi^5^; F. Santorelli^8^; A. Ardissone^3^; T. Mongini^6^; S. Servidei^4^; V. Carelli^2^; M. Mancuso^5^; O. Musumeci^9^

##### ^*1*^ *BIOMORF Department, University of Messina;* ^*2*^ *IRCCS Istituto delle Scienze Neurologiche di Bologna;* ^*3*^ *UO Medical Genetics and Neurogenetics, Fondazione IRCCS Istituto Neurologico “Carlo Besta”, Milan, Italy;* ^*4*^ *Fondazione Policlinico Universitario A. Gemelli, IRCCS, Rome, Italy;* ^*5*^ *Department of Clinical and Experimental Medicine, Neurological Clinic, University of Pisa;* ^*6*^ *Department of Neurosciences, University of Torino;* ^*7*^ *Dino Ferrari Center, Department of Pathophysiology and Transplantation, University of Milan;* ^*8*^ *Molecular Medicine for Neurodegenerative and Neuromuscular Diseases Unit, IRCCS Stella Maris Foundation;* ^*9*^ *Unit of Neurology and Neuromuscular Disorders, Department of Clinicaldi and Experimental Medicine, University of Messina, Messina, Italy*

**Background and Aims:** Parkinsonism represents a common hypokinetic manifestation in individuals with primary mitochondrial diseases (PMDs) and has been reported in association with both mitochondrial and nuclear DNA variants. Despite this, its phenotypic features, associated comorbidities, and genetic landscape remain insufficiently defined. This study aimed to characterize the clinical and genetic profile of parkinsonism in a large, multicenter PMD cohort through a detailed phenotyping approach.

**Methods:** Patients were identified from the Italian Mitochondrial Disease Registry (Genomit). Available clinical records were reviewed to collect demographic, genetic, imaging, and comorbidity data.

**Results:** ixty‐six patients with genetically confirmed PMDs were included, of whom 45 carried nuclear DNA variants and 21 mitochondrial DNA variants. The most frequently implicated genes were POLG (*n* = 15), TWNK (*n* = 10), OPA1 (*n* = 9). Parkinsonism onset occurred at a median age of 62.2 years, notably later than the median age at PMD onset (40.0 years), although in approximately 20% of cases both conditions manifested within 1 year. The most prevalent clinical syndrome was CPEO plus with several other mitochondrial syndromes observed less frequently. DAT‐SCAN imaging mostly demonstrated bilateral dopaminergic deficits in over half of cases, along with additional abnormalities such as substantia nigra alterations and asymmetric dopamine uptake.

**Conclusion:** In PMDs, parkinsonism typically emerges later than the underlying mitochondrial disorder. These findings expand the clinical characterization of parkinsonism in PMDs and support the need for larger, longitudinal studies to better delineate genotype–phenotype relationships and disease evolution.

**Disclosure:** Nothing to disclose.

## OPR‐030

### Clinical and genotypic spectrum of twinkle‐related disorders: Insights from a multinational cohort study

#### P. Lopriore ^1^; Z. Ünlütürk^2^; T. Klopstock^3^; A. Karaa^4^; C. Rouzier^5^; C. Domínguez González^6^; C. Lamperti^7^; M. Mancuso^1^; TReDIC^8^

##### ^*1*^ *Department of Clinical and Experimental Medicine, Neurological Institute, University of Pisa;* ^*2*^ *Neurology Clinic, University of Health Sciences Kocaeli Derince Training Hospital, Türkiye;* ^*3*^ *Friedrich‐Baur‐Institute at the Department of Neurology, LMU University Hospital, Ludwig‐Maximilians‐Universität (LMU) München, Germany;* ^*4*^ *Massachusetts General Hospital Genetics Division, Harvard Medical School, Boston, USA;* ^*5*^ *Service de génétique médicale, Centre de Référence des Maladies Mitochondriales, CHU Nice, Université Côte d'Azur, CNRS, INSERM, IRCAN, Nice, France;* ^*6*^ *Servicio de Neurología – Unidad de Neuromuscular – ERN‐NMD CIBERER, Instituto de Investigación imas12. Hospital Universitario 12 de Octubre, Madrid, Spain;* ^*7*^ *Fondazione IRCCS Istituto Neurologico Carlo Besta, Medical Genetics and Neurogenetics Unit, Milan, Italy;* ^*8*^ *Twinkle‐Related Disorders International Consortium for Trial Readiness (TReDIC)*

**Background and Aims:** Twinkle, encoded by the TWNK gene, is a mitochondrial DNA helicase that unwinds DNA during replication, crucial for mitochondrial function. Twinkle‐related disorders encompass diverse genetic conditions with mitochondrial dysfunction. Despite several described phenotypes, the full clinical and molecular spectrum remains unclear. This study characterizes phenotypic and genotypic variability in multinational patients with Twinkle‐related disorders.

**Methods:** A retrospective cohort study included patients from specialized centers in Italy, France, Germany, Spain, Denmark, Hungary, and the United States, establishing the Twinkle‐Related Disorders International Consortium for Trial Readiness (TReDIC). Data from medical records included clinical features, age at onset, disease progression, and genetic testing results. Phenotypes were classified as infantile‐onset cerebellar ataxia, parkinsonism, primary mitochondrial myopathy, multisystem involvement, asymptomatic carriers, undetermined, or other. All diagnoses were genetically confirmed, with variants documented. Outcomes included phenotype prevalence, symptom chronology, and mutational patterns.

**Results:** The study analyzed 189 patients (116 females), mean onset 40.3 years; 70.4% were alive at analysis. Primary mitochondrial myopathy was predominant (85.2%). Common features included progressive external ophthalmoplegia (84.7%), skeletal myopathy (55.6%), hearing loss (17.5%), and psychiatric symptoms (15.3%). At onset, 76.8% had neuromuscular symptoms, 19.6% CNS involvement, and 3.6% multi‐organ; after >8 years, proportions shifted to 54.4%, 23.3%, and 23.3%. Seventy‐three TWNK variants (16 novel), mostly missense, clustered in critical functional regions.

**Figure:**
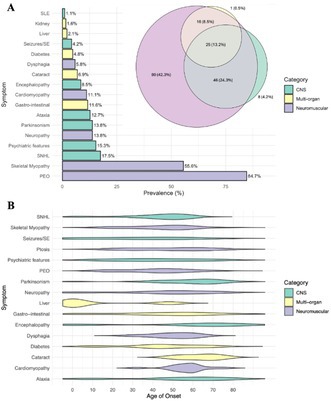
**FIGURE 1** Clinical features and years into disease of each individual symptom in patients with Twinkle‐related disorder.

**Figure:**
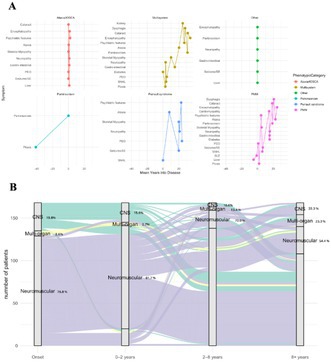
**FIGURE 2** Disease progression in patients with Twinkle‐related disorder.

**Figure:**
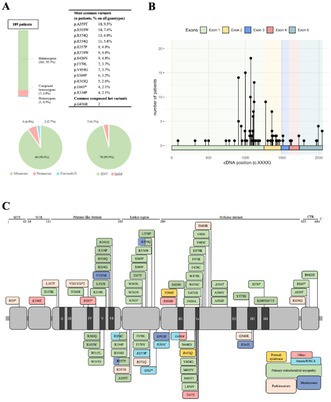
**FIGURE 3** Variants across TWNK gene and protein domains.

**Conclusion:** This multinational cohort clarifies the broad phenotypic spectrum, progression patterns, and clinically relevant mutational hotspots in Twinkle‐related disorders. International collaborations like TReDIC are essential to improve understanding and support future clinical trial design in this rare disease context.

**Disclosure:** Nothing to disclose.

## OPR‐031

### CANVAS‐associated AAGGG repeat expansions cause cell‐specific reduction in RFC1 expression and impaired DNA damage response

#### R. Curro ^1^; N. Dominik^1^; S. Facchini^1^; R. Parolin Schnekenberg^1^; C. Perini^2^; R. Ronco^3^; B. Rugginini^4^; A. Ghia^4^; S. Lowe^5^; A. Hicks^6^; E. Vegezzi^7^; E. Abati^8^; R. Velasco^9^; M. Sereno^10^; G. Gutiérrez‐Gutiérrez^11^; S. Thomas^12^; P. Alberti^13^; Z. Chen^14^; M. Grant Peters^15^; J. Brenton^15^; Y. Lim^16^; Z. Jaunmuktane^17^; S. Brandner^6^; D. Bennett^18^; A. Hoke^19^; M. Reilly^1^; H. Houlden^1^; M. Ryten^20^; G. Cavaletti^21^; A. Argyriou^22^; V. Pirota^23^; J. Bruna^9^; C. Briani^24^; E. Crespan^2^; J. Jepson^5^; A. Cortese^1^

##### ^*1*^ *Department of Neuromuscular Diseases, UCL Queen Square Institute of Neurology, London, UK;* ^*2*^ *Institute of Molecular Genetics IGM‐CNR “Luigi Luca Cavalli‐Sforza”, Pavia, Italy;* ^*3*^ *Neurology Department, NRSC, Fondazione IRCCS Istituto Neurologico Carlo Besta, Milan, Italy;* ^*4*^ *Department of Brain and Behavioral Sciences, University of Pavia, Pavia, Italy;* ^*5*^ *Department of Epilepsy, UCL Queen Square Institute of Neurology, London, UK;* ^*6*^ *Department of Neurodegenerative Disease, UCL Queen Square Institute of Neurology, London, UK;* ^*7*^ *IRCCS Mondino Foundation, Pavia, Italy;* ^*8*^ *Department of Pathophysiology and Transplantation (DEPT), University of Milan, Milan, Italy;* ^*9*^ *Neuro‐Oncology Unit, Department of Neurology, Hospital Universitari de Bellvitge, Barcelona, Spain;* ^*10*^ *Department of Medical Oncology, Hospital Universitario Infanta Sofía, Alcobendas, Madrid, Spain;* ^*11*^ *Department of Neurology, Hospital Universitario Infanta Sofía, Universidad Europea de Madrid, Madrid, Spain;* ^*12*^ *Department of Neurology, Johns Hopkins University School of Medicine, Baltimore, USA;* ^*13*^ *Experimental Neurology Unit, School of Medicine and Surgery, University of Milano‐Bicocca, Monza, Italy;* ^*14*^ *Centre for Preventive Neurology, Wolfson Institute of Population Health, Queen Mary University of London, Charterhouse Square, London, UK;* ^*15*^ *UK Dementia Research Institute, University of Cambridge, Cambridge, UK;* ^*16*^ *Division of Neuropathology, UCL Queen Square Institute of Neurology, London, UK;* ^*17*^ *Queen Square Brain Bank for Neurological Disorders, UCL Queen Square Institute of Neurology, London, UK;* ^*18*^ *Nuffield Department of Clinical Neuroscience, University of Oxford, John Radcliffe Hospital, Oxford, UK;* ^*19*^ *Department of Neuroscience, Johns Hopkins University School of Medicine, Baltimore, USA;* ^*20*^ *Department of Clinical Neurosciences, School of Clinical Medicine, University of Cambridge, Cambridge, UK;* ^*21*^ *Fondazione IRCCS San Gerardo dei Tintori di Monza, Monza, Italy;* ^*22*^ *Department of Neurology, Agios Andreas State General Hospital of Patras, Patras, Greece;* ^*23*^ *Department of Chemistry, University of Pavia, Pavia, Italy;* ^*24*^ *Department of Neurosciences, University of Padova, Padova, Italy*

**Background and Aims:** Cerebellar ataxia, neuropathy, vestibular areflexia syndrome (CANVAS), caused by biallelic AAGGG expansions in RFC1, is increasingly recognised as one of the most common causes of ataxia and sensory neuropathy. Despite previous studies failed to detect a decrease in RFC1 transcript and protein expression, the recessive mode of inheritance and the identification of patients carrying compound heterozygous null variants suggest a loss‐of‐function mechanism underlying this disease.

**Methods:** We assessed the effects of AAGGG expansions on RFC1 expression and function, leveraging reporter assays, brain tissue, patient‐derived cell lines, and a Drosophila model. Moreover, we investigated the association between RFC1 expansions and chemotherapy‐induced neuropathy in a cohort of cancer patients who underwent oxaliplatin treatment.

**Results:** AAGGG expansions cause a length‐dependent impairment of transcription efficiency in reporter assays and decrease RFC1 expression in human cerebellum and iPSC‐neurons. Patient‐derived cell lines showed an increased apoptotic response after exposure to platinum compounds. CRISPR/Cas9‐mediated excision of the AAGGG repeat restored RFC1 expression and the DNA damage response. Furthermore, neuronal‐specific knockdown of RFC1 in flies led to reduced survival, motor impairment, and increased DNA damage. Finally, individuals carrying RFC1 expansions and exposed to oxaliplatin treatment presented a higher risk to develop a clinically significant neuropathy compared to non‐carriers.

**Conclusion:** Our data suggest that AAGGG expansions cause a cell‐ and tissue‐specific decrease in RFC1 expression with consequent impairment of DNA damage response. The rescue of RFC1 expression and DNA repair after repeat‐excision lays the groundwork for future therapeutic avenues.

**Disclosure:** Nothing to disclose.

## Neurorehabilitation

## OPR‐032

### Auditory cueing modulates motor performance and brain activity in people with Parkinson's disease: A kinematic and fMRI study

#### A. Gardoni ^1^; E. Sarasso^2^; M. Forghieri^1^; A. Grassi^1^; R. Balestrino^3^; S. Basaia^1^; C. Tripodi^1^; E. Canu^4^; M. Volontè^5^; D. Corbetta^6^; M. Filippi^7^; F. Agosta^4^

##### ^*1*^ *Neuroimaging Research Unit, Division of Neuroscience, IRCCS San Raffaele Scientific Institute, Milan, Italy; and Neurotech Hub, Vita‐Salute San Raffaele University, Milan, Italy;* ^*2*^ *Neuroimaging Research Unit, Division of Neuroscience, IRCCS San Raffaele Scientific Institute, Milan, Italy; and Neurotech Hub, Vita‐Salute San Raffaele University, Milan, Italy; and DINOGMI, University of Genoa, Genoa, Italy;* ^*3*^ *Neurology Unit, and Neurorehabilitation Unit, IRCCS San Raffaele Scientific Institute, Milan, Italy;* ^*4*^ *Neuroimaging Research Unit, Division of Neuroscience, and Neurology Unit, IRCCS San Raffaele Scientific Institute, Milan, Italy; and Neurotech Hub, Vita‐Salute San Raffaele University, Milan, Italy;* ^*5*^ *Neurology Unit, IRCCS San Raffaele Scientific Institute, Milan, Italy;* ^*6*^ *Department of Rehabilitation and Functional Recovery, IRCCS San Raffaele Scientific Institute, Milan, Italy;* ^*7*^ *Neuroimaging Research Unit, Division of Neuroscience, Neurology Unit, Neurorehabilitation Unit, and Neurophysiology Service, IRCCS San Raffaele Scientific Institute, Milan, Italy; and Neurotech Hub, Vita‐Salute San Raffaele University, Milan, Italy*

**Background and Aims:** Cues are specific references that support motor performance by improving speed, timing, and amplitude while reducing variability. This study aimed to investigate the effects of external auditory cueing on bradykinesia‐related kinematic deficits and their neural correlates during a hand‐tapping task in people with Parkinson's disease (pwPD) and healthy controls (HC).

**Methods:** Twenty‐six pwPD and 20 HC performed a right hand‐tapping fMRI task either in a self‐paced condition (NOCue) or following an auditory cue (Cue). Hand‐tapping kinematics were objectively quantified using an optical‐fiber glove.

**Results:** PwPD showed reduced hand‐tapping amplitude and a greater sequence effect on amplitude than HC during NOCue condition. Cueing attenuated the sequence effect on amplitude in pwPD. During the NOCue condition, pwPD showed reduced activity of pallidum relative to HC. No group differences emerged during Cue condition. During Cue relative to NOCue condition, pwPD showed higher activity of pallidum, parietal, temporal, sensorimotor, insular, and cerebellar regions, alongside reduced activation in of paracentral gyrus and cerebellum. Higher activity of pallidum and lower activity of paracentral gyrus correlated with better motor performance and higher positive effects of cueing.

**Conclusion:** Our results demonstrated that auditory cueing reduces bradykinesia in pwPD by facilitating basal ganglia activity and reducing the need of movement programming.

**Disclosure:** Supported by Italian Ministry of Health grant number GR‐2018‐12366005. Andrea Gardoni, Marco Forghieri, Andrea Grassi, Chiara Tripodi, and Maria Antonietta Volontè declare no financial competing interests. Elisabetta Sarasso, Roberta Balestrino, Silvia Basaia, Elisa Canu, and Davide Corbetta are recipient of a grant from the Italian Ministry of Health. Massimo Filippi is Editor‐in‐Chief of the Journal of Neurology, Associate Editor of Human Brain Mapping, Neurological Sciences, and Radiology; received compensation for consulting services from Almirall, Biogen, Bristol‐Myers Squibb, Eli Lilly, Merck, Novartis, Roche, Sanofi; speaking activities from Amgen, Bayer, Biogen, Bristol‐Myers Squibb, Celgene, Chiesi Italia SpA, Eisai, Eli Lilly, Fujirebio, Genzyme, Janssen, Merck, Neopharmed Gentili, Neuraxpharm, Novartis, Novo Nordisk, Roche, Sanofi, Takeda; participation in Advisory Boards for Alexion, Biogen, Bristol‐Myers Squibb, Eli Lilly, GE Healthcare Ltd, Merck, Neuraxpharm, Novartis, Roche, Sandoz, Sanofi, Takeda; scientific direction of educational events for Biogen, Merck, Roche, Celgene, Bristol‐Myers Squibb, Lilly, Novartis, Sanofi‐Genzyme; he receives research support from Biogen Idec, Merck‐Serono, Novartis, Roche, the Italian Ministry of Health, the Italian Ministry of University and Research, and Fondazione Italiana Sclerosi Multipla. Federica Agosta is Associate Editor of NeuroImage: Clinical, has received speaker honoraria from Biogen Idec, Italfarmaco, Roche, Zambon, Eli Lilly, GE Healthcare and Bristol Myers Squibb, and receives or has received research supports from the Italian Ministry of Health, the Italian Ministry of University and Research, AriSLA (Fondazione Italiana di Ricerca per la SLA), the European Research Council, the EU Joint Programme—Neurodegenerative Disease Research (JPND) and Foundation Research on Alzheimer Disease (France).

## OPR‐033

### Mapping attentional deficits: From virtual reality oculomotor biomarkers to atlas‐based structural disconnectivity

#### D. Zeugin ^1^; L. Catinari^2^; L. Defferrard^1^; Y. Perret^3^; D. Perez‐Marcos^3^; A. Serino^1^; S. Crottaz‐Herbette^1^

##### ^*1*^ *Service Universitaire de Neuroréhabilitation (SUN), Lausanne University Hospital, Institution of Lavigny and University of Lausanne, Switzerland;* ^*2*^ *Centre for Studies and Research in Cognitive Neuroscience, University of Bologna, Cesena, Italy. Department of Psychology, University of Bologna, Bologna, Italy;* ^*3*^ *NeuroX group SA (MindMaze), Lausanne, Switzerland*

**Background and Aims:** Standard neuropsychological assessments of post‐stroke attentional deficits often suffer from limited ecological validity and insufficient sensitivity to complex behavioral patterns. Immersive virtual reality (iVR) combined with eye tracking (ET) offers a high‐dimensional characterization of attentional functioning; however, the structural substrates underlying these digital oculomotor biomarkers remain insufficiently understood. This study uses lesion‐based connectomics to link ET‐derived metrics with focal brain damage.

**Figure:**
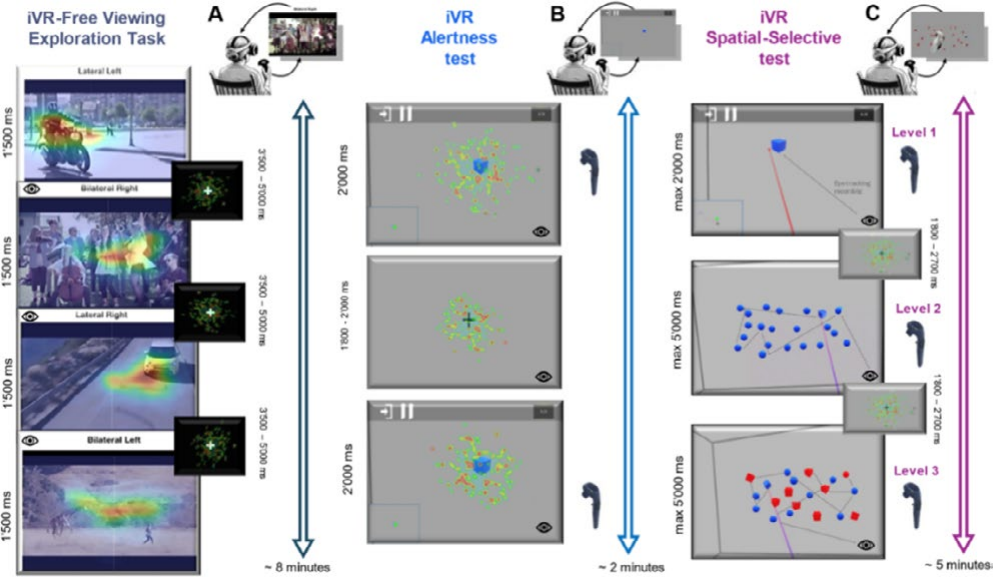
**FIGURE 1** iVR tasks. (A) Free viewing: 4 conditions (lateral/bilateral × left/right) with fixation cross. (B) Alertness: target detection. (C) Spatial selective attention: target localization and selection.

**Methods:** Fifty patients with acquired brain injury underwent iVR‐based assessments of spatial attention. Random forest classifiers were trained to identify attentional deficits from ET metrics. Structural lesion impact was quantified using the Lesion Quantification Toolkit (LQT), estimating parcelwise gray matter lesion load (Schaefer–Yeo atlas) and white matter disconnection severity (HCP‐1065 tractography atlas). Principal component analysis was applied to derive latent oculomotor dimensions. To explore prognostic relevance, longitudinal changes in oculomotor behavior were related to baseline structural disconnectivity.

**Results:** Classification models achieved high diagnostic performance (F1 = 0.88). Lesion quantification revealed that the principal oculomotor component characterizing spatial neglect—reflecting increased gaze bias and reduced visual exploration—was associated with cumulative damage within dorsal and ventral attention networks, notably involving the right frontal aslant tract and superior longitudinal fasciculi. Ongoing longitudinal analyses aim to determine whether these structural‐functional associations predict recovery trajectories.

**Figure:**
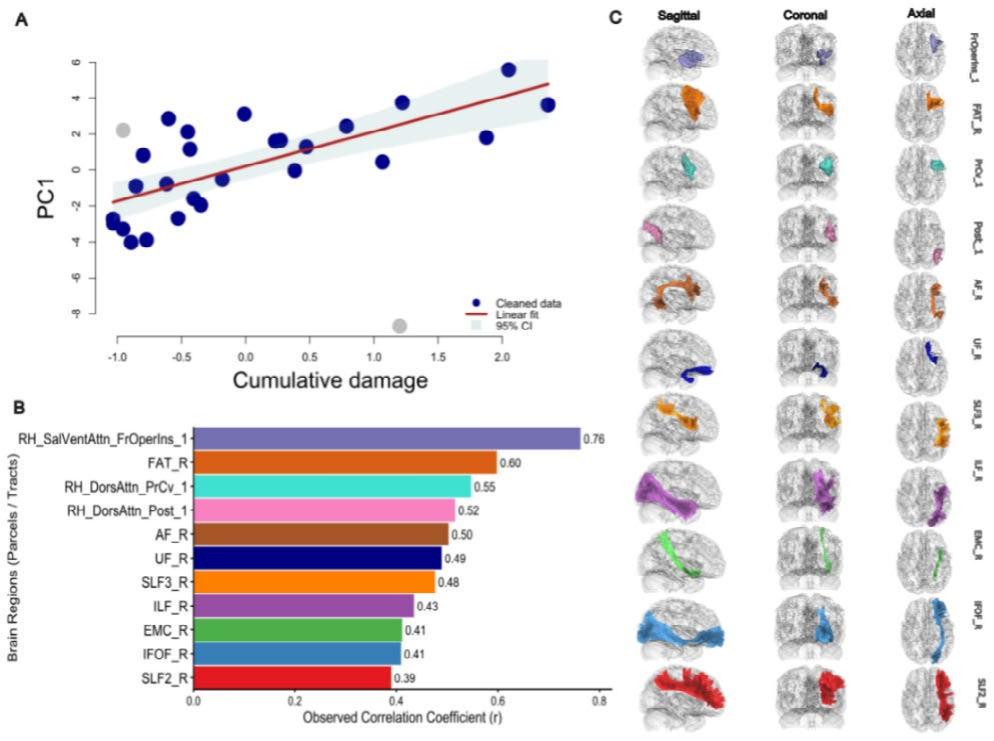
**FIGURE 2** Structural correlates of the oculomotor latent factor (PC1). (A) Linear association between cumulative structural damage across the Sal‐VAN/DAN and the PC1. (B) Region‐wise permutation‐based Spearman correlations. (C) Brain renderings

**Conclusion:** By integrating iVR‐based eye tracking with lesion‐informed connectomics, this study provides a novel framework for characterizing attentional deficits and their neural substrates. The findings suggest that network‐level structural integrity plays a key role in shaping oculomotor manifestations of spatial attention deficits and may inform future digital diagnostic and prognostic tools.

**Disclosure:** Nothing to disclose.

## OPR‐034

### Toward personalized neurorehabilitation: A deep reinforcement learning framework for predicting attentional deficits via in‐silico synthetic lesions

#### L. Defferrard ^1^; D. Zeugin^1^; J. Signer^2^; A. Azar‐Pey^3^; S. Crottaz‐Herbette^1^

##### ^*1*^ *Service Universitaire de Neuroréhabilitation (SUN), Lausanne University Hospital, Institution of Lavigny, Switzerland;* ^*2*^ *University of Lausanne, Switzerland;* ^*3*^ *EPFL, Swiss Federal Institute of Technology in Lausanne, Switzerland*

**Background and Aims:** Attentional deficits, notably hemispatial neglect, are highly disabling consequences of acquired brain injury (ABI) that significantly impair recovery. Current rehabilitation lacks a granular understanding of the underlying behavioral dynamics. This study develops a predictive Deep Neural Network (DNN) framework to model attentional‐motor behavior and assess deficits via synthetic lesions, aiming to provide a foundational tool for automated diagnosis and personalized neurorehabilitation (See Figure 1).

**Figure:**
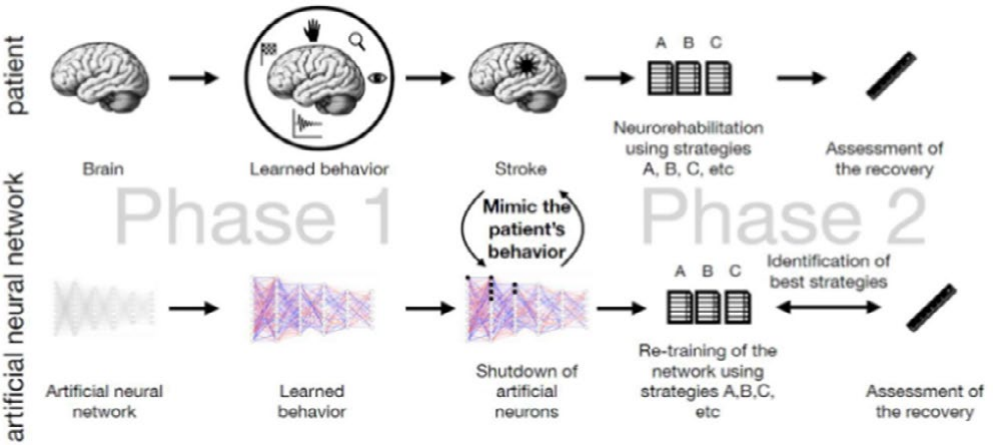
**FIGURE 1** Schematic representation of the different phases of the project.

**Methods:** We integrated a Convolutional Neural Network (CNN) for foveated visual perception with a Deep Reinforcement Learning (DRL) agent using Proximal Policy Optimization (PPO) to resolve a visuospatial selective attention task (Figure 2). Lesion models were generated by selectively deactivating neurons across neural networks layers. A Multilayer Perceptron (MLP) was trained on 17 behavioral metrics (e.g., mean step size, velocity variance) to predict lesion location and magnitude. Behavioral data were validated against a cohort of 43 ABI patients and 19 healthy controls.

**Figure:**
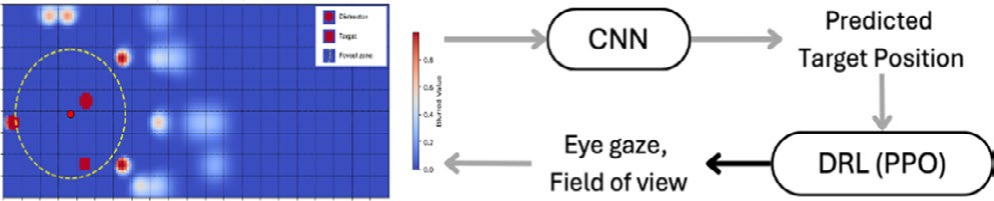
**FIGURE 2** Overview of the deep neural network framework designed to mimic healthy human behavior during a visuospatial selective attention task.

**Results:** The DRL agent successfully mimicked healthy human exploration patterns and trajectories. The MLP demonstrated discriminative performance in identifying in‐silico lesion patterns. While group‐level behavioral metrics showed convergence between in‐silico and in‐vivo data, direct transfer of the MLP's predictive performance to patient data remains limited and need further improvements.

**Conclusion:** This project establishes a robust methodology for computational cognitive modeling. It demonstrates that synthetic lesions in DNNs can reflect specific behavioral impairments, offering a promising path toward data‐driven diagnostic tools and the optimization of individualized rehabilitation strategies in clinical neurology.

**Disclosure:** Nothing to disclose.

## OPR‐035

### Investigating the relationship of trunk and postural control with pulmonary functions in subacute stroke patients

#### N. Duruturk ^1^; S. Ozkan^2^

##### ^*1*^ *Baskent University, Physical Therapy and Rehabilitation Department, Ankara, Türkiye;* ^*2*^ *Physiocare Rehabilitation Center, Ankara, Türkiye*

**Background and Aims:** Stroke is a disease with high mortality and morbidity that not only causes weakness in the extremities, loss of balance and disturbances in trunk and postural control, but also affects respiratory function. The aim of this study was to investigate the relationship between trunk and postural control and pulmonary function in stroke patients.

**Methods:** 32 volunteer patients who were diagnosed with hemiplegia and who met the inclusion criteria participated in the study. Functional independence of the participants was evaluated using Modified Rankin Scale (mRS) and their cognitive function was assessed with the Standardized Mini Mental State Examination. Respiratory function was evaluated with spirometric measurements, inspiratory muscle strength was evaluated with intraoral pressure measurements, trunk control was evaluated using the Trunk Impairment Scale (TIS), postural control was evaluated using the Postural Assessment Scale for Stroke Patients (PASS‐T), computerized postural sway evaluation, and static posture analysis.

**Results:** A significant correlation was found between the TIS scores and inspiratory muscle strength (*p* < 0.05). A significant correlation was also found between the PASS‐T scores and inspiratory muscle strength and pulmonary function (*p* < 0.05). All of the center of pressure parameters measured were significantly correlated with the PEF(L/s) and FEF25–75 (L/s) (*p* < 0.05).

**TABLE 1** Demographic characteristics of the participants.

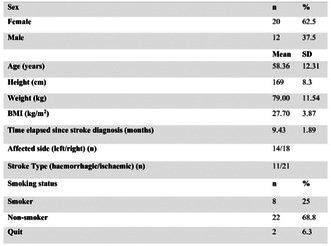

**TABLE 2** Relationship between functional independence and trunk‐postural control and inspiratory muscle strength and respiratory functions.

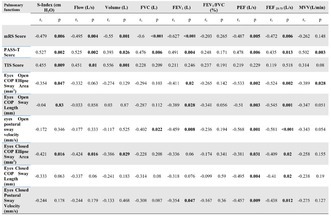

**Conclusion:** In conclusion, this study showed that trunk and postural control are associated with inspiratory muscle strength and pulmonary function. It is recommended that evaluation of trunk and postural control and respiratory functions, as well as exercise training to improve these parameters, should be included in rehabilitation programs for individuals with stroke.

**Disclosure:** Nothing to disclose.

## OPR‐036

### Aerobic training and potential neurogenesis in progressive multiple sclerosis: Focus on the hippocampus and the subventricular zone

#### T. Morozumi ^1^; P. Valsasina^2^; P. Preziosa^3^; A. Meani^2^; R. Motl^4^; M. Amato^5^; G. Brichetto^6^; D. Boccia^7^; J. Chataway^8^; N. Chiaravalloti^9^; G. Cutter^10^; U. Dalgas^11^; J. DeLuca^9^; R. Farrel^12^; P. Feys^13^; J. Freeman^14^; M. Inglese^15^; C. Meza^16^; A. Salter^17^; B. Sandroff^9^; A. Feinstein^16^; M. Filippi^18^; M. Rocca^3^

##### ^*1*^ *Neuroimaging Research Unit, Division of Neuroscience, IRCCS San Raffaele Scientific Institute, Milan, Italy; and Vita‐Salute San Raffaele University, Milan, Italy;* ^*2*^ *Neuroimaging Research Unit, Division of Neuroscience, IRCCS San Raffaele Scientific Institute, Milan, Italy;* ^*3*^ *Neuroimaging Research Unit, Division of Neuroscience, and Neurology Unit, IRCCS San Raffaele Scientific Institute, Milan, Italy; and Vita‐Salute San Raffaele University, Milan, Italy;* ^*4*^ *Department of Kinesiology and Nutrition, University of Illinois Chicago, Chicago, USA;* ^*5*^ *Department NEUROFARBA, Section Neurosciences, University of Florence, Italy; and IRCCS Fondazione Don Carlo Gnocchi, Florence, Italy;* ^*6*^ *Scientific Research Area, Italian Multiple Sclerosis Foundation (FISM), Genoa, Italy; and AISM Rehabilitation Service, Italian Multiple Sclerosis Society, Genoa, Italy;* ^*7*^ *Department of Neuroscience, Rehabilitation, Ophthalmology, Genetics, Maternal and Child Health, and Center of Excellence for Biomedical Research, University of Genoa, Genoa, Italy;* ^*8*^ *Queen Square Multiple Sclerosis Centre, Department of Neuroinflammation, UCL Queen Square Institute of Neurology, Faculty of Brain Sciences, University College London, London, UK; National Institute for Health Research, University College London Hospital;* ^*9*^ *Kessler Foundation, West Orange, USA; and Department of Physical Medicine & Rehabilitation, Rutgers NJ Medical School, Newark, USA;* ^*10*^ *Department of Biostatistics, University of Alabama at Birmingham, Birmingham, USA;* ^*11*^ *Exercise Biology, Department of Public Health, Aarhus University, Aarhus, Denmark;* ^*12*^ *Queen Square Multiple Sclerosis Centre, Department of Neuroinflammation, UCL Queen Square Institute of Neurology, Faculty of Brain Sciences, University College London, London, UK;* ^*13*^ *REVAL, Faculty of Rehabilitation Sciences, Hasselt University, Diepenbeek, Belgium; UMSC Hasselt, Pelt, Belgium;* ^*14*^ *Faculty of Health, School of Health Professions, University of Plymouth, Devon, UK;* ^*15*^ *Department of Neuroscience, Rehabilitation, Ophthalmology, Genetics, Maternal and Child Health, and Center of Excellence for Biomedical Research, University of Genoa, Genoa, Italy; IRCCS Ospedale Policlinico San Martino, Genoa, Italy;* ^*16*^ *Department of Psychiatry, University of Toronto and Sunnybrook Health Sciences Centre, Toronto, Canada;* ^*17*^ *Department of Neurology, Section on Statistical Planning and Analysis, UT Southwestern Medical Center, Dallas, USA;* ^*18*^ *Neuroimaging Research Unit, Division of Neuroscience, Neurology Unit, Neurorehabilitation Unit, and Neurophysiology Service, IRCCS San Raffaele Scientific Institute, Milan, Italy; Vita‐Salute San Raffaele University, Milan, Italy*

**Background and Aims:** In multiple sclerosis (MS) patients, aerobic exercise may exert a neuroprotective role on the brain by stimulating neurogenesis in the dentate gyrus (DG) of the hippocampus and in the subventricular zone (SVZ), but further evidence is needed. We assessed the effects of aerobic exercise on the volume of the hippocampus and its subfields and on diffusivity measures of the SVZ in progressive MS.

**Methods:** We retrospectively analyzed data from 84 patients with progressive MS enrolled at four sites in the CogEx MRI substudy. Thirty‐nine patients performed aerobic training, while 45 undertook a balance‐ and stretching‐based sham intervention. Both groups trained twice weekly for twelve weeks. At baseline, post‐intervention and 6‐month follow‐up, patients underwent MRI assessment on a 3.0 T scanner. FreeSurfer's longitudinal pipeline was used to analyze hippocampal subfields' volumes. Fractional anisotropy and mean diffusivity were extracted from the SVZ and the thalamus (as control region).

**Results:** No baseline differences were observed between the aerobic and the sham exercise groups (*p* ≥ 0.070). The DG showed a significant volume increase post‐intervention in the aerobic group (mean change = 0.63%, 95% confidence interval [95% CI] = 0.04%; 1.22%, *p* = 0.035), but not in the sham exercise group (mean change = −0.26%, 95% CI = −0.81%; 0.28%, *p* = 0.337). Between‐group difference in DG volume change was significant (*p* = 0.029). No significant changes were found in the other hippocampal subfields or SVZ diffusivity metrics (*p* ≥ 0.057). No changes in either group were found between post‐intervention and 6‐month follow‐up (*p* ≥ 0.061).

**Conclusion:** Aerobic exercise increases DG volume in progressive MS patients but does not modify microstructural integrity of the SVZ.

**Disclosure:** Funding. This study was supported in part by funds from MSCanada (grant EGID 3185). Ancillary funding was provided by the Consortium of Multiple Sclerosis Centres, the Danish Multiple Sclerosis Society, and the US National Multiple Sclerosis Society.

## Headache 1

## OPR‐037

### Migraine in older adults: A multicenter real‐world analysis from the Italian RICe registry

#### G. Paparella ^1^; L. Iannone^2^; G. Paparella^1^; S. Scannicchio^1^; C. Abbatantuono^1^; L. Clemente^1^; M. Delussi^3^; M. Fanelli^4^; M. de Tommaso^1^

##### ^*1*^ *Neurophysiopathology Unit, Department of Translational Biomedicine and Neuroscience (DiBraiN), University of Bari Aldo Moro, Bari, Italy;* ^*2*^ *Department of Biomedical, Metabolic and Neural Sciences, University of Modena and Reggio Emilia, Modena, Italy;* ^*3*^ *Department of Education, Psychology, Communication (For.Psi.Com.), University of Bari Aldo Moro, Bari, Italy;* ^*4*^ *Section of Molecular Pathology, Department of Precision and Regenerative Medicine and Ionian Area (DiMePRe‐J), University of Bari Aldo Moro, Bari, Italy*

**Background and Aims:** Primary headaches, particularly migraine, are prevalent disorders insufficiently characterized in the growing elderly population. This multicentre study provides a evaluation of headache diagnoses, with a focus on migraine, across different age groups in tertiary headache centers participating in the Italian RICe registry.

**Methods:** We retrospectively analyzed 3472 patients evaluated at thirty Headache Centers between 2021 and 2025. Patients were stratified into four age groups: young adults (18–30 years), adults (31–45 years), middle‐aged (46–59 years), and elderly (≥60 years). Data collected included International Headache Society (IHS) headache diagnoses (ICHD3) and demographic and clinical features. Statistical analyses included chi‐square tests and Kruskal–Wallis ANOVAs.

**Results:** Of the total sample, 643 patients (18.5%) were elderly, 1285 (37.0%) middle‐aged, 988 (28.5%) adults, and 556 (16.0%) young adults. The distribution of diagnoses differed significantly across groups [*χ* ^2^(117) = 254, *p* < 0.001]. Elderly patients were more likely to receive a diagnosis of chronic migraine (*z* = 3.41). Migraine with aura was more prevalent in younger individuals (*z* = 5.32). Among other primary headaches, tension‐type headache was more frequent in the elderly (*z* = 2.47). Medication overuse headache (MOH) occurred more often in middle‐aged patients (*z* = 4.64). Older patients reported a longer disease duration, but with less intense pain severity, and fewer associated symptoms (all *p* < 0.01).

**Conclusion:** Headache diagnoses vary significantly with age. In elderly patients, chronic migraine predominates and it is characterized by longer attacks with reduced associated symptoms. MOH represents a relevant concern in middle‐aged individuals. These findings highlight the need for age‐specific diagnostic and therapeutic strategies, particularly in older adults.

**Disclosure:** Nothing to disclose.

## OPR‐038

### Onabotulinum toxin type‐A for the management of medically refractory SUNHA: A preliminary retrospective single centre experience

#### M. Guarinoni ^1^; A. Andreou^2^; M. Murphy^1^; B. Hill^1^; G. Lambru^1^

##### ^*1*^ *Headache and Facial Pain Service, Guy's and St Thomas' NHS Foundation Trust, London, UK;* ^*2*^ *Headache Research‐Wolfson Sensory, Pain and Regeneration Centre, Institute of Psychology, Psychiatry and Neuroscience, King's College London, London, UK*

**Background and Aims:** Short‐lasting unilateral neuralgiform headache attacks (SUNHA) are rare primary head and facial pain disorders that share clinical features and treatment challenges with trigeminal neuralgia (TN). Onabotulinum Toxin A (OnaBoNT) demonstrated promising efficacy in refractory TN. Based on this rationale, we began offering OnaBoNT to SUNHA patients refractory to medical management. Here we aim to evaluate the effectiveness of OnaBoNT in patients with refractory SUNHA.

**Methods:** Medically refractory SUNHA patients diagnosed between January 2024 and January 2026, were offered OnaBoNT using a “follow‐the‐pain protocol”, performed 3–4 months apart. Efficacy outcomes were retrospectively assessed using the Patient Global Impression of Change (PGIC) scale, percentage of symptom improvement, and duration of benefit. Side effects were collated.

**Results:** Of the 214 patients with SUNHA evaluated during the study period, outcomes were available for 18 individuals who received at least one OnaBoNT treatment (Tables 1 and 2). A median dose of 112.5U [IQR 80–145U] was administered per treatment cycle. At 3‐ and 6‐month follow‐ups, 9/18 (50%) and 6/12 (50%) patients respectively reported a PGIC of ≥6 and a ≥50% pain reduction (median: 90%, IQR: 80%–100%). The mean duration of benefit was 9.5 weeks (3–16 weeks). The six responders to the first two treatments displayed a sustained improvement up to their last treatment cycle (range: 5–13 cycles). Mild transient facial asymmetry was reported in 5/18 (28%) patients.

**TABLE 1** Demographic characteristics.

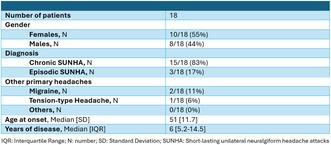

**TABLE 2** Clinical features, previous and current treatment.

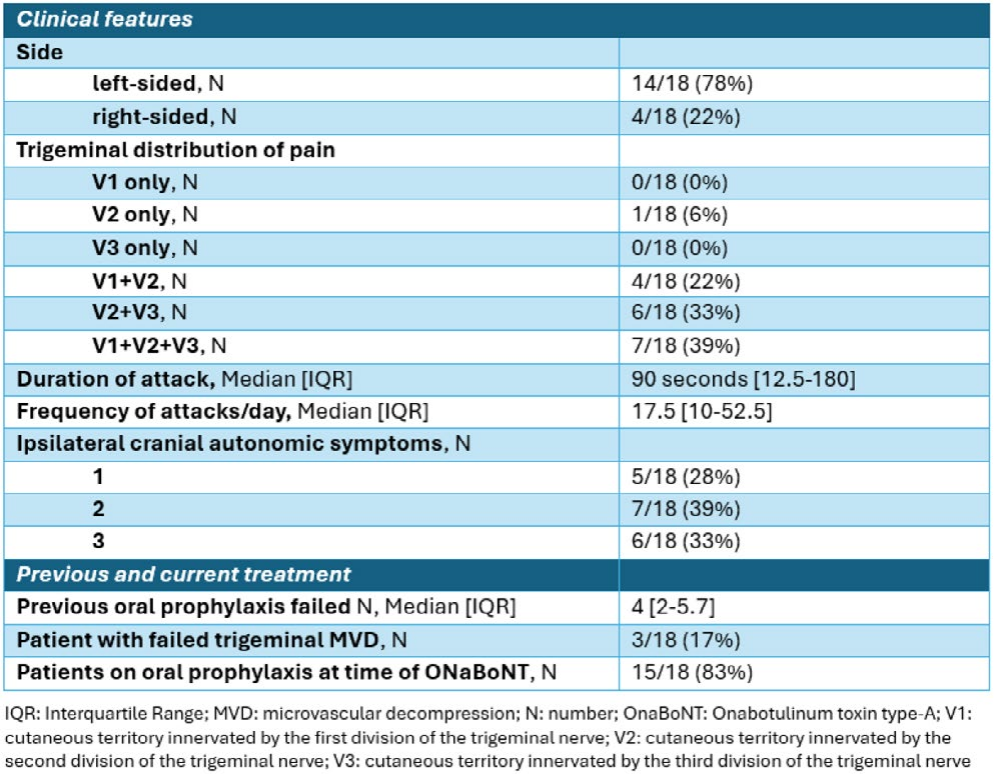

**Conclusion:** Our initial experience with OnaBoNT injected with a “follow‐the‐pain protocol” may indicate its safety and sustained efficacy overtime in a subpopulation of patients with refractory SUNHA.

**Disclosure:** Nothing to disclose.

## OPR‐039

### Predictive factors of response to anti‐CGRP monoclonal antibodies in patients with multiple sclerosis

#### S. Cesarano ^1^; M. Romozzi^2^; V. Nocitti^2^; R. Totaro^3^; M. Albanese^4^; A. Casalena^5^; P. Annovazzi^6^; R. Fantozzi^7^; C. Tortorella^8^; M. Vercellino^9^; L. Iannone^10^; G. De Luca^11^; V. Tommassini^11^; M. Di Filippo^12^; L. Lorefice^13^; G. Maniscalco^14^; D. Paolicelli^15^; F. Pinardi^16^; V. Torri Clerici^17^; C. Solaro^18^; C. Gasperini^8^; P. Calabresi^2^; M. Mirabella^2^; C. Vollono^2^; E. Cocco^13^; D. Centonze^4^; G. Marfia^4^; F. Pistoia^1^

##### ^*1*^ *Department of Biotechnological and Applied Clinical Sciences University of L'Aquila;* ^*2*^ *Dipartimento Universitario di Neuroscienze, Università Cattolica del Sacro Cuore, Rome, Italy;* ^*3*^ *Centro Malattie Demielinizzanti, Clinica Neurologica Ospedale San Salvatore, L'Aquila, Italy;* ^*4*^ *Department of Systems Medicine, University of Rome Tor Vergata, Rome, Italy;* ^*5*^ *Neurologia e Stroke Unit; Centro Cefalee, Azienda Ospedaliera “S. Maria,” Teramo, Italy;* ^*6*^ *Neurologia ad Indirizzo Neuroimmunologico‐Centro Sclerosi Multipla‐ASST della Valle Olona, Ospedale di Gallarate, Italy;* ^*7*^ *Unit of Neurology – IRCCS Neuromed, Pozzilli, Isernia, Italy;* ^*8*^ *Multiple Sclerosis Center, Neurology Unit S. Camillo‐Forlanini Hospital, Rome, Italy;* ^*9*^ *Department of Neuroscience, City of Health and Science University Hospital of Turin, Turin, Italy;* ^*10*^ *Section of Clinical Pharmacology and Oncology, Department of Health Sciences, University of Florence, Florence, Italy;* ^*11*^ *Multiple Sclerosis Center, Policlinico SS. Annunziata, Chieti, Italy;* ^*12*^ *Section of Neurology, Department of Medicine and Surgery, University of Perugia, Italy;* ^*13*^ *Multiple Sclerosis Center, Binaghi Hospital, ASL Cagliari, Italy; Department of Medical Sciences and Public Health, University of Cagliari, Italy;* ^*14*^ *Multiple Sclerosis Center, Cardarelli Hospital, Naples, Italy;* ^*15*^ *Department of Basic Medical Sciences, Neuroscience and Sense Organs, University of Bari “Aldo Moro”, Bari, Italy;* ^*16*^ *Istituto delle scienze neurologiche di Bologna, UOSI Riabilitazione Sclerosi Multipla, Bologna, Italy;* ^*17*^ *Neuroimmunology and Neuromuscular Diseases Unit, Fondazione IRCCS Istituto Neurologico Carlo Besta, Milan, Italy;* ^*18*^ *Rehabilitation Department, Mons. L. Novarese, Moncrivello, Vercelli, Italy*

**Background and Aims:** Migraine is common in people with multiple sclerosis (PwMS) and substantially contributes to disability and impaired quality of life. Although calcitonin gene‐related peptide (CGRP)–targeting therapies have reshaped migraine prevention, evidence on their use in PwMS remains scarce, particularly in patients receiving concomitant disease‐modifying therapies (DMTs).

**Methods:** We retrospectively collected data from 17 Italian MS centers on adult PwMS with comorbid migraine treated with anti‐CGRP monoclonal antibodies or atogepant in addition to stable DMTs. Monthly headache days (MHDs) and analgesic intake were compared between treatment initiation and last available follow‐up. MS activity was assessed through relapses, Expanded Disability Status Scale (EDSS), and MRI findings. A ≥50% reduction in MHDs defined treatment response. Multivariate regression models were used to explore predictors of clinical improvement.

**Results:** Fifty‐four patients were included (46 women; mean age 42.3 years; 85% relapsing MS). Baseline MHDs averaged 19.9 ± 7.0 and declined to 11.4 ± 9.4 at follow‐up (*p* < 0.001), with a parallel decrease in analgesic use (*p* < 0.001). A responder rate of 53.7% was observed. MS disease activity remained stable, with no significant changes in relapse frequency, EDSS, or MRI activity. Higher baseline headache burden was associated with greater reduction in MHDs (β = 0.64, *p* < 0.001), whereas longer MS duration predicted poorer response (OR = 1.70, 95% CI 1.11–2.63). Mild adverse events occurred in four patients, without treatment discontinuation.

**TABLE 1** Patient demographics and clinical features at baseline.

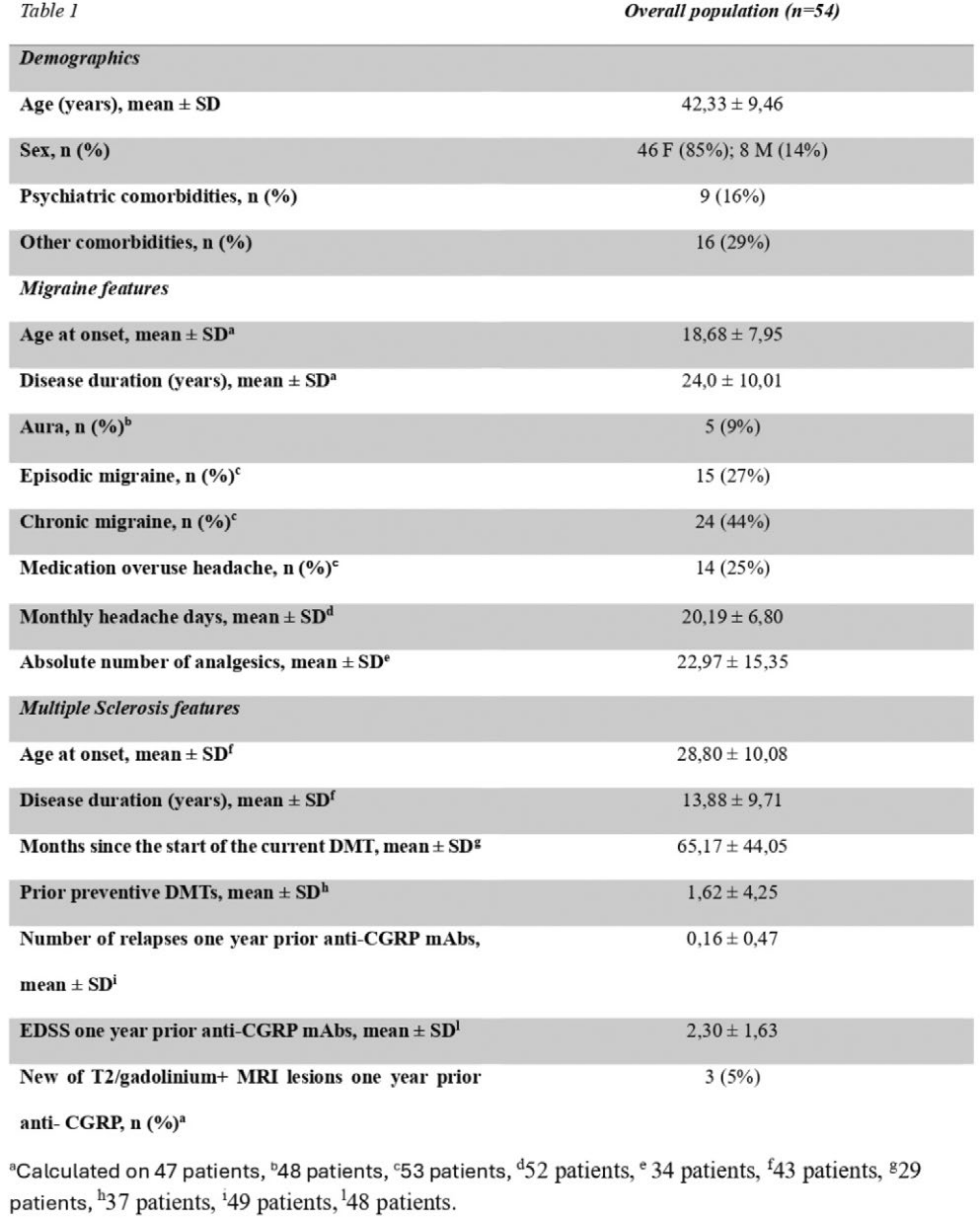

**TABLE 2** Patient demographics and clinical features at baseline: Responders vs. non responders.

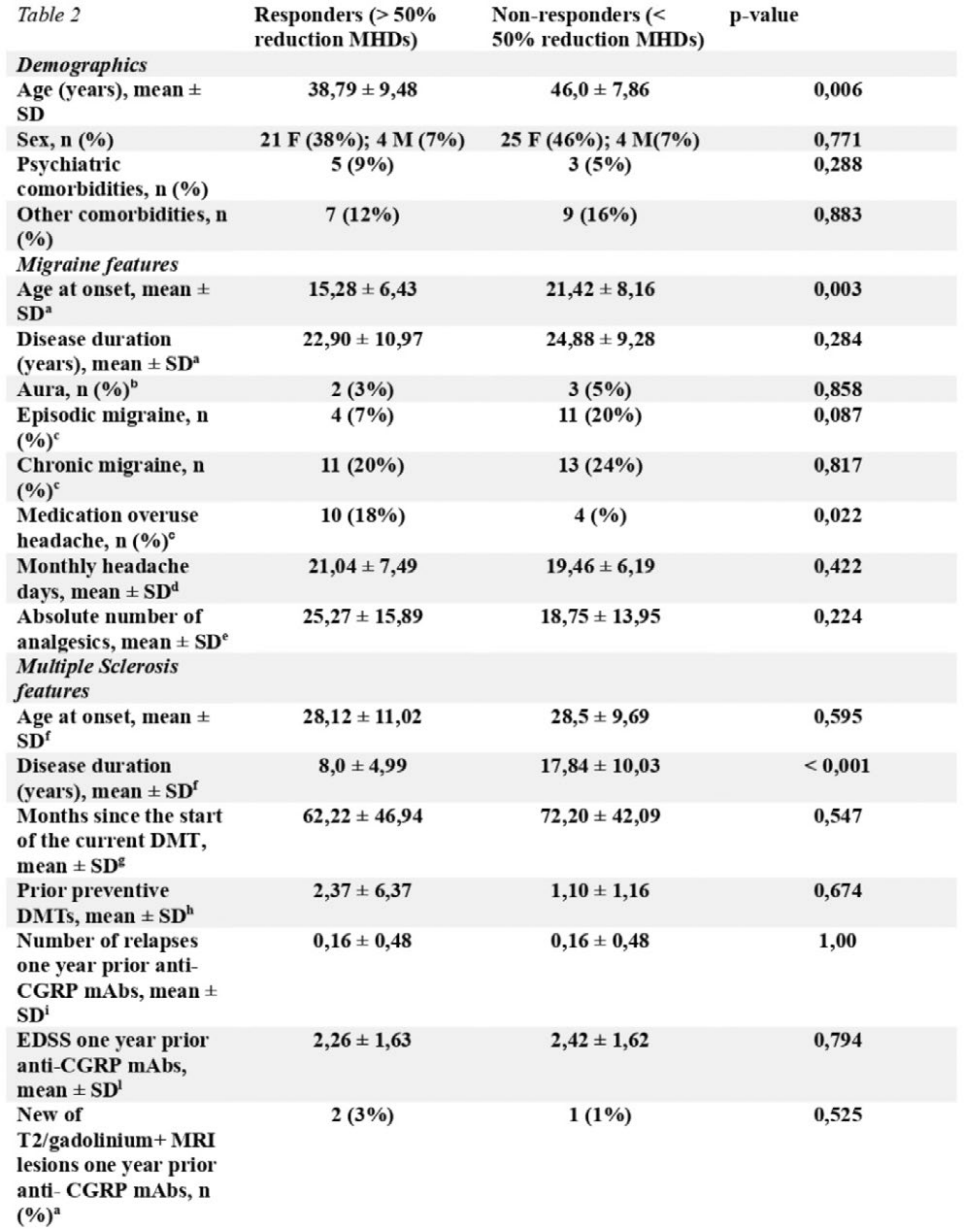

**Figure:**
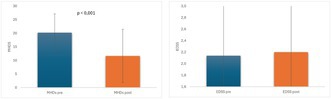
**FIGURE 1** Number of MHD and EDSS score at baseline, and the last follow‐up during treatment with CGRP‐mAbs in combination with DMT for multiple sclerosis.

**Conclusion:** In this real‐world multicenter cohort, anti‐CGRP therapies provided meaningful migraine improvement in PwMS while maintaining MS stability. Disease duration and baseline headache frequency may influence therapeutic response.

**Disclosure:** Nothing to disclose.

## OPR‐040

### Randomised controlled trial with translational validation: Migraine induced by vascular KATP channel activation is independent of HCN channel activity

#### Z. Zhuang ^1^; J. Thuraiaiyah^1^; L. Kokoti^1^; R. Moreno^1^; E. Gozalov^1^; Z. Hakimzadeh^1^; M. Al‐Karagholi^1^; A. Clement^2^; D. Kristensen^2^; J. Abdulla^3^; S. Christensen^2^; M. Ashina^4^

##### ^*1*^ *Department of Neurology, Danish Headache Centre, Copenhagen University Hospital – Rigshospitalet, Copenhagen, Denmark;* ^*2*^ *Translational Research Centre, Copenhagen University Hospital – Rigshospitalet, Copenhagen, Denmark;* ^*3*^ *Department of Cardiology, Amager‐Hvidovre Hospital, Copenhagen, Denmark;* ^*4*^ *Centre for Discoveries in Migraine, Danish Headache Centre, Copenhagen University Hospital – Rigshospitalet, Copenhagen, Denmark*

**Background and Aims:** Vascular ATP‐sensitive potassium (KATP) channel activation can trigger migraine, but the mechanisms remain unclear. Proposed pathways involve either direct neuronal activation via hyperpolarisation‐activated cyclic nucleotide–gated (HCN) channels or signalling at the vessel–to–neuron interface. We tested whether HCN channel blockade alters KATP channel‐induced migraine.

**Methods:** We conducted a single‐centre, randomised, double‐blind, placebo‐controlled, 2‐way crossover study in individuals with migraine without aura and mechanistically aligned preclinical experiments in a validated mouse model. Participants received intravenous levcromakalim followed by oral ivabradine or placebo on separate days. The primary outcome was the 12‐h incidence of levcromakalim‐induced migraine; secondary outcomes included area under the curve (AUC) for headache intensity and haemodynamic responses. In mice, von Frey testing was used to assess whether ivabradine, given as pre‐ or rescue treatment, modified levcromakalim‐induced hypersensitivity.

**Results:** Twenty‐seven participants completed the human study and were included in the analysis. Ivabradine had no effect on the incidence of levcromakalim‐induced migraine (22/27 after ivabradine and 22/27 after placebo; *p* > 0.99) or on AUC for headache intensity (*p* = 0.11). Haemodynamic responses did not differ between study days. In mice, multiple doses of ivabradine neither prevented nor reversed tactile hypersensitivity caused by repeated levcromakalim administration.

**Conclusion:** HCN channel blockade does not affect migraine or nociceptive behaviour triggered by vascular KATP channel activation, suggesting that HCN channels are not required for translating vascular KATP channel activation into migraine pain. Instead, our findings support a model in which migraine is initiated via signalling at the vessel‐to‐neuron interface.

**Disclosure:** The authors have no conflicts of interest relevant to the content of this study. The study was supported by the Danish National Research Foundation (DNRF188) and the Lundbeck Foundation Professor Grant (R310‐2018‐371 1), with no role of funders in study design, conduct, data collection, analysis, interpretation, or manuscript preparation. Additional support include: Z. A. Z., Lundbeck Foundation Scholarship (Danish Neurological Society) and Rigshospitalets Forskningspuljer (E‐23327‐03); A. C., Foreningen til støtte af forskning ved Dansk Hovedpine Center; S. L. C., Candys Foundation and BRIDGE – Translational Excellence Programme, Faculty of Health and Medical Sciences, University of Copenhagen, supported by Novo Nordisk Foundation (NNF23SA0087869).

## Neuroimaging 1

## OPR‐041

### APOE ε4 gene dose is associated with more typical and homogeneous amyloid deposition across the Alzheimer's disease continuum

#### A. Mendes ^1^; C. Boccalini^2^; F. Ribaldi^1^; M. Pievani^3^; D. Peretti^2^; V. Garibotto^2^; A. Chincarini^4^; G. Frisoni^1^

##### ^*1*^ *Laboratory of Neuroimaging of Aging (LANVIE), University of Geneva, Geneva, Switzerland;* ^*2*^ *Laboratory of Neuroimaging and Innovative Molecular Tracers (NIMTlab), Geneva University Neurocenter and Faculty of Medicine, University of Geneva, Geneva, Switzerland;* ^*3*^ *Laboratory of Alzheimer's Neuroimaging and Epidemiology (LANE), IRCCS Istituto Centro San Giovanni di Dio Fatebenefratelli, Brescia, Italy;* ^*4*^ *National Institute of Nuclear Physics (INFN), Genoa section, Genoa, Italy*

**Background and Aims:** Although amyloid deposition is a core pathological feature of Alzheimer's disease (AD), its spatial expression varies widely across individuals. The probabilistic amyloid hypothesis predicts that APOE ε4 constrains amyloid accumulation toward more stereotyped spatial patterns. Whether APOE ε4 gene dose increases the predictability and homogeneity of amyloid topography across disease stages remains unknown.

**Methods:** We performed voxel‐wise similarity and spatial homogeneity analyses of amyloid‐PET data in amyloid‐positive participants from ADNI (CU, MCI, dementia). Participants were stratified by APOE ε4 genotype. Individual amyloid‐PET maps were correlated with two independent canonical AD amyloid reference maps. Within‐group spatial homogeneity was quantified using inter‐subject voxel‐wise correlation matrices. Replication was performed in an independent cohort from the Geneva Memory Center.

**Results:** APOE ε4 genotype predicted amyloid‐PET pattern similarity to canonical AD maps, independent of age, sex, and global amyloid burden. Similarity increased in a dose‐dependent manner from non‐carriers to heterozygotes and homozygotes. APOE ε4 carriers also showed significantly greater within‐group spatial homogeneity of amyloid deposition, with the highest convergence observed in homozygotes. These effects were most pronounced in CU and MCI stages but persisted across the AD continuum. All findings replicated across cohorts, PET tracers, and canonical reference maps.

**Conclusion:** The APOE ε4 gene dose is associated with increasingly predictable and homogeneous amyloid topographies, supporting a gradient of pathological determinism in AD. These results provide in vivo support for the probabilistic model of AD and suggest that APOE ε4 homozygosity drives the topographical predictability and homogeneity of amyloid deposition, with implications for targeted anti‐amyloid interventions.

**Disclosure:** GBF has received consulting fees through his institution from Biogen, Diadem, Roche, Eisai, Eli Lilly, Ac Immune, Novo Nordisk, Schwabe, Bromatech, AtonRâ, World Clinical Trials, and J&J Innovative Medicine. GBF has received payment or honoraria for lectures, presentations, speakers bureaus, manuscript writing, or educational events through his institution from Biogen, Roche, Novo Nordisk, GE HealthCare, and Vifor Pharma. VG received research support and speaker fees through her institution from GE Healthcare, Siemens Healthineers, Novo Nordisk, Janssen and Novartis. All other authors have nothing to disclose.

## OPR‐042

### Functional connectivity reveals gene expression‐driven network vulnerability across frontotemporal lobar degeneration variants

#### F. Orlandi ^1^; E. Spinelli^1^; A. Ghirelli^1^; S. Basaia^2^; V. Castelnovo^2^; E. Canu^3^; G. Cecchetti^4^; F. Caso^5^; G. Magnani^5^; T. Domi^6^; L. Pozzi^6^; P. Carrera^7^; F. Agosta^1^; M. Filippi^8^

##### ^*1*^ *Neuroimaging Research Unit, Division of Neuroscience, and Neurology Unit, IRCCS San Raffaele Scientific Institute, Milan, Italy; Vita‐Salute San Raffaele University, Milan, Italy;* ^*2*^ *Neuroimaging Research Unit, Division of Neuroscience, IRCCS San Raffaele Scientific Institute, Milan, Italy;* ^*3*^ *Neuroimaging Research Unit, Division of Neuroscience, Neurology Unit, IRCCS San Raffaele Scientific Institute, Milan, Italy;* ^*4*^ *Neuroimaging Research Unit, Division of Neuroscience, Neurology Unit, and Neurophysiology Service, IRCCS San Raffaele Scientific Institute, Milan, Italy;* ^*5*^ *Neurology Unit, IRCCS San Raffaele Scientific Institute, Milan, Italy;* ^*6*^ *Experimental Neuropathology Unit, Division of Neuroscience, Institute of Experimental Neurology, IRCCS San Raffaele Scientific Institute, Milan, Italy;* ^*7*^ *Unit of Genomics for Human Disease Diagnosis, IRCCS San Raffaele Scientific Institute, Milan, Italy;* ^*8*^ *Neuroimaging Research Unit, Division of Neuroscience, Neurology Unit, Neurorehabilitation Unit, Neurophysiology Service, IRCCS San Raffaele Scientific Institute, Milan, Italy; Vita‐Salute San Raffaele University, Milan, Italy*

**Background and Aims:** Frontotemporal lobar degeneration (FTLD) includes several syndromes with specific regional susceptibility. How molecular architecture shapes degeneration across functional networks remains unclear. Stepwise functional connectivity (SFC) captures how disease epicenters communicate across network distances. We investigated whether transcriptomic profiles of key FTLD‐associated genes align with SFC patterns.

**Methods:** Variant‐specific atrophy epicenters were identified using voxel‐based morphometry in patients with behavioral variant FTD (bvFTD, *n* = 11), semantic variant PPA (svPPA, *n* = 18), nonfluent variant PPA (nfvPPA, *n* = 14), and semantic behavioral variant FTD (sbvFTD, *n* = 15). Regional SFC topological distances from epicenters were derived using resting‐state fMRI in 50 healthy controls. Spatial correlations were computed between SFC distance and gene expression maps from Allen Human Brain Atlas. Genes enriched near epicenters underwent pathway analysis.

**Results:** Epicenters were located in the left anterior insula (bvFTD), left anterior inferior temporal gyrus (svPPA), left supplementary motor area (nfvPPA), and right temporal pole (sbvFTD). GRN and MAPT were enriched near epicenters, whereas C9orf72 expression increased with SFC distance. Pathway analysis identified two molecular clusters: bvFTD/nfvPPA‐associated genes were linked to mitochondrial metabolism, oxidative stress, and proteostasis; svPPA/sbvFTD‐associated genes were associated with neuromodulation and synaptic plasticity.

**Conclusion:** Regional transcriptomic patterns reflect functional network architecture in FTLD, suggesting molecular vulnerability shapes network‐specific degeneration. GRN and MAPT enrichment near epicenters indicates local susceptibility, while C9orf72 patterns suggest distinct propagation. Variant‐associated clusters may reflect differential vulnerability to tau or TDP‐43 pathology, potentially explaining clinical and anatomical differences.

**Disclosure:** Supported by European Research Council (StG‐2016_714388_NeuroTRACK); Foundation Research on Alzheimer Disease; and Next Generation EU [DM 1557 11.10.2022]. Francesca Orlandi, Edoardo Gioele Spinelli, Alma Ghirelli, Silvia Basaia, Veronica Castelnovo, Elisa Canu, Francesca Caso, Giuseppe Magnani, Teuta Domi, Laura Pozzi, and Paola Carrera have nothing to disclose. Giordano Cecchetti has received speaker honoraria from Neopharmed Gentili; F. Agosta is Associate Editor of NeuroImage: Clinical and the European Journal of Neurology; has received speaker honoraria from Biogen Idec, Bristol Myers Squibb, Eisai, Eli Lilly, GE Healthcare, Neuraxpharm, and Roche; and receives or has received research supports from the Italian Ministry of Health, the Italian Ministry of University and Research, AriSLA (Fondazione Italiana di Ricerca per la SLA), the European Research Council (ERC), the EU Joint Programme – Neurodegenerative Disease Research (JPND), and Foundation Research on Alzheimer Disease (France). M. Filippi is Editor‐in‐Chief of the Journal of Neurology, Associate Editor of Human Brain Mapping, Neurological Sciences, and Radiology; received compensation for consulting services from Almirall, Biogen, Bristol‐Myers Squibb, Eli Lilly, Merck, Novartis, Roche, Sanofi; speaking activities from Amgen, Bayer, Biogen, Bristol‐Myers Squibb, Celgene, Chiesi Italia SpA, Eisai, Eli Lilly, Fujirebio, Genzyme, Janssen, Merck, Neopharmed Gentili, Neuraxpharm, Novartis, Novo Nordisk, Roche, Sanofi, Takeda; participation in Advisory Boards for Alexion, Biogen, Bristol‐Myers Squibb, Eli Lilly, GE Healthcare Ltd, Merck, Neuraxpharm, Novartis, Roche, Sandoz, Sanofi, Takeda; scientific direction of educational events for Biogen, Merck, Roche, Celgene, Bristol‐Myers Squibb, Lilly, Novartis, Sanofi‐Genzyme; he receives research support from Biogen Idec, Merck‐Serono, Novartis, Roche, the Italian Ministry of Health, the Italian Ministry of University and Research, and Fondazione Italiana Sclerosi Multipla.

## OPR‐043

### Glymphatic impairment in GRN‐mutated frontotemporal dementia: A distinct pathophysiological signature

#### I. Bottale ^1^; E. Spinelli^1^; A. Ghirelli^1^; S. Basaia^2^; T. Battani^3^; V. Castelnovo^2^; E. Canu^4^; G. Cecchetti^5^; F. Caso^6^; G. Magnani^6^; T. Domi^7^; L. Pozzi^7^; P. Carrera^8^; M. Filippi^9^; F. Agosta^1^

##### ^*1*^ *Neuroimaging Research Unit, Division of Neuroscience, and Neurology Unit, IRCCS San Raffaele Scientific Institute, Milan, Italy; Vita‐Salute San Raffaele University, Milan, Italy;* ^*2*^ *Neuroimaging Research Unit, Division of Neuroscience, IRCCS San Raffaele Scientific Institute, Milan, Italy;* ^*3*^ *Vita‐Salute San Raffaele University, Milan, Italy;* ^*4*^ *Neuroimaging Research Unit, Division of Neuroscience, and Neurology Unit, IRCCS San Raffaele Scientific Institute, Milan, Italy;* ^*5*^ *Neuroimaging Research Unit, Division of Neuroscience, Neurology Unit, and Neurophysiology Service, IRCCS San Raffaele Scientific Institute, Milan, Italy;* ^*6*^ *Neurology Unit, IRCCS San Raffaele Scientific Institute, Milan, Italy;* ^*7*^ *Experimental Neuropathology Unit, Division of Neuroscience, Institute of Experimental Neurology, IRCCS San Raffaele Scientific Institute, Milan, Italy;* ^*8*^ *Unit of Genomics for Human Disease Diagnosis, IRCCS San Raffaele Scientific Institute, Milan, Italy;* ^*9*^ *Neuroimaging Research Unit, Division of Neuroscience, Neurology Unit, Neurorehabilitation Unit, and Neurophysiology Service, IRCCS San Raffaele Scientific Institute, Milan, Italy; Vita‐Salute San Raffaele University, Milan, Italy*

**Background and Aims:** Diffusion tensor imaging analysis along the perivascular space (DTI‐ALPS) is a non‐invasive MRI‐based proxy of glymphatic dysfunction, a mechanism implicated in the accumulation of protein aggregates in several neurodegenerative disorders. This study aimed to evaluate glymphatic function using DTI‐ALPS in GRN‐mutated frontotemporal dementia (FTD), comparing them to sporadic cases and healthy controls (HC), and investigate its clinical relevance and relationship with white‐matter (WM) damage.

**Methods:** Eighteen GRN‐mutated patients with an FTD syndrome (10 behavioural variant FTD [bvFTD], 5 non‐fluent primary progressive aphasia [nfvPPA], 3 other PPA variants), 43 sporadic FTD patients, and 32 age‐ sex‐matched HC underwent 3T MRI. The DTI‐ALPS index was calculated for each participant. Group comparisons were performed using age‐ sex‐ and fractional anisotropy (FA)‐adjusted ANCOVA. Associations with cognitive performance, behavioural symptoms, and sleep disturbances were assessed. WM microstructural integrity was evaluated using tract‐based spatial statistics with voxel‐wise FA.

**Results:** GRN‐patients showed significantly lower DTI‐ALPS values than HC (*p* < 0.001) and sporadic FTD patients with bvFTD (*p* = 0.027) and semantic variant of PPA (*p* = 0.003). Within the GRN‐group, lower DTI‐ALPS was associated with poorer executive performance, higher impairment on selected Frontal Behavioural Inventory items, and the presence of insomnia (*p* = 0.025). DTI‐ALPS values positively correlated with FA in the anterior and superior corona radiata, internal and external capsules, corpus callosum, and left superior longitudinal fasciculus (*p* < 0.05, Family‐Wise Error corrected for multiple comparisons).

**Conclusion:** Glymphatic dysfunction is more pronounced in GRN‐FTD than in sporadic FTD and is associated with WM damage, executive, behavioural alterations, and sleep disturbances, supporting a gliovascular contribution to GRN‐mediated neurodegeneration.

**Disclosure:** Supported by European Research Council (StG‐2016_714388_NeuroTRACK); Next Generation EU, in the context of the National Recovery and Resilience Plan, Investment PE8 – Project Age‐It: “Ageing Well in an Ageing Society; Foundation Research on Alzheimer Disease”. I. Bottale, T. Battani, A. Ghirelli, V. Castelnovo, F. Caso, G. Magnani, T. Domi, L. Pozzi, and P. Carrera report no competing interests. E.G. Spinelli and G. Cecchetti have received speaker honoraria from Eli Lilly. E. Canu and S. Basaia received research support from the Italian Ministry of Health. M. Filippi is Editor‐in‐Chief of the Journal of Neurology, Associate Editor of Human Brain Mapping, Neurological Sciences, and Radiology; received compensation for consulting services from Almirall, Biogen, Bristol‐Myers Squibb, Eli Lilly, Merck, Novartis, Roche, Sanofi; speaking activities from Amgen, Bayer, Biogen, Bristol‐Myers Squibb, Celgene, Chiesi Italia SpA, Eisai, Eli Lilly, Fujirebio, Genzyme, Janssen, Merck, Neopharmed Gentili, Neuraxpharm, Novartis, Novo Nordisk, Roche, Sanofi, Takeda; participation in Advisory Boards for Alexion, Biogen, Bristol‐Myers Squibb, Eli Lilly, GE Healthcare Ltd, Merck, Neuraxpharm, Novartis, Roche, Sandoz, Sanofi, Takeda; scientific direction of educational events for Biogen, Merck, Roche, Celgene, Bristol‐Myers Squibb, Lilly, Novartis, Sanofi‐Genzyme; he receives research support from Biogen Idec, Merck‐Serono, Novartis, Roche, the Italian Ministry of Health, the Italian Ministry of University and Research, and Fondazione Italiana Sclerosi Multipla.F. Agosta is Associate Editor of NeuroImage: Clinical and the European Journal of Neurology; has received speaker honoraria from Biogen Idec, Bristol Myers Squibb, Eisai, Eli Lilly, GE Healthcare, Neuraxpharm, and Roche; and receives or has received research supports from the Italian Ministry of Health, the Italian Ministry of University and Research, AriSLA (Fondazione Italiana di Ricerca per la SLA), the European Research Council (ERC), the EU Joint Programme – Neurodegenerative Disease Research (JPND), and Foundation Research on Alzheimer Disease (France).

## OPR‐044

### Metabolically defined lesion clusters show differential associations with clinical disability in multiple sclerosis

#### R. Rumbak ^1^; A. Petrova^1^; A. Kloss‐Brandstätter^2^; F. Niess^1^; B. Strasser^1^; L. Hingerl^1^; T. Berger^3^; G. Grabner^4^; P. Rommer^3^; W. Bogner^1^; E. Niess^1^; A. Dal‐Bianco^3^

##### ^*1*^ *High‐field MR Center, Department of Biomedical Imaging and Image‐guided Therapy, Medical University of Vienna, Vienna, Austria;* ^*2*^ *Department of Engineering & IT, Carinthia University of Applied Sciences, Villach, Austria;* ^*3*^ *Department of Neurology, Medical University of Vienna, Vienna, Austria;* ^*4*^ *Department of Medical Engineering, Carinthia University of Applied Sciences, Klagenfurt, Austria*

**Background and Aims:** Lesion burden on conventional magnetic resonance imaging correlates only weakly with clinical disability in multiple sclerosis (MS). We investigated whether metabolically defined lesion clusters identified by 7T MR spectroscopic imaging show differential associations with clinical disability.

**Methods:** In this cross‐sectional observational study, 50 people with MS underwent 7T MR spectroscopic imaging. After quality control, 304 white matter lesions from 45 participants were analysed using metabolite ratios reflecting lesion metabolic state. Data‐driven clustering identified metabolically distinct lesion clusters. Lesion burden within each cluster was correlated with clinical disability measures, including the Expanded Disability Status Scale (EDSS), Symbol Digit Modalities Test (SDMT), Timed 25‐Foot Walk (T25FW), and 9‐Hole Peg Test (9‐HPT), using age‐adjusted models.

**Results:** Three reproducible metabolic lesion clusters were identified (Figure 1). Cluster 1, consistent with newly emerging, actively inflammatory and expanding lesions, showed the strongest clinical associations, with higher lesion count correlating with worse SDMT performance (*r* = −0.34, *p* = 0.027). Cluster 2, reflecting metabolically exhausted lesions with ongoing tissue damage, was associated with impaired motor function, including slower T25FW (*r* = 0.31, *p* = 0.044) and 9‐HPT (*r* = 0.29, *p* = 0.031). Cluster 3, characterized by metabolically stable lesions with features consistent with partial repair, showed no significant associations with disability (*p* > 0.1). Associations between cluster‐specific lesion burden and disability are illustrated in Figure 2.

**Figure:**
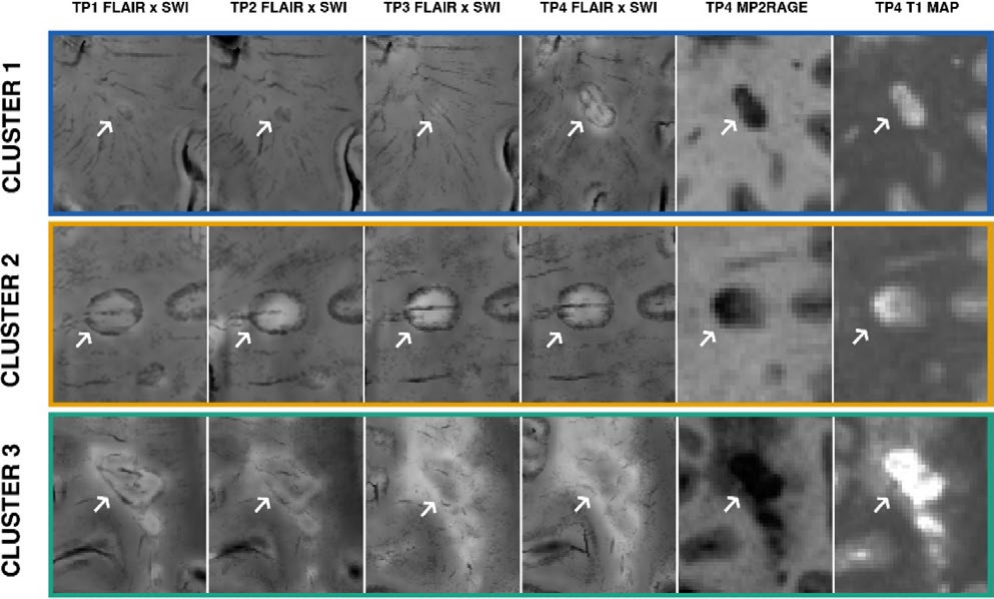
**FIGURE 1** Longitudinal examples of metabolically clustered lesions (Clusters 1–3) across coregistered FLAIR + SWI, MP2RAGE, and quantitative T1 maps from baseline to 8‐year follow‐up.

**Figure:**
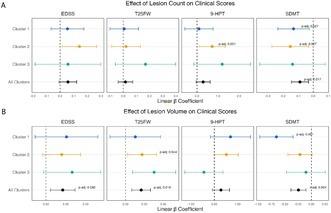
**FIGURE 2** Linear regression models assessed the effect of lesion count (A) and log‐transformed lesion volume (cm^3^) on disability.

**Conclusion:** These findings indicate that lesion metabolic state, rather than lesion presence alone, determines clinical relevance, supporting metabolic imaging as a tool for improved stratification of disease impact.

**Disclosure:** This research was funded in whole, or in part, by the Austrian Science Fund (FWF): [10.55776/DFH50] and Austrian MS Gesellschaft.

## Sunday, June 28 2026

## MS and Related Disorders 2

## OPR‐045

### Early cognitive trajectories over 5 years following the first demyelinating event and their baseline magnetic resonance imaging predictors

#### E. Olivieri ^1^; P. Ananthavarathan^1^; M. Pitteri^2^; S. Collorone^1^; M. Foster^1^; F. Prados^1^; B. Kanber^3^; S. Salama^1^; F. Barkhof^3^; C. Gandini Wheeler‐Kingshott^1^; D. Chard^1^; O. Ciccarelli^1^; A. Toosy^1^

##### ^*1*^ *NMR Research Unit, Queen Square MS Centre, Department of Neuroinflammation, UCL Queen Square Institute of Neurology, Faculty of Brain Sciences, University College London, London, UK;* ^*2*^ *Department of Neuropsychology, National Hospital for Neurology and Neurosurgery, London, UK;* ^*3*^ *Centre for Medical Image Computing (CMIC), Department of Medical Physics and Biomedical Engineering, University College London, London, UK*

**Background and Aims:** Cognitive decline in early MS remains understudied. We investigated longitudinal cognitive trajectories and baseline magnetic resonance imaging (MRI) predictors following the first demyelinating event.

**Methods:** Patients were enrolled within 3 months of symptom onset and followed for 5 years. Baseline MRI metrics included brain matter fractions and neurite orientation dispersion and density imaging (NODDI) parameters, specifically neurite density index (NDI), orientation dispersion index (ODI) and isotropic volume fraction (Viso). The Brief Visuospatial Memory Test‐Revised (BVMT‐R), California Verbal Learning Test (CVLT) and Symbol Digit Modalities Test (SDMT) were collected at each visit. Quadratic and single‐knot piecewise linear mixed models evaluated the impact of baseline MRI metrics on cognitive trajectories.

**Results:** 100 patients (mean age 32.3 ± 7.5 years, 33% male) were included, 35% of whom had MS at baseline. BVMT‐R and CVLT improved until year 3 by 0.15 (SE: 0.03; *p* < 0.001) and 0.21 (SE: 0.04; *p* < 0.001) *z*‐scores/year, respectively, before declining. SDMT remained steady throughout the study period. A 1% decrease in deep grey matter fraction (DGMF) predicted 0.92 (*p* = 0.009, SE = 0.35) and 1.11 (*p* = 0.022, SE = 0.48) *z*‐scores/year declines in BVMT‐R and CVLT, respectively, while a 0.1‐unit increase in cortical grey matter isotropic volume fraction (CGMViso) predicted a 0.47 *z*‐score/year decline in BVMT‐R (*p* = 0.003, SE = 0.15).

**Conclusion:** After the first demyelinating event, patients showed initial cognitive improvement followed by decline in multiple domains. Baseline DGMF and CGMViso predicted this decline, highlighting their value as prognostic markers for cognitive trajectories in early MS.

**Disclosure:** Nothing to disclose.

## OPR‐046

### Lifespan modelling of choroid plexus volume in multiple sclerosis and its dynamic associations with clinical, magnetic resonance imaging and human leukocyte antigen susceptibility

#### G. Corazzolla ^1^; P. Preziosa^1^; A. Meani^2^; M. Margoni^3^; L. Storelli^2^; E. Pagani^2^; M. Rubin^1^; F. Clarelli^4^; E. Mascia^4^; M. Sorosina^4^; F. Esposito^5^; M. Rocca^1^; M. Filippi^6^

##### ^*1*^ *Neuroimaging Research Unit, Division of Neuroscience, and Neurology Unit, IRCCS San Raffaele Scientific Institute, Milan, Italy; Vita‐Salute San Raffaele University, Milan, Italy;* ^*2*^ *Neuroimaging Research Unit, Division of Neuroscience, IRCCS San Raffaele Scientific Institute, Milan, Italy;* ^*3*^ *Neuroimaging Research Unit, Division of Neuroscience, Neurology Unit, and Neurorehabilitation Unit, IRCCS San Raffaele Scientific Institute, Milan, Italy;* ^*4*^ *Laboratory of Human Genetics of Neurological Disorders, IRCCS San Raffaele Scientific Institute, Milan, Italy;* ^*5*^ *Neurology Unit, and Laboratory of Human Genetics of Neurological Disorders, IRCCS San Raffaele Scientific Institute, Milan, Italy;* ^*6*^ *Neuroimaging Research Unit, Division of Neuroscience, Neurology Unit, Neurorehabilitation Unit, and Neurophysiology Service, IRCCS San Raffaele Scientific Institute, Milan, Italy; Vita‐Salute San Raffaele University, Milan, Italy*

**Background and Aims:** The choroid plexus (ChP) regulates cerebrospinal fluid production and central nervous system homeostasis. In multiple sclerosis (MS), ChP enlargement occurs early in the disease course, yet its normative lifespan trajectory and disease‐specific associations remain unclear. We defined normative ChP volume trajectories across the healthy lifespan and assessed ChP enlargement in MS to evaluate associations with demographic, clinical, MRI, and genetic variables, including Human Leukocyte Antigen (HLA) and non‐HLA polygenic risk scores (PRSs).

**Methods:** This monocentric retrospective study included 461 healthy controls (HC) and 727 MS patients (age 18–70 years) who underwent 3T brain MRI and neurological assessment. ChP volumes were quantified using ASCHOPLEX, normalized for head size, and modeled in HC to compute *z*‐scores for MS patients. PRSs were calculated from established MS susceptibility loci, encompassing both HLA and non‐HLA regions.

**Results:** In HC, normalized ChP volume (NChPV) was predicted by normalized brain volume, lateral ventricle volume, and its squared term (*R* ^2^ = 0.54), remaining stable until age 35, then increasing nonlinearly (p‐FDR < 0.002). MS patients had significantly higher NChPV *z*‐scores than controls (p‐FDR < 0.001), independent of age or phenotype. NChPV *z*‐scores rose during the first five years after onset (p‐FDR = 0.012–0.015) before plateauing. Higher *z*‐scores were associated with greater T2‐hyperintense white matter lesion volume (*p* < 0.001) and HLA genetic burden (*p* = 0.027) but not non‐HLA PRSs (*p* ≥ 0.177).

**Conclusion:** We provided normative ChP trajectories and showed early, sustained ChP enlargement in MS, linked to inflammatory lesion burden and HLA‐mediated genetic risk. The ChP may represent a non‐invasive biomarker of MS susceptibility and neuroinflammation.

**Disclosure:** P. Preziosa received speaker honoraria from Roche, Biogen, Novartis, Merck, Bristol Myers Squibb, Genzyme, Horizon and Sanofi. G. Corazzolla, A. Meani, L. Storelli, E. Pagani, M. Rubin, F. Clarelli, E. Mascia, M. Sorosina, F. Esposito have nothing to disclose. M. Margoni reports grants and personal fees from Sanofi Genzyme, Merck Serono, Roche, Biogen, Amgen and Novartis. M.A. Rocca received consulting fees from Biogen, Bristol Myers Squibb, Roche; and speaker honoraria from Alexion, Biogen, Bristol Myers Squibb, Celgene, Horizon Therapeutics Italy, Merck Serono SpA, Mitsubishi‐Tanabe Pharma, Neuraxpharm, Novartis, Roche, Sandoz, and Sanofi. She receives research support from the MS Society of Canada, the Italian Ministry of Health, the Italian Ministry of University and Research, and Fondazione Italiana Sclerosi Multipla. She is Associate Editor for Multiple Sclerosis and Related Disorders; and Associate Co‐Editor for Europe and Africa for Multiple Sclerosis Journal. M. Filippi is Editor‐in‐Chief of the Journal of Neurology, Associate Editor of Human Brain Mapping, Neurological Sciences, and Radiology; received compensation for consulting services from Almirall, Biogen, Bristol‐Myers Squibb, Eli Lilly, Merck, Novartis, Roche, Sanofi; speaking activities from Amgen, Bayer, Biogen, Bristol‐Myers Squibb, Celgene, Chiesi Italia SpA, Eisai, Eli Lilly, Fujirebio, Genzyme, Janssen, Merck, Neopharmed Gentili, Neuraxpharm, Novartis, Novo Nordisk, Roche, Sanofi, Takeda; participation in Advisory Boards for Alexion, Biogen, Bristol‐Myers Squibb, Eli Lilly, GE Healthcare Ltd, Merck, Neuraxpharm, Novartis, Roche, Sandoz, Sanofi, Takeda; scientific direction of educational events for Biogen, Merck, Roche, Celgene, Bristol‐Myers Squibb, Lilly, Novartis, Sanofi‐Genzyme; he receives research support from Biogen Idec, Merck‐Serono, Novartis, Roche, the Italian Ministry of Health, the Italian Ministry of University and Research, and Fondazione Italiana Sclerosi Multipla.

## OPR‐047

### The sphingosine‐1‐phosphate mimetic FTY720 inhibits mitochondrial activity in lymphocytes from healthy donors

#### I. Gómez‐Delgado ^1^; A. González‐Jiménez1^1^; P. López‐Cotarelo^1^; A. Moreno‐Jerez^1^; J. Vela‐Artiza^1^; E. Mena‐Plaza^1^; Y. Aladro^2^; B. Pilo^2^; C. Oreja‐Guevara^3^; I. Gómez‐Estévez^3^; A. R. López‐Pastor^1^; E. Urcelay^1^

##### ^*1*^ *Laboratory of Genetics and Molecular Bases of Complex Diseases, Health Research Institute of Hospital Clínico San Carlos (IdISSC), Madrid, Spain;* ^*2*^ *Department of Neurology, Hospital Universitario de Getafe, Getafe, Spain;* ^*3*^ *Department of Neurology, Hospital Clínico San Carlos, Health Research Institute of Hospital Clínico San Carlos (IdISSC), Madrid, Spain*

**Background and Aims:** A non‐selective agonist of sphingosine‐1‐phosphate (S1P) receptors, FTY720, inhibits lymphocyte migration and mitigates subsequent inflammation. Mounting evidence supports the participation of S1P signaling in additional processes besides cell migration, but FTY720 mechanisms are not fully understood at present and possible effects on mitochondrial integrity are now explored. This study aims to investigate whether the S1P analog FTY720 modulates mitochondrial homeostasis in lymphocytes from healthy donors. Specifically, we evaluated its in vitro effects on peripheral blood mononuclear cells (PBMCs) isolated from controls.

**Methods:** Mitochondrial respiration and glycolytic activity were assessed by Seahorse XFp extracellular flux analysis. Mitochondrial mass, membrane potential, reactive oxygen species (ROS) production, and glycolytic mediators were evaluated by flow cytometry, and electron transport chain (ETC) complexes by Western Blot.

**Results:** FTY720 blocked early and late activation of PBMCs and inhibited basal and PHA‐stimulated respiration (basal, maximal, and ATP‐linked respiration) and glycolytic capacities (basal/maximal/glycolytic reserve). We observed significantly decreased mitochondrial depolarization and coupling in basal and stimulated conditions, with parallel increased ROS levels, together with lower fold‐change of complex III of the ETC upon PHA stimulation. Both in basal and PHA‐stimulated conditions, we found consistently reduced levels of fission/fusion (DRP1, FIS1, OPA1, MFN2) proteins and a reduced fold‐change in mitochondrial mass of B cells. Upon PHA stimulation, lactate transporters (MCT1 and 4) remained at unstimulated levels, while PFKB3 showed increased prestimulated levels already in the absence of PHA.

**Figure:**
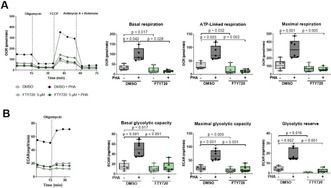
**FIGURE 1** Respirometry of control lymphocytes incubated with FTY720. (A) Oxygen consumption rate and (B) extracellular acidification rate profiles and associated parameters in unstimulated and PHA‐stimulated PBMCs in the presence of 5μM FTY720 or vehicle.

**Figure:**
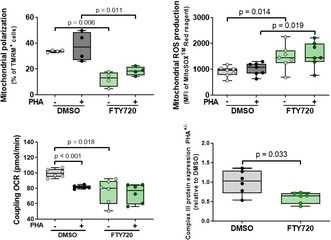
**FIGURE 2** Changes in mitochondrial polarization, coupling, ROS and complex III levels upon in vitro PHA‐stimulation of control lymphocytes incubated with FTY720.

**Figure:**
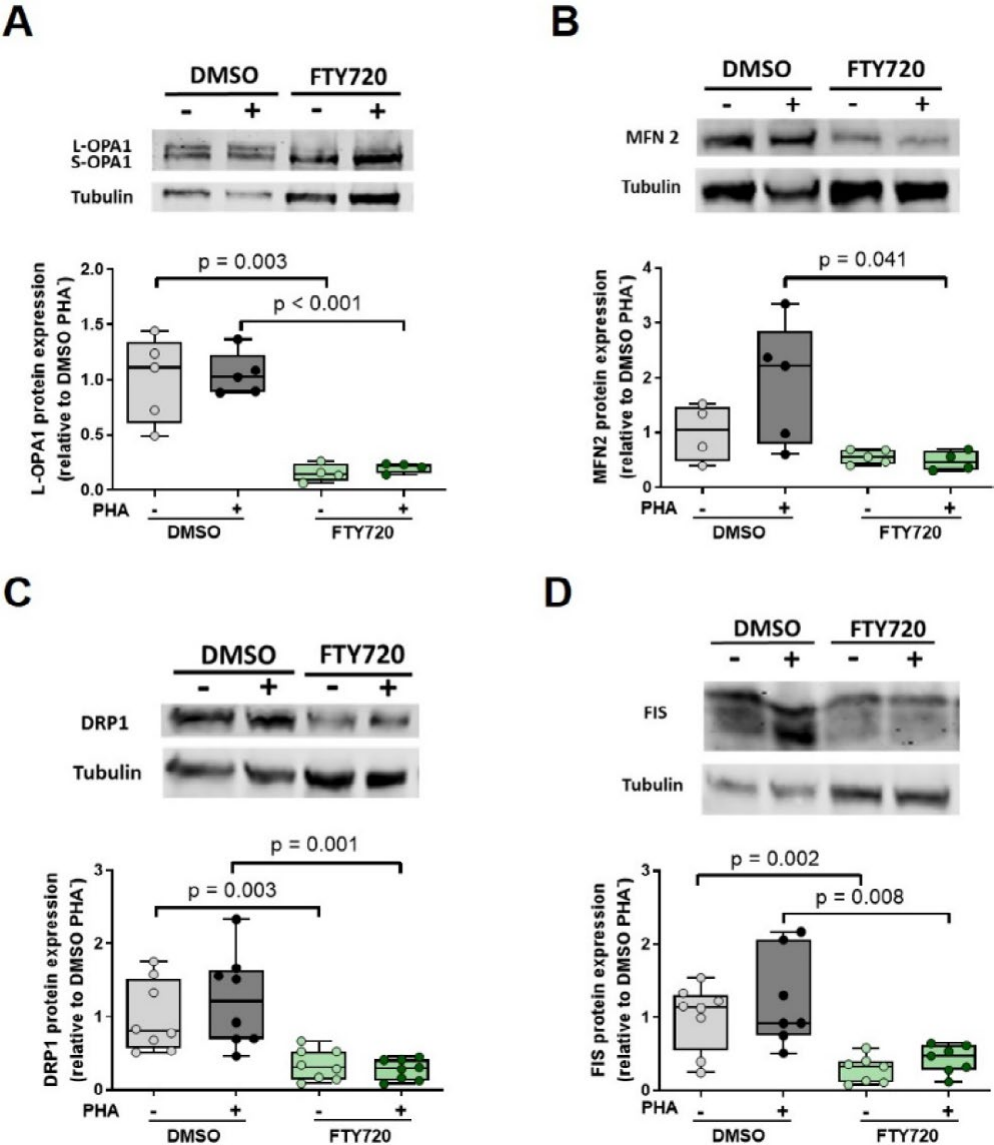
**FIGURE 3** Changes in mitochondrial fusion and fission protein levels of lymphocytes incubated with FTY720. Representative Western blots and quantification of (A) OPA‐L, (B) MFN2, (C) DRP1, and (D) FIS1 proteins from PBMCs.

**Conclusion:** FTY720 modulates mitochondrial activity of PBMCs isolated from healthy donors, indicative of additional pathways contributing to its therapeutic effects.

**Disclosure:** C.O.G. has received speaking and/or consultancy fees from Alexion, Biogen Idec, Bristol‐Myers‐Squibb, Janssen, Merck, Novartis, Roche, Sanofi‐Genzyme, and Teva. Y.A. has received research grants, travel support, and lecturing and consulting fees from Bayer, Biogen, Roche, Merck, Novartis, Almirall, Sanofi‐Genzyme, Janssen, and Bristol Myers Squibb. B.P. has received speaking, travel and/or training fees from Novartis, Almirall, Merck and Sanofi‐Genzyme. The other authors have nothing to disclose.

## OPR‐048

### Mitochondrial DNA copy number correlates with brain atrophy in multiple sclerosis

#### M. Granig ^1^; M. Martinez‐Serrat^1^; C. Tafrali^1^; I. Ivic^2^; M. Rados^2^; R. Demjaha^1^; M. Haindl^3^; B. Helmlinger^4^; E. Hofer^5^; B. Heschl^3^; A. Damulina^1^; E. Platos^3^; K. Liutkevych^3^; D. Pinter^4^; S. Ropele^3^; C. Enzinger^3^; C. Windpassinger^6^; J. Fessler^2^; M. Khalil^1^

##### ^*1*^ *Neurology Biomarker Research Unit, Department of Neurology, Medical University of Graz, Graz, Austria;* ^*2*^ *Division of Immunology, Medical University of Graz, Graz, Austria;* ^*3*^ *Department of Neurology, Medical University of Graz, Graz, Austria;* ^*4*^ *Research Unit for Neuronal Plasticity and Repair, Department of Neurology, Medical University of Graz, Graz, Austria;* ^*5*^ *Institute for Medical Informatics, Statistics and Documentation, Medical University of Graz, Graz, Austria;* ^*6*^ *Diagnostic and Research Institute of Human Genetics, Medical University of Graz, Graz, Austria*

**Background and Aims:** Multiple sclerosis (MS) is a chronic inflammatory autoimmune disease of the central nervous system characterized by demyelination and neurodegeneration. Mitochondrial DNA copy number (mtDNA‐CN) is a proposed biomarker of mitochondrial health, with reduced mtDNA‐CN being related to disease duration and worsening in MS. However, its temporal dynamics and association with brain atrophy remain unclear. This study investigates longitudinal changes in mtDNA‐CN in peripheral blood and its relationship with brain volume loss in people with MS (pwMS).

**Methods:** This study included 123 pwMS (mean (±SD) age 33.2 ± 8.5 years, 59.3% female, median disease duration 3.1 years [IQR: 1.0–7.9]), median Expanded Disability Status Scale (EDSS) score 1.0 [IQR: 0.0–2.0] and 47 healthy controls (HC; mean (±SD) age 34.1 ± 10.7 years, 72.3% female). In a subgroup of 62 pwMS, a second sample was collected after 2.6 ± 1.6 years. mtDNA‐CN was quantified by real‐time quantitative polymerase chain reaction in peripheral blood. Magnetic resonance imaging was performed on a 3‐T Tim Trio system and longitudinal brain volume changes were assessed using SIENA.

**Results:** The mtDNA‐CN was significantly lower in MS compared to HCs (*p* < 0.05). mtDNA‐CN was unrelated to age, sex, disease duration and EDSS. No significant longitudinal change in mtDNA‐CN was observed in the subgroup analysis. Of note, lower mtDNA‐CN at baseline correlated with a higher percentage of annualized brain volume loss during the follow‐up period (*ρ* = 0.266, *p* < 0.05).

**Conclusion:** These findings confirm lower mtDNA‐CN in pwMS and suggest that mtDNA‐CN might serve as a biomarker for the rate of brain atrophy in pwMS.

**Disclosure:** Marietta Granig has nothing to disclose. Maria Martinez‐Serrat has received travel funding and spaker honoraria from Novartis. Cansu Tafrali has received travel funding and speaker honoraria from Merck and Novartis. Ines Ivic has nothing to disclose. Matea Rados has nothing to disclose. Rina Demjaha has received travel funding from Janssen, Novartis and Sanofi. Michaela Tanja Haindl has nothing to disclose. Birgit Helmlinger received speaker honoraria from Roche, Bristol‐Myers Squibb, and Sanofi, and travel funding from Janssen. Edith Hofer has nothing to disclose. Bettina Heschl has nothing to disclose. Anna Damulina has participated in meetings sponsored by, received speaker honoraria or travel funding from Sanofi‐Aventis, Novartis, and Janssen. Emilia Platos has received a sponsored preceptorship from Neuraxpharm. Kateryna Liutkevych has nothing to disclose. Daniela Pinter is a member of the advisory board for “Cognition and MS” for Novartis and has received speaking honoraria from Biogen, Novartis, MedAhead and Bristol‐Myers Squibb. Stefan Ropele has nothing to disclose. Christian Enzinger received funding for traveling and speaker honoraria from Biogen Idec, Bayer Schering Pharma, Merck Serono, Novartis, Genzyme and Teva Pharmaceutical Industries Ltd./sanofi‐aventis, Shire; received research support from Merck Serono, Biogen Idec, and Teva Pharmaceutical Industries Ltd./sanofi‐aventis; and serves on scientific advisory boards for Bayer Schering Pharma, Biogen Idec, Merck Serono, Novartis, Genzyme, Roche, and Teva Pharmaceutical Industries Ltd./sanofi‐Aventis. Christian Windpassinger has nothing to disclose. Johannes Fessler has nothing to disclose. Michael Khalil has received travel funding and speaker honoraria from Bayer, Biogen, Novartis, Merck, Sanofi and Teva and serves on scientific advisory boards for Biogen, Bristol‐Myers Squibb, Gilead, Merck, Neuraxpharm, Novartis, Alexion, Amgen and Roche. He received research grants from Biogen, Novartis and Teva.

## OPR‐049

### Correlation of real‐world digital biomarkers with clinical standards, findings from the MS‐DETECT study

#### P. Vermersch ^1^; M. Filippi^2^; C. Oreja‐Guevara^3^; J. Oh^4^; T. Sejbæk^5^; J. Graves^6^; L. Klaeylé^7^; S. Bieuvelet^7^; L. Carment^7^; P. Drouin^7^; S. Zinaï^7^; P. Rufi^8^; B. Padrazzi^9^; T. Ziemssen^10^

##### ^*1*^ *Univ. Lille, UMR Inserm U1172, CHU Lille, Lille, France;* ^*2*^ *Neurology Unit, IRCCS San Raffaele Scientific Institute, Milan, Italy; Vita‐Salute San Raffaele University, Milan, Italy;* ^*3*^ *Vita‐Salute San Raffaele University, Milan, Italy;* ^*4*^ *Hospital Clinico San Carlos, IdISSC, Madrid, Spain;* ^*5*^ *Department of Neurology, Esbjerg Hospital, University Hospital of Southern Denmark, Esbjerg, Denmark;* ^*6*^ *Department of Neurosciences, University of California San Diego, San Diego, USA;* ^*7*^ *Ad Scientiam, Paris, France;* ^*8*^ *Sanofi, Vitry‐sur‐Seine, France;* ^*9*^ *Sanofi, Paris, France;* ^*10*^ *MS Center, Center of Clinical Neuroscience, Neurological Clinic, University Clinic Carl Gustav Carus, TU Dresden, Dresden, German*

**Background and Aims:** MSCopilot®, a Software as a Medical Device, remotely assesses key functions such as cognition, dexterity, vision and walking. French studies showed that MSCopilot® tests correlate with the Extended Disability Scale Score (EDSS) and the 4‐item MS Functional composite (MSFC‐4) when assessed in‐clinic. The international MS‐DETECT study (NCT05816122) evaluates MSCopilot®'s ability to detect disability worsening during 18–24 months. This analysis compares individual MSFC‐4 scores with MSCopilot® digital biomarkers measured in‐clinic or at home.

**Methods:** MSFC‐4 scores were assessed in‐clinic during the inclusion visit. MSCopilot® tests were performed with and without supervision in‐clinic and at home, respectively. Association between the digital scores and their clinical counterparts was assessed using Pearson correlation coefficients.

**Results:** 244 patients (68.4% female, mean age 49.15 ± 8.74, mean disease duration 17.57 ± 8.04 and mean EDSS: 3.8 ± 1.3) were included in this analysis. We collected 973 in‐clinic and 870 remote MSCopilot® evaluations (243/218 cognition, 244/219 dexterity, 244/215 vision and 242/218 walking). Significant correlations were found between individual MSFC‐4 items and digital tests: cognition (*r* = 0.71/0.62), dexterity (0.45/0.47), vision (0.36/0.37) and walking (0.54/0.66) tests. Composite MSFC‐4 scores significantly correlated with MSCopilot®'s measured in‐clinic (0.71) and remotely (0.74).

**Conclusion:** MSCopilot® digital tests, whether performed in‐clinic or at home, correlated with their clinical counterparts. These findings emphasize their potential use in detecting disease worsening thus leading to improved clinical monitoring of MS.

**Disclosure:** P. Vermersch: AB Science, Ad Scientiam, Biogen, Imcyse, Janssen, Merck, Novartis, Roche, Sanofi, and Teva—consulting fees. Novartis, Roche, and Sanofi—research support. M. Filippi: The Journal of Neurology—Editor‐in‐Chief; Human Brain Mapping, Neurological Sciences, and Radiology—Associate editor; Alexion, AstraZeneca Rare Disease, Almirall, Biogen, Merck, Novartis, Roche, Sanofi—consulting fees. Bayer, Biogen, Celgene, Chiesi Italia SpA, Eli Lilly, Genzyme, Janssen, Merck‐Serono, Neopharmed Gentili, Novartis, Novo Nordisk, Roche, Sanofi, Takeda, and TEVA—speaking fees; Alexion, AstraZeneca Rare Disease, Biogen, Bristol‐Myers Squibb, Merck, Novartis, Roche, Sanofi, Sanofi‐Aventis, Sanofi‐Genzyme, Takeda; scientific direction of educational events for Biogen, Merck, Roche, Celgene, Bristol‐Myers Squibb, Lilly, Novartis, Sanofi—advisory board member; Biogen Idec, Merck‐Serono, Novartis, Roche, the Italian Ministry of Health, the Italian Ministry of University and Research, and Fondazione Italiana Sclerosi Multipla—research support. C. Oreja‐Guevara: Alexion, AstraZeneca rare Disease, Amgen, Biogen Idec, BMS, Horizon, Janssen, Merck, Novartis, Roche, Sanofi‐Genzyme, Sandoz, Viatris, Neuraxpharm and Teva—speaking and consulting fees; advisory board member. J. Oh: Amgen, Biogen, Eli Lilly and Company, EMD Serono, Novartis, Roche, Sanofi—consulting and/or speaking fees; Biogen, Roche—research support. T. Sejbæk: Tobias Sejbæk received travel grants from Biogen, Merck, Novartis, Roche and Sanofi—travel grants; Biogen, Merck and Sanofi—research grants; Biogen, Merck, Neuroxpharm, Novartis, Roche and Sanofi—advisory board member. JS. Graves: Sanofi, Genentech, Ad Scientium, Octave and EMD Serono—research grants; Octave, TR1X and Google—consulting fees. L. Klaeylé, S. Bieuvelet, L. Carment, P. Drouin, S. Zinaï: Ad Scientiam employees; may hold shares or stock options. P. Rufi, B. Padrazzi: Sanofi employees; may hold shares or stock options. T. Ziemssen: Biogen, Roche, Neuraxpharm, Novartis, Viatris and Merck—scientific advisory board and/or consulting fees; Neuraxpharm, Roche, Novartis, Merck, Sanofi, BMS, and Biogen—speaking fees; Neuraxpharm, Roche, Novartis, Merck, and Sanofi—research support.

## Ageing and Dementia 1

## OPR‐050

### Cortical thickness signature of early‐onset Alzheimer's disease across preclinical and prodromal stages in a real‐world memory clinic

#### A. Zilioli ^1^; R. Mohanty^2^; A. Rosenberg^2^; A. Matton^2^; T. Granberg^2^; G. Hagman^2^; M. Spallazzi^1^; M. Kivipelto^2^; E. Westman^2^

##### ^*1*^ *Department of Neurology, University‐Hospital of Parma, Parma, Italy;* ^*2*^ *Division of Clinical Geriatrics, Center for Alzheimer Research, Department of Neurobiology, Care Sciences and Society, Karolinska Institutet, Stockholm, Sweden*

**Background and Aims:** The cortical thinning signature of early‐onset Alzheimer's disease (EOAD) is well characterized in dementia, but its expression at earlier stages remains unclear. We aimed to determine when EOAD‐related cortical atrophy becomes detectable and assess ATN biomarker contribution.

**Methods:** We studied individuals <65 years who underwent 3T structural MRI and cerebrospinal fluid–based ATN profiling. Cortical thickness was derived from T1‐weighted images using FreeSurfer. EOAD signature regions were defined through vertex‐wise comparisons between AD dementia and ATN‐negative subjective cognitive decline (SCD). Regional cortical thickness and hippocampal volume were evaluated across SCD and mild cognitive impairment (MCI) groups stratified by ATN status. Brain–cognition associations were assessed.

**Results:** The cohort included 507 individuals (mean age 57.7 ± 5.3 years), of whom 185 within the AD continuum. EOAD dementia showed marked thinning in parietal and lateral temporal cortices. In MCI with AD biomarkers, cortical thickness was significantly reduced across EOAD signature regions, including the inferior and superior parietal lobules, precuneus, and lateral temporal cortex, together with hippocampal volume (all *p* < 0.01; Cohen's *d* ≤ −0.50). In SCD, cortical thinning was observed only in A+T+ individuals, involving the precuneus and the banks of the superior temporal sulcus (all *p* < 0.05; *d* ≤ −0.60), with no differences in A+T− SCD. In MCI‐AD, reduced cortical thickness correlated with poorer global cognition and episodic and visuospatial memory (all *p* < 0.05), while no associations emerged in SCD.

**Figure:**
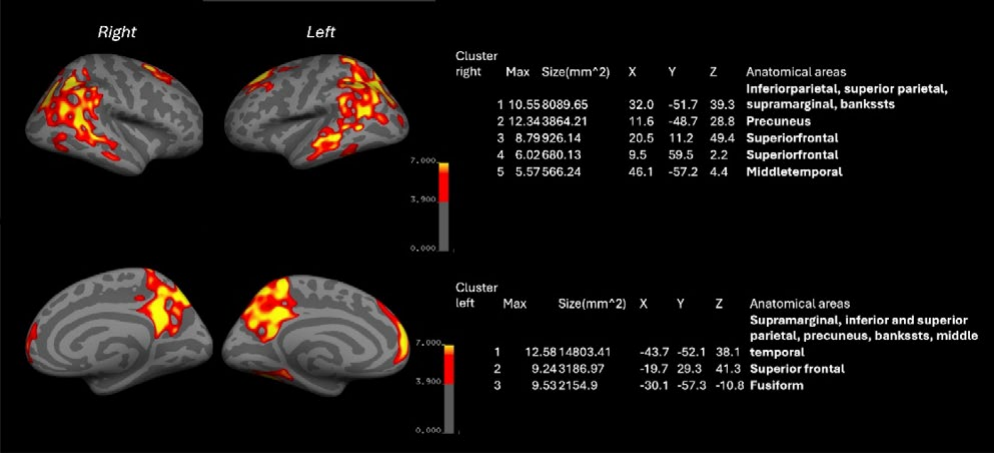
**FIGURE 1** Cortical thinning pattern in EOAD dementia compared to the reference group of ATN‐negative SCD.

**Figure:**
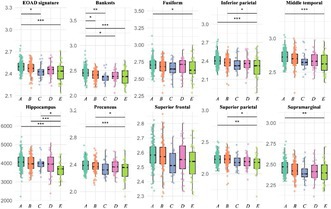
**FIGURE 2** Regional cortical thickness and hippocampal volume in MCI subgroups. (A) SCD ATN negative, (B) MCI ATN negative, (C) MCI non AD‐change, (D) MCI A+T−, (E) MCI A+T+.

**Figure:**
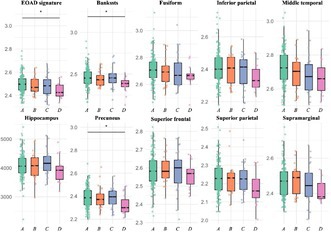
**FIGURE 3** Regional cortical thickness and hippocampal volume in SCD groups. (A) SCD ATN negative, (B) SCD non AD change, (C) SCD A+T−, (D) SCD A+T+.

**Conclusion:** EOAD‐related cortical thinning at preclinical and prodromal stages depends on combined amyloid and tau pathology.

**Disclosure:** Nothing to disclose.

## OPR‐051

### Automatic primary progressive aphasia variant classification from transcripts and diffusion magnetic resonance imaging

#### S. Pisano ^1^; S. Basaia^1^; L. Lumaca^1^; A. Riva^1^; E. Canu^2^; S. Musicco^1^; B. Zorniotti^1^; A. Corini^1^; V. Castelnovo^2^; E. Spinelli^3^; G. Cecchetti^4^; A. Ghirelli^3^; G. Rugarli^3^; F. Caso^5^; G. Magnani^5^; P. Caroppo^6^; S. Prioni^6^; C. Villa^6^; M. Filippi^7^; F. Agosta^3^

##### ^*1*^ *Neuroimaging Research Unit, Division of Neuroscience, IRCCS San Raffaele Scientific Institute, Milan, Italy;* ^*2*^ *Neuroimaging Research Unit, Division of Neuroscience, and Neurology Unit, IRCCS San Raffaele Scientific Institute, Milan, Italy;* ^*3*^ *Neuroimaging Research Unit, Division of Neuroscience, and Neurology Unit, IRCCS San Raffaele Scientific Institute, Milan, Italy; and Vita‐Salute San Raffaele University, Milan, Italy;* ^*4*^ *Neuroimaging Research Unit, Division of Neuroscience, Neurology Unit, and Neurophysiology Service, IRCCS San Raffaele Scientific Institute, Milan, Italy;* ^*5*^ *Neurology Unit, IRCCS San Raffaele Scientific Institute, Milan, Italy;* ^*6*^ *Unit of Neurology 5, Clinical Neuropathology Unit, Fondazione IRCCS Istituto Neurologico Carlo Besta, Milan, Italy;* ^*7*^ *Neuroimaging Research Unit, Division of Neuroscience, Neurology Unit, Neurorehabilitation Unit, and Neurophysiology Service, IRCCS San Raffaele Scientific Institute, Milan, Italy; and Vita‐Salute San Raffaele University, Milan, Italy*

**Background and Aims:** Primary Progressive Aphasia (PPA) comprises three main clinical variants, nonfluent/agrammatic (nfvPPA), semantic (svPPA), and logopenic (lvPPA), each with distinct linguistic profiles and neurodegeneration patterns. Differential diagnosis relies heavily on subjective clinical judgment, limiting standardization. This study developed a multimodal pipeline combining speech‐derived linguistic features with diffusion MRI markers for automated PPA classification.

**Methods:** This study included data from 91 healthy controls and 94 PPA patients (38 nfvPPA, 36 svPPA and 20 lvPPA). Connected speech was recorded during Picnic scene description task and automatically transcribed using Microsoft Azure after preprocessing optimization. Linguistic features were extracted via Python pipelines; diffusion features derived from atlas‐based fractional anisotropy measures. A multinomial logistic regression classifier was trained with nested cross‐validation. Transcriptions were compared against manual gold‐standard using word error rate (WER). A web‐based application integrating transcription with clinician‐supervised revision and automatic feature extraction was implemented.

**Results:** Automatic transcription achieved mean WER of 12 ± 7% (controls) and 19 ± 10% (PPA). Linguistic features captured variant‐specific patterns (reduced output and slowed speech in nfvPPA; increased pauses and word‐finding difficulty in lvPPA; fluent but semantically less informative speech in svPPA). The multimodal classifier achieved balanced accuracy of 87 ± 6.9%, outperforming speech‐only and imaging‐only models. Per‐variant accuracy was 93% (nfvPPA), 95% (svPPA), with lower performance for lvPPA.

**Conclusion:** This multimodal framework integrates speech and diffusion MRI to support standardized PPA variant differentiation with strong diagnostic performance. The interactive web platform improves transcription accuracy throught clinician oversight, supporting harmonized diagnosis and longitudinal monitoring.

**Disclosure:** Supported by European Research Council (StG‐2016_714388_NeuroTRACK). Stefano Pisano, Laura Lumaca, Annamaria Riva, Simona Musicco, Beatrice Zorniotti, Alice Corini, Veronica Castelnovo, Edoardo G. Spinelli, Alma Ghirelli, Giulia Rugarli, Francesca Caso, Giuseppe Magnani, Paola Caroppo, Sara Prioni, and Cristina Villa have nothing to disclose; S Basaia receives research support from the Italian Ministry of Health; Elisa Canu receives research support from the Italian Ministry of Health; Giordano Cecchetti received speaker honoraria from Neopharmed Gentili; M. Filippi is Editor‐in‐Chief of the Journal of Neurology, Associate Editor of Human Brain Mapping, Neurological Sciences, and Radiology; received compensation for consulting services from Almirall, Biogen, Bristol‐Myers Squibb, Eli Lilly, Merck, Novartis, Roche, Sanofi; speaking activities from Amgen, Bayer, Biogen, Bristol‐Myers Squibb, Celgene, Chiesi Italia SpA, Eisai, Eli Lilly, Fujirebio, Genzyme, Janssen, Merck, Neopharmed Gentili, Neuraxpharm, Novartis, Novo Nordisk, Roche, Sanofi, Takeda; participation in Advisory Boards for Alexion, Biogen, Bristol‐Myers Squibb, Eli Lilly, GE Healthcare Ltd, Merck, Neuraxpharm, Novartis, Roche, Sandoz, Sanofi, Takeda; scientific direction of educational events for Biogen, Merck, Roche, Celgene, Bristol‐Myers Squibb, Lilly, Novartis, Sanofi‐Genzyme; he receives research support from Biogen Idec, Merck‐Serono, Novartis, Roche, the Italian Ministry of Health, the Italian Ministry of University and Research, and Fondazione Italiana Sclerosi Multipla. F. Agosta is Associate Editor of NeuroImage: Clinical and the European Journal of Neurology; has received speaker honoraria from Biogen Idec, Bristol Myers Squibb, Eisai, Eli Lilly, GE Healthcare, Neuraxpharm, and Roche; and receives or has received research supports from the Italian Ministry of Health, the Italian Ministry of University and Research, AriSLA (Fondazione Italiana di Ricerca per la SLA), the European Research Council (ERC), the EU Joint Programme – Neurodegenerative Disease Research (JPND), and Foundation Research on Alzheimer Disease (France).

## OPR‐052

### Independent effects of amyloid‐β and tau pathology on synaptic damage across the Alzheimer's continuum

#### F. Roveta ^1^; A. Mendes^2^; V. Garibotto^3^; C. Boccalini^3^; A. Lathuiliere^4^; I. Rainero^1^; K. Blennow^5^; H. Zetterberg^5^; N. Ashton^6^; F. Ribaldi^2^; G. Frisoni^2^

##### ^*1*^ *Aging Brain and Memory Clinic, Department of Neuroscience “Rita Levi‐Montalcini”, University of Turin, Turin, Italy;* ^*2*^ *Geneva Memory Center, Department of Rehabilitation and Geriatrics, Geneva University Hospitals, Geneva, Switzerland;* ^*3*^ *Division of Nuclear Medicine and Molecular Imaging, Diagnostic Department, Geneva University Hospitals, Geneva, Switzerland;* ^*4*^ *Laboratory for Translational Research in Neurodegeneration, University of Geneva, Geneva, Switzerland;* ^*5*^ *Department of Psychiatry and Neurochemistry, Institute of Neuroscience & Physiology, The Sahlgrenska Academy at the University of Gothenburg, Mölndal, Sweden;* ^*6*^ *Banner Alzheimer's Institute and University of Arizona, Phoenix, USA*

**Background and Aims:** Synaptic dysfunction is the biological substrate of cognitive decline in Alzheimer's disease (AD). While amyloid‐β and tau are known to interact in driving synaptic damage, their independent contributions remain unclear.

**Methods:** 109 cognitively unimpaired individuals an d patients with mild cognitive impairment or dementia from the Geneva Memory Center with available CSF synaptic biomarkers (neurogranin, SNAP25, GAP43) were included and classified according to amyloid (A) and tau (T) status. Group comparisons and correlations between synaptic and AD biomarkers were performed. Multiple linear regression models assessed the independent associations of amyloid and tau with synaptic biomarkers. Model I included CSF Aβ42 and CSF p‐tau181, whereas Model II included amyloid PET centiloid and CSF p‐tau181. Mediation analyses explored the relationships between amyloid, tau and synaptic biomarkers. All analyses were replicated in the Alzheimer's Disease Neuroimaging Initiative (ADNI) cohort (*n* = 787).

**Results:** Synaptic biomarkers concentrations were significantly higher in A+/T+ participants (*n* = 27) compared with A−/T− (*n* = 52) and A+/T− (*n* = 30) groups (*p* < 0.001). CSF p‐tau181 showed the strongest correlations with synaptic biomarkers (rs up to 0.83, *p* < 0.001). In Model I, GAP43 was positively associated with CSF Aβ42 (β = 0.31, 95% CI 0.23–0.40, *p* < 0.001; partial *R* ^2^ = 0.321) and p‐tau181 (β = 0.94, 95% CI 0.84–1.04, *p* < 0.001; partial *R* ^2^ = 0.809). In Model II, lower centiloid (β = −0.31, 95% CI −0.46 to −0.16, *p* < 0.001; partial *R* ^2^ = 0.225) and higher p‐tau181 (β = 0.94, 95% CI 0.84–1.04, *p* < 0.001; partial *R* ^2^ = 0.758) predicted higher GAP43. Mediation analyses indicated tau as the mediator of the direct association between amyloid burden and GAP43.

**Conclusion:** These results indicate tau pathology as the main driver of synaptic damage across the Alzheimer's disease continuum.

**Disclosure:** Nothing to disclose.

## OPR‐053

### Residual cerebrospinal fluid NPTX2 predicts dementia progression beyond neurogranin and core pathology in prodromal Alzheimer's disease

#### F. Massa ^1^; F. De Cesari^1^; G. Bozzo^1^; A. Panza^1^; M. Urbinati^1^; V. Pelagotti^1^; A. Lechiara^2^; E. Mobilia^2^; F. Bozzano^2^; D. Visigalli^2^; L. Lorenzini^1^; M. Losa^1^; A. Brugnolo^1^; N. Girtler^1^; D. Arnaldi^1^; A. Uccelli^2^; G. Pesce^2^; M. Pardini^1^

##### ^*1*^ *Department of Neuroscience, Rehabilitation, Ophthalmology, Genetics, Maternal and Child Health (DINOGMI), University of Genoa, Genoa, Italy;* ^*2*^ *IRCCS Ospedale Policlinico San Martino, Genoa, Italy*

**Background and Aims:** In Alzheimer's disease (AD) core markers have limited ability in predicting progression from mild cognitive impairment (MCI) to dementia. Synaptic cerebrospinal fluid (CSF) biomarkers may add prognostic information, spanning processes from injury to adaptive network capacity. We tested whether a residual component of CSF neuronal pentraxin‐2 (NPTX2), a synaptic protein linked to network homeostasis, beyond neurogranin (Ng) reflecting synaptic injury and core AD biomarker burden predicts dementia progression.

**Methods:** We retrospectively studied 104 patients with typical MCI‐AD supported by core CSF biomarkers. CSF NPTX2 and Ng were quantified by commercial immunoassays. Early vs. late MCI sub‐stages were defined by proximity to progression (LMCI ≤2 years, EMCI > 2 years or stable). A residual NPTX2 component (Res_NPTX2) was defined after regressing standardized (*z*‐transformed) NPTX2 on Ng, a core AD biomarker burden composite (phosphorylated tau181 divided by amyloid‐β42/amyloid‐β40), and age. Associations with Mini‐Mental State Examination (MMSE) and time to progression were tested using linear regression, Kaplan–Meier and Cox analyses, including ≤2‐year administrative censoring.

**Results:** Ng correlated with NPTX2 (*ρ* = 0.67, *p* < 0.001). Res_NPTX2 was higher in early than late MCI (*p* = 0.0006) and associated with better MMSE at baseline (*p* = 0.008) and less decline over time (ΔMMSE, *p* = 0.002). During follow‐up, 55/104 (52.9%) progressed to dementia. Higher Res_NPTX2 predicted lower hazard of progression (HR = 0.63; *p* = 0.007). Res_NPTX2 tertiles separated Kaplan–Meier curves (log‐rank *p* = 0.006), with stronger discrimination within 2 years (High vs. Low HR = 0.18; *p* = 0.008).

**Figure:**
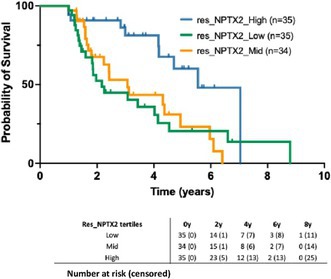
**FIGURE 1** Kaplan–Meier curves for time‐to‐progression to AD dementia across tertiles of Res_NPTX2.

**Conclusion:** A residual CSF NPTX2 component beyond Ng and core biomarker burden identifies clinically meaningful heterogeneity and improves near‐term prognostic stratification for dementia progression in MCI due to AD.

**Disclosure:** Nothing to disclose.

## OPR‐054

### Plasma TREM2 marks limbic disconnection in Alzheimer's disease

#### T. Merati ^1^; A. Pilotto^1^; A. Rondina^2^; A. Galli^2^; V. Bettonagli^2^; C. Martinuzzo^1^; C. Tolassi^3^; C. Trasciatti^2^; I. Girotto^3^; M. Bertoni^3^; A. Benedet^4^; G. Di Molfetta^4^; I. Pola^4^; K. Tan^4^; W. Traichel^4^; A. Toja^1^; S. Caratozzolo^1^; M. Blennow^5^; H. Zettergerg^5^; N. Ashton^6^; A. Padovani^1^

##### ^*1*^ *Department of Clinical and Experimental Sciences, Neurology Unit, University of Brescia, Brescia, Italy;* ^*2*^ *Department of Continuity of Care and Frailty, Neurology Unit, ASST Spedali Civili Brescia University Hospital, Brescia, Italy;* ^*3*^ *Neurobiorepository and Laboratory of advanced biological markers, University of Brescia and ASST Spedali Civili Hospital, Brescia, Italy;* ^*4*^ *Department of Psychiatry and Neurochemistry, Institute of Neuroscience & Physiology, the Sahlgrenska Academy at the University of Gothenburg, Mölndal, Sweden;* ^*5*^ *Clinical Neurochemistry Laboratory, Sahlgrenska University Hospital, Mölndal, Sweden;* ^*6*^ *Banner Alzheimer's Institute and University of Arizona, Phoenix, USA*

**Background and Aims:** TREM2 (Triggering Receptor Expressed on Myeloid Cells 2) modulates microglial responses to neurodegeneration and plasma soluble TREM2 has been linked to white matter hyperintensity burden. However, whether plasma TREM2 relates to tract‐specific white matter disconnection in Alzheimer's disease (AD) is unknown. To determine whether plasma TREM2 is associated with white matter tract disconnection in AD and whether such associations show anatomical specificity.

**Methods:** Fifty‐seven AD patients (age 72 ± 6 years, 42% male) underwent 3T MRI and plasma collection. TREM2 was measured using the NULISA CNS Disease Panel. White matter hyperintensities were segmented on FLAIR and MPRAGE images and tract‐specific disconnection probability was estimated for white matter tracts using volBrain DeepLesion, an automated deep learning‐based pipeline. Tracts were grouped into limbic, association, callosal and projection categories. Partial correlations adjusted for age and sex tested associations between plasma TREM2 and each outcome.

**Results:** Plasma TREM2 showed a trend association with total WMH volume (*r* = 0.28, *p* = 0.07). Strikingly, TREM2 was selectively associated with limbic tract disconnection: uncinate fasciculus (*r* = 0.46, *p* = 0.002), rostral cingulum (*r* = 0.40, *p* = 0.007), frontal‐parahippocampal cingulum (*r* = 0.34, *p* = 0.024), and parahippocampal‐parietal cingulum (*r* = 0.32, *p* = 0.040). Overall, 4/12 limbic tracts reached significance (mean *r* = +0.38), compared to 0/4 callosal and 0/6 projection tracts (Figure 2). Higher TREM2 consistently predicted greater disconnection in memory‐critical pathways (Figure 1).

**Figure:**
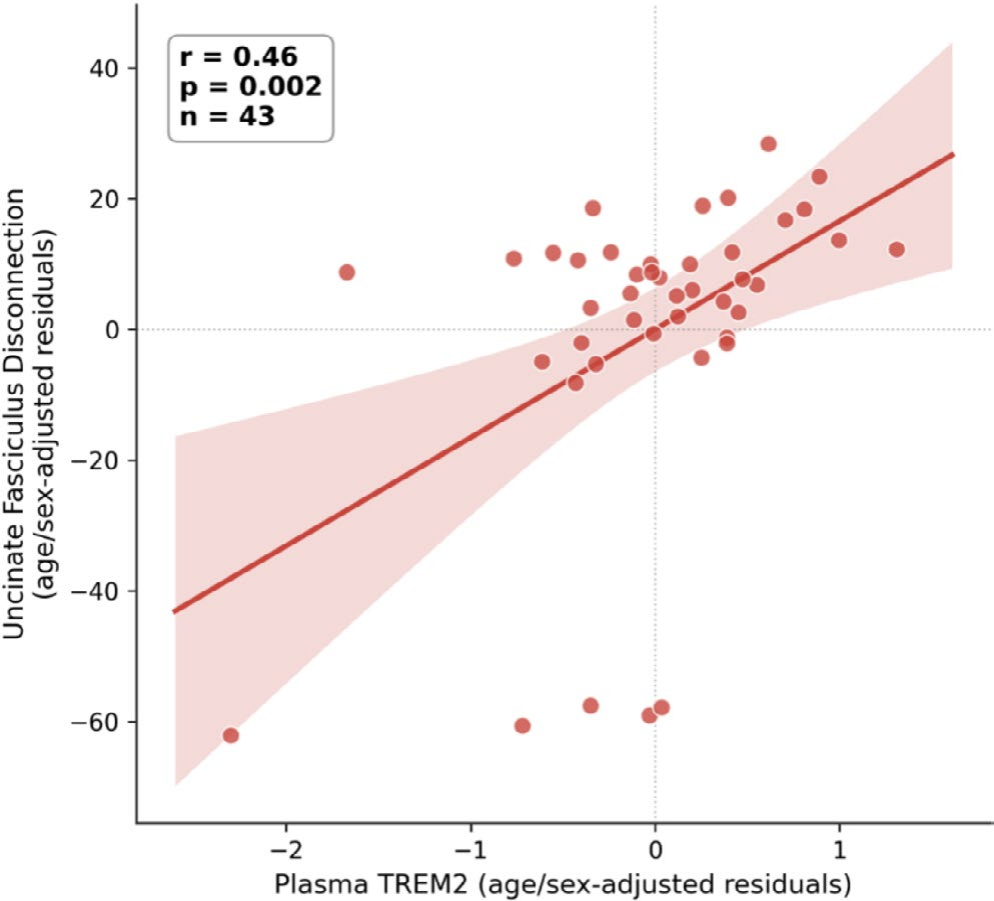
**FIGURE 1** Plasma TREM2 and uncinate fasciculus disconnection in Alzheimer's disease. Scatter plot shows age‐ and sex‐adjusted residuals.

**Figure:**
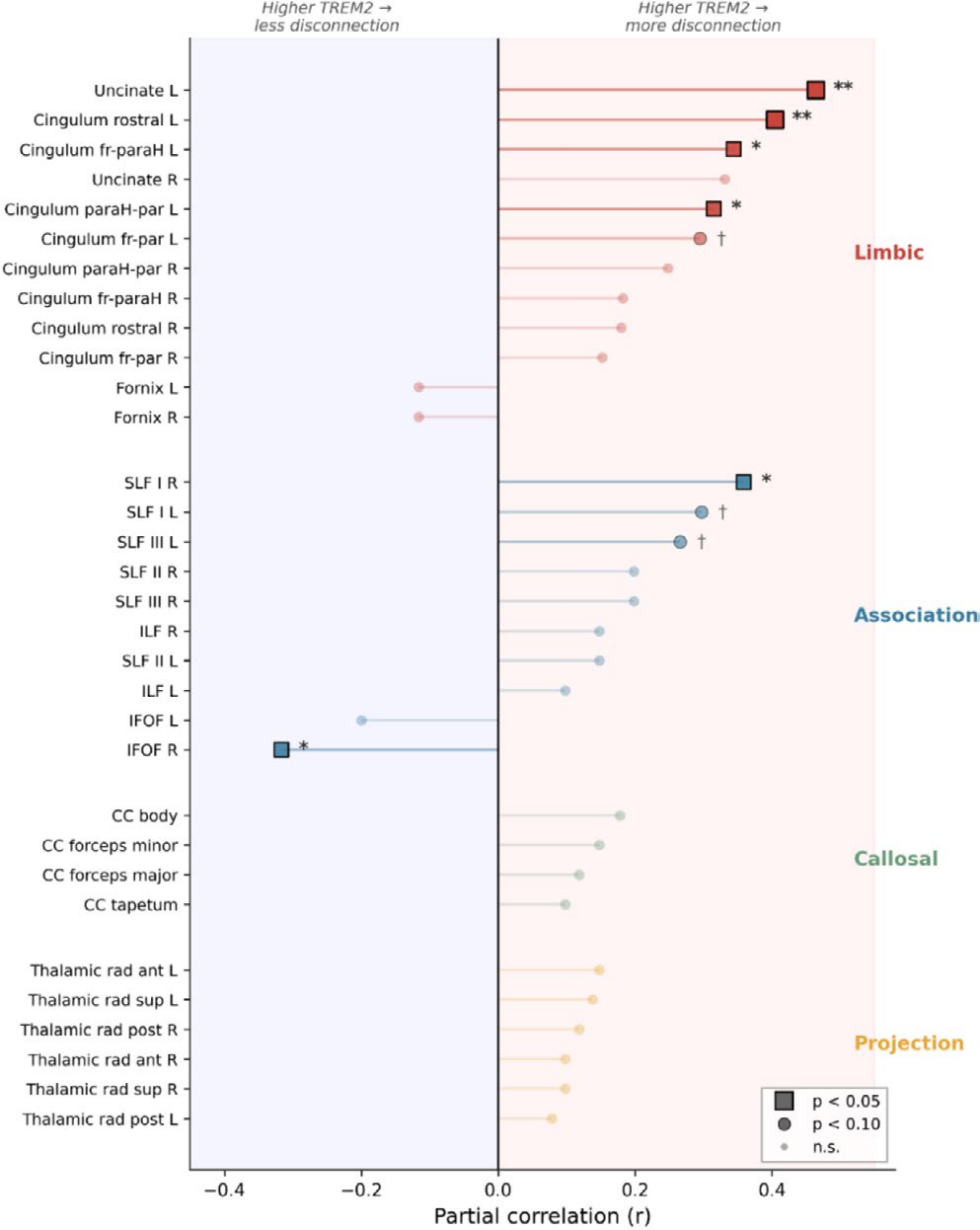
**FIGURE 2** Forest plot of TREM2 associations with white matter tract disconnection.

**Conclusion:** Plasma TREM2 is specifically associated with limbic white matter disconnection in AD. This anatomical selectivity mirrors the regional vulnerability of AD and implicates microglial activation in disrupting memory‐relevant circuits. Plasma TREM2 may serve as an accessible biomarker of limbic network integrity in AD.

**Disclosure:** Nothing to disclose.

## Peripheral Nerve Disorders

## OPR‐055

### Serum inflammatory proteomic signatures stratify disease activity in chronic inflammatory demyelinating polyneuropathy

#### A. Bhandage^1^; F. Stascheit^2^; H. Preßler^2^; S. Hoffmann^2^; A. Meisel^3^; A. Rostedt Punga ^4^

##### ^*1*^ *Department of Medical Sciences, Clinical Neurophysiology, Uppsala University, Uppsala, Sweden;* ^*2*^ *Department of Neurology with Experimental Neurology and Neuroscience Clinical Research Center, Charité‐Universitätsmedizin Berlin, corporate member of Freie Universität Berlin and Humboldt Universität zu Berlin, Berlin, Germany;* ^*3*^ *Department of Neurology with Experimental Neurology and Center for Stroke Research Berlin, Charité‐Universitätsmedizin Berlin, corporate member of Freie Universität Berlin and Humboldt Universität zu Berlin, Berlin, Germany;* ^*4*^ *Department of Medical Sciences, Clinical Neurophysiology, Uppsala University and Clinical Neurophysiology, Uppsala University Hospital, Uppsala, Sweden*

**Background and Aims:** Chronic inflammatory demyelinating polyneuropathy (CIDP) is an immune‐mediated neuropathy characterized by marked clinical heterogeneity and variable treatment response. Objective blood‐based biomarkers reflecting disease activity and therapeutic responsiveness remain an unmet need, particularly in the context of immunoglobulin therapy, which may confound immune profiling. We aimed to identify serum inflammatory proteomic signatures associated with clinical phenotype, disease activity, and treatment exposure in CIDP.

**Methods:** In this case–control study, a multiplex proximity extension assay (PEA) and ELISA were used to quantify inflammatory proteins in serum from patients with CIDP and age‐ and sex‐matched healthy controls, as well as from neurological disease controls including multiple sclerosis and IVIG‐treated myasthenia gravis. Multivariable regression with correction for multiple testing, principal component analysis, and correlation analyses were performed to define disease‐specific protein signatures and their associations with clinical characteristics, disease stability, and longitudinal outcomes.

**Results:** Distinct inflammatory proteomic patterns differentiated 51 patients with CIDP from 192 healthy controls and 105 neurological disease controls, indicating immune signatures beyond treatment‐related effects. Specific protein clusters were associated with clinical phenotype, unstable disease, and subsequent clinical improvement, whereas others reflected immunoglobulin‐modifiable pathways. Integrative analyses further suggested links between systemic inflammatory activity and markers of axonal injury.

**Conclusion:** This study identifies clinically relevant serum inflammatory signatures in CIDP and supports their potential as translational biomarkers for patient stratification, disease monitoring and therapy optimization.

**Disclosure:** F.S., A.R.P., S.H., and A.M. report speaker fees, consultancy honoraria, advisory board participation, or institutional research support from companies active in neuromuscular therapeutics, including Alexion, argenx, UCB, and others. These relationships are unrelated to the present study. All other authors declare no competing interests.

## OPR‐056

### Patient‐reported carpal tunnel syndrome outcomes with and without surgery during COVID‐19: A real‐world follow‐up study

#### G. Rusin ^1,2^; A. Fryźlewicz^1^; J. Antczak^1,2^

##### ^*1*^ *Department of Neurology, University Hospital in Cracow, Poland;* ^*2*^ *Department of Neurology, Jagiellonian University Medical College, Cracow, Poland*

**Background and Aims:** During the COVID‐19 period, some patients postponed or declined planned surgery for carpal tunnel syndrome (CTS). We assessed whether surgery was associated with better longitudinal outcomes on symptom severity and functional status.

**Methods:** We analyzed follow‐up data from 73 patients (17.8% men, mean age 59.5 ± 12.0) first assessed pre‐COVID and re‐contacted later. Surgery status was self‐reported (operated *n* = 29; non‐operated *n* = 44). Overall, 40 (54.8%) used ≥1 additional option (rehabilitation, nocturnal splinting, reduced manual work). Baseline Padua severity was similar: median 3 (IQR 3–3) in both groups. Primary outcomes were Boston Carpal Tunnel Questionnaire subscales: Symptom Severity Scale (SSS) and Functional Status Scale (FSS). Between‐group differences in improvement were tested, and multivariable linear models evaluated follow‐up SSS/FSS adjusted for baseline score, follow‐up interval, age, and Padua score.

**Results:** Both groups improved significantly over time. SSS improved from 2.77 ± 0.78 to 1.64 ± 0.80 in non‐operated and from 2.94 ± 0.83 to 1.58 ± 0.65 in operated patients (both *p* < 0.001). FSS improved from 2.47 ± 0.95 to 1.70 ± 0.81 in non‐operated and from 2.58 ± 0.95 to 1.65 ± 0.75 in operated patients (both *p* < 0.001). Mean SSS improvement was 1.13 ± 1.12 vs. 1.36 ± 1.17 (*p* = 0.40; Figure 1). Mean FSS improvement was 0.77 ± 1.26 vs. 0.93 ± 1.11 (*p* = 0.562; Figure 2). In adjusted models, surgery was not independently associated with follow‐up SSS (β = −0.10, 95% CI −0.49 to 0.28; *p* = 0.60) or FSS (β = −0.04, 95% CI −0.45 to 0.36; *p* = 0.83).

**Figure:**
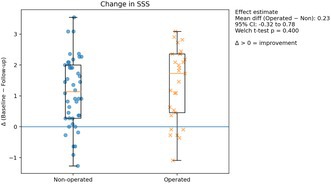
**FIGURE 1** Change in symptom severity scale from baseline to follow‐up by surgery status.

**Figure:**
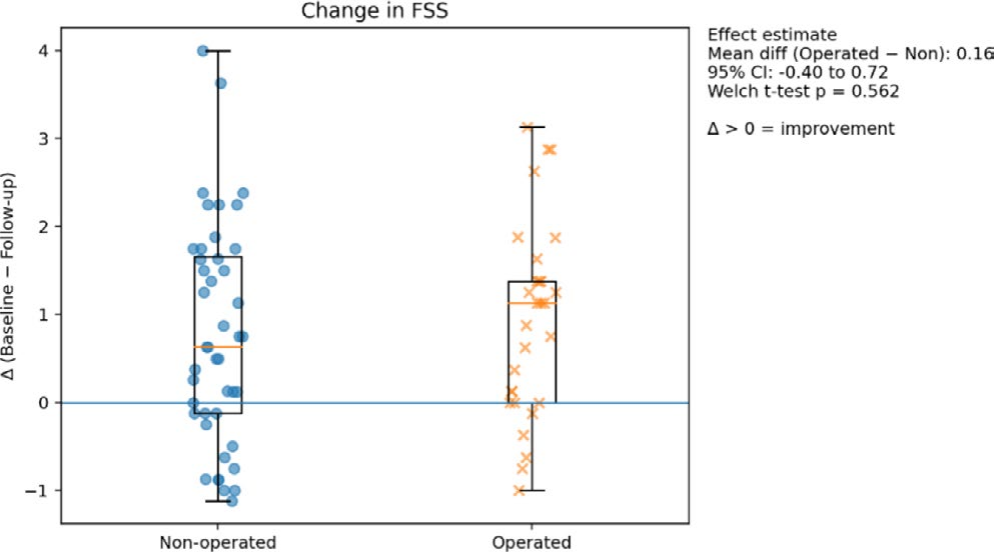
**FIGURE 2** Change in functional status scale from baseline to follow‐up by surgery status.

**Conclusion:** In this cohort, symptom and function scores improved over time regardless of surgery status. Surgery was not associated with greater longitudinal improvement in SSS or FSS after adjustment, suggesting that non‐surgical factors and follow‐up timing may substantially influence patient‐reported outcomes.

**Disclosure:** Nothing to disclose.

## OPR‐057

### Diaphragmatic dysfunction after lung transplantation: Longitudinal assessments and intraoperative neurophysiological monitoring of the phrenic nerve

#### G. Libelli ^1^; M. Vergari^2^; A. Priori^3^; F. Cogiamanian^2^

##### ^*1*^ *Department of Medicine and Surgery, University of Parma, Ospedale Maggiore, Parma, Italy;* ^*2*^ *Department of Neurophysiology, Fondazione IRCCS Ca' Granda Ospedale Maggiore Policlinico, Milan, Italy;* ^*3*^ *Department of Neurology, ASST Santi Paolo e Carlo, Ospedale San Paolo, Milan, Italy*

**Background and Aims:** The diaphragm is the main respiratory muscle, controlled by the phrenic nerve, essential for breathing. Diaphragmatic dysfunction affects up to 43% of lung transplant (LTx) patients due to phrenic nerve injury from surgical factors, causing respiratory complications. This thesis combines two investigations: a prospective cohort study of longitudinal diaphragmatic function pre‐ and post‐LTx, and an intraoperative neurophysiological monitoring sub‐study of phrenic nerve damage dynamics.

**Methods:** Thirty patients were assessed for respiratory function, diaphragm ultrasound, and phrenic conduction preoperatively, at discharge, 6 and 12 months. Three patients underwent intraoperative testing at multiple surgical points, focusing on ice slush effects.

**Results:** Despite good clinical recovery (spirometry, 6MWT), phrenic cMAP amplitude reduced at discharge and 6 months but normalized by 12 months, underscoring multimodal monitoring needs. Intraoperative tests showed immediate cMAP drop with ice slush (*p* < 0.05) suggesting cryogenic axonal damage, emphasizing the utility of intraoperative neurophysiological monitoring in detecting and potentially mitigating nerve damage.

**Figure:**
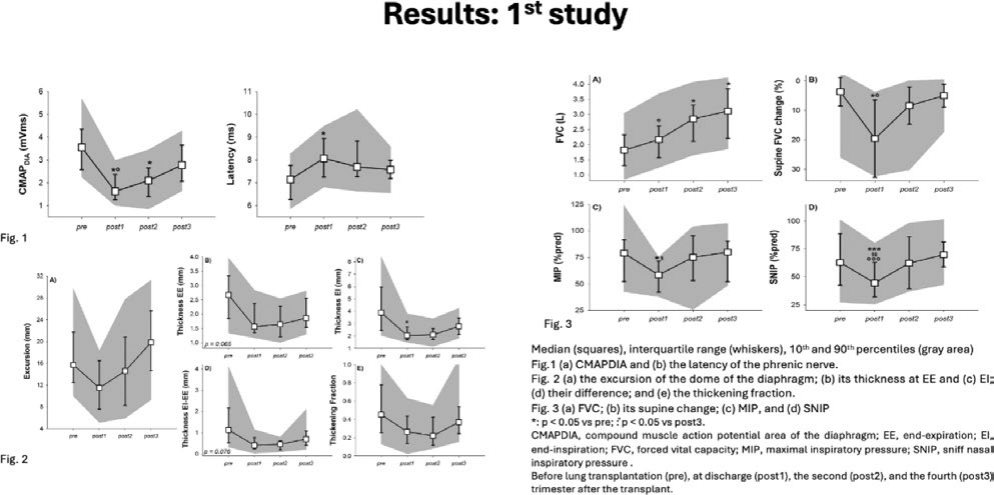
**FIGURE 1** Longitudinal changes in phrenic nerve cMAP amplitude and respiratory parameters in 30 lung transplant patients.

**Figure:**
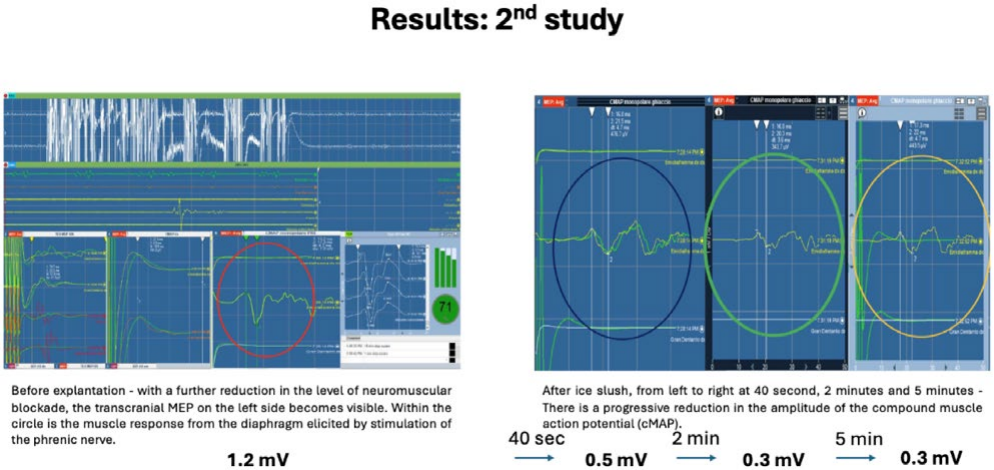
**FIGURE 2** Intraoperative neurophysiological monitoring of phrenic nerve during lung transplantation. Immediate cMAP amplitude reduction after ice slush application, indicating cryogenic axonal damage.

**Figure:**
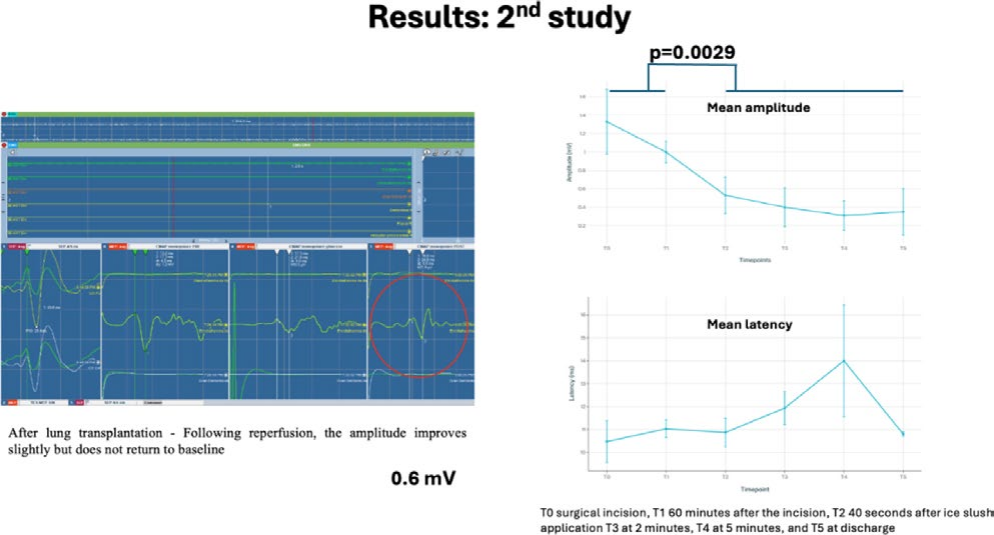
**FIGURE 3** Statistical representation of phrenic cMAP reduction post‐ice slush (*p* = 0.0029) and latency prolongation, indicating cryogenic nerve damage despite partial subsequent recovery.

**Conclusion:** This study highlights the importance of routine diaphragm assessment during and after surgery to identify phrenic nerve damage mechanisms, guide strategies to reduce respiratory complications, and ultimately improve management protocols and optimize transplant outcomes.

**Disclosure:** Nothing to disclose.

## OPR‐058

### Anti‐PLP1 antibody as a biomarker in acute and chronic polyradiculoneuropathies

#### G. Taieb^1^; T. Nguyen^1^; J. El‐Bechir^2^; C. Roué^2^; S. Masciocchi^3^; M. Gasltaldi^3^; A. Jentzer^4^; J. Devaux ^1^

##### ^*1*^ *Department of Neurology, Centre Hospitalier Universitaire Montpellier, Hôpital Gui de Chauliac, Montpellier, France;* ^*2*^ *Institut de Génomique Fonctionnelle, Université de Montpellier, CNRS, INSERM, Montpellier, France;* ^*3*^ *Neuroimmunology Laboratory and Neuroimmunology Research Section, IRCCS Mondino Foundation, Pavia, Italy;* ^*4*^ *Laboratory of Immunology, Centre Hospitalier Universitaire Montpellier, Montpellier, France*

**Background and Aims:** Up to 24% of patients with combined central and peripheral demyelination (CCPD) harbor IgG antibodies against proteolipid protein 1 (PLP1), a key component of CNS and PNS myelin. Given peripheral demyelination in these cases, we assessed anti‐PLP1 prevalence in Guillain‐Barré syndrome (GBS) and chronic inflammatory demyelinating polyradiculoneuropathy (CIDP).

**Methods:** Sera from GBS (*n* = 66), CIDP (*n* = 46), and CCPD (*n* = 16) were tested for IgG/IgM against PLP1 using live cell‐based assays on HEK293 cells as well as oligodendrocyte and myelinating Schwann cell/dorsal root ganglion cocultures. Clinical features of seropositive and seronegative patients were compared. Controls included autoimmune nodopathies (*n* = 25), Charcot‐Marie‐Tooth disease (*n* = 20), and healthy donors (*n* = 23).

**Results:** Anti‐PLP1 antibodies were found in 17% of GBS patients (IgG: *n* = 3; IgM: *n* = 8), associated with greater disability (ONLS 8 vs. 5; *p* = 0.019), sensory‐motor subtype (91% vs. 47%; *p* = 0.008), AIDP (91% vs. 51%; *p* = 0.014), and higher CSF protein (0.88 vs. 0.6 g/L; *p* = 0.011). In CIDP, 24% were seropositive (IgG: *n* = 1; IgM: *n* = 10), linked to older age (66 vs. 55 years; *p* = 0.003), greater disability (ONLS 6 vs. 4; *p* = 0.041), and reduced IVIg response (36% vs. 70%; *p* = 0.05). Four CCPD patients (25%) were positive. All controls were negative. Antibody binding to oligodendrocytes confirmed reactivity.

**Conclusion:** Anti‐PLP1 antibodies, mainly IgM, correlate with severe demyelinating GBS and typical CIDP with greater disability and poorer IVIg response. These antibodies likely target conformational epitopes on myelin. Further studies are needed to confirm these findings and to determine the pathogenic actions of these antibodies.

**Disclosure:** Nothing to disclose.

## OPR‐059

### Evaluating peripherin as a biomarker of axonal damage in chemotherapy‐induced peripheral neuropathy

#### M. Awadelkareem^1^; R. Bellanti ^1^; D. Bennett^1^; M. Lunn^2^

##### ^*1*^ *Nuffield Department of Clinical Neurosciences, University of Oxford, Oxford OX3 9DU, UK;* ^*2*^ *Department of Neuromuscular Diseases, Queen Square Institute of Neurology, University College London, London, UK*

**Background and Aims:** Chemotherapy‐induced peripheral neuropathy (CIPN) is a common, often dose‐limiting adverse effect of several chemotherapeutic agents, preferentially affecting sensory neurons. Sensitive biomarkers of early axonal injury are currently lacking. Neurofilament light chain (NfL) is not specific to sensory neurons. As an initial, proof‐of‐concept approach, we investigated whether peripherin, an intermediate filament enriched in peripheral sensory axons, could serve as a biomarker of CIPN using human induced pluripotent stem cell (iPSC)‐derived sensory neuron cultures, aiming to inform subsequent evaluation in patient samples.

**Methods:** Human iPSC‐derived sensory neurons were exposed to a range of concentrations of vincristine or paclitaxel. Culture media were collected at 24, 48, and 72 h and analysed for peripherin using Simoa and NfL using an electrochemiluminescence assay. Axonal integrity was quantified by an axonal degeneration index using immunostaining and confocal microscopy. To explore mechanistic linkage, parallel cultures were treated with a small‐molecule SARM1 inhibitor to block the Wallerian degeneration pathway.

**Results:** Peripherin release into culture media increased in a dose‐ and time‐dependent manner with both vincristine and paclitaxel, correlating with the progression of the axonal degeneration index and levels of NfL. Levels of both biomarkers were lower in cultures treated with SARM1 inhibitor, indicating reduced axonal degeneration.

**Conclusion:** In vitro, peripherin shows promise as a biomarker of chemotherapy‐related axonal damage. Work is underway to measure peripherin and NfL in large, deeply phenotyped patient cohorts of CIPN. We will determine whether axonal fluid biomarkers correlate with clinical features and existing outcome measures, and evaluate their individual and combined contributions to clinical evaluation.

**Disclosure:** Nothing to disclose.

## Coma and Chronic Disorders of Consciousness

## OPR‐060

### Reconstructing treatment preferences for patients with disorders of consciousness: A longitudinal qualitative study

#### N. Kok ^1^; J. Van Gurp^1^; W. Van Erp^2^

##### ^*1*^ *Radboudumc, Department of IQ Health, Section Ethics of Healthcare, Nijmegen, The Netherlands;* ^*2*^ *Radboudumc, Department of Primary Care, Nijmegen, The Netherlands*

**Background and Aims:** For patients with neurological conditions that impair their capacity to participate in shared decision‐making, guidelines recommend that physicians help surrogates to reconstruct these patients' treatment preferences. This study's objective was to explore the way physicians reconstruct preferences for patients with disorders of consciousness (DoC).

**Methods:** This 2‐year longitudinal qualitative case study took place in the specialized Dutch chain of care patients with DoC. Eligible patients (>18 years of age) had a Coma Recovery Scale‐revised based diagnosis of unresponsive wakefulness syndrome or minimally conscious state. Interactions between physicians and surrogates were audio‐recorded. Additionally, we interviewed healthcare professionals.

**Results:** Characteristics of included patients are reported in Table 1. Across 16 observed healthcare trajectories, 46 physician‐surrogate interactions were audio‐recorded and 32 healthcare professionals were interviewed. We identified five recurring activities physicians' support of surrogates in reconstructing treatment preferences (Table 2): (1) clarifying responsibilities, (2) exploring clues that point to patient's preferences, e.g., the patient's described personality or identity and verbal advance directives, (3) discussing the preferences revealed by the patient's behavior, (4) establishing the minimal level of functioning thought to result in an acceptable quality of life, and (5) discussing sources of surrogate bias. More interactions involved an exploration of patients' personality or identity (51.2%) than prior expressed wishes (25.6%). Inferring preferences from the patient's behavior was important for continuing or withdrawing life‐sustaining treatment.

**TABLE 1** Case characteristics.

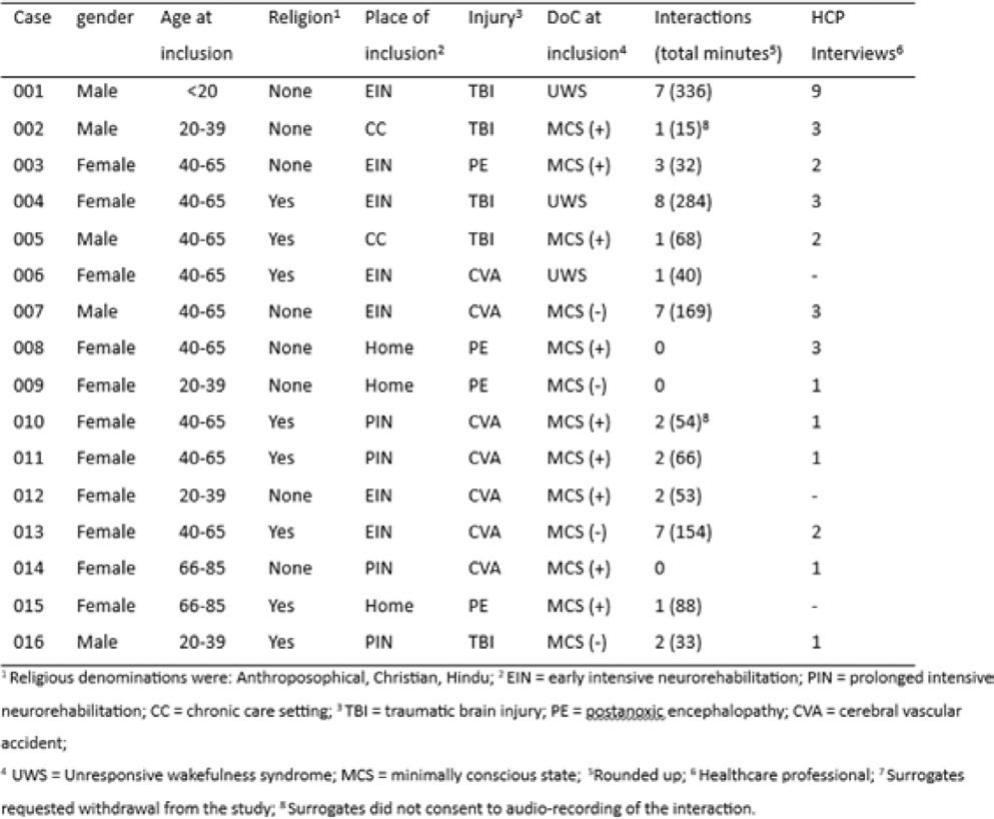

**TABLE 2** Physician‐activities in reconstructed judgment.

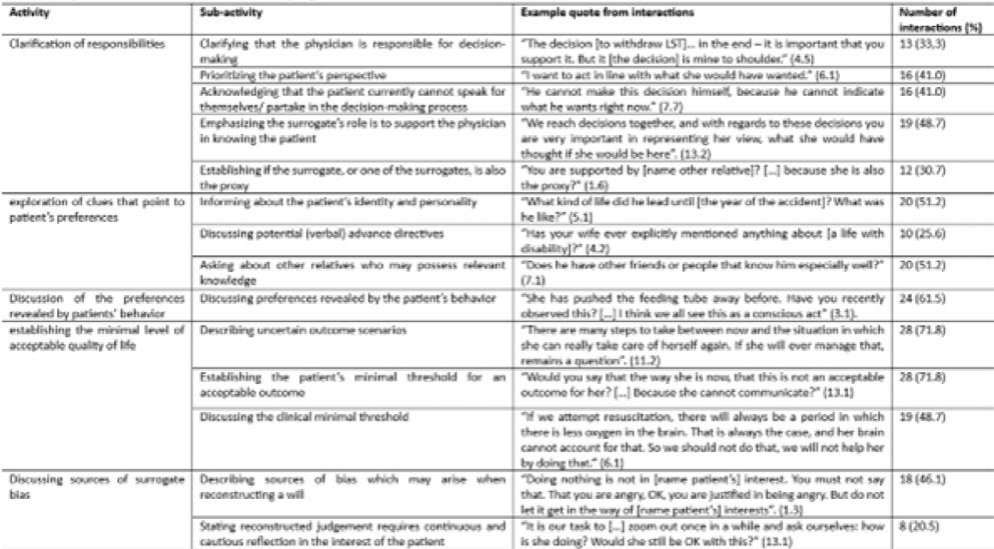

**Conclusion:** This qualitative analysis of reconstructed judgement for patients with DoC shows physicians' activities are mainly focused on triangulating multiple sources for the reconstructed judgement across several sources, including patients' family members.

**Disclosure:** Nothing to disclose.

## OPR‐061

### Pupillary light‐off latency predicts 7‐day improvement in consciousness in patients with acute disorders of consciousness

#### P. Laigaard ^1^; S. Stückler^1^; A. Eigenbrodt^1^; M. Hassani^1^; N. Kühl^2^; D. Kondziella^1^

##### ^*1*^ *Department of Neurology, Copenhagen University Hospital – Rigshospitalet, Copenhagen, Denmark;* ^*2*^ *Department of Applied Mathematics and Computer Science, Cognitive Systems, Danish Technical University (DTU), Lyngby, Denmark*

**Background and Aims:** Objective: To determine whether features of the late light off response (LOR) from automat‐ed pupillometry predict recovery of consciousness over clinically meaningful follow up inter‐vals in patients with acute brain injury in the Intensive Care Unit (ICU).

**Methods:** In a prospective longitudinal cohort (*n* = 250) with impaired consciousness after traumatic or non traumatic brain injury, we performed daily pupillometry paired with serial neurological examinations for up to 20 ICU days. Within a single standardized measurement, we quantified the pupillary light reflex (PLR) and decomposed the LOR into early and late components. The primary predictor was late LOR latency, defined as T50 dilation occurring ≥3 s after light offset, normalized to age and sex matched healthy controls (*n* = 30). Associations with subsequent change in consciousness were analyzed using mixed effects models predicting Full Outline of UnResponsiveness (FOUR)‐score at +7 days, adjusting for ICU Day, baseline FOUR, time since injury, sedation, and injury etiology.

**Results:** Late LOR latency was dissociated from same day behavioral responsiveness yet independently predicted improvement in FOUR scores seven days later, beyond baseline FOUR and time since injury. The prognostic relationship was modified by sedation status and etiology, with effects most evident in non‐sedated patients and predominantly among those with anoxic–ischemic injury (etiology interactions exploratory). In contrast, PLR based metrics, including Neurological Pupil Index and PLR latency, did not predict later gains in consciousness despite being within normal ranges early after injury.

**Figure:**
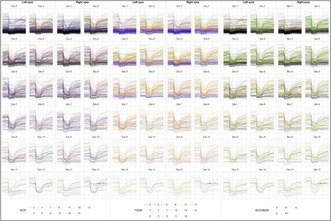
**FIGURE 1** Longitudinal pupillary reflex trajectories across ICU days and levels of consciousness. Daily pupillometry recordings are shown for 250 DoC patients (truncated at ICU day 12).

**Figure:**
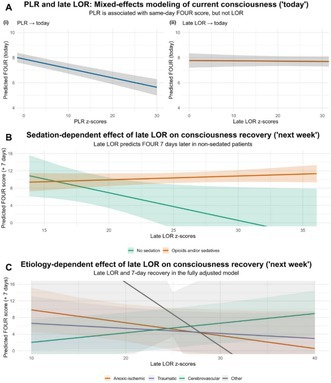
**FIGURE 2** Modeling of consciousness state and recovery. (A) Partial effect plots from linear mixed‐effects models (adjusted for ICU day). (B) Sedation‐stratified sensitivity analysis (mixed model adjusted for ICU day). (C) Etiology‐dependent analysis.

**Figure:**
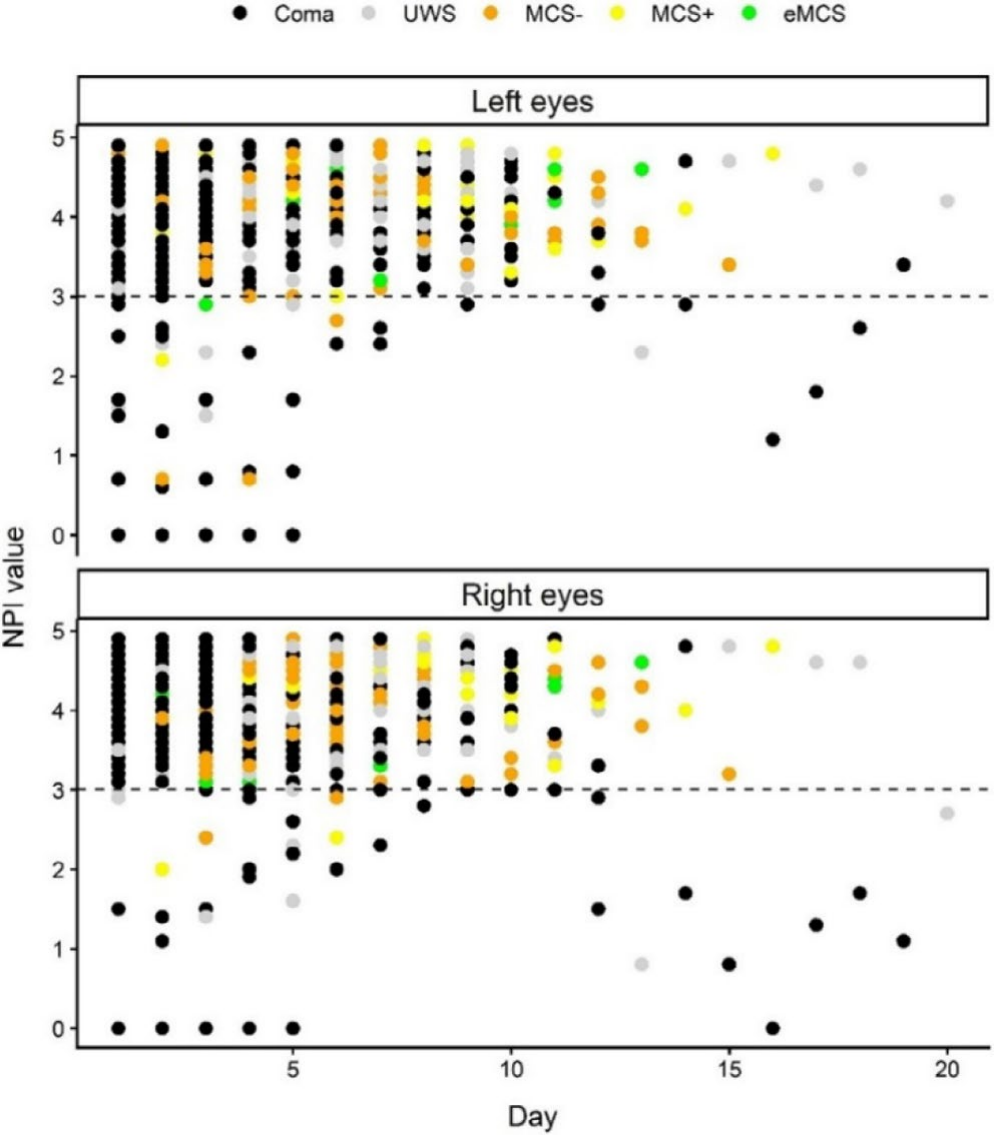
**FIGURE 3** Longitudinal NPi values across ICU days and levels of consciousness. Daily NPi recordings are shown for 250 DoC patients.

**Conclusion:** Conclusion: Late LOR latency captures recovery related pupillary dynamics that anticipates clinically meaningful improvement in consciousness.

**Disclosure:** This project was funded by the Lundbeck Foundation (R507‐2025‐286) and the Independent Research Fund Denmark. Foundation (5333‐00034B). Additional funding was supplied by Offerfonden. Material execution, content and results are the sole responsibility of the authors. The assessments and views expressed in the material are the authors' own and are not nec‐essarily shared by the Offerfonden.

## OPR‐062

### Baseline pupillometry reveals dissociation between the pupillary light reflex and late light off response in acute brain injury

#### S. Stückler ^1^; P. P. Laigaard^1^; A. Eigenbrodt^1^; M. Hassani^1^; N. Kühl^2^; D. Kondziella^1^

##### ^*1*^ *Department of Neurology, Copenhagen University Hospital – Rigshospitalet, Copenhagen, Denmark;* ^*2*^ *Department of Applied Mathematics and Computer Science, Cognitive Systems, Danish Technical University (DTU), Lyngby, Denmark*

**Background and Aims:** We investigated whether distinct components of the automated pupillary reflex – including the pupillary light‐off response (LOR) – measured in the intensive care unit (ICU) reflect level of consciousness after acute brain injury, beyond summary metrics such as the Neurological Pupil Index (NPi).

**Methods:** In a prospective cohort of 250 ICU patients with acute brain injury and impaired consciousness, alongside 30 age‐ and sex‐matched healthy controls, we performed standardized automated pupillometry during the early ICU period. Within a single measurement, we quantified the pupillary light reflex (PLR), decomposed LOR into early and late components, and computed late LOR latency (T50: time to 50% dilation ≥3 s after light offset), normalized to controls. Associations with concurrent level of consciousness, measured by the Full Outline of UnResponsive‐ness (FOUR) score, were assessed using mixed effects models adjusted for ICU Day, baseline severity, sedation, and injury etiology.

**Figure:**
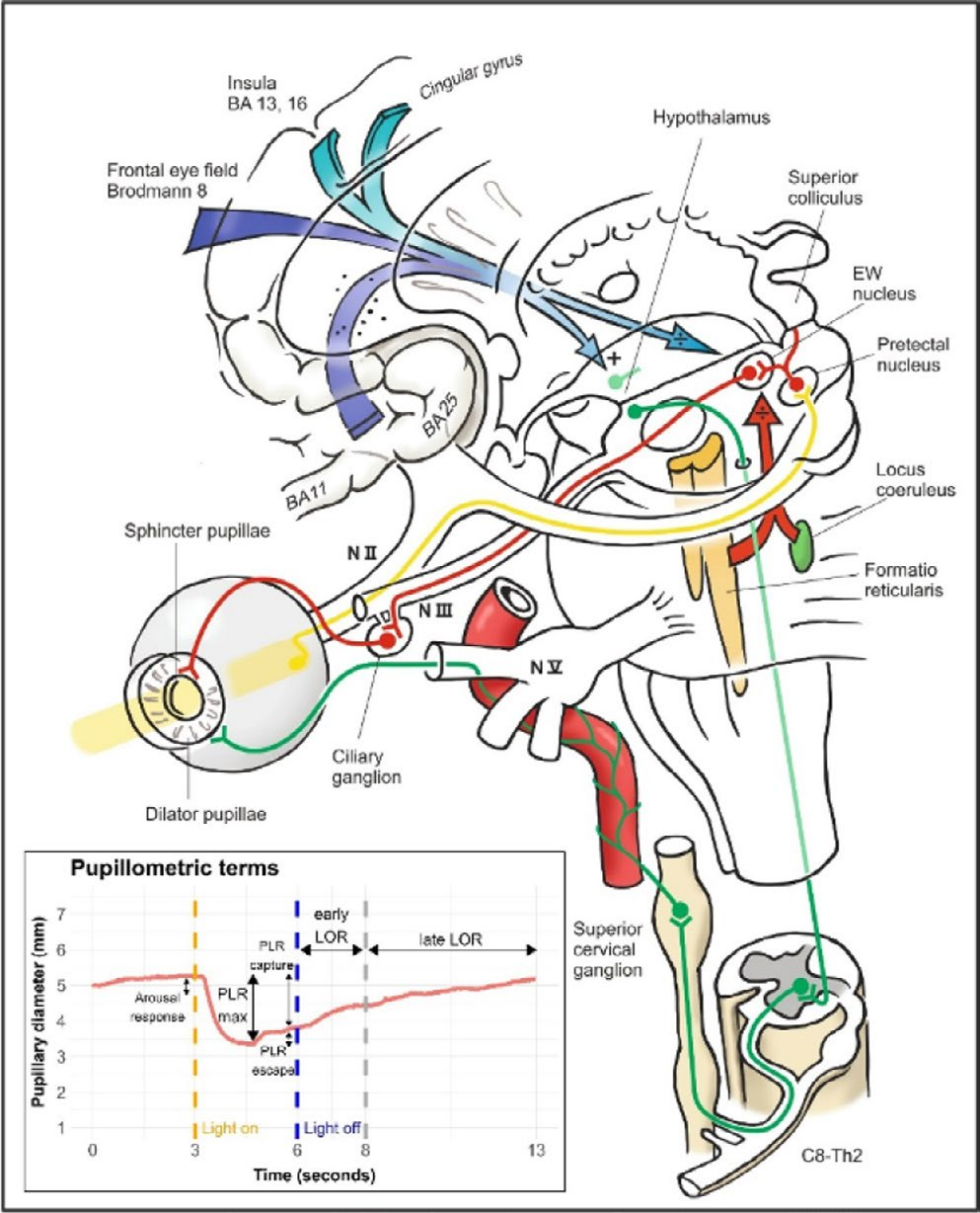
**FIGURE 1** Neural pathways underlying pupillary reflexes and terminology used in this study.

**Results:** In acute coma, PLR and early LOR components were largely preserved. In contrast, late LOR latency was markedly attenuated despite normal NPi‐values and showed substantial interindividual variability. Unsupervised hierarchical clustering of raw pupillary waveforms demonstrated a selective loss of delayed pupillary dilation early after injury. PLR latency showed a strong asso‐ciation with same day FOUR scores, whereas late LOR latency was not correlated with concurrent behavioral responsiveness.

**Figure:**
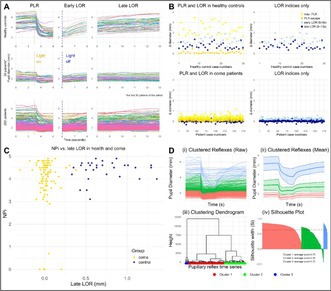
**FIGURE 2** Pupillary indices in healthy controls and acute coma (ICU day 1).

**Conclusion:** Pupillometry reveals dissociable features of the pupillary reflex arc in coma which are not captured by conventional metrics, indicating that PLR and late LOR index distinct aspects of brainstem–arousal network function early after brain injury.

**Figure:**
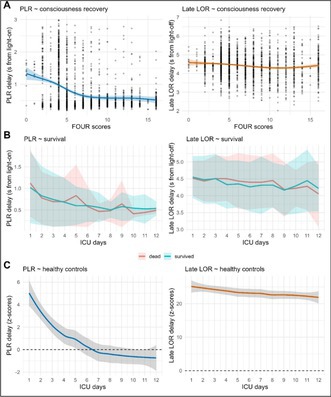
**FIGURE 3** Dissociation between pupillary constriction, late dilation, clinical outcomes, and normalization to healthy controls.

**Disclosure:** This work was funded by the Lundbeck Foundation (R507‐2025‐286) and the Independent Research Fund Denmark. Foundation (5333‐00034B). Additional funding was supplied by Offer‐fonden. Material execution, content and results are the sole responsibility of the authors. The assessments and views expressed in the material are the authors' own and are not necessarily shared by the Offerfonden.

## OPR‐063

### Long‐term outcomes of prolonged disorders of consciousness: A prospective nationwide cohort study

#### D. Driessen^1^; C. Utens^2^; G. Ribbers^1^; L. de Boer‐Schellekens^3^; W. van Erp ^2^; M. Heijenbrok‐Kal^1^

##### ^*1*^ *Rehabilitation Medicine, Erasmus MC, Rotterdam, The Netherlands;* ^*2*^ *Primary Care, Radboudumc, Nijmegen, The Netherlands,* ^*3*^ *Libra Rehabilitation & Audiology, Tilburg, The Netherlands*

**Background and Aims:** Prolonged disorders of consciousness (PDOC) include the unresponsive wakefulness syndrome and the minimally conscious state. Long‐term outcomes following specialized intensive neurorehabilitation are largely unknown, complicating personalized decision‐making. This study aimed to determine the 2‐year outcomes of PDOC patients in the Netherlands regarding consciousness, mortality, disability, functioning, and independence.

**Methods:** Prospective cohort study including PDOC patients ≥16 years admitted to the nationwide Dutch PDOC chain of care (2019–2023). Baseline measurements at admission to Early Intensive Neurorehabilitation (EIN): Coma Recovery Scale‐Revised (CRS‐R), Disability Rating Scale (DRS), Functional Independence Measure (FIM). Follow‐up at 14, 28, 40, 52, and 104 weeks post‐admission. Mortality, recovery of consciousness, functional outcomes, and independence (FIM ≥78) were analysed using descriptive statistics and generalized estimating equations.

**Results:** Of 129 enrolled patients (mean age 38 years; 52% male), 43% regained consciousness during EIN, with an additional 19% within 2 years. Overall, 62% recovered consciousness. Functional independence increased from 4% at 14 weeks to 41% at 2 years among conscious survivors. The most substantial improvements in DRS and FIM occurred between 14–28 weeks post‐admission, with continued, though smaller, gains up to 2 years. Mortality was 26%, predominantly following a decision to withhold or withdraw life‐sustaining treatment.

**Conclusion:** PDOC patients demonstrate significant recovery of consciousness and functional independence during the first 2 years of specialized rehabilitation, predominantly within the first 9 months. These findings emphasize the importance of early, intensive, and prolonged neurorehabilitation and support informed decision‐making in severe acquired brain injury.

**Disclosure:** Nothing to disclose.

## Movement Disorders 1

## OPR‐064

### Alterations of cerebellar volumes and functional connectivity and gait changes in Parkinson's disease subjects with freezing of gait

#### A. Gardoni ^1^; E. Sarasso^2^; A. Grassi^1^; S. Basaia^1^; G. Bonardi^3^; S. Carta^3^; S. Mariotto^3^; I. Di Vico^3^; E. Mantovani^3^; E. Canu^4^; M. Bressan^3^; V. Castelnovo^1^; E. Sibilla^1^; C. Tripodi^1^; F. Freri^1^; R. Balestrino^5^; M. Trentinaglia^3^; F. Maffei^3^; V. Chiodega^3^; S. Tamburin^3^; M. Volontè^6^; M. Tinazzi^3^; F. Agosta^4^; M. Filippi^7^

##### ^*1*^ *Neuroimaging Research Unit, Division of Neuroscience, IRCCS San Raffaele Scientific Institute, Milan, Italy; and Neurotech Hub, Vita‐Salute San Raffaele University, Milan, Italy;* ^*2*^ *Neuroimaging Research Unit, Division of Neuroscience, IRCCS San Raffaele Scientific Institute, Milan, Italy; and Neurotech Hub, Vita‐Salute San Raffaele University, Milan, Italy; and Department of Neuroscience, Rehabilitation, Ophthalmology, Genetics and;* ^*3*^ *University of Verona, Verona, Italy;* ^*4*^ *Neuroimaging Research Unit, Division of Neuroscience, and Neurology Unit, IRCCS San Raffaele Scientific Institute, Milan, Italy; and Neurotech Hub, Vita‐Salute San Raffaele University, Milan, Italy;* ^*5*^ *Neurology Unit, and Neurorehabilitation Unit, IRCCS San Raffaele Scientific Institute, Milan, Italy; and Neurotech Hub, Vita‐Salute San Raffaele University, Milan, Italy;* ^*6*^ *Neurology Unit, IRCCS San Raffaele Scientific Institute, Milan, Italy;* ^*7*^ *Neuroimaging Research Unit, Division of Neuroscience, Neurology Unit, Neurorehabilitation Unit, and Neurophysiology Service, IRCCS San Raffaele Scientific Institute, Milan, Italy; and Neurotech Hub, Vita‐Salute San Raffaele University, Milan, Italy*

**Background and Aims:** Cerebellum plays an increasingly recognized role in gait modulation and freezing of gait (FoG) in Parkinson's disease (PD). This study investigated gait kinematics, cerebellar structural and functional connectivity (FC) changes in PD patients with and without FoG (PD‐FoG, PD‐NO‐FoG).

**Methods:** Thirty PD‐FoG, 30 PD‐NO‐FoG (with postural instability and gait disorders), and 30 healthy controls (HC) underwent clinical, gait analysis and MRI evaluations. Seed‐based FC used cerebellar vermis as region of interest, based on volumetric findings.

**Results:** PD‐FoG showed lower balance confidence and greater impact of symptoms on daily activities than PD‐NO‐FoG, while cognitive performance was similar. PD groups exhibited reduced stride length, foot‐strike angle, and gait velocity. PD‐FoG displayed higher asymmetry, slower Timed‐Up‐and‐Go (TUG) relative to HC, and slower turning velocity, greater stride variability, and shorter turning stride length than PD‐NO‐FoG; while PD‐NO‐FoG showed reduced trunk mobility and gait rhythm relative to HC. MRI analyses revealed reduced cerebellar lobule‐VI volume in PD vs. HC. PD‐FoG showed increased volumes of lobule‐VIIIb, vermis‐X, and vermis‐Crus‐II then PD‐NO‐FoG. Resting‐state FC analyses demonstrated increased vermis connectivity with frontal and cerebellar areas (VI, IX, vermis‐VII, and Crus‐I) in PD‐FoG relative to HC, while PD‐NO‐FoG showed enhanced FC between vermis and postcentral, middle temporal, and occipital cortices. In PD‐FoG, increased cerebellar FC correlated with increased cerebellar volume, which also correlated with worse gait parameters; reduced frontal areas FC correlated with worse depression.

**Conclusion:** PD‐FoG patients displayed specific alterations. Increased FC of cerebellum suggests an attempt to hyper‐control movement that may result in increased volume related to overuse.

**Disclosure:** Supported by Italian Ministry of Health grant GR‐2021‐12374005. A Gardoni, A Grassi, G Bonardi, S Carta, I Di Vico, E Mantovani, M Bressan, VCastelnovo, E Sibilla, C Tripodi, F Freri, M Trentinaglia, F Maffei, V Chiodega, S Tamburin, and MA Volontè declare no financial competing interests. E Sarasso, S Basaia, S Mariotto, E Canu, R Belestrino and M Tinazzi are recipient of grants from the Italian Ministry of Health. M Filippi Filippi is Editor‐in‐Chief of the Journal of Neurology, Associate Editor of Human Brain Mapping, Neurological Sciences, and Radiology; received compensation for consulting services from Almirall, Biogen, Bristol‐Myers Squibb, Eli Lilly, Merck, Novartis, Roche, Sanofi; speaking activities from Amgen, Bayer, Biogen, Bristol‐Myers Squibb, Celgene, Chiesi Italia SpA, Eisai, Eli Lilly, Fujirebio, Genzyme, Janssen, Merck, Neopharmed Gentili, Neuraxpharm, Novartis, Novo Nordisk, Roche, Sanofi, Takeda; participation in Advisory Boards for Alexion, Biogen, Bristol‐Myers Squibb, Eli Lilly, GE Healthcare Ltd, Merck, Neuraxpharm, Novartis, Roche, Sandoz, Sanofi, Takeda; scientific direction of educational events for Biogen, Merck, Roche, Celgene, Bristol‐Myers Squibb, Lilly, Novartis, Sanofi‐Genzyme; he receives research support from Biogen Idec, Merck‐Serono, Novartis, Roche, the Italian Ministry of Health, the Italian Ministry of University and Research, and Fondazione Italiana Sclerosi Multipla. F Agosta is Associate Editor of NeuroImage: Clinical, has received speaker honoraria from Biogen Idec, Italfarmaco, Roche, Zambon, Eli Lilly, GE Healthcare and Bristol Myers Squibb, and receives or has received research supports from the Italian Ministry of Health, the Italian Ministry of University and Research, AriSLA (Fondazione Italiana di Ricerca per la SLA), the European Research Council, the EU Joint Programme—Neurodegenerative Disease Research (JPND) and Foundation Research on Alzheimer Disease (France).

## OPR‐065

### NIO752, a tau‐targeting antisense oligonucleotide, in progressive supranuclear palsy: Phase I results and phase III design

#### C. Serban ^1^; G. Höglinger^2^; A. Boxer^3^; H. Gunduz‐Bruce^4^; N. Pezous^1^; L. Kovacic^1^; A. Pethe^5^; A. Graf^1^; M. Brys^5^

##### ^*1*^ *Novartis Pharma AG, Basel, Switzerland;* ^*2*^ *Neurology, LMU Hospital, Munich, Germany;* ^*3*^ *Fein Memory and Aging Center, Department of Neurology, University of California, San Francisco, USA;* ^*4*^ *Novartis BioMedical Research, Cambridge, USA;* ^*5*^ *Novartis Pharmaceuticals, East Hanover, USA*

**Background and Aims:** Progressive supranuclear palsy (PSP) is a rare, debilitating primary tauopathy with no effective therapies. NIO752, an antisense oligonucleotide (ASO), inhibits MAPT mRNA translation, reducing production of all tau protein isoforms and pathogenic aggregation into neurofibrillary tangles, and thereby potentially attenuating tau‐mediated neurodegeneration. NIO752 is being investigated in PSP for its potential to slow clinical progression. Here we present results from the first‐in‐human Phase I study (NCT04539041) of NIO752 in PSP and Phase III development plans.

**Methods:** In the Phase I, randomized, double‐blind, placebo‐controlled, multiple dose–escalation study, eligible patients had a PSP diagnosis for <5 years classified as probable PSP Richardson syndrome (PSP‐RS). Patients received intrathecal NIO752 or placebo. The Phase III randomized, double‐blind, placebo‐controlled, parallel group study will enroll patients with possible/probable PSP‐RS for <5 years to receive NIO752 or placebo. The goal is to evaluate efficacy and safety of NIO752.

**Results:** In the Phase I study, 59 patients were enrolled to receive NIO752 (*n* = 45) or placebo (*n* = 14). Median time since diagnosis was 1.6 years (range: 0.3–4.8 years). NIO752 had an acceptable safety profile; most adverse events were mild to moderate and consistent with the nature of PSP. Serious adverse events were reported in treatment and placebo cohorts at similar rates. Pharmacokinetic profile and exploratory biomarker data will also be presented.

**Conclusion:** NIO752 is the first MAPT ASO investigated as a potential PSP treatment. Phase I results support proceeding to Phase III to establish the potential of NIO752 as a disease‐modifying therapy in PSP‐RS.

**Disclosure:** Adam Boxer: Paid consultant to Novartis Mirek Brys: Novartis employee and stockholder Ana Graf: Novartis employee and stockholder Handan Gunduz‐Bruce: Novartis employee and stockholder Gunter Hoglinger: Received consultancy fee from Novartis Lidija Kovacic: Novartis employee and stockholder Abhijit Pethe: Novartis employee and stockholder Nicole Pezous: Novartis employee and stockholder Carmen Serban: Novartis employee and stockholder.

## OPR‐066

### Efficacy and safety of dimethyl fumarate in Friedreich ataxia: A phase 2, randomized, double‐blind, placebo‐controlled DMF‐FA‐201 trial

#### C. Pane; M. Gramaglia; A. Sarnataro; A. Bonfini Rendina; G. Puorro; A. Marsili; C. Giannini; C. Linguetta; V. Giordano; L. Aliberti; G. Esposito; A. De Mare; A. Cittadini; A. Marra; F. Saccà

##### University of Naples Federico II, Napoli, Italy

**Background and Aims:** FA is an autosomal recessive neurodegenerative disease. Compared to controls, FXN gene expression is 20% in patients and 50% in healthy carriers. Treatment of multiple sclerosis patients with DMF is known to increase FXN expression by 70%. Objective of the study was to evaluate the efficacy and safety of Dimethyl fumarate (DMF) in patients with Friedreich Ataxia (FA).

**Methods:** We conducted a phase II trial with a randomized, placebo‐controlled, 12 weeks phase, and an identical extension open‐label phase (Figure 1). Primary endpoint was the efficacy of DMF on FXN gene transcription. Secondary objectives were: frataxin protein, stimulation of the nrf2 pathway and mitochondrial biogenesis, safety and tolerability, and clinical measures of disease (Figure 2). EudraCT number: 2021‐006274‐23.

**Figure:**
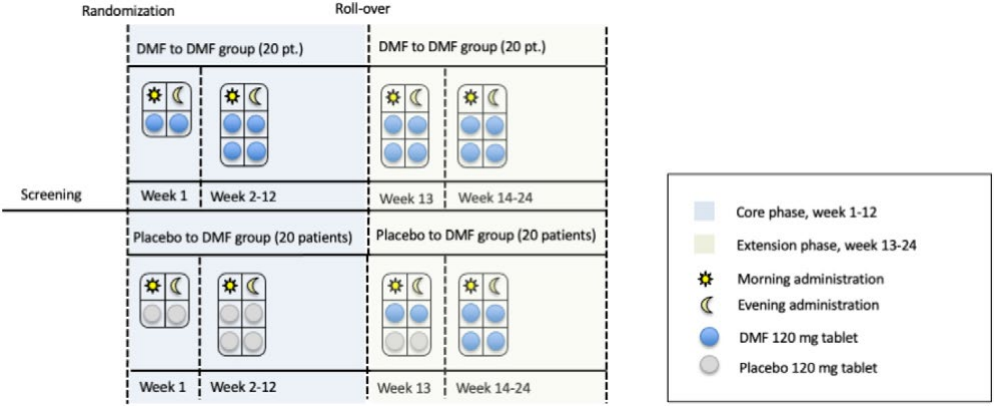
FIGURE 1

**Figure:**
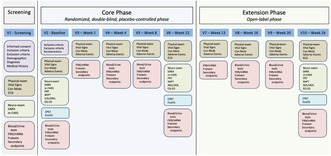
FIGURE 2

**Results:** I40 patients (female, 57.5%; mean [SD] age, 36.1 [12.6] years; disease duration 16.8 [6.9] years), were randomized (DMF *n* = 20, placebo *n* = 20). At the end of the core phase, 33 patients continued to the open‐label extension. DMF increased FXN gene expression after 12 weeks of treatment (difference compared to placebo +119.0%, 95% CI +55.3, +182.7, *p* = 0.018; Figure 3). In the DMF‐DMF group FXN expression increased to +205% after 24 weeks compared to baseline (*p* < 0.001). Secondary endpoints showed a non‐significant trend towards improvement after treatment. Frequent adverse events were gastrointestinal, flushing, eosinophilia, lymphopenia, and elevated liver enzymes.

**Figure:**
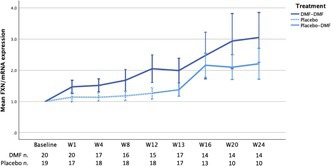
FIGURE 3

**Conclusion:** DMF significantly increases transcription of the FXN gene, with long‐term stability, to a degree that restores expression levels to those of healthy carriers. The drug appeared safe, with adverse events consistent with DMF pharmacovigilance.

**Disclosure:** Francesco Saccà received public speaking honoraria from Alexion, argenx, Biogen, Genpharm, Johnson&Johnson, Medpharma, Medison Pharma, Neopharm Israel, Novartis, UCB, Zai Lab; he also received compensation for Advisory boards or consultation fees from Alexion, Amgen, argenx, Astrazeneca, Avexis, Biogen, Dianthus, Johnson&Johnson, Lexeo, Novartis GmBh, Reata, Sandoz, UCB, Zai Lab; he is PI in clinical trials for Alexion, argenx, Carthesian, Dianthus, Immunovant, Lediant, Lexeo, Novartis, Prilenia, Remgen, Sanofi. The other authors report no disclosures. Funding: Agenzia Italiana del Farmaco (AIFA), Associazione Italiana per la lotta alle Sindromi Atassiche (AISA), Freidreich Ataxia Research Alliance (FARA), Roche SpA, Tristan Allamby Research Fund (TARFfa), Alexion.

## OPR‐067

### Safety and efficacy of dual‐target MRgFUS thalamotomy for dystonic tremor: A prospective single‐centre cohort

#### F. Paio ^1^; G. Ricciardi^2^; G. Bulgarelli^3^; M. Tagliamonte^2^; T. Bovi^4^; M. Longhi^3^; A. NIcolato^3^; F. Sala^5^; B. Petralia^2^; B. Bonetti^4^; M. Tinazzi^1^; S. Tamburin^1^

##### ^*1*^ *Neurology Section, Department of Neurosciences, Biomedicine, and Movement Sciences, University of Verona, Verona, Italy;* ^*2*^ *Neuroradiology Unit, Department of Pathology and Diagnostics, Azienda Ospedaliera Universitaria Integrata, Verona, Italy;* ^*3*^ *Stereotactic Neurosurgery and Radiosurgery Unit, Department of Neurosciences, Azienda Ospedaliera Universitaria Integrata, Verona, Italy;* ^*4*^ *Neurology Unit, Department of Neurosciences, Azienda Ospedaliera Universitaria Integrata, Verona, Italy;* ^*5*^ *Neurosurgery Section, Department of Neurosciences, Biomedicine, and Movement Sciences, University of Verona, Verona, Italy*

**Background and Aims:** MR‐guided focused ultrasound (MRgFUS) is an established treatment for essential tremor when targeting the ventral intermediate nucleus (VIM), whereas its efficacy in dystonic tremor (DT) remains uncertain. Emerging evidence suggests that a more anterior targeting may improve clinical outcomes. Based on the involvement of distinct circuits, we explored a dual‐target approach combining VIM and Ventralis oralis (Vo) targeting.

**Methods:** Initial treatment targeted the VIM using standard stereotactic coordinates. In cases of partial tremor control, additional targeting of the most anterior (i.e., pallidal) portion of the Vo was performed. Efficacy was assessed by changes in CRST A + B scores of the treated hand at 1 and 6 months, while functional outcomes were evaluated using CRST Part C. Safety was assessed through systematic collection and grading of adverse events (AEs).

**Results:** Fifteen patients (6 female; mean age 68.3 ± 5.8 years) with DT underwent dual‐target thalamotomy. Significant improvements in tremor severity were observed at both follow‐up timepoints, reflected by reductions in CRST A + B scores of the treated hand (−56% and −60% at 1 and 6 months, respectively). Functional improvement was observed on CRST Part C (−60% and −61% at 1 and 6 months). A total of 22 AEs were recorded across all timepoints, all of which were mild. At the last available follow‐up, only two AEs persisted (worsening of pre‐existing gait instability and finger hypoesthesia), both occurring in the same patient.

**Figure:**
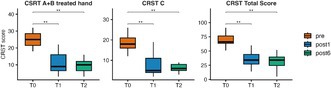
**FIGURE 1** Boxplots of the Clinical Rating Scale for Tremor (CRST) scores at T0, T1, and T2, showing a significant post‐treatment reduction in CRST A + B for the treated hand, CRST C, and total CRST scores.

**Figure:**
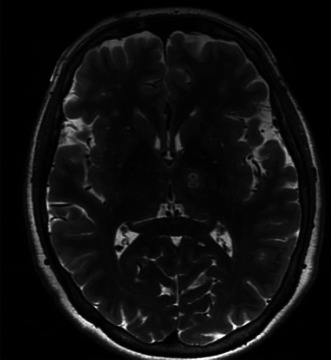
**FIGURE 2** Axial T2 MRI sequence showing dual lesion at 1‐month follow‐up.

**Conclusion:** These preliminary findings suggest that a dual‐target MRgFUS thalamotomy approach is feasible in patients with DT and is associated with an acceptable safety profile.

**Disclosure:** Nothing to disclose.

## OPR‐068

### Motor outcomes and network effects of adaptive deep brain stimulation programming in Parkinson's disease

#### P. Serrao^1^; L. Caffi^2^; F. Luiso^1^; C. Palmisano^2^; G. Pezzoli^3^; S. Marceglia^4^; A. Priori^4^; A. Mazzoni^5^; M. Arlotti^6^; L. Rossi^6^; A. Pedrocchi^7^; I. Isaias ^2^

##### ^*1*^ *Parkinson Institute of Milan, ASST G.Pini‐CTO, Milano, Italy;* ^*2*^ *University Hospital of Würzburg and Julius Maximilian University of Würzburg, Würzburg, Germany;* ^*3*^ *Fondazione Pezzoli per la Malattia di Parkinson, Milano, Italy;* ^*4*^ *Università degli Studi di Milano, Aldo Ravelli Research Center for Neurotechnology and Experimental Neurotherapeutics, Dipartimento di Scienze della Salute, Milano, Italy;* ^*5*^ *The BioRobotics Institute, Sant'Anna School of Advanced Studies, Pisa, Italy;* ^*6*^ *Newronika SpA, Milan, Italy;* ^*7*^ *Nearlab, Department of Electronics, Information and Bioengineering, Politecnico di Milano, Milano, Italy*

**Background and Aims:** In Parkinson's disease (PD), adaptive deep brain stimulation (aDBS) of the subthalamic nucleus (STN) may enhance clinical benefits and reduce side effects compared with conventional DBS (cDBS). In single‐drive aDBS (SD‐aDBS), bilateral stimulation is modulated using local field potentials (LFPs) recorded from one hemisphere, making interhemispheric coupling a critical determinant of appropriate stimulation. Here, we investigated the effects of chronic cDBS and SD‐aDBS on subthalamic interhemispheric coupling in PD.

**Methods:** Twelve PD patients were treated in a randomized, double‐blind crossover design with cDBS and SD‐aDBS (AlphaDBS, Newronika) for two consecutive weeks per condition, with dopaminergic medication unchanged. In SD‐aDBS, stimulation amplitude was adjusted every minute using a linear proportional algorithm based on patient‐specific beta‐range STN‐LFP amplitude. Motor symptoms were assessed using the MDS‐UPDRS‐III at baseline (stim‐OFF/meds‐OFF) and after each treatment period (stim‐ON/meds‐ON). ON‐ and OFF‐time percentages were derived from Hauser diaries. Continuous STN‐LFP recordings were decomposed into periodic and aperiodic spectral components. Interhemispheric coupling was quantified using Pearson's *r* ^2^ for aperiodic offset and slope, and low‐beta (12–20 Hz) and high‐beta (21–30 Hz) oscillatory amplitudes.

**Results:** cDBS and SD‐aDBS yielded comparable motor improvement (mean MDS‐UPDRS‐III change: 59% vs. 58%), with greater ON‐time during SD‐aDBS. Ten of twelve patients blindly preferred SD‐aDBS. Interhemispheric coupling was significant under both conditions. Low‐beta interhemispheric coupling was higher during cDBS than SD‐aDBS and, during SD‐aDBS, positively correlated with OFF‐time.

**Conclusion:** Chronic cDBS and SD‐aDBS differentially modulate subthalamic interhemispheric coupling, with a greater reduction during SD‐aDBS. This mechanism may contribute to its additional clinical value.

**Disclosure:** M.A. is an employee of Newronika S.p.A. L.R., S.M., and A.P. are founders and shareholders of Newronika S.p.A. I.U.I. is a Newronika S.p.A. consultant and shareholder and received funding for research activities from Newronika S.p.A. I.U.I. received lecture honoraria and research funding from Medtronic Inc. I.U.I. is an Adjunct Professor at the Department of Neurology, NYU Grossman School of Medicine.

## OPR‐069

### α‐Synuclein‐associated mitochondria‐Nrf2 dysfunction in Parkinson's disease olfactory mucosa

#### J. Bissacco ^1^; D. Maftei^1^; M. Di Certo^2^; R. Maurizi^3^; M. Rosina^4^; D. Mascioli^1^; R. Bovenzi^1^; C. Simonetta^1^; M. Mancini^1^; V. Buttarazzi^1^; S. Viola^3^; A. Gravina^3^; M. Maglie^3^; A. Rinaldi^1^; F. Gabanella^2^; L. Saso^5^; A. Stefani^1^; F. Passali^3^; S. Di Girolamo^3^; M. Pierantozzi^1^; C. Valle^6^; A. Ferri^6^; D. Centonze^7^; R. Lattanzi^5^; C. Severini^2^; T. Schirinzi^1^

##### ^*1*^ *Unit of Neurology, Department of Systems Medicine, Tor Vergata University of Rome, Rome, Italy;* ^*2*^ *National Research Council (CNR), Institute of Biochemistry and Cell Biology (IBBC), Rome, Italy;* ^*3*^ *Unit of ENT, Department of Clinical Sciences and Translational Medicine, Tor Vergata University of Rome, Rome, Italy;* ^*4*^ *Department of Neuroscience, Italian National Institute of Health (ISS), Rome, Italy;* ^*5*^ *Department of Physiology and Pharmacology “V. Erspamer”, Sapienza University of Rome, Rome, Italy;* ^*6*^ *IRCCS Fondazione Santa Lucia, Rome, Italy;* ^*7*^ *Neurology Unit – IRCCS Neuromed, Pozzilli (IS), Italy*

**Background and Aims:** In this study we outline the dynamics of the mitochondrial network and cytoprotective response in patients with Parkinson's disease (PD)‐derived olfactory mucosa neurons (ONs) across disease stages.

**TABLE 1** Demographic, clinical, and biochemical features of the study population.

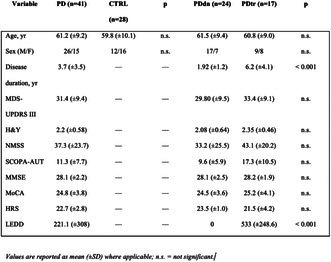

**Methods:** ONs obtained by nasal brush from 41 well‐phenotyped patients with PD (*n* = 24 PD de novo [PDdn] and *n* = 17 under treatment [PDtr]) and 29 healthy controls were examined through real‐time polymerase chain reaction (RT‐PCR), immunofluorescence, and Western blot. An integrative set of experiments using SH‐SY5Y neuronal cells was also performed.

**Results:** PD ONs accumulated α‐synuclein oligomers in association with an aberrant subcellular distribution of mitochondrial markers COX IV and HSP60. DJ‐1 ONs expression was permanently reduced in PD, revealing the mitochondrial dysfunction and justifying the defective Nrf2‐mediated cytoprotective response. The Nrf2/SOD‐1 pathway was indeed downregulated in PD ONs, although with stage‐specific differences. In PDdn ONs, Nrf2 mostly presented in the inactive cytosolic localization with a major reduction of SOD‐1 expression, whereas in PDtr, the Nrf2 active nuclear fraction increased, and the SOD‐1 expression raised. In SH‐SY5Y cells, we demonstrated that dopamine administration increases the Nrf2 nuclear fraction, acting as a possible pathway's inducer.

**Figure:**
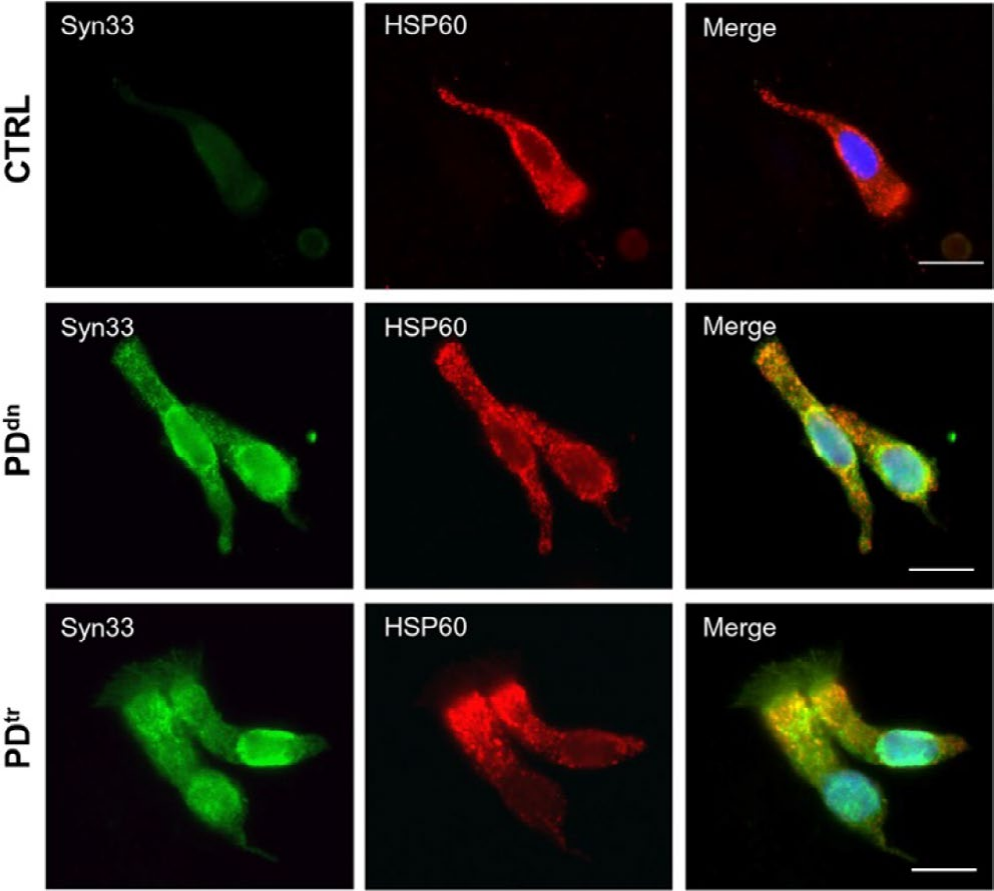
**FIGURE 1** Immunolocalization of oligomeric α‐syn and mitochondria. (A) Representative immunofluorescence of oligomeric α‐syn (Syn33, green) and HSP60 (red) in ONs from healthy controls and PD. Nuclei: DAPI (blue). Scale bar: 10 μm.

**Figure:**
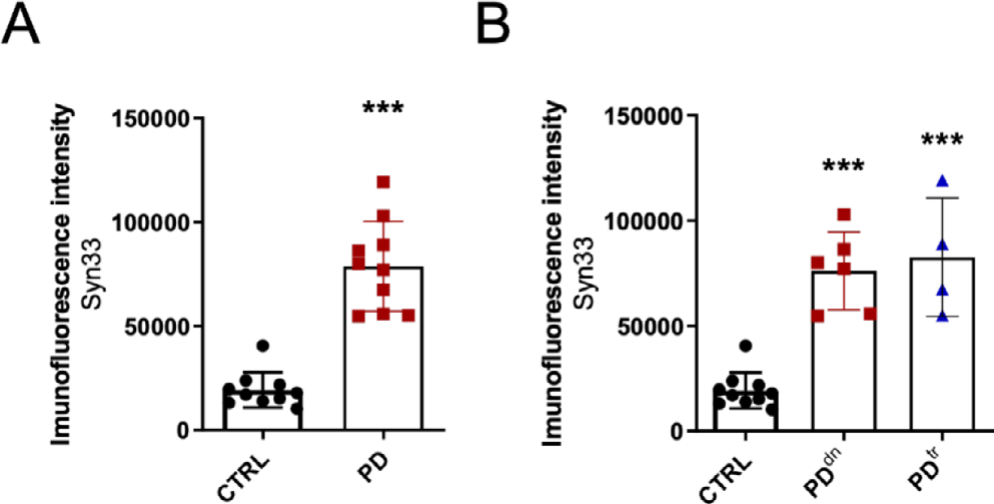
**FIGURE 2** Graphs showing immunofluorescence intensity of oligomeric α‐syn in each group, quantified with ImageJ. Data are mean ± SEM. Asterisks indicate significant differences by unpaired *t* test or 1‐way ANOVA with Tukey post hoc. ***p < 0.001.

**Conclusion:** Human‐derived ONs may recapitulate PD pathogenic milestones, exhibiting stage‐specific interactions among α‐synuclein oligomers, mitochondrial metabolism, and cytoprotective response. These findings highlighted mitochondrial dysfunction as primary target for therapeutic interventions and a potential axis for the biological stratification of patients. Moreover, they supported the translational value of ONs, as a source of biomarkers or models, which is critical in the current changing paradigm of PD toward a biological‐based approach.

**Disclosure:** JB: Italian National Recovery and Resilience Plan (PNRR) “Next Generation EU”, through the Italian Ministry of Health (MCNT1‐2023‐12378408), project titled “Exploring the upper airways through an integrated multidisciplinary approach to tackle pathogenic trajectories and disclose novel targets in Parkinson disease” (CUP MASTER E83C24000720001). DM: the author declares that there are no additional disclosures to report. MGC: the author declares that there are no additional disclosures to report. RM: the author declares that there are no additional disclosures to report. FV: the author declares that there are no additional disclosures to report. MR: the author declares that there are no additional disclosures to report. DM: the author declares that there are no additional disclosures to report. RB: the author declares that there are no additional disclosures to report. CS: the author declares that there are no additional disclosures to report. MM: the author declares that there are no additional disclosures to report. VB: the author declares that there are no additional disclosures to report. SV: the author declares that there are no additional disclosures to report. AG: the author declares that there are no additional disclosures to report. MGM: the author declares that there are no additional disclosures to report. AMR: the author declares that there are no additional disclosures to report. FG: the author declares that there are no additional disclosures to report. LS: the author declares that there are no additional disclosures to report. AS: the author declares that there are no additional disclosures to report. FMP: the author declares that there are no additional disclosures to report. SDG: the author declares that there are no additional disclosures to report. MP: the author declares that there are no additional disclosures to report. CV: the author declares that there are no additional disclosures to report. AF: the author declares that there are no additional disclosures to report. DC: the author declares that there are no additional disclosures to report. RL: the author declares that there are no additional disclosures to report. CS: the author declares that there are no additional disclosures to report. TS: Italian National Recovery and Resilience Plan (PNRR) “Next Generation EU”, through the Italian Ministry of Health (MCNT1‐2023‐12378408), project titled “Exploring the upper airways through an integrated multidisciplinary approach to tackle pathogenic trajectories and disclose novel targets in Parkinson disease” (CUP MASTER E83C24000720001).

## Autonomic Nervous System Diseases 2

## OPR‐070

### Haemodynamics of supine graded exercise and orthostatic blood pressure in autonomic failure

#### A. van der Stam; E. Barnes; Z. Jiang; G. Chiaro; V. Iodice

##### National Autonomic Centre, National Hospital for Neurology and Neurosurgery, London, UK

**Background and Aims:** The prevalence of exercise‐induced hypotension and exacerbation of post‐exercise orthostatic hypotension across phenotypes of autonomic failure is currently not well documented. In this work, we aimed to fill this gap as both symptoms can significantly impair mobility, quality of life and rehabilitation efforts.

**Methods:** We performed a retrospective analysis of blood pressure (BP) and heart rate (HR) recorded with Dinamap during supine rest, supine graded cycling exercise (25 W, 50 W, 75 W) and an active standing test before and after exercise. Additionally, the orthostatic intolerance ratio (OIR) was calculated. Participants (*n* = 140) were diagnosed with Parkinson's disease/Lewy body dementia (PD/LBD, *n* = 32), multiple system atrophy (MSA, *n* = 16), pure autonomic failure (PAF, *n* = 41), autoimmune autonomic ganglionopathy (AAG, *n* = 13), peripheral autonomic neuropathy (*n* = 27), or rare disorders featuring autonomic failure (*n* = 11).

**Results:** Systolic BP (SBP) did not increase during exercise in 69 (52%) people (Figure 1, Table 1). Different patterns were observed in post‐exercise supine recovery of SBP (Figure 2). The magnitude of the SBP drop upon standing did not increase post‐exercise compared to pre‐exercise, but the absolute SBP was lower in those with alpha‐synucleinopathies (Table 1). The OIR only worsened in those with MSA (Table 1). HR increased during exercise and did not fully recover during 10 min of post‐exercise supine rest.

**Figure:**
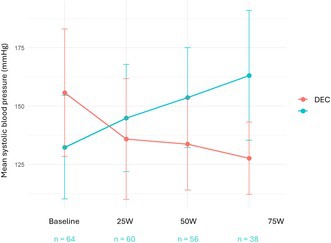
**FIGURE 1** Systolic blood pressure during supine graded exercise, split into those who had an increase in systolic blood pressure during exercise (INC) and those who had a decrease during exercise (DEC) (−19.6 mmHg per exercise increment, *p* < 0.001).

**Figure:**
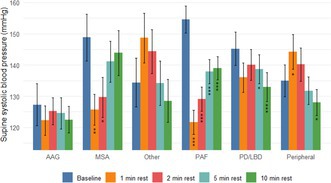
**FIGURE 2** Recovery of supine systolic blood pressure during a 10‐min post‐exercise rest. Measurements were taken at 1, 2, 5 and 10 min after cessation of exercise, and compared to the pre‐exercise supine baseline.*p = <0.05; **p = <0.001.

**TABLE 1** Blood pressure response during and after supine graded exercise. Includes supine and standing SBP, change in SBP and the Orthostatic Intolerance Ratio (OIR). *p = <0.05; **p = <0.001.

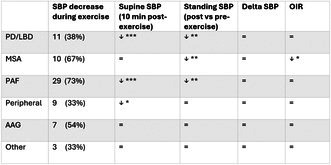

**Conclusion:** An abnormal BP response to exercise is common in autonomic failure, and the baroreflex impairment in alpha‐synucleinopathies exacerbates orthostatic hypotension post‐exercise. Both should be considered when advising on physical activity and rehabilitation in clinic.

**Disclosure:** Nothing to disclose.

## OPR‐071

### Autonomic involvement across the spectrum of genetic prion diseases

#### F. Vitali ^1^; G. Chiaro^1^; T. Mok^2^; S. Mead^2^; V. Iodice^1^

##### ^*1*^ *National Autonomic Centre, National Hospital for Neurology and Neurosurgery, London, UK;* ^*2*^ *NHS National Prion Clinic, National Hospital for Neurology and Neurosurgery, University College London Hospitals NHS Foundation Trust, London, UK*

**Background and Aims:** Genetic prion diseases (gPrDs) are fatal neurodegenerative disorders caused by pathogenic PRNP mutations. Among them, fatal familial insomnia (FFI) and PrP systemic amyloidosis (PrP‐SA) show prominent autonomic nervous system involvement. FFI is marked by selective thalamic degeneration, leading to sleep disruption and cardiovascular sympathetic dysregulation. PrP‐SA, in contrast, involves systemic amyloid deposition affecting peripheral organs and nerves, resulting in early gastrointestinal dysfunction, sensorimotor neuropathy, and widespread autonomic failure.

**Methods:** This study aimed to assess cardiovascular autonomic involvement across gPrDs, focusing on FFI and PrP‐SA. We retrospectively evaluated 5 patients with FFI, 6 with PrP‐SA, and 3 asymptomatic PRNP variant carriers. Cardiovascular autonomic function was assessed using standardized autonomic testing (AFT) with Finapres® NOVA plus and 24‐h blood pressure monitoring.

**Results:** At the time of assessment (median age 44 years; IQR: 33–52), all symptomatic patients (*n* = 11) reported autonomic symptoms, predominantly affecting cardiovascular, gastrointestinal, and urogenital systems. FFI patients exhibited sympathetic overactivity – supine hypertension, nocturnal hypertension, and resting tachycardia – alongside AFT evidence of cardiovagal and adrenergic impairment. In contrast, PrP‐SA showed combined sympathetic and parasympathetic failure, characterized by neurogenic orthostatic hypotension, reduced heart rate variability, and poorer postural tolerance than FFI patients. Asymptomatic carriers displayed subtle AFT abnormalities despite the absence of clinical symptoms.

**Conclusion:** FFI and PrP systemic amyloidosis exhibit distinct autonomic phenotypes – central dysregulation in FFI and peripheral autonomic failure in PrP‐SA. Routine autonomic assessment may facilitate early detection and targeted symptom management across the spectrum of gPrDs.

**Disclosure:** Nothing to disclose.

## OPR‐072

### Linking autonomic dysfunction, immune adrenergic signaling, and neurofilament light chain in multiple sclerosis

#### M. Sredanović ^1^; K. Jerčinović^2^; K. Tešija^1^; A. Savić Mlakar^3^; Ž. Vogrinc^4^; I. Adamec^1^; Ž. Cvetić^3^; T. Tunjić^4^; T. Gabelić^1^; B. Barun^1^; K. Bendelja^3^; M. Krbot Skorić^1^; M. Habek^1^

##### ^*1*^ *University Hospital Center Zagreb;* ^*2*^ *School of Medicine, University of Zagreb, Zagreb, Croatia;* ^*3*^ *Center for Research and Knowledge Transfer in Biotechnology, University of Zagreb;* ^*4*^ *University Hospital Center Zagreb, Department of Laboratory Diagnostics, Zagreb, Croatia*

**Background and Aims:** To investigate the association between autonomic dysfunction and immune cell neurotransmitter receptor expression in people with multiple sclerosis (pwMS), and to explore relationships with serum neurofilament light chain levels (sNfL) as a marker of disease activity.

**Methods:** Autonomic dysfunction was assessed in 68 pwMS using the Composite Autonomic Scoring Scale (CASS). Expression of Adrenoceptor Beta 2 (ADRB2), Nicotinic Acetylcholine Receptor α7, Dopamine Receptors D3 and D5, Interleukin‐1 receptors (IL1R1, IL1R2, IL1R8), and Interleukin‐23 receptor was measured on T‐ and B‐lymphocyte subsets, natural killer (NK) cells, and monocytes. Serum neurofilament light chain levels were quantified using the Roche Elecsys NfL assay.

**Results:** PwMS with objective autonomic dysfunction showed significantly lower ADRB2 expression on total T cells (*p* = 0.037), cytotoxic T cells (*p* = 0.016), including memory and naïve subsets, B lymphocytes (*p* = 0.019), and NK cells (*p* = 0.026), compared with pwMS with normal autonomic function. No differences were observed for other neurotransmitter or cytokine receptors. sNfL levels did not differ between groups; however, sNfL negatively correlated with ADRB2 expression on cytotoxic T cells (*r* = −0.294, *p* = 0.015), including effector/memory and naïve non‐regulatory subsets.

**Conclusion:** Autonomic dysfunction in pwMS is associated with reduced ADRB2 expression across multiple immune cell populations, suggesting altered adrenergic neuroimmune regulation. The inverse relationship between ADRB2 expression on cytotoxic T cells and sNfL levels points to a potential link between impaired adrenergic signaling and inflammatory disease activity in multiple sclerosis.

**Disclosure:** MS: Nothing to disclose. KJ: Nothing to disclose. KT: Nothing to disclose. ASM: Nothing to disclose. ŽV: Nothing to disclose. IA: Nothing to disclose. ŽC: Nothing to disclose. TT: Nothing to disclose. TG: Received consultation and/or speaker fees from Biogen, Merck, Novartis, Roche. BB: Received consultation and/or speaker fees from Biogen, Merck, Novartis, Roche KB: MKS: Received consultation and/or speaker fees from Merck, Novartis, Roche. MH: Received consultation and/or speaker fees from Biogen, Merck, Novartis, Roche, Astra Zeneca, Amgen.

## OPR‐073

### Pattern and progression of sudomotor dysfunction in alpha synucleinopathies

#### Z. Jiang; G. Chiaro; V. Iodice

##### National Autonomic Centre, The National Hospital for Neurology and Neurosurgery, London, UK

**Background and Aims:** Pure autonomic failure (PAF) is an alpha‐synucleinopathy with a 30%–40% risk of phenoconversion to Parkinson's disease (PD), dementia with lewy bodies (DLB), or multiple system atrophy (MSA). While several biomarkers have been proposed to predict phenoconversion, sudomotor dysfunction remains underexplored. This study examined the distribution and longitudinal progression of sweat loss in patients with PAF.

**Methods:** As part of an ongoing natural history study, consecutive patients diagnosed with PAF were systematically phenotyped and followed longitudinally until phenoconversion or death. Sudomotor function was assessed using thermoregulatory sweat testing (TST) and dynamic sweat testing (DST). Combined results were used to localize sudomotor dysfunction as preganglionic, postganglionic, or mixed.

**Results:** 69 PAF patients underwent sudomotor testing: 40/69 (58%) completed TST and 45/69 (65%) completed DST. On TST, 29/40 (73%) demonstrated global anhidrosis, 8/40 (20%) length‐dependent sweat loss, 2/40 (5%) regional anhidrosis, and 1/40 (2%) unilateral anhidrosis. Among those with global anhidrosis, 22/29 (76%) remained PAF, 3/29 (10%) developed DLB, and 4/29 (14%) developed MSA. Patients without global anhidrosis either remained PAF or phenoconverted to PD/DLB. DST showed reduced sweating in 43/45 patients (95.5%) and a predominantly length dependent distribution in patients with stable PAF compared with MSA, with no difference in the rate of sweat output decline over time.

**Figure:**
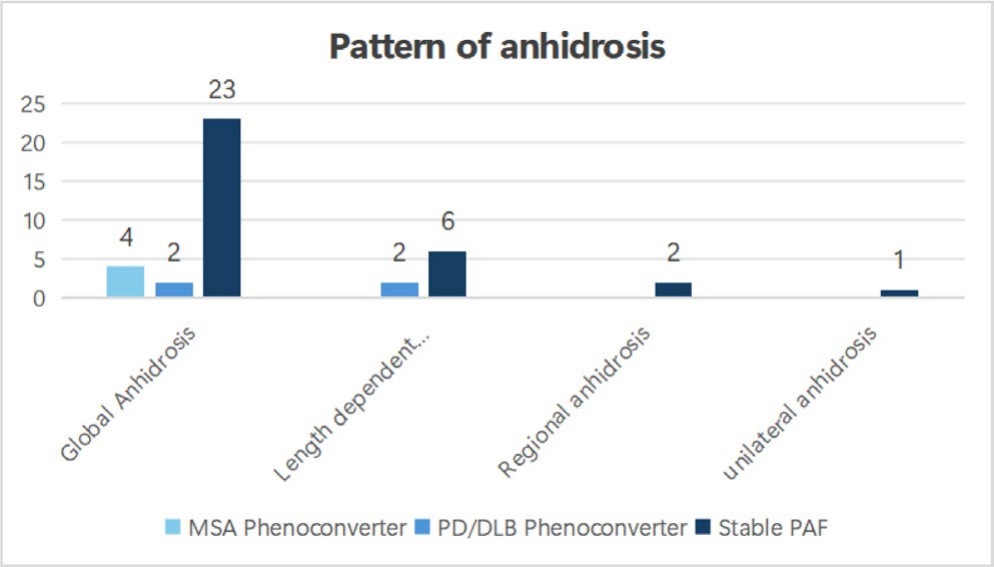
**FIGURE 1** Pattern of anhidrosis.

**Conclusion:** Global anhidrosis is the most common sudomotor pattern in MSA and is associated with a non–length‐dependent impairment of postganglionic sudomotor fibres. In contrast, a length‐dependent sudomotor deficit is more suggestive of a restricted postganglionic alpha‐synucleinopathy, such as PAF.

**Disclosure:** Nothing to disclose.

## Ageing and Dementia 2

## OPR‐074

### Plasma ApoE proteotyping on Lumipulse for ApoE epsilon4 determination: A clinically practical tool in the era of anti‐amyloid therapies

#### F. Coraglia ^1^; G. Cecchetti^2^; G. Rugarli^1^; E. Spinelli^1^; A. Ghirelli^1^; S. Pisano^3^; E. Canu^3^; F. Caso^4^; G. Magnani^4^; M. Filippi^5^; F. Agosta^1^

##### ^*1*^ *Neuroimaging Research Unit, Division of Neuroscience, and Neurology Unit, IRCCS San Raffaele Scientific Institute, Milan, Italy; and Vita‐Salute San Raffaele University, Milan, Italy;* ^*2*^ *Neuroimaging Research Unit, Division of Neuroscience, Neurology Unit, and Neurophysiology Service, IRCCS San Raffaele Scientific Institute, Milan, Italy;* ^*3*^ *Neuroimaging Research Unit, Division of Neuroscience, and Neurology Unit, IRCCS San Raffaele Scientific Institute, Milan, Italy;* ^*4*^ *Neurology Unit, IRCCS San Raffaele Scientific Institute, Milan, Italy;* ^*5*^ *Neuroimaging Research Unit, Division of Neuroscience, Neurology Unit, Neurorehabilitation Unit, and Neurophysiology Service, IRCCS San Raffaele Scientific Institute, Milan, Italy; and Vita‐Salute San Raffaele University, Milan, Italy*

**Background and Aims:** To determine whether plasma‐based quantification of apolipoprotein E4 (ApoE4) and total apolipoprotein E (PanApoE) using a Lumipulse immunoassay can reliably identify APOE epsilon4 carrier status as a practical alternative to genetic testing in patients along the Alzheimer's disease (AD) continuum.

**Methods:** We retrospectively included 110 patients with cognitive impairment evaluated at a tertiary memory clinic. APOE genotyping classified individuals as epsilon4 non‐carriers, heterozygous, or homozygous carriers. Plasma ApoE4 and PanApoE were measured using the Lumipulse 600 G platform, and the ApoE4 to PanApoE ratio was calculated. Additional plasma biomarkers were assessed, including AB40, AB42, pTau181, pTau217, NfL, GFAP, and sTREM2. Diagnostic accuracy for epsilon4 carrier identification was assessed using receiver operating characteristic analyses, deriving area under the curve values and optimal Youden thresholds.

**Results:** The ApoE4 to PanApoE ratio increased stepwise with epsilon4 allele dose, with complete separation between carrier status. For identifying epsilon4 carriers, the ratio achieved perfect discrimination, with an area under the curve of 1.00 and no misclassified individuals at the optimal cut‐off. ApoE4 concentration alone showed substantially lower accuracy. No significant differences were observed in other plasma AD biomarkers between epsilon4 carriers and non‐carriers.

**Conclusion:** The ApoE ratio measured on the Lumipulse platform accurately reflects APOE epsilon4 status and allele dose. This proteotype‐based approach may represent a rapid and accessible alternative to genotyping to support risk stratification and decision‐making in the anti‐amyloid treatment era.

**Disclosure:** We express our gratitude to Fujirebio Italia for their invaluable support in providing part of the research kits free of charge for our scientific investigation. F Coraglia has nothing to disclose; Giordano Cecchetti received speaker honoraria from Neopharmed Gentili; Giulia Rugarli has nothing to disclose; Edoardo Gioele Spinelli has nothing to disclose; Alma Ghirelli has nothing to disclose; Stefano Pisano has nothing to disclose; Elisa Canu receives research support from the Italian Ministry of Health; Francesca Caso has nothing to disclose; Giuseppe Magnani has nothing to disclose; M Filippi is Editor‐in‐Chief of the Journal of Neurology, Associate Editor of Human Brain Mapping, Neurological Sciences, and Radiology; received compensation for consulting services from Almirall, Biogen, Bristol‐Myers Squibb, Eli Lilly, Merck, Novartis, Roche, Sanofi; speaking activities from Amgen, Bayer, Biogen, Bristol‐Myers Squibb, Celgene, Chiesi Italia SpA, Eisai, Eli Lilly, Fujirebio, Genzyme, Janssen, Merck, Neopharmed Gentili, Neuraxpharm, Novartis, Novo Nordisk, Roche, Sanofi, Takeda; participation in Advisory Boards for Alexion, Biogen, Bristol‐Myers Squibb, Eli Lilly, GE Healthcare Ltd, Merck, Neuraxpharm, Novartis, Roche, Sandoz, Sanofi, Takeda; scientific direction of educational events for Biogen, Merck, Roche, Celgene, Bristol‐Myers Squibb, Lilly, Novartis, Sanofi‐Genzyme; he receives research support from Biogen Idec, Merck‐Serono, Novartis, Roche, the Italian Ministry of Health, the Italian Ministry of University and Research, and Fondazione Italiana Sclerosi Multipla. F Agosta is Associate Editor of NeuroImage: Clinical and the European Journal of Neurology; has received speaker honoraria from Biogen Idec, Bristol Myers Squibb, Eisai, Eli Lilly, GE Healthcare, Neuraxpharm, and Roche; and receives or has received research supports from the Italian Ministry of Health, the Italian Ministry of University and Research, AriSLA (Fondazione Italiana di Ricerca per la SLA), the European Research Council (ERC), the EU Joint Programme – Neurodegenerative Disease Research (JPND), and Foundation Research on Alzheimer Disease (France).

## OPR‐075

### Automated diagnosis using deep neural networks: MRI‐based classification of Alzheimer's disease and behavioral variant frontotemporal dementia

#### F. Orlandi ^1^; S. Basaia^2^; S. Pisano^3^; S. Musicco^2^; E. Spinelli^1^; E. Canu^3^; V. Castelnovo^3^; G. Cecchetti^4^; A. Ghirelli^1^; M. Cosseddu^5^; R. Gasparotti^6^; B. Borroni^5^; F. Caso^7^; G. Magnani^7^; P. Caroppo^8^; S. Prioni^8^; C. Villa^8^; E. Stefanova^9^; V. Kostic^9^; F. Agosta^1^; M. Filippi^10^

##### ^*1*^ *Neuroimaging Research Unit, Division of Neuroscience, and Neurology Unit, IRCCS San Raffaele Scientific Institute, Milan, Italy; Vita‐Salute San Raffaele University, Milan, Italy;* ^*2*^ *Neuroimaging Research Unit, Division of Neuroscience, and Neurology Unit, IRCCS San Raffaele Scientific Institute, Milan, Italy;* ^*3*^ *Neuroimaging Research Unit, Division of Neuroscience, and Neurology Unit, IRCCS San Raffaele Scientific Institute, Milan, Italy;* ^*4*^ *Neuroimaging Research Unit, Division of Neuroscience, Neurology Unit, and Neurophysiology Service, IRCCS San Raffaele Scientific Institute, Milan, Italy;* ^*5*^ *Neurology Unit, Department of Clinical and Experimental Sciences, University of Brescia, Brescia, Italy;* ^*6*^ *Neuroradiology Unit, Department of Medical and Surgical Specialties, Radiological Sciences, and Public Health, University of Brescia and ASST Spedali Civili Hospital, Brescia, Italy;* ^*7*^ *Neurology Unit, IRCCS San Raffaele Scientific Institute, Milan, Italy;* ^*8*^ *Neurology V and Neuropathology Unit, C. Besta Neurological Institute and Foundation (IRCCS), Milano, Italy;* ^*9*^ *Clinic of Neurology, Faculty of Medicine, University of Belgrade, Belgrade, Serbia;* ^*10*^ *Neuroimaging Research Unit, Division of Neuroscience, Neurology Unit, Neurorehabilitation Unit, Neurophysiology Service, IRCCS San Raffaele Scientific Institute, Milan, Italy; Vita‐Salute San Raffaele University, Milan, Italy*

**Background and Aims:** Frontotemporal lobar degeneration (FTLD) includes heterogeneous neuropathological and clinical profiles often overlapping with Alzheimer's disease (AD). Neuroimaging is essential for differential diagnosis alongside biomarkers and clinical assessment. We validated a deep learning approach for classifying behavioral variant frontotemporal dementia (bvFTD), AD, and healthy controls (HC) using single high‐resolution T1‐weighted MRI scans.

**Methods:** 3T T1‐weighted MRI scans from 120 bvFTD, 147 AD, and 202 HC from five institutions were analyzed, plus 651 scans (293 AD, 358 HC) from ADNI. 3D convolutional neural networks (CNNs) were implemented in PyTorch using raw MRI volumes. Data were stratified by center and split into training (64%), validation (16%), and test (20%) sets, with random augmentation. Two architectures were evaluated: fully convolutional and convolutional–pooling networks. Performance was measured by balanced accuracy and F1‐score. Grad‐CAM maps were generated for the best model to identify regions driving class‐specific predictions.

**Results:** The best model was the fully convolutional network, achieving 72% balanced accuracy and 77.7% F1‐score with class‐specific F1‐scores of 67% (bvFTD), 76% (AD), and 82% (HC). Grad‐CAM highlighted the right frontal lobe for bvFTD, bilateral parietal cortices for AD, and periventricular regions for HC, demonstrating anatomically plausible patterns.

**Conclusion:** Our findings support structural MRI as a potential biomarker for early disease screening using end‐to‐end CNN models. While performance was lower for bvFTD, likely reflecting heterogeneity and sample imbalance, anatomically plausible patterns were identified. CNN‐based MRI analysis may serve as a complementary tool for early differential diagnosis of neurodegenerative disorders.

**Disclosure:** Supported by European Research Council (StG‐2016_714388_NeuroTRACK). F. Orlandi has nothing to disclose; S. Basaia receives research support from the Italian Ministry of Health; S. Pisano has nothing to disclose; S. Musicco has nothing to disclose; E.G. Spinelli has nothing to disclose; E. Canu receives research support from the Italian Ministry of Health; V. Castelnovo has nothing to disclose; G. Cecchetti has received speaker honoraria from Neopharmed Gentili; A. Ghirelli has nothing to disclose; M. Cosseddu has nothing to disclose; R. Gasparotti has nothing to disclose; B. Borroni has nothing to disclose; F. Caso has nothing to disclose; G. Magnani has nothing to disclose; P. Caroppo has nothing to disclose; S. Prioni has nothing to disclose; C. Villa has nothing to disclose; E. Stefanova has received payment for honoraria for lectures, presentations, speakers bureaus, manuscript writing, or educational events from Roche, Actavis, and Krka, and support for attending meetings and/or travel from Krka, Goodwill Pharma, and Roche; V. Kostic has nothing to disclose; F. Agosta is Associate Editor of NeuroImage: Clinical and the European Journal of Neurology; has received speaker honoraria from Biogen Idec, Bristol Myers Squibb, Eisai, Eli Lilly, GE Healthcare, Neuraxpharm, and Roche; and receives or has received research supports from the Italian Ministry of Health, the Italian Ministry of University and Research, AriSLA (Fondazione Italiana di Ricerca per la SLA), the European Research Council (ERC), the EU Joint Programme – Neurodegenerative Disease Research (JPND), and Foundation Research on Alzheimer Disease (France). M. Filippi is Editor‐in‐Chief of the Journal of Neurology, Associate Editor of Human Brain Mapping, Neurological Sciences, and Radiology; received compensation for consulting services from Almirall, Biogen, Bristol‐Myers Squibb, Eli Lilly, Merck, Novartis, Roche, Sanofi; speaking activities from Amgen, Bayer, Biogen, Bristol‐Myers Squibb, Celgene, Chiesi Italia SpA, Eisai, Eli Lilly, Fujirebio, Genzyme, Janssen, Merck, Neopharmed Gentili, Neuraxpharm, Novartis, Novo Nordisk, Roche, Sanofi, Takeda; participation in Advisory Boards for Alexion, Biogen, Bristol‐Myers Squibb, Eli Lilly, GE Healthcare Ltd, Merck, Neuraxpharm, Novartis, Roche, Sandoz, Sanofi, Takeda; scientific direction of educational events for Biogen, Merck, Roche, Celgene, Bristol‐Myers Squibb, Lilly, Novartis, Sanofi‐Genzyme; he receives research support from Biogen Idec, Merck‐Serono, Novartis, Roche, the Italian Ministry of Health, the Italian Ministry of University and Research, and Fondazione Italiana Sclerosi Multipla.

## OPR‐076

### **1**‐year real‐world use of lecanemab and donanemab in an Italian memory clinic

#### G. Rugarli ^1^; G. Cecchetti^2^; E. Spinelli^1^; A. Ghirelli^1^; S. Pisano^2^; F. Coraglia^1^; E. Canu^2^; V. Castelnovo^2^; E. Sibilla^2^; A. Gilioli^2^; C. Tripodi^1^; F. Freri^2^; A. Bianchi^3^; P. Vezzulli^4^; S. Calloni^4^; A. Falini^5^; A. Samanes Gajate^6^; A. Panzacchi^6^; G. Pepe^6^; C. Ferri^7^; A. Chiti^6^; F. Agosta^1^; M. Filippi^8^

##### ^*1*^ *Center for Alzheimer's disease and related disorders (CARD), Neurology Unit, and Neuroimaging Research Unit, Division of Neuroscience, IRCCS San Raffaele Scientific Institute, Milan, Italy; Vita‐Salute San Raffaele University, Milan, Italy;* ^*2*^ *Center for Alzheimer's disease and related disorders (CARD), Neurology Unit, and Neuroimaging Research Unit, Division of Neuroscience, IRCCS San Raffaele Scientific Institute, Milan, Italy;* ^*3*^ *Neuroimaging Research Unit, Division of Neuroscience, RCCS San Raffaele Scientific Institute, Milan, Italy;* ^*4*^ *Neuroradiology Service, IRCCS San Raffaele Scientific Institute, Milan, Italy;* ^*5*^ *Neuroradiology Service, IRCCS San Raffaele Scientific Institute, Milan, Italy; Vita‐Salute San Raffaele University, Milan, Italy;* ^*6*^ *Nuclear Medicine Service, IRCCS San Raffaele Scientific Institute, Milan, Italy;* ^*7*^ *Pharmacy Unit, IRCCS San Raffaele Scientific Institute, Milan, Italy;* ^*8*^ *CARD, Neurology Unit, Neurorehabilitation Unit, Neurophysiology Service, and Neuroimaging Research Unit, Division of Neuroscience, IRCCS San Raffaele Scientific Institute, Milan, Italy; Vita‐Salute San Raffaele University, Milan, Italy*

**Background and Aims:** To report feasibility, safety, and early biological outcomes of lecanemab and donanemab therapy for early Alzheimer's disease (AD) in a real‐world tertiary setting.

**Methods:** This observational study included consecutive patients treated with anti‐amyloid antibodies at the San Raffaele Memory Center (Milan, Italy) between September 2024 and October 2025. All patients fulfilled clinical and biomarker criteria for mild cognitive impairment or mild dementia due to AD. Lecanemab (10 mg/kg biweekly) and donanemab (350–1400 mg monthly titration) were administered under EMA‐aligned protocols with standardized MRI monitoring. Safety outcomes included amyloid‐related imaging abnormalities (ARIA) and infusion‐related reactions (IRR). Biological effects were assessed using amyloid‐PET Centiloid quantification, plasma biomarkers, and cognitive measures at 6 months.

**Results:** Twenty‐nine treatment courses were delivered (9 lecanemab, 20 donanemab). ARIA‐E occurred in 2 (10%) and ARIA‐H in 3 (15%) donanemab‐treated patients, all resolving with corticosteroids. One mild asymptomatic ARIA‐H (11%) was observed with lecanemab. IRRs occurred in 6 patients (21%), all mild‐to‐moderate. At 6 months, cortical amyloid burden decreased by −61 ± 30 Centiloids (*p* < 0.001); 75% of donanemab‐treated patients achieved PET negativity (<11 CL). Plasma GFAP (−20 ± 26 pg/mL) and pTau181 (−0.8 ± 0.9 pg/mL) declined, while NfL showed a mild increase (+4.4 ± 4.7 pg/mL). Cognitive scores (MMSE, CDR‐SB) remained stable. No discontinuations occurred for safety or logistical reasons.

**Conclusion:** Anti‐amyloid antibodies can be safely implemented in real‐world tertiary centers. Amyloid clearance and plasma biomarker dynamics mirror pivotal trials, supporting the feasibility of disease‐modifying therapy for AD in structured clinical practice.

**Disclosure:** Giulia Rugarli has nothing to disclose; Giordano Cecchetti received speaker honoraria from Neopharmed Gentili; Edoardo Gioele Spinelli has nothing to disclose; Alma Ghirelli has nothing to disclose; Stefano Pisano has nothing to disclose; F Coraglia has nothing to disclose; Elisa Canu receives research support from the Italian Ministry of Health; V Castelnovo has nothing to disclose; E Sibilla has nothing to disclose; A Gilioli has nothing to disclose; C Tripodi has nothing to disclose; F Freri has nothing to disclose; A Bianchi has nothing to disclose; P Vezzulli has nothing to disclose; S Calloni has nothing to disclose; A Falini has nothing to disclose; AM Samanes Gajate has nothing to disclose. A. Panzacchi has nothing to disclose. G. Pepe has nothing to disclose. C. Ferri has nothing to disclose. A. Chiti reports consulting or advisory role for Blue Earth Diagnostics, Telix Pharmaceuticals, InnovaRadi Therapeutic, and General Electric Healthcare; and Speaker's Bureaus for Bracco Diagnostics, General Electric Healthcare, Novartis, Telix Pharmaceuticals, and United Imaging; he is Editor in Chief of The EANM Journal. F Agosta is Associate Editor of NeuroImage: Clinical and the European Journal of Neurology; has received speaker honoraria from Biogen Idec, Bristol Myers Squibb, Eisai, Eli Lilly, GE Healthcare, Neuraxpharm, and Roche; and receives or has received research supports from the Italian Ministry of Health, the Italian Ministry of University and Research, AriSLA (Fondazione Italiana di Ricerca per la SLA), the European Research Council (ERC), the EU Joint Programme – Neurodegenerative Disease Research (JPND), and Foundation Research on Alzheimer Disease (France). M Filippi is Editor‐in‐Chief of the Journal of Neurology, Associate Editor of Human Brain Mapping, Neurological Sciences, and Radiology; received compensation for consulting services from Almirall, Biogen, Bristol‐Myers Squibb, Eli Lilly, Merck, Novartis, Roche, Sanofi; speaking activities from Amgen, Bayer, Biogen, Bristol‐Myers Squibb, Celgene, Chiesi Italia SpA, Eisai, Eli Lilly, Fujirebio, Genzyme, Janssen, Merck, Neopharmed Gentili, Neuraxpharm, Novartis, Novo Nordisk, Roche, Sanofi, Takeda; participation in Advisory Boards for Alexion, Biogen, Bristol‐Myers Squibb, Eli Lilly, GE Healthcare Ltd, Merck, Neuraxpharm, Novartis, Roche, Sandoz, Sanofi, Takeda; scientific direction of educational events for Biogen, Merck, Roche, Celgene, Bristol‐Myers Squibb, Lilly, Novartis, Sanofi‐Genzyme; he receives research support from Biogen Idec, Merck‐Serono, Novartis, Roche, the Italian Ministry of Health, the Italian Ministry of University and Research, and Fondazione Italiana Sclerosi Multipla.

## OPR‐077

### Frailty is associated with structural and metabolic brain changes in cognitively unimpaired adults

#### M. Toccaceli Blasi ^1^; J. Contador^2^; G. Bruno^1^; A. González‐Escalante^2^; I. Cumplido^2^; O. Grau‐Rivera^2^; D. Vállez García^2^; J. Jisper^2^; G. Salvadò Blasco^2^; M. Canevelli^1^; M. Suárez‐Calvet^2^

##### ^*1*^ *Department of Human Neuroscience, “Sapienza” University, Rome, Italy;* ^*2*^ *Barcelonaβeta Brain Research Center (BBRC), Pasqual Maragall Foundation, Barcelona, Spain*

**Background and Aims:** Frailty is a multidimensional syndrome that entails an increased risk of dementia. Characterizing early frailty‐related brain changes in cognitively unimpaired (CU) individuals may help to clarify its role in subsequent cognitive decline, particularly beyond Alzheimer's pathology.

**Methods:** We investigated the relationship between frailty and 3T‐MRI, and [18F] FDG PET biomarkers in CU middle‐aged adults from the ALFA+ cohort with available cerebrospinal fluid (CSF) Alzheimer's disease (AD) biomarkers. Frailty was assessed using a 35‐item Frailty Index (FI), and its relationship with predicted brain age, global and regional cortical thickness (CTh), brain volume, white matter hyperintensities (WMH), and cerebral hypometabolism was investigated using linear regression models.

**Results:** Out of the 418 included participants, 57 (13.64%) were classified as frail. Frailty was associated with older predicted brain age, decreased mean, fronto‐temporal and Jack's signature CTh, and hippocampal volume, as well as mean, parieto‐temporal and posterior cingulate [18F] FDG uptake. Frailty was also associated with augmented white matter hyperintensities (WMH), especially in fronto‐parietal and basal ganglia regions. These associations remained significant also after adjusting for CSF AB42/40 levels.

**Figure:**
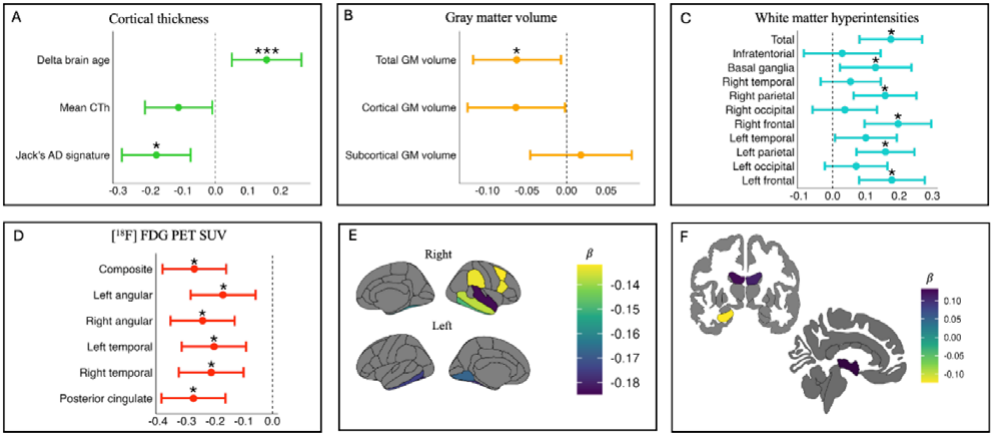
**FIGURE 1** Forest plots of linear regression models, corrected by age, sex, CSF AB42/40, and total intracranial volume if necessary, exploring the impact of frailty on cortical thickness.

**Conclusion:** Independent of amyloid pathology, frailty was associated with atrophy, vascular damage, and hypometabolism in AD and aging regions in CU adults. These findings highlight frailty as an early contributor to brain vulnerability, potentially preceding dementia.

**Disclosure:** Nothing to disclose.

## OPR‐078

### MRI‐based atrophy subtypes in a young memory clinic cohort: Associations with clinical and biomarker profiles

#### A. Zilioli ^1^; R. Mohanty^2^; A. Rosenberg^2^; A. Matton^2^; T. Granberg^2^; G. Hagman^2^; M. Spallazzi^1^; D. Ferreira^2^; M. Kivipelto^2^; E. Westman^2^

##### ^*1*^ *Department of Neurology, University‐Hospital of Parma, Parma, Italy;* ^*2*^ *Division of Clinical Geriatrics, Center for Alzheimer Research, Department of Neurobiology, Care Sciences and Society, Karolinska Institutet, Stockholm, Sweden*

**Background and Aims:** MRI‐based brain atrophy subtypes are increasingly recognized in Alzheimer's disease (AD) dementia, but their relevance across the real‐world memory clinic spectrum remains unclear. We aimed to identify atrophy subtypes in a young memory clinic cohort and examine their associations with demographics, cerebrospinal fluid (CSF) biomarkers, and cerebrovascular burden.

**Methods:** We included consecutive patients (subjective cognitive impairment [SCI] to dementia) evaluated at the Karolinska Memory Clinic (2018–2023) with available 3T MRI. Subtypes were defined using FreeSurfer volumetric measures and a validated algorithm combining categorical classification (typical, limbic predominant, cortical predominant, minimal atrophy) with continuous indices of typicality and severity. Cognitive, APOE ε4, CSF biomarkers (Aβ42, Aβ42/40, p‐tau181, total tau, neurofilament light [NFL]), and cerebrovascular burden were compared across subtypes. Analyses were replicated in Aβ‐positive and anti‐Aβ‐therapy‐eligible patients.

**Results:** Among 809 patients (median age 60 years; 56.1% female), 38.2% had SCI, 44.4% MCI, and 17.4% dementia. Limbic predominant and typical subtypes showed higher male prevalence, APOE ε4 frequency, cerebrovascular burden, poorer memory, and greater Aβ positivity (all *p* ≤ 0.02). The cortical predominant subtype was more frequent in females, whereas minimal atrophy was associated with milder cognitive impairment and higher depressive symptoms. In Aβ‐positive patients (*n* = 231), typical and limbic subtypes showed higher p‐tau181 and NFL levels and lower Aβ42/40 ratios (all *p* < 0.05). Results were consistent across continuous atrophy measures and in patients eligible for anti‐Aβ therapy (*n* = 89).

**Figure:**
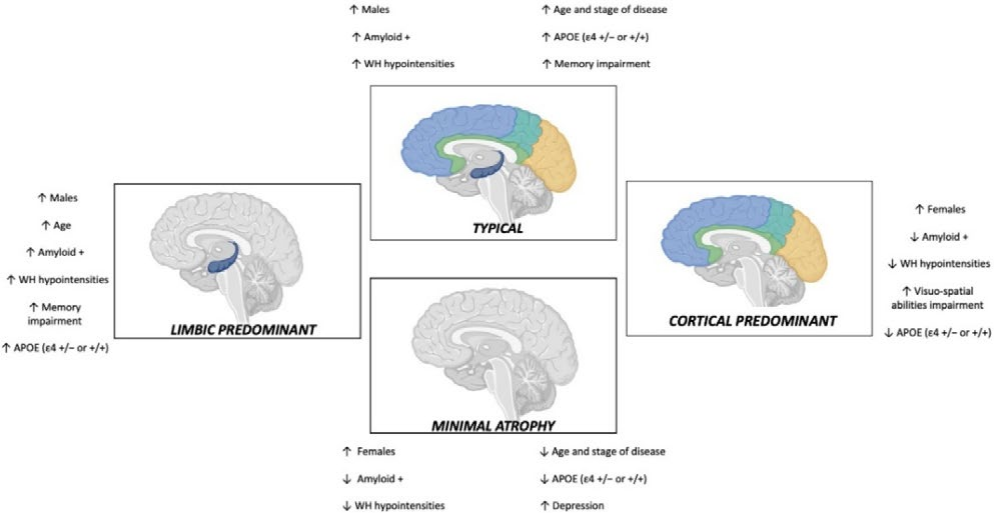
**FIGURE 1** Summary of demographic, cognitive assessment, CSF, and vascular profiles among the MRI‐based atrophy subtypes.

**Figure:**
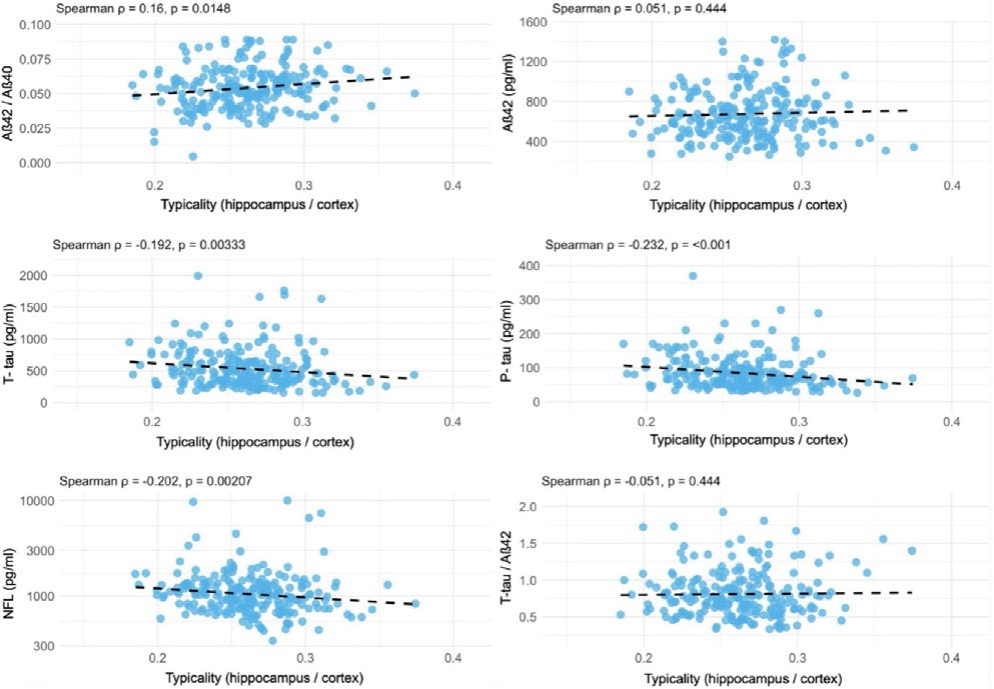
**FIGURE 2** Scatterplots of correlation between typicality (hippocampus/cortex ratio) and CSF biomarkers in the Aβ‐positive cohort.

**Figure:**
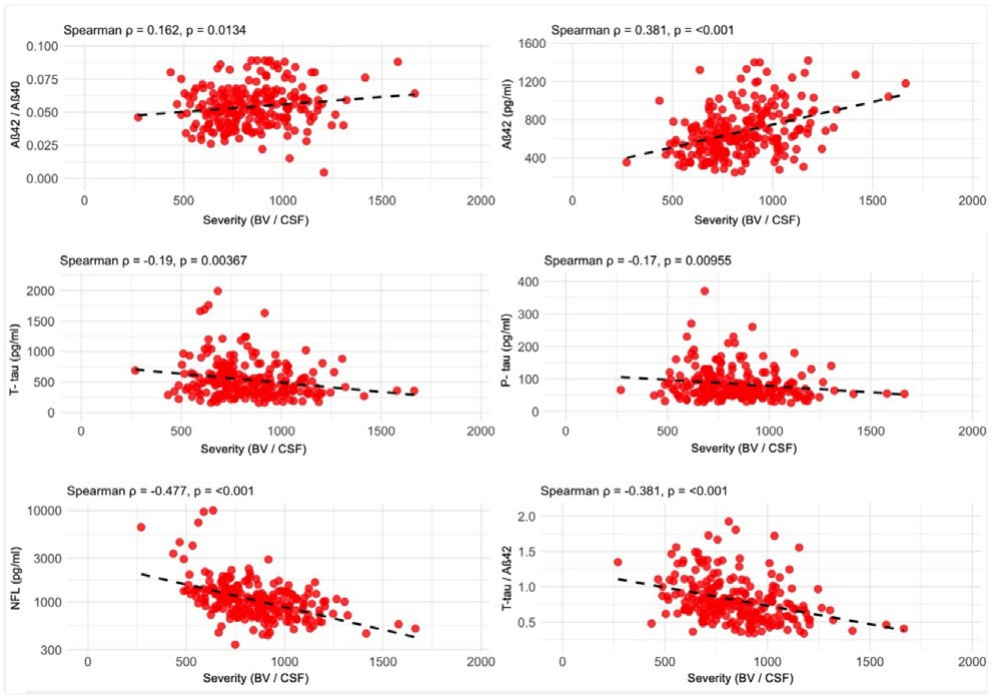
**FIGURE 3** Scatterplots of correlation between severity (brain volume/CSF ratio) and CSF biomarkers in the Aβ‐positive cohort.

**Conclusion:** MRI‐based atrophy subtypes display distinct clinical and biomarker profiles across the memory clinic spectrum, supporting their diagnostic value and relevance for biologically targeted AD trials.

**Disclosure:** Nothing to disclose.

## Neuroimmunology 1

## OPR‐079

### Clinical and prognostic features of de novo central nervous system non‐MS demyelinating disorders following immune checkpoint inhibitor therapy

#### A. Malvaso ^1^; S. Masciocchi^2^; K. Bihan^3^; A. Dinoto^4^; A. Farina^5^; A. Picca^6^; C. Izquierdo Gracia^7^; L. Martín‐Aguilar^8^; J. Bruna^9^; V. Damato^10^; E. Sechi^11^; S. Rossi^12^; N. De Rossi^13^; E. Ariño^14^; A. Cobo‐Calvo^14^; S. Mariotto^15^; B. Joubert^5^; E. Marchioni^16^; A. Vogrig^17^; M. Gastaldi^2^

##### ^*1*^ *Department of Brain and Behavioral Sciences, University of Pavia; Neuroimmunology Laboratory and Neuroimmunology Research Section, IRCCS Mondino Foundation, Pavia, Italy;* ^*2*^ *Neuroimmunology Laboratory and Neuroimmunology Research Section, IRCCS Mondino Foundation, Pavia, Italy;* ^*3*^ *Department of Pharmacology, Institut National de la Santé et de la Recherche Médicale (INSERM), Assistance Publique – Hôpitaux de Paris (AP‐HP), Clinical Investigation Center (CIC‐1901), Sorbonne Université, Pitié‐Salpêtrière Hospital, Paris, France;* ^*4*^ *Neuroimmunology and Neuromuscular Disease Unit, Fondazione IRCCS Istituto Neurologico Carlo Besta, Milan, Italy;* ^*5*^ *French Reference Centre on Paraneoplastic Neurological Syndromes and Autoimmune Encephalitis, Hospices Civils de Lyon, Hôpital Neurologique, Bron Cedex, France;* ^*6*^ *Neuro‐Oncology Service, AP‐HP, Pitié‐Salpêtrière Hospital and Sorbonne Université, Paris, France;* ^*7*^ *Multiple Sclerosis Unit, Department of Neurosciences, Hospital Universitari Germans Trias i Pujol, Badalona, Spain;* ^*8*^ *Neuromuscular Diseases Unit, Department of Neurology, Hospital de la Santa Creu i Sant Pau, Barcelona, Spain;* ^*9*^ *Neuro‐Oncology Unit, Hospital Universitari de Bellvitge‐IDIBELL, L'Hospitalet de Llobregat, Barcelona, Spain;* ^*10*^ *Department of Neurosciences, Psychology, Drug Research and Child Health (NEUROFARBA), University of Florence, Florence, Italy;* ^*11*^ *Neurology Unit, University Hospital of Sassari, Sassari, Italy,* ^*12*^ *IRCCS Istituto delle Scienze Neurologiche di Bologna (S.R., R.R., M. Guarino), Italy;* ^*13*^ *ASST‐Spedali Civili di Brescia, Montichiari, Italy;* ^*14*^ *Neurology Department, Centre d'Esclerosi Múltiple de Catalunya (Cemcat), Hospital Universitari Vall d'Hebron, Vall d'Hebron Institut de Recerca, Barcelona, Spain;* ^*15*^ *Neurology Unit, Department of Neurosciences, Biomedicine and Movement Sciences, University of Verona, Verona, Italy;* ^*16*^ *Neuroncology and Neuroinflammation Unit, IRCCS Mondino Foundation, Pavia, Italy;* ^*17*^ *Department of Medicine (DMED), University of Udine, Udine, Italy; Clinical Neurology, Azienda Sanitaria Universitaria Friuli Centrale (ASUFC), Udine, Italy*

**Background and Aims:** Immune‐related adverse events (irAEs) can occur after the administration of immune‐checkpoint inhibitors (ICI). De novo demyelinating irAEs (DEMirAEs) are rare, poorly characterized and often challenging. We aimed to define the demographic, clinical, therapeutic and prognostic features of DEMirAEs.

**Methods:** We conducted a multicentric retrospective study including patients with DEMirAEs evaluated between 2011 and 2024 in nine European neurology units. Additional cases were identified through a systematic literature review according to PRISMA guidelines. Statistical analyses were performed using R software version 4.0.3.

**Results:** We identified 80 patients with DEM‐irAEs. Median age was 59 years (range: 9–84), with male predominance (52/80, 65%). DEM‐irAEs developed after a median of 18 weeks (range: 2–178) following ICI initiation (anti‐PD1 *n* = 49, 61%; anti‐PD‐L1 *n* = 4, 5%; anti‐CTLA4 *n* = 5, 6%; anti‐PD1 plus anti‐CTLA4 *n* = 22, 28%). Clinical manifestations included optic neuritis (*n* = 17, 8 bilateral), myelitis (*n* = 29, including 24 LETM), acute disseminated encephalomyelitis (*n* = 6), atypical DEM‐irAEs (*n* = 7), and overlapping syndromes (*n* = 21). CSF‐restricted oligoclonal bands (12/50, 24%) and glial autoantibodies were uncommon (11/80, 14%). Immunotherapy was administered in 74/80 (93%) of patients, mainly steroids (*n* = 61), IVIg (*n* = 16), plasmapheresis (*n* = 23), rituximab (*n* = 8), and cyclophosphamide (*n* = 8). Outcomes were complete response in 15 patients (24%), partial response in 25 (40%), and no response in 22 (35%). Compared with AQP4‐NMOSD (*n* = 42) and MOGAD (*n* = 22), DEM‐irAEs showed greater attack severity and worse outcomes (mRS > 2; *p* < 0.01).

**Conclusion:** DEMirAEs represent a rare and heterogeneous complication of ICI treatment. Long‐term immunosuppression should be considered in selected cases, as approximately one‐quarter of patients relapse, and outcomes appear worse than in AQP4‐NMOSD and MOGAD.

**Disclosure:** Nothing to disclose.

## OPR‐080

### Circumventricular organ permeability to NMO IgG allows AQP4 targeting in the mouse brain

#### C. Peynet ^1^; S. Joly^1^; A. Chan^1^; A. Ben‐Zvi^2^; V. Pernet^1^

##### ^*1*^ *Neurology, Inselspital, Bern, Switzerland;* ^*2*^ *Developmental Biology and Cancer Research, The Institute for Medical Research‐Israel‐Canada, Jerusalem, Israel*

**Background and Aims:** The penetration of circulating aquaporin‐4 (AQP4) IgG into the CNS is a major event in neuromyelitis optica (NMO)‐induced astrocytopathy. After intravenous injection in rodents, recombinant AQP4 IgG predominantly accumulates in circumventricular organs (CVOs), such as the median eminence. Physiologically, protein passage through the fenestrated blood vessels of CVOs importantly contributes to brain‐periphery communication. The aim of the present study was to determine whether the median eminence is an entry site for AQP4 IgG in the mouse CNS, leading to AQP4 targeting.

**Methods:** AQP4 expression was examined in the brain of C57BL/6JRj female mice by immunofluorescence on coronal sections. The biodistribution of recombinant human anti‐AQP4 antibody (rAb‐53) was observed 24 h after intravenous injection (7.5 mg/kg body weight). Vascular endothelial cells and tanycytes were labeled using podocalyxin and vimentin, respectively.

**Results:** AQP4 was present in perivascular astrocytic endfeet and in ependymal cells lining the 3rd ventricle, but not in tanycyte cell bodies which occupy a more ventral position. After intravenous injection, a high level of rAb‐53 was detected within the parenchyma and around podocalyxin‐positive blood vessels of the median eminence. Ependymal cells, but not tanycyte cell bodies, were also positive for rAb‐53. High‐resolution microscopy revealed perivascular colocalization of AQP4 and rAb‐53, thus suggesting antigen‐antibody interaction.

**Conclusion:** Our data demonstrate that AQP4 IgG reaches AQP4 in astrocytes and ependymal cells through the leaky vasculature of the median eminence in the mouse brain, suggesting that CVOs are potential entry sites for pathogenic antibodies in NMO.

**Disclosure:** This project has been supported by the Swiss National Science Foundation (SNSF project grant #10003453 to VP and AC). Andrew Chan has served on advisory boards for, and received funding for travel or speaker honoraria from, Actelion‐Janssen, Alexion, Almirall, Biogen, BMS, Celgene, Dresden International Uni, Horizon, Merck, Mylan (Viatris), Novartis, Roche, Sanofi‐Genzyme, Teva, UCB, Uni Leipzig, Wiley, all for hospital research funds; and research support from Biogen, CSL Behring, Genzyme, Roche, UCB and European Union and Swiss National Research Foundation. A. Chan is associate editor of the European Journal of Neurology and serves on the editorial board for Clinical and Translational Neuroscience and as topic editor for the Journal of International Medical Research.

## OPR‐081

### GAD65 epitope predictive for Stiff‐person syndrome

#### E. Leisgang ^1^; I. Talucci^2^; S. Seefried^1^; A. Wiessler^3^; C. Kurth^1^; C. Villmann^3^; H. Maric^4^; C. Sommer^1^

##### ^*1*^ *Department of Neurology, University Hospital Würzburg, Würzburg, Germany;* ^*2*^ *Northwestern University, Evanston, USA;* ^*3*^ *Institute for Clinical Neurobiology, University of Würzburg, Würzburg, Germany;* ^*4*^ *Rudolf Virchow Center for Integrative and Translational Bioimaging, University of Würzburg, Würzburg, Germany*

**Background and Aims:** Stiff‐person‐syndrome (SPS) is a rare but treatable neurological autoimmune disease associated with GAD65‐autoantibodies (aAb). However, GAD65aAb may also be associated with other diseases, like epilepsy or ataxia. We therefore aimed at determining SPS‐specific GAD65 epitopes.

**Methods:** Epitope binding was screened in sera (*n* = 46) and cerebrospinal fluid (*n* = 4) from a SPS patient cohort (*n* = 30) via microarray binding assay including full positional scans. Controls included healthy controls (*n* = 7), a GAD65‐aAb‐positive diabetes cohort (*n* = 10) and a GlyRα1‐aAb SPSD cohort (*n* = 24).

**Results:** 80% (24/30 patients) showed aAb binding to at least one out of five epitopes (E1–E5) and a single dominant epitope (E1) was recognized in 56% of all SPS samples. For 20% (6/30 patients) no aAb binding epitopes could be determined. In 14 patients epitopes were analysed before and after plasmapheresis (PE). Here we found a baseline polyclonal antibody response that disappeared after PE, only the response to E1 remained. No correlation between epitope profile and clinical phenotype was visible within the SPS spectrum. Statistical analysis validated E1 as the SPS‐specific GAD65 epitope, not recognized by any of the tested controls (*p* < 0.0001).

**Figure:**
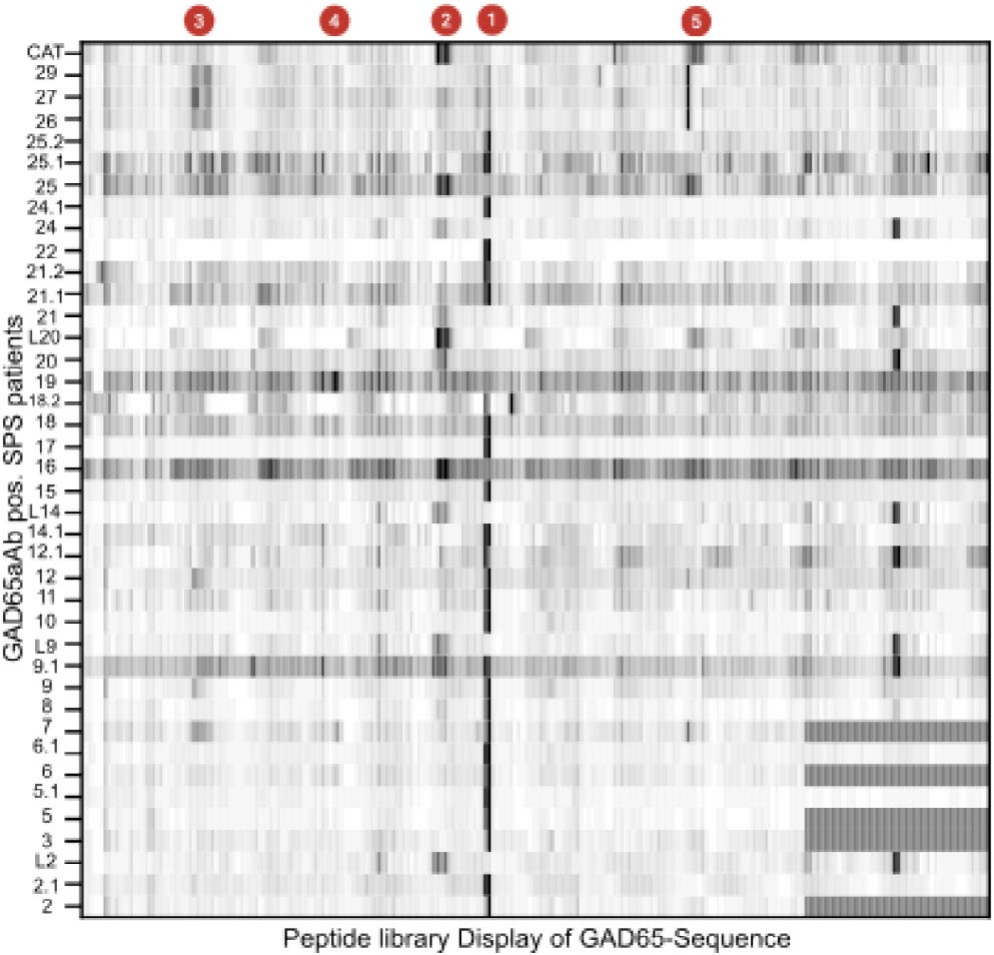
**FIGURE 1** Epitope mapping in Stiff person syndrome cohort reveals five distinct epitopes. Heatmap representing overview of initial screening results for GAD65 epitopes with microarray binding assay including baseline, follow up and CSF samples.

**Figure:**
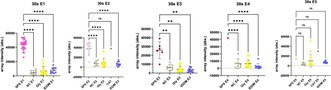
**FIGURE 2** Correlation among disease and GAD65 epitope E1, E2, E3, E4 and E5. Horizontal line refers to median, error bars indicate interquartile range. Prior to conducting the one‐way ANOVA, the assumption of homogeneity of variances was tested.

**Conclusion:** Epitope screening, mapping and characterization defined five distinct GAD65 epitopes shared within SPS patients. Amongst all, E1 stands out as the strongest predictor for SPS due to its prevalence and consistency. Epitopes in SPS GAD65‐aAbs and diabetes GAD65‐aAbs were clearly distinct. This knowledge has the potential for more precise SPS diagnostics and broad implications for SPS pathophysiological research.

**Disclosure:** Nothing to disclose.

## OPR‐082

### Long‐term cognitive recovery and disease‐specific patient‐reported outcomes of patients with ant‐LGI1 encephalitis

#### J. Brenner ^1^; A. Tolido^1^; Y. Crijnen^1^; C. Ruhe^1^; T. Brand^1^; S. Olijslagers^2^; M. Mandarakas^3^; E. van den Berg^1^; M. Titulaer^1^

##### ^*1*^ *Erasmus University Medical Center Rotterdam, The Netherlands;* ^*2*^ *Catharina Hospital Eindhoven, The Netherlands;* ^*3*^ *UNSW Medicine and Health Sydney*

**Background and Aims:** Studies on the long‐term cognitive abilities and psychosocial outcomes of individuals with anti‐LGI1 encephalitis are scarce. Recently, a disease‐specific Patient‐Reported Outcomes Score for (autoimmune) Encephalitis (PROSE; score 0–100) was developed. We assessed cognitive and psychosocial recovery in a large national cohort, including the first patient‐data using PROSE.

**Methods:** All adults diagnosed with anti‐LGI1 encephalitis in the Netherlands were invited to participate, provided they could self‐report and participate in cognitive testing. Patients were evaluated cross‐sectionally, and those within 1.5 years of diagnosis were also followed prospectively. Functional (mRS, CASE), cognitive (comprehensive test‐battery covering major cognitive domains) and self‐reported outcomes (PROSE, SF‐36, EQ‐5D‐5L, WHO‐5, WHO‐DAS‐II, FSS, BDI, HADS) were assessed at various disease stages.

**Results:** We included 106 patients with anti‐LGI1 encephalitis (mean age 68 years, 33% female). Two years after diagnosis, 50% of patients (*n* = 37/74) had fully recovered and returned to their regular life, while 40% had significant cognitive impairments (<−1.5 SD below norm) in at least one domain, most commonly verbal memory (average *z*‐score >2years −1.12, SD = 0.90). Cognitive recovery plateaued around 24 months post‐treatment, whereas psychosocial recovery often continued for at least 3 years after treatment (PROSE mean score <1 year: 30, >3 years: 26).

**Figure:**
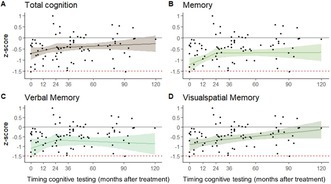
**FIGURE 1** Cognitive outcomes in relation to time after diagnosis and treatment.

**Figure:**
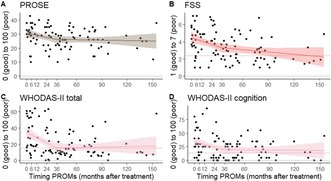
**FIGURE 2** Self‐reported psychosocial outcomes in relation to time after diagnosis and treatment, including the first patient‐data on the PROSE.

**Conclusion:** Anti‐LGI1 is a serious neurological condition. Around 50% fully recovers but many experience lasting cognitive and psychosocial difficulties, and would benefit from long‐term monitoring and targeted rehabilitation. We outline the recovery trajectory of anti‐LGI1 encephalitis and present the first patient data on the disease‐specific PROSE.

**Disclosure:** J. Brenner and M.J. Titulaer, on behalf of the Erasmus University Medical Centre, hold intellectual property rights for the PROSE.

## OPR‐083

### Prolonged vegetative state in anti‐NMDA receptor encephalitis: A retrospective international cohort study of complications, outcomes, and implications

#### M. Guasp ^1^; M. Simabukuro^2^; G. Muñoz‐Sánchez^3^; J. Brenner^4^; K. Wurdack^5^; K. Wang^6^; P. Lalive^7^; G. Olivé‐Cirera^8^; J. Yeung^9^; K. Wandinger^10^; S. Macher^11^; G. Di Liberto^12^; E. Lancaster^13^; T. Casadevall^14^; M. Martínez‐González^15^; J. Fernández‐Carrasco^16^; M. Shibata^17^; E. Fukao^18^; T. Mishima^19^; H. Nakagawa^20^; T. Takizawa^21^; S. Shimohama^21^; Y. Izawa^21^; S. Neshige^22^; R. Borràs^23^; E. Aguilar^8^; L. Marmolejo^8^; J. Planagumà^8^; A. Heller^24^; M. Tojima^25^; A. Ikeda^25^; D. Wu^6^; M. Spatola^8^; Y. Blanco^1^; R. Du Pasquier^12^; K. Sühs^26^; F. Leypoldt^10^; R. Höftberger^27^; C. Finke^5^; M. Titulaer^4^; T. Armangué^8^; F. Graus^8^; T. Iizuka^28^; J. Dalmau^8^

##### ^*1*^ *Neuroimmunology Unit, Department of Neurology, Hospital Clínic de Barcelona, University of Barcelona, Spain;* ^*2*^ *Division of Neurology, Hospital das Clinicas (HCFMUSP), Faculty of Medicine, University of São Paulo, São Paulo, Brazil;* ^*3*^ *Department of Immunology, Hospital Clínic de Barcelona, University of Barcelona, Spain;* ^*4*^ *Department of Neurology, Erasmus University Medical Center Rotterdam, The Netherlands;* ^*5*^ *Department of Neurology, Charité Universitätsmedizin Berlin, Berlin, Germany;* ^*6*^ *Department of Neurology, the First Affiliated Hospital, School of Medicine, Zhejiang University, Hangzhou, China;* ^*7*^ *Department of Clinical Neurosciences, Division of Neurology; and Department of Pathology and Immunology, Faculty of Medicine, University Hospital of Geneva, Geneva, Switzerland;* ^*8*^ *Neuroimmunology Program, Fundació de Recerca Clínic Barcelona‐Institut d'Investigacions Biomèdiques August Pi i Sunyer (FRCB‐IDIBAPS), and CaixaResearch Institute (CRI), Barcelona, Spain;* ^*9*^ *Department of Medicine, Alice Ho Miu Ling Nethersole Hospital, Hong Kong;* ^*10*^ *Institute of Clinical Chemistry, University Hospital Schleswig‐Holstein Kiel/Lübeck, Germany;* ^*11*^ *Department of Neurology, Medical University of Vienna, Austria;* ^*12*^ *Service of Neurology, Department of Clinical Neurosciences, Lausanne University Hospital (CHUV) and University of Lausanne, Switzerland;* ^*13*^ *Department of Neurology, Perelman School of Medicine, University of Pennsylvania, Philadelphia, USA;* ^*14*^ *Department of Neurology, Hospital Comarcal Sant Jaume de Calella, Spain;* ^*15*^ *Pediatric Neurology Unit, Cruces University Hospital, Biobizkaia Health Research Institute, Barakaldo, Spain;* ^*16*^ *Department of Neurology, Hospital Carlos Van Buren de Valparaíso, Universidad de Valparaíso, Chile;* ^*17*^ *Department of Neurology, Tokyo Dental College Ichikawa General Hospital, Ichikawa, Japan;* ^*18*^ *Department of Neurology, Showa General Hospital, Tokyo, Japan;* ^*19*^ *Division of Neurology, Department of Internal Medicine, Sakura Medical Center, Toho University, Sakura, Japan;* ^*20*^ *Neurology Department, Kagoshima City Medical Association Hospital, Kagoshima, Japan;* ^*21*^ *Department of Neurology, Keio University School of Medicine, Tokyo, Japan;* ^*22*^ *Department of Clinical Neuroscience and Therapeutics, Hiroshima University Graduate School of Biomedical and Health Sciences, Hiroshima, Japan;* ^*23*^ *Medical Statistics, Institut d'Investigacions Biomèdiques August Pi i Sunyer (IDIBAPS), Barcelona, Spain;* ^*24*^ *Neuroimmunology Division, Children's Hospital of Pittsburgh/University of Pittsburgh Medical Center, USA;* ^*25*^ *Department of Neurology, Kyoto University Hospital, Kyoto, Japan;* ^*26*^ *Department of Neurology, Hannover Medical School, Germany;* ^*27*^ *Division of Neuropathology and Neurochemistry, Department of Neurology and Comprehensive Center for Clinical Neurosciences and Mental Health, Medical University of Vienna, Austria;* ^*28*^ *Department of Neurology, Kitasato University School of Medicine, Sagamihara, Japan*

**Background and Aims:** In anti‐NMDA receptor encephalitis (NMDARe) delayed and slow improvement complicates assessment of treatment refractoriness, creating a larger knowledge gap in prolonged impaired consciousness. We aimed to assess these features and outcomes in patients with prolonged vegetative state.

**Methods:** Retrospective cohort study of NMDARe patients who remained in vegetative state for ≥9 months. Outcomes were: death; achieving command‐following, mRS = 2, or recovery (mRS = 0 with return to premorbid activities).

**Results:** Forty‐five patients were identified (38 female; median age 22 [IQR 19–31]). All had impaired consciousness 9 (5–16) days after symptom onset. All received first‐line immunotherapy; 41 (91%) second‐line, and 18 (40%) third‐line. Twenty‐one (47%) had ovarian teratomas. Median durations were: vegetative state 399 days (307–698), ICU stay 275 days (189–354), and hospitalization 474 days (349–715). Thirteen (28%) patients were resuscitated from dysautonomic cardiac arrest. After a median of 5 years (2.5–6.8), 15 (33%) recovered, 13 (29%) substantially improved (mRS ≤ 2), 11 (24%) had mRS = 3–5, and 6 (13%) died. Five began command‐following 11 months (1.5–21) after last immunotherapy. Estimated cumulative incidences at 5 and 10 years were 66% and 76% for achieving mRS = 2, and 32% and 54%, respectively, for recovery. Teratomas were associated with lower probability of achieving mRS = 2, whereas older age and higher NEOS2 score were associated with increased mortality.

**Conclusion:** In NMDARe, recovery from prolonged impaired consciousness is more common than expected. Early assessment of treatment response or refractoriness may underestimate delayed improvement, prolonged therapy needs, or spontaneous recovery. Futility decisions should be individualized with multidisciplinary input after extended follow‐up.

**Disclosure:** Dr. Dalmau holds patents licensed to Euroimmun for the use of NMDA, GABAB receptor, GABAA receptor, DPPX and IgLON5 as autoantibody tests, for which he receives royalties. Dr. Graus holds a patent licensed to Euroimmun for the use of IgLON5 in an autoantibody test, for which he receives royalties. He receives honoraria from MedLink Neurology for his role as associate editor. Dr. Brenner and Dr. Titulaer hold a copyright, on behalf of Erasmus MC, for the patient‐reported outcome measure PROSE. Dr. Brenner is supported by the AEA Seed Grant 2024. The Medical University of Vienna, Austria (employer of Dr. Höftberger) receives payment for antibody assays and for antibody validation experiments organized by Euroimmun (Lübeck, Germany). The rest of the authors (MG, MMS, GMS, KW, KW, PHL, GOC, JY, KPW, SM, GDL, EL, TC, MJMG, JPFC, MS, EF, TM, HN, TT, SS, YI, SN, RB, EA, LM, JP, AH, MT, AI, DW, MS, YB, RDP, KWS, FL, CF, TA, TI) declare no conflicts of interest related to this work.

## Muscle and Neuromuscular Junction Disorder

## OPR‐084

### Investigating intravenous efgartigimod in juvenile generalized myasthenia gravis: Results from the ADAPT JR study

#### A. Kostera‐Pruszczyk ^1^; A. N. Schwaede^2^; N. L. Kuntz^2^; S. Ramdas^3^; A. Bogatyreva^4^; J. Giacobbe^4^; F. Menezes^4^; L. Lan^4^; J. Noukens^5^; T. van Bragt^5^; E. Harmen Niks^6^

##### ^*1*^ *Department of Neurology, Medical University of Warsaw, Warsaw, Poland;* ^*2*^ *Division of Neurology, Department of Pediatrics, Ann & Robert H. Lurie Children's Hospital of Chicago, Northwestern University Feinberg School of Medicine, Chicago, USA;* ^*3*^ *Department of Paediatric Neurology, John Radcliffe Hospital, Oxford, UK; MDUK Oxford Neuromuscular Centre, Department of Paediatrics, University of Oxford, Oxford, UK;* ^*4*^ *argenx, Ghent, Belgium;* ^*5*^ *Curare Consulting BV, Liempde, The Netherlands;* ^*6*^ *Department of Neurology, Leiden University Medical Center, Leiden, The Netherlands*

**Background and Aims:** Efgartigimod is a human immunoglobulin G1 (IgG1) antibody Fc fragment that blocks the neonatal Fc receptor. Phase 3 trials (ADAPT/ADAPT+) demonstrated that intravenous (IV) efgartigimod is efficacious and well tolerated in adults with generalized myasthenia gravis (gMG). However, there remains an unmet need for treatments in patients with juvenile gMG. We present interim results from a Phase 2/3 trial assessing efgartigimod IV in juvenile participants with anti‐acetylcholine receptor antibody–positive (AChR‐Ab+) gMG (ADAPT JR; NCT04833894).

**Methods:** ADAPT JR will ultimately recruit ≥12 participants (aged 2–17 years), using a staggered design starting with an adolescent cohort (aged 12–17 years). The study comprises a single‐dose confirmatory Part A (≥8 weeks) and a multiple‐dose treatment‐response confirmatory Part B (≥18 weeks).

**Results:** Eleven adolescent participants were enrolled as of February 2025. Efgartigimod treatment decreased both total IgG and AChR‐Ab levels. In Part B, the mean (SE) total Myasthenia Gravis Activities of Daily Living (MG‐ADL) score change between cycle baseline to Week 4 was −3.5 (0.94) and −4.0 (1.34) during Cycles 1 and 2, respectively. Quantitative Myasthenia Gravis (QMG) scores exhibited a similar trend. Minimal symptom expression (MSE; MG‐ADL, 0–1) was achieved by 72.7% (Cycle 1) and 80.0% (Cycle 2) of participants. Efgartigimod was well tolerated.

**Figure:**
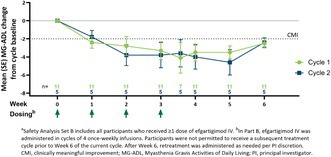
FIGURE 1

**Figure:**
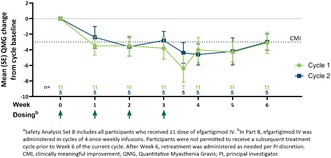
FIGURE 2

**Conclusion:** Efgartigimod treatment was well tolerated in adolescents and resulted in decreased levels of both total IgG and AChR‐Abs, similar to those observed in adult studies. Clinically meaningful improvements in MG‐ADL and QMG occurred, with many participants achieving MSE. Efgartigimod may address an unmet need in juvenile gMG therapy.

**Disclosure:** This study was sponsored by argenx; AK‐P, ANS, NLK, SR, and EHN reported financial/nonfinancial relationships with argenx at the time of submission. AB, JG, FM, and LL are employees of argenx, and JN and TvB are consultants for argenx.

## OPR‐085

### Patient acceptable symptom state in MG patients: A cross‐sectional study

#### P. Szczudlik; E. Sobieszczuk; K. Badowski; D. Orzechowski; B. Szyluk; A. Kostera‐Pruszczyk

##### Department of Neurology, Medical University of Warsaw, ERN EURO NMD, Poland

**Background and Aims:** To assess patient acceptable symptom state (PASS) in tertiary center cohort.

**Methods:** Cross‐sectional study of 101 MG patients, age 60.7 + 18.5 years, 67.3% F, 42.6% with EOMG, conducted between Feb2024 to Jun2025.

**Results:** 91% of patients received AChEI, 72.3% glucocorticoids (CS), 20.8% AZA, 19.8% MFM, 2% MTX, 44.6% were treated with IVIG (23.8% previous year), 12.9% with PLEX (5.9% previous year). 13.9% had history of myasthenic crisis, 15% had ocular symptoms only. 40.6% of patients were PASS‐positive, with MG‐ADL 4.4 + 3.8 vs. 8.3 + 4.1 in PASS‐negative, MGQoL15 was 16.3 + 10.5 vs. 32.2 + 12.3, respectively (*p* < 0.05). Patients treated with CS had lower MGQoL15 (27.22 + 13.7 vs. 16.9 + 11.6, *p* < 0.05) as compared with untreated, with no significant differences in MG‐ADL, age or sex between groups. Patients treated with IVIG or PLEX within last 12 months had worse MGQoL15 (29.9 + 15.2 vs. 23.0 + 13.7, *p* < 0.05) and MG‐ADL (8.1 + 5.1 vs. 5.5 + 4.0, *p* < 0.05). Females, as compared with males were more often PASS‐negative; (51.5% vs. 27.3%), had higher MG‐ADL (6.8 + 4.5 vs. 4.4 + 3.6) and MGQoL (26.8 + 14.0 vs. 19.7 + 14.2) *p* < 0.05. There were no differences between PASS‐positive and negative in age, history of myasthenic crisis. More patients with only ocular or no symptoms were PASS‐positive (67%) compared with gMG (35%, *p* < 0.01), with better MGQoL15 (9.7 + 10.3 vs. 28.2 + 12.8, *p* < 0.001). Threshold for PASS‐positive answer was ≤4.0 points in MG‐ADL (sensitivity 58%, specificity 75%).

**Conclusion:** Nearly 60% of patients do not accept their MG state. Treatment with CS had negative impact on PASS‐status despite no differences in MG‐ADL, sex or age, compared with those who not CS‐treated.

**Disclosure:** Nothing to disclose.

## OPR‐086

### From evidence to practice: Clinical audit of uptake of heart failure therapy in adult Duchenne muscular dystrophy at a tertiary centre

#### L. Sese ^1^; M. Guillermo^2^

##### ^*1*^ *Neuromuscular Complex Care Centre, National Hospital for Neurology and Neurosurgery, London, UK;* ^*2*^ *Department of Paediatric Cardiology, Great Ormond Street Hospital for Children, London, UK*

**Background and Aims:** Duchenne muscular dystrophy (DMD) is a progressive X‐linked neuromuscular disorder that leads to dilated cardiomyopathy and heart failure with reduced ejection fraction (HFrEF) in most patients by adulthood. Despite consensus recommendations, the extent of guideline‐directed medical therapy (GDMT) utilization remains varied across institutions. The objective of the study is to evaluate the proportion of patients who are receiving GDMT under specialist care at an accredited Duchenne centre.

**Methods:** A retrospective audit was conducted of patients aged ≥18 years with confirmed DMD and HFrEF under the care of an accredited Duchenne centre in January 2025. Prescription rates for key GDMT classes which include renin‐angiotension‐aldosterone‐system inhibitors (RAASi), beta‐blockers, mineralocorticoid receptor antagonists (MRA), and sodium‐glucose‐co‐transporter‐2 inhibitors were analysed.

**Results:** A total of 49 patients met inclusion criteria (mean age 23.5 ± 3.9 years; mean BMI 25.8 ± 8.4 kg/m^2^; mean LVEF 29.7 ± 9.1%). Genetic variants in the dystrophin gene included deletions (*n* = 32), duplications (*n* = 11), nonsense (*n* = 5), and point mutations (*n* = 1). 29 (59%) were on long‐term corticosteroids, and 28 (57%) used chronic non‐invasive ventilation. Medication utilisation was high: RAASi 96%, beta‐blocker 100%, MRA 88%, and SGLT2 inhibitor 82% patients. Others included ivabradine in 24% and loop diuretics in 10%.

**Figure:**
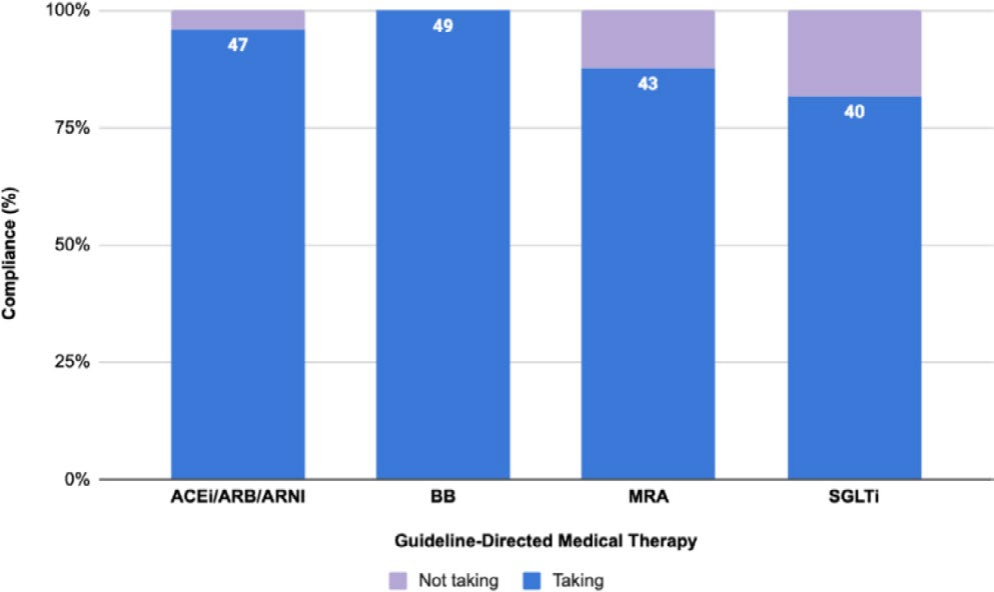
**FIGURE 1** GDMT compliance.

**Conclusion:** This audit demonstrated excellent adherence to GDMT among adults with DMD‐related HFrEF under an accredited Duchenne centre, with near‐universal prescription of beta‐blockers and renin–angiotensin system inhibitors. SGLT2i and MRA use were also notably high which shows proactive adoption of evidence‐based HF management.

**Disclosure:** Nothing to disclose.

## OPR‐087

### Rituximab in acetylcholine receptor antibody‐positive generalised myasthenia gravis: A single‐centre experience

#### M. Noushad; C. Allen; A. Pinto; F. Fatehi; G. Burke

##### Wessex Neuroscience Centre, Plymouth, UK

**Background and Aims:** This study evaluated the effectiveness of rituximab in acetylcholine receptor antibody (AChR‐Ab)‐positive generalised myasthenia gravis (MG) patients with explosive onset, steroid intolerance, or treatment resistance at Wessex Neurological Centre (2018–2025).

**Methods:** Retrospective analysis of 40 AChR‐Ab‐positive generalised MG patients (mean age 60 ± 17 years; 58% male; mean baseline Myasthenia Gravis Activities of Daily Living [MG‐ADL] 10 ± 6, Myasthenia Gravis Composite [MGC] 15 ± 9) receiving rituximab (median initial dose 2000 mg). Outcomes included MG‐ADL/MGC at baseline and 3–24 months, CD19 B‐cell counts, IgG, prednisolone dose. Repeated‐measures ANOVA assessed changes; McNemar tests compared pre/post RTX hospitalisations/plasmapheresis; *p* < 0.05 significant (JASP software).

**Results:** MG‐ADL (*F*(6,54) = 4.67, *p* = 0.001) and MGC (*F*(6,42) = 3.68, *p* = 0.005) improved significantly over time, though post‐hoc pairwise differences were non‐significant after Bonferroni correction. CD19 counts depleted markedly (*F*(6,12) = 10.71, *p* = 0.001); Rituximab led to clinically meaningful improvement, with 71% of patients achieving MCID (≥2‐point MG‐ADL and ≥3‐point MGC reduction) at one or more visits, alongside sustained B‐cell depletion and reduced rescue therapies. IgG remained stable (*p* = 0.93). Prednisolone dose reduced (*F*(6,42) = 5.49, *p* = 0.001). Hospital admissions dropped from 47.5% to 20% pre/post RTX (*p* = 0.013); plasmapheresis from 50% to 25% (*p* = 0.021). No serious adverse events with RTX.

**Figure:**
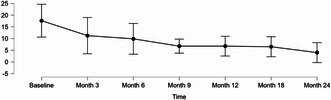
**FIGURE 1** MG ADL reduction.

**Figure:**
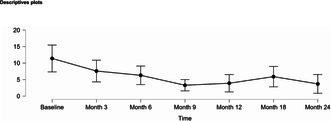
**FIGURE 2** MGC reduction.

**Figure:**
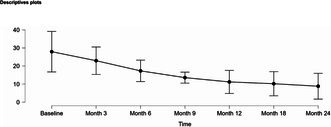
**FIGURE 3** Prednisolone dose reduction.

**Conclusion:** Rituximab achieved sustained B‐cell depletion, clinical improvement, and reduced rescue therapies/steroids in refractory AChR‐MG without impacting IgG, supporting its role in difficult‐to‐treat cases.

**Disclosure:** Nothing to disclose.

## OPR‐088

### Natural progression of oxidative phosphorylation pathology in mitochondrial myopathy

#### T. Bernardino Gomes ^1^; V. Di Leo^1^; C. Warren^1^; A. Khan^2^; I. Barrow^3^; G. Hudson^4^; D. M. Turnbull^1^; C. Lawless^1^; A. E. Vincent^1^

##### ^*1*^ *Mitochondrial Research Group, Translational and Clinical Research Institute, Newcastle University, UK;* ^*2*^ *Centre for Doctoral Training in Cloud Computing and Big Data, Newcastle University, UK;* ^*3*^ *NIHR Biomedical Research Centre, Translational and Clinical Research Institute, Newcastle University, UK;* ^*4*^ *Mitochondrial Research Group, Biosciences Institute, Newcastle University, UK*

**Background and Aims:** Mitochondrial myopathies are progressive and disabling disorders. Although their hallmark muscle oxidative phosphorylation (OXPHOS) pathology is widely measured in diagnosis and research, its longitudinal behaviour remains poorly characterised, limiting mechanistic insight and the interpretability of OXPHOS markers. Building on our previous characterisation of cross‐sectional intra‐individual variability in muscle OXPHOS pathology (PMID:41419999), we now aim to assess its natural history using comparable methods.

**Methods:** Imaging mass cytometry was used to quantify myofibre deficiency across OXPHOS complex (C) I, III, IV and V. Repeat muscle biopsies from a genetically confirmed mitochondrial myopathy cohort were analysed using our previous methods (PMID:41419999), with rates of change estimated from per‐patient bootstrapped differences.

**Results:** Myofibre deficiency in CI or CIV, and a combined CI‐IV‐V‐deficient profile, were the most prevalent abnormalities. Longitudinal differences were heterogeneous, with CI, CIV and CV often changing in tandem and preserving baseline relationships, while CIII deficiency was rare and changed inconsistently. Some patients exhibited stable or declining pathology over time. Both the magnitude and direction of change were patient‐specific and showed no association with major genotype‐phenotype groups, demographics or biopsy‐related factors.

**Conclusion:** These data show a slow, heterogeneous progression of muscle OXPHOS pathology in mitochondrial myopathy and indicate that extended follow‐up or large effect sizes are likely to be required for its use as a reliable indicator of disease modification. Apparent declines in some patients further indicate that endogenous or exogenous biological factors and/or sampling effects must be considered in future longitudinal research.

**Disclosure:** Nothing to disclose. This work was funded by the Wellcome Trust, UK.

## OPR‐089

### Incidence of lipid storage myopathy in Southwestern Sweden

#### U. Lindgren ^1^; A. Oldfors^2^; C. Hedberg‐Oldfors^3^

##### ^*1*^ *Department of Neurology and Department of Clinical Pathology, both Sahlgrenska University Hospital, and Department of Laboratory Medicine, Institute of Biomedicine, University of Gothenburg, Gothenburg, Region Västra Götaland, Sweden;* ^*2*^ *Department of Laboratory Medicine, Institute of Biomedicine, University of Gothenburg, and Department of Clinical Pathology, Sahlgrenska University Hospital, Gothenburg, Region Västra Götaland, Sweden;* ^*3*^ *Department of Clinical Genetics and Genomics and Department of Clinical Pathology, both Sahlgrenska University Hospital, and Department of Laboratory Medicine, Institute of Biomedicine, University of Gothenburg, Gothenburg, Region Västra Götaland, Sweden*

**Background and Aims:** Lipid storage myopathies (LSM) are a group of genetic and acquired rare disorders affecting the lipid metabolism. Symptoms typically derive from organs with a high energy demand and onset can occur from birth to late adulthood. This study aimed to describe the incidence and primary causes of adult‐onset LSM in a tertiary muscle biopsy centre serving >1.4 million adult inhabitants during 12 years.

**Methods:** A registry search identified patients with a histopathological diagnosis of adult‐onset LSM (Figure 1) 2014–2025 at Sahlgrenska University Hospital, Gothenburg, Sweden. We retrospectively reviewed histopathology, genetic data and medical records.

**Figure:**
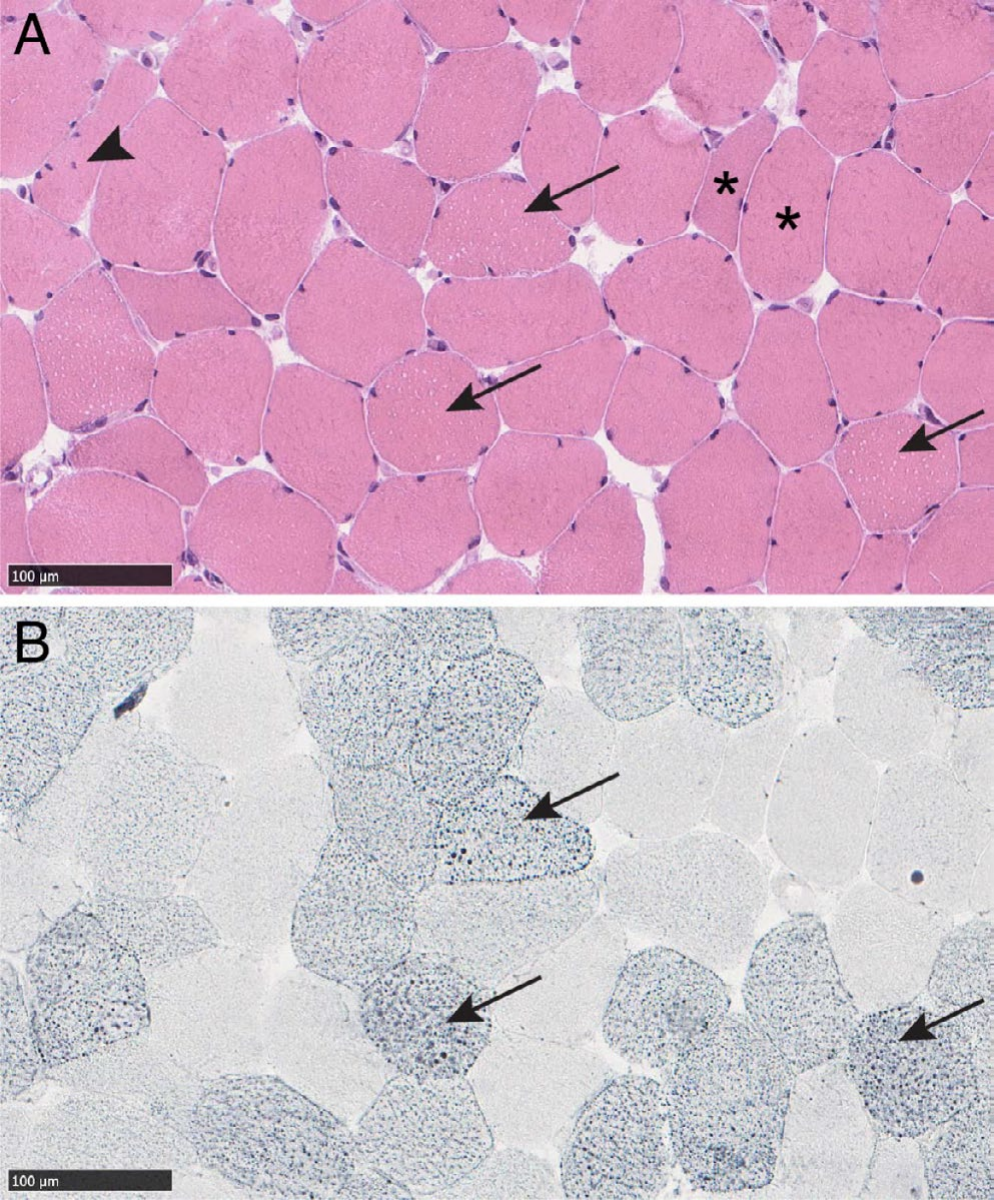
**FIGURE 1** Muscle biopsy showing lipid storage myopathy. (A) Vacuoles (arrows), fibre size variation (asterix) and internal nuclei (arrowheads) are seen (Haematoxylin/eosin). (B) Multiple lipid droplets are stained with Sudan black (arrows).

**Results:** A total of 31 patients were included (17 women, 14 men). Median age at biopsy was 55 years (range 23–79 years). The primary cause of LSM was identified in 24 patients. Sixteen patients had sertraline‐associated MADD (Figure 2A). Three patients had a genetically confirmed disease with variants in ETFDH, PNPLA2 and CPTII respectively. One patient was a symptomatic carrier of an ETFDH variant. Other causes included one patient with a pituitary tumour, and three patients with inflammatory myopathy. Seven patients lacked sufficient data to establish the cause of LSM. The mean incidence of LSM in adults in Region Västra Götaland, Sweden, was 1.45 patients per million inhabitants per year (Figure 2B). The mean incidence of sertraline‐associated MADD was 1.76 patients per 100 000 inhabitants treated with sertraline per year.

**Figure:**
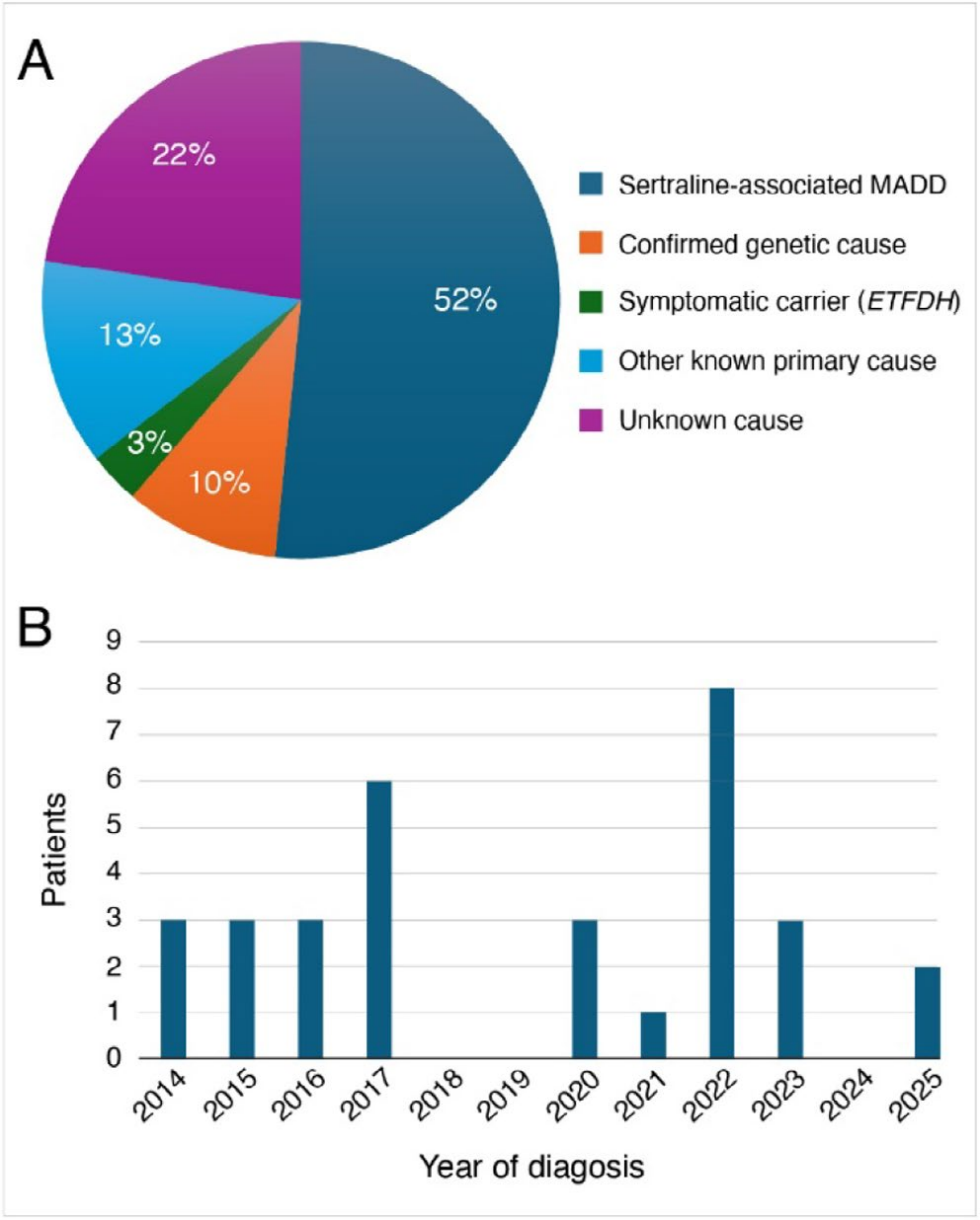
**FIGURE 2** (A) Primary causes and (B) Incidence of adult‐onset lipid storage myopathy in 31 adult patients diagnosed at Sahlgrenska University Hospital, Gothenburg, Sweden 2014–2025.

**Conclusion:** Sertraline‐associated MADD was the most common primary cause of LSM in this study and is important to consider in patients diagnosed with lipid storage myopathy without genetic cause.

**Disclosure:** Nothing to disclose.

## Monday, June 29 2026

## Neuroinformatics

## OPR‐090

### Brain and CSF volume changes in AI‐assisted MRI volumetry in spontaneous intracranial hypotension

#### A. El Rahal ^1^; P. Scheffler^1^; K. Wolf^1^; F. Volz^1^; N. Lützen^2^; C. Zander^3^; H. Urbach^2^; J. Beck^1^

##### ^*1*^ *Department of Neurosurgery, Medical Center University of Freiburg, Germany;* ^*2*^ *Department of Diagnostic and Interventional Neuroradiology, Medical Center University of Freiburg, Freiburg, Germany;* ^*3*^ *Department of Diagnostic and Interventional Radiology, Medical Center University of Freiburg, Freiburg, Germany*

**Background and Aims:** Surgical closure of spinal CSF leaks in SIH patients is associated with quantifiable MRI changes, such as increased intraventricular CSF volume and region‐specific expansion and contraction of cerebral lobes, which could help monitoring these patients on MRI follow‐up. The pathophysiological significance and prognostic value of brain and CSF volume changes remain to be elucidated. AI‐assisted image quantification could be a useful, time‐efficient tool for analyzing these changes on a larger scale.

**Methods:** We analyzed 107 patients with SIH who underwent surgical closure of a spinal CSF leak. We used AI‐assisted volumetry of pre‐ and postoperative MRIs using the AssemblyNet segmentation model to quantify CSF and brain region volumes, which were correlated with patient‐reported clinical outcome measures.

**Results:** Patients demonstrated a significant increase in lateral ventricle volume (+2.83 cm^3^, +17.8%) as well as third (+0.22 cm^3^, +30.1%) and fourth ventricle volume (+0.20 cm^3^, +14.1%), after SIH surgery (*p* < 0.001 for all). There was an increased frontal lobe volume (+6.23 cm^3^, +3.1%, *p* < 0.050), and there were small but significant relative volume decreases in temporal horn volume, brainstem, and occipital lobe volume. Clinical scores improved significantly postoperatively but showed low correlation with volumetric changes.

**Figure:**
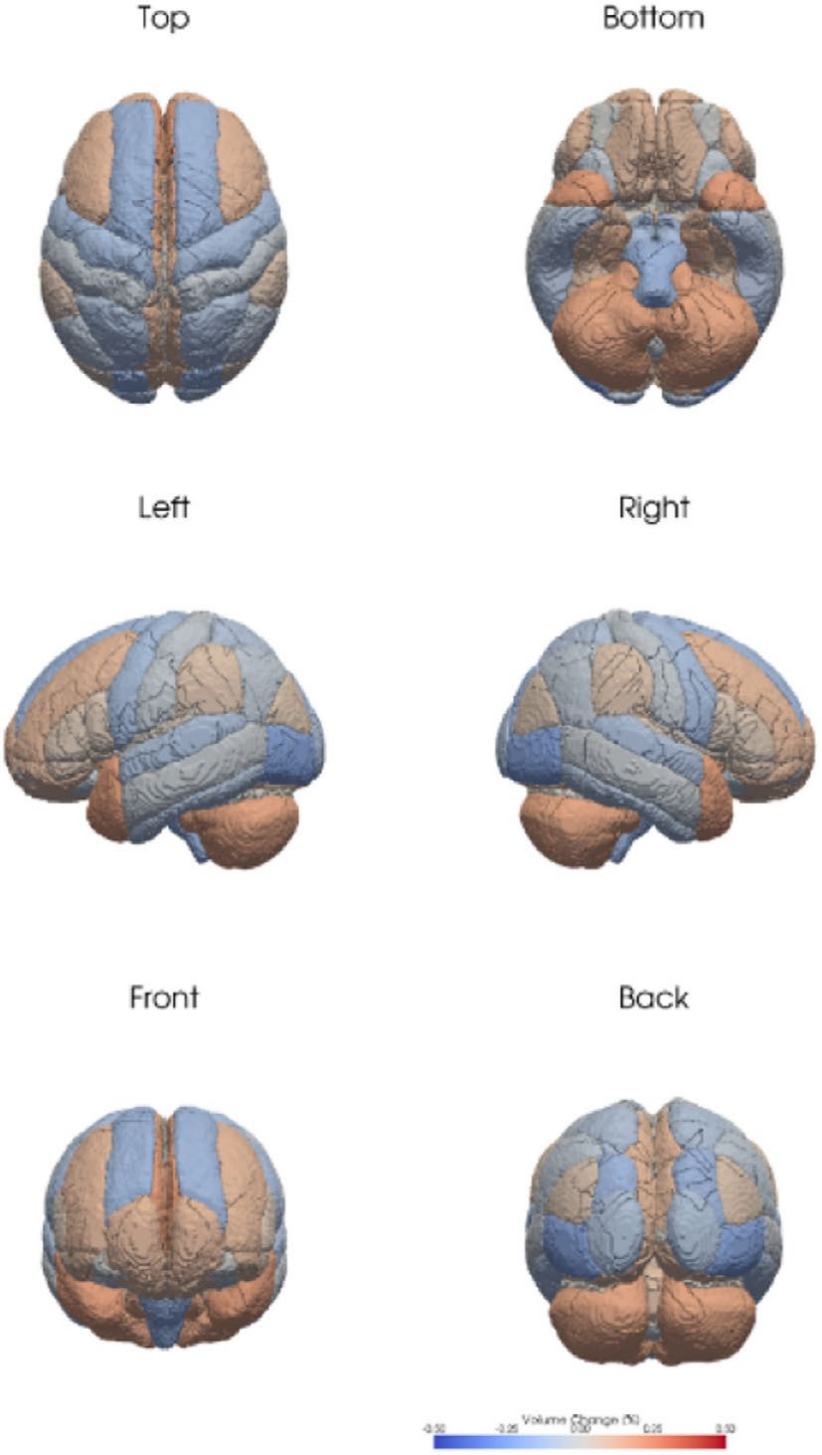
**FIGURE 1** Illustration of volume changes by area. Areas in orange are expanding, areas in blue are contracting.

**Figure:**
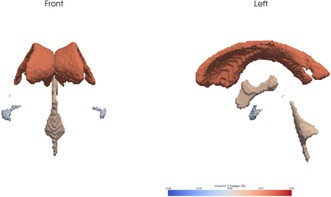
**FIGURE 2** Illustration of volume changes within the ventricular system. Areas in red are expanding, areas in blue are contracting.

**Figure:**
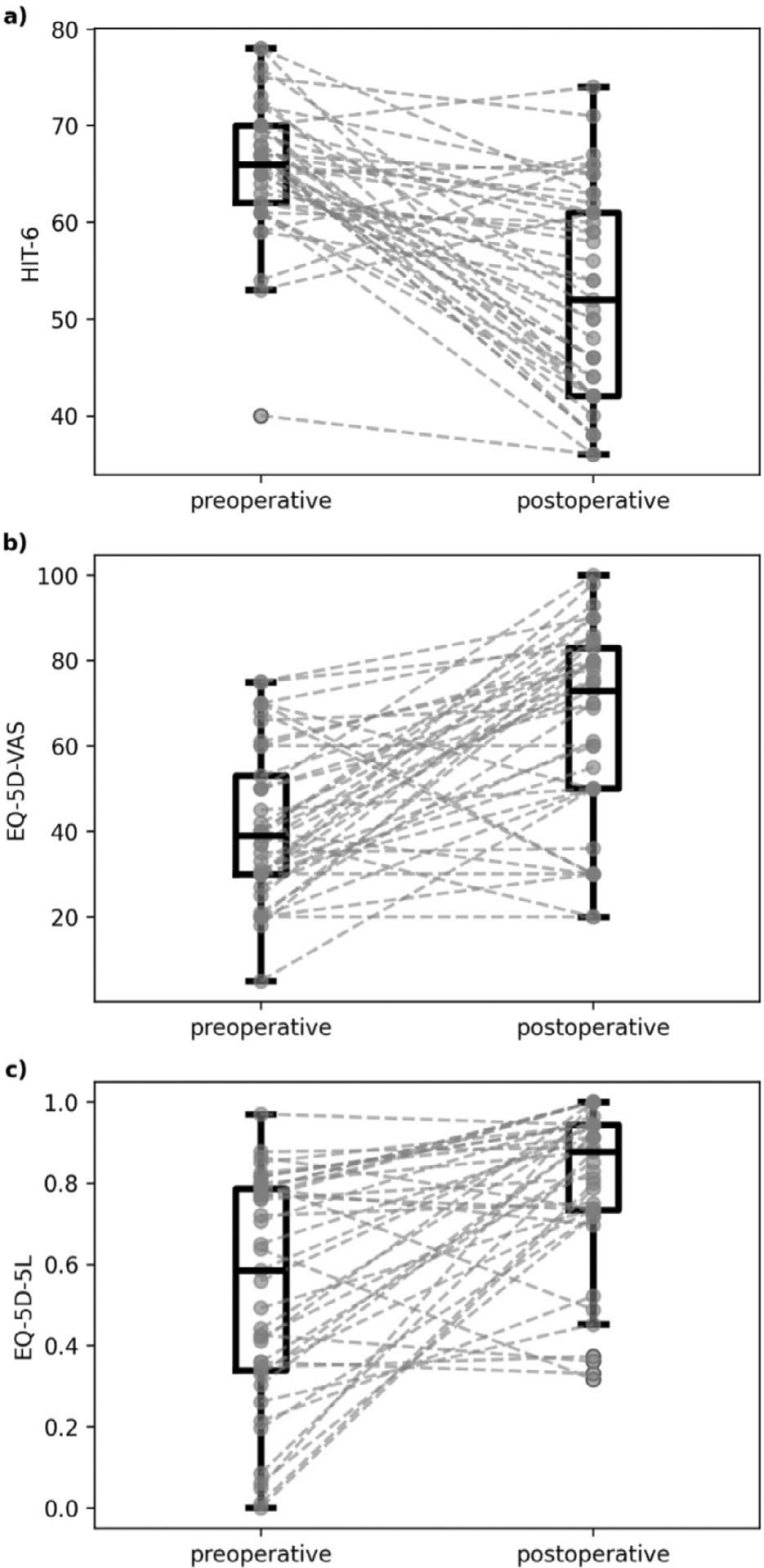
**FIGURE 3** Boxplots compare preoperative and postoperative HIT‐6 scores at (a), EQ‐VAS scores at (b), and EQ‐5D‐5L scores at (c). The dotted lines represent the evolution of each patient's scores.

**Conclusion:** Surgical closure of spinal CSF leaks in SIH patients is associated with quantifiable MRI changes: increased intraventricular CSF volume and region‐specific expansion and contraction of cerebral lobes, which could help monitor these patients on MRI follow‐up. AI‐assisted image quantification could be a useful, time‐efficient tool for analyzing these changes at scale.

**Disclosure:** Nothing to disclose.

## OPR‐091

### Artificial intelligence analysis of patient videos for neurological diagnosis: A systematic review

#### C. Tatit^1^; S. Vallamchetla^1^; A. Patel^1^; A. Safa^1^; F. Colella ^2^; A. Mahadeen^1^; A. Kissinger‐Knox^1^; Y. Khand^1^; C. Tao^3^; A. Abdelhameed^3^; M. Roberts^3^; W. Tatum^1^; B. Freund^1^

##### ^*1*^ *Department of Neurology, Mayo Clinic, Jacksonville, USA;* ^*2*^ *Department of Biomedical Sciences, Humanitas University, Milan, Italy;* ^*3*^ *Department of AI and Informatics, Mayo Clinic, Jacksonville, USA*

**Background and Aims:** Neurological diagnosis frequently depends on visual assessment during examination and video review for paroxysmal and movement disorders. However, clinician interpretation remains subjective, variable, and unsuited to growing tele‐neurology data volumes. Computer vision and deep learning advances enable quantitative analysis, potentially converting qualitative neurological signs into reproducible digital biomarkers.

**Methods:** Following a registered PROSPERO protocol (CRD420251070499), we searched databases from inception to June 2025 for studies using AI on video to detect, classify, or quantify neurological disorders. From included papers, we extracted clinical aim, video modality, model type, validation approach, diagnostic metrics. Risk of Bias assessment was conducted following the Newcastle–Ottawa Scale tool. Heterogeneity precluded meta‐analytic synthesis.

**Results:** Thirty studies including 22,532 participants met inclusion criteria. Performance varied by condition: acute stroke/TIA identification reached 85.8% accuracy; post‐stroke motor deficit quantification reported 78%–83% accuracy, while facial weakness detection reached 94.3% accuracy. Neonatal seizure detection achieved >90% specificity. Movement‐disorder applications (bradykinesia, tremor, gait, tics) reported 73%–97% accuracies. Pediatric and fetal tasks reported 0.80–0.90 AUCs for early cerebral palsy and congenital malformations. Most studies were moderate quality with limited external validation.

**Figure:**
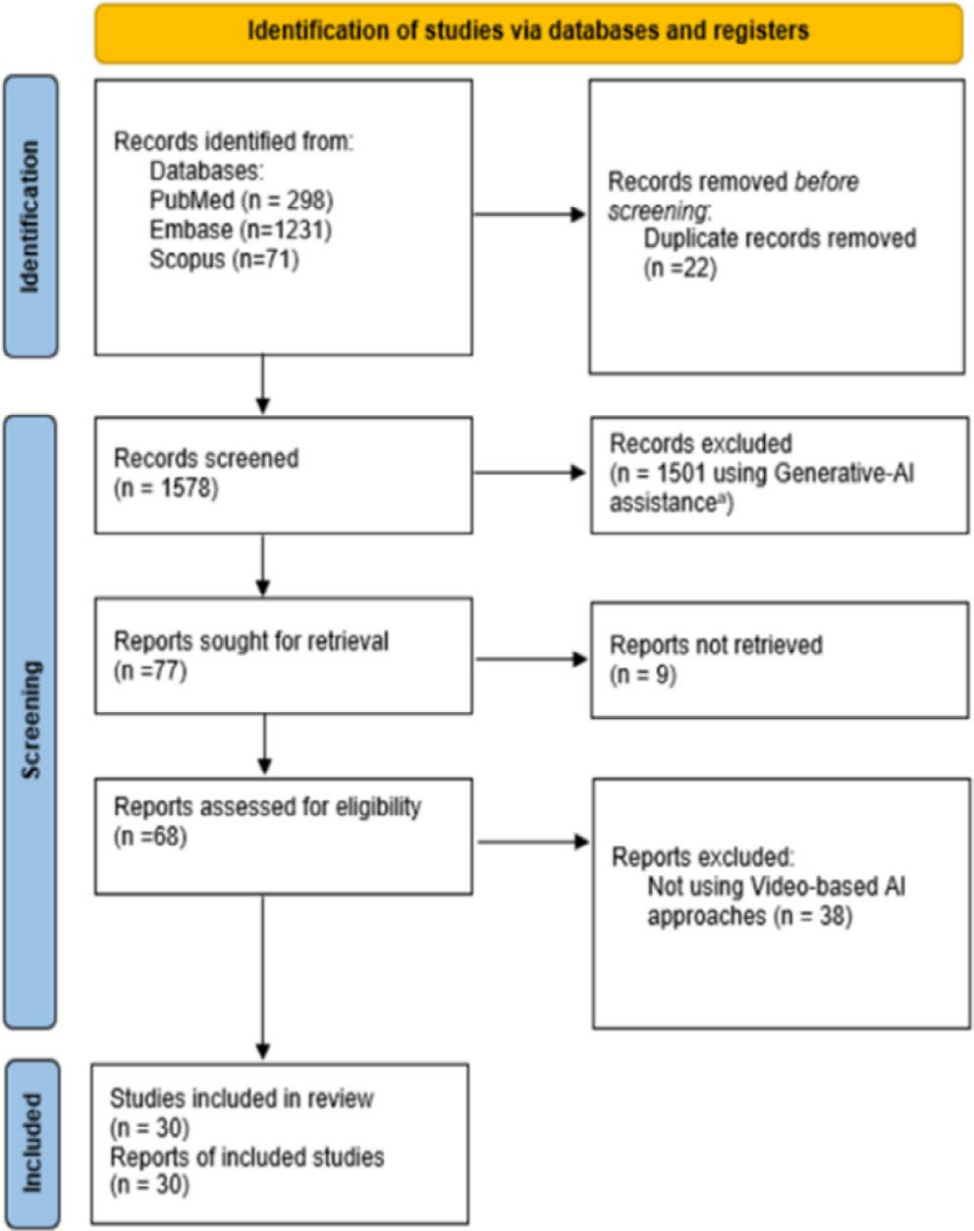
**FIGURE 1** PRISMA flow diagram illustrating the screening process.

**Figure:**
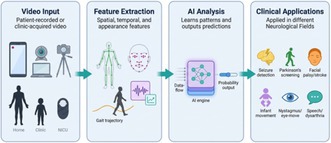
**FIGURE 2** Illustration of the typical video‐based AI workflow.

**Figure:**
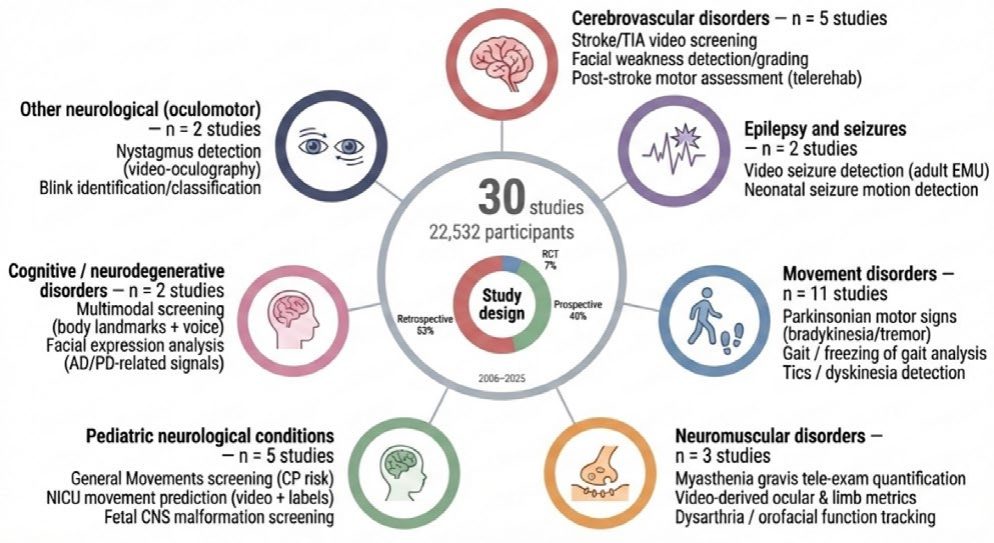
**FIGURE 3** Graphical synthesis of included studies.

**Conclusion:** AI based video analysis shows potential to support neurological evaluation across acute, chronic, and developmental disorders, but translation to clinical practice is limited by small datasets, heterogeneous protocols, and scarce real‐world evaluation. Prospective multicenter evidence with standardized methodology and robust external validation are required to support the integration of AI‐video analysis in neurological care.

**Disclosure:** Nothing to disclose.

## OPR‐092

### Speech‐based biomarkers for scalable detection of Alzheimer's disease, mild cognitive impairment, and Parkinson's disease

#### L. Gervaise ^1^; D. Avalos^2^; O. Kauffman^2^; R. Etemad‐Sajadi^3^; S. Ghosh^4^

##### ^*1*^ *Virtuosis Artificial Intelligence SA, Lausanne, Switzerland;* ^*2*^ *Swiss Data Science Center (SDSC), EPFL, Lausanne, Switzerland;* ^*3*^ *Hospitality Innovation and Data Analytics, EHL Hospitality Business School, Lausanne, Switzerland;* ^*4*^ *Department of Brain and Cognitive Sciences, McGovern Institute for Brain Research at MIT, Cambridge, USA*

**Background and Aims:** Early detection of neurodegenerative disorders is limited by the cost, accessibility, and burden of standard clinical assessments. Speech analysis offers a low‐burden, non‐invasive, and scalable alternative. This study evaluates a unified speech‐based framework for detecting Alzheimer's disease (AD), Mild Cognitive Impairment (MCI), and Parkinson's disease (PD) from spontaneous speech, with an emphasis on robustness across clinical sites, recording conditions, and speech varieties.

**Figure:**
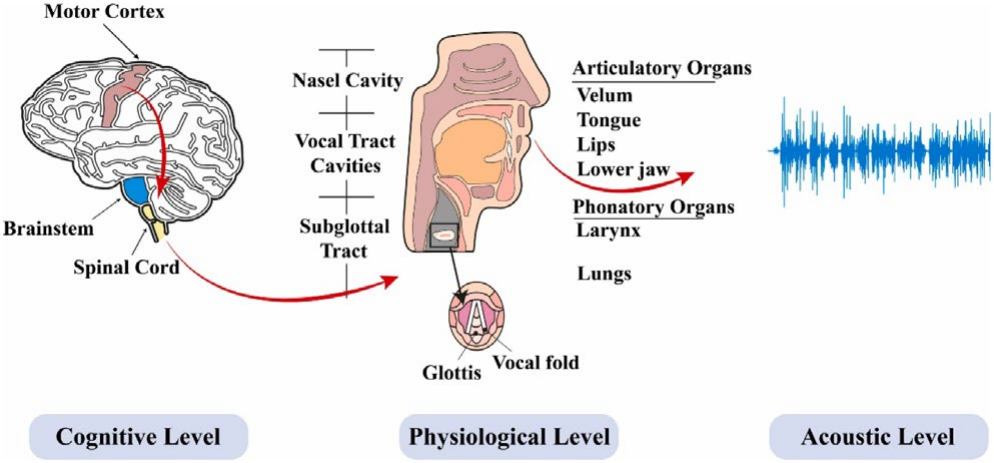
**FIGURE 1** Schematic representation of the neural, physiological, and acoustic levels involved in speech production, from cortical and brainstem control to vocal tract articulation and the resulting acoustic signal.

**Methods:** Spontaneous speech recordings were analyzed from multiple clinical and research cohorts, including six datasets for cognitive impairment and five for PD, each treated as an independent domain. Audio was standardized and processed using a shared self‐supervised speech encoder. To reduce site‐, language‐, and recording‐related bias, domain‐adversarial learning was applied. For cognitive impairment, binary (AD vs. healthy control), three‐class (healthy/MCI/AD), and ordinal (≥MCI, ≥AD) classifiers were trained using a two‐phase strategy combining pretraining and multitask learning. PD detection used binary classification, including a model adapted to Swiss‐dialect speech. Performance was evaluated using five‐fold cross‐validation.

**Figure:**
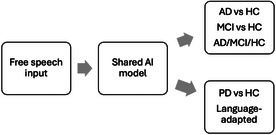
**FIGURE 2** Overview of the unified speech analysis framework, in which a shared model processes spontaneous speech and supports multiple classification tasks for cognitive impairment and Parkinson's disease.

**Results:** For cognitive impairment, AD versus healthy classification achieved a sensitivity of 0.794 and specificity of 0.911, while MCI versus healthy reached a sensitivity of 0.851 and specificity of 0.782; three‐class AD/MCI/healthy classification showed a macro‐sensitivity of 0.723 and macro‐specificity of 0.860. For Parkinson's disease, baseline detection achieved a sensitivity of 0.659 and specificity of 0.937, while dialect‐adapted models improved sensitivity to 0.845 and specificity to 0.831.

**TABLE 1** Sensitivity and specificity of speech‐based models for Alzheimer's disease, mild cognitive impairment, and Parkinson's disease.

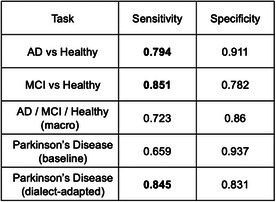

**Conclusion:** Speech‐based biomarkers enable scalable, low‐burden screening and monitoring of neurodegenerative diseases and show robust performance across disorders, centers, and speech varieties, supporting their potential integration into routine neurological practice.

**Disclosure:** Lara Gervaise holds equity in Virtuosis.

## OPR‐093

### Machine learning for multiple sclerosis: Classification, phenotype differentiation and disability prediction using demographic, clinical and MRI data

#### M. Rocca ^1^; P. Valsasina^2^; L. Storelli^2^; N. Tedone^3^; P. Preziosa^1^; P. Pantano^4^; S. Tommasin^5^; A. Gallo^6^; A. Bisecco^6^; A. Bianchi^7^; N. De Stefano^7^; M. Filippi^8^

##### ^*1*^ *Neuroimaging Research Unit, Division of Neuroscience, and Neurology Unit, IRCCS San Raffaele Scientific Institute, Milan, Italy; Vita‐Salute San Raffaele University, Milan, Italy;* ^*2*^ *Neuroimaging Research Unit, Division of Neuroscience, IRCCS San Raffaele Scientific Institute, Milan, Italy;* ^*3*^ *Neuroimaging Research Unit, Division of Neuroscience, IRCCS San Raffaele Scientific Institute, Milan, Italy; Vita‐Salute San Raffaele University, Milan, Italy;* ^*4*^ *Sapienza University of Rome, Department of Human Neuroscience, Rome, Italy; IRCCS Neuromed, Pozzilli (IS) , Italy;* ^*5*^ *Department of Human Neuroscience, Sapienza University of Rome, Rome, Italy;* ^*6*^ *Department of Advanced Medical and Surgical Sciences, University of Campania Luigi Vanvitelli, Naples, Italy; AOU Luigi Vanvitelli, First Division of Neurology and Neurophysiopathology, Naples, Italy;* ^*7*^ *Department of Medicine, Surgery and Neuroscience, University of Siena, Siena, Italy;* ^*8*^ *Neuroimaging Research Unit, Division of Neuroscience, Neurology Unit, Neurorehabilitation Unit, and Neurophysiology Service, IRCCS San Raffaele Scientific Institute, Milan, Italy; Vita‐Salute San Raffaele University, Milan, Italy*

**Background and Aims:** Multiple sclerosis (MS) shows marked heterogeneity in clinical presentation and progression patterns. Here, we used machine learning (ML) to investigate the combined ability of demographic, clinical and MRI variables in: (i) identifying MS patients from healthy controls (HC); (ii) classify MS phenotypes; and (iii) predict disability, assessed using the Expanded Disability Status Scale (EDSS) score.

**Methods:** From the Italian Neuroimaging Network Initiative, we selected 1554 MS patients (1143 relapsing/411 progressive MS) and 520 age‐matched HC. Participants underwent neurological evaluation and T2‐/3D T1‐weighted MRI, from which lesion volume (LV), normalized whole‐brain and tissue compartment volumes were calculated. Different ML models (including support vector machines and multi‐layer perceptron) were implemented for the above‐mentioned classification tasks. SHAP analysis ranked the most important variables.

**Results:** The described ML tools were able to classify MS patients vs. HC with an accuracy ranging from 94% to 96%. Measures consistently showing top contributions included T2 LV and normalized cerebellar/brainstem volumes. ML models were able to classify relapsing from progressive MS patients with 92% accuracy. Top discriminating variables were EDSS score, age, normalized cortical grey matter volume, and normalized hippocampal/thalamic volumes. Finally, ML models were able to predict EDSS score with 0.74–0.76 intra‐class correlation. The top variables contributing to EDSS prediction were T2 LV, sex, normalized cortical grey matter and thalamic volumes.

**Conclusion:** ML models were able to differentiate MS patients from HC, classify MS subtypes and predict disability with high accuracy, supporting the potential of ML in advancing personalized MS care.

**Disclosure:** Funded by the European Union, Next Generation EU, NRRP M6C2, Investment 2.1 Enhancement and strengthening of biomedical research in the NHS, PNRR‐MAD‐2022‐12376530, CUP master C43C22001290007 MA Rocca received consulting fees from Biogen, Bristol Myers Squibb, Roche; and speaker honoraria from Alexion, Biogen, Bristol Myers Squibb, Celgene, Horizon Therapeutics Italy, Merck Serono SpA, Mitsubishi‐Tanabe Pharma, Neuraxpharm, Novartis, Roche, Sandoz, and Sanofi. She receives research support from the MS Society of Canada, the Italian Ministry of Health, the Italian Ministry of University and Research, and FISM. P. Valsasina, L. Storelli, N. Tedone, S. Tommasin, A. Bisecco have nothing to disclose. P Preziosa received speaker honoraria from Roche, Biogen, Novartis, Merck, Bristol Myers Squibb, Genzyme, Horizon and Sanofi. P. Pantano has received funding for travel from Novartis, Genzyme, and Bracco, and a speaking honorarium from Biogen. She receives research support from the Italian Ministry of Health, the Italian Ministry of University and Research, and FISM. A. Gallo received speaker and consulting fees from Biogen, Bristol Myers Squibb, Genzyme, Janssen, Juivsè, Merck Serono, Mylan, Novartis, Roche, Sandoz, and Teva, and receives research support from the Italian Ministry of Health, the Italian Ministry of University and Research, and FISM. A. Bianchi received a research grant from the Italian Society of Neurology, a MAGNIMS‐ECTRIMS fellowship in 2023; she received speaking honoraria from Biogen. N. De Stefano is a consultant for Biogen, Merck KGaA, Novartis, Sanofi‐Genzyme, Roche and Teva, has grants from FISM and Novartis, is on the speakers' bureau of Biogen, Merck KGaA, Novartis, Roche, Sanofi‐Genzyme, and Teva; and received travel funds from Merck KGaA, Novartis, Roche, Sanofi‐Genzyme and Teva. He is co‐founder of Siena Imaging s.r.l. M Filippi received compensation for consulting services from Almirall, Biogen, Bristol‐Myers Squibb, Eli Lilly, Merck, Novartis, Roche, Sanofi; speaking activities from Amgen, Bayer, Biogen, Bristol‐Myers Squibb, Celgene, Chiesi Italia SpA, Eisai, Eli Lilly, Fujirebio, Genzyme, Janssen, Merck, Neopharmed Gentili, Neuraxpharm, Novartis, Novo Nordisk, Roche, Sanofi, Takeda; participation in Advisory Boards for Alexion, Biogen, Bristol‐Myers Squibb, Eli Lilly, GE Healthcare Ltd, Merck, Neuraxpharm, Novartis, Roche, Sandoz, Sanofi, Takeda; scientific direction of educational events for Biogen, Merck, Roche, Celgene, Bristol‐Myers Squibb, Lilly, Novartis, Sanofi‐Genzyme; he receives research support from Biogen Idec, Merck‐Serono, Novartis, Roche, the Italian Ministry of Health, the Italian Ministry of University and Research, and FISM.

## Movement Disorders 2

## OPR‐094

### Primary brain calcification: Survey on the efficacy of medical therapy on movement and psychiatric disorders in an Italian cohort

#### G. Bonato ^1^; F. Pistonesi^2^; A. Antonini^2^; M. Carecchio^2^

##### ^*1*^ *Movement Disorders Unit, Neuroscience Department, University of Padova, Padova, Italy; Foundation IRCCS Ca' Granda Ospedale Maggiore Policlinico, Neurology Unit, University of Milan, Milan, Italy;* ^*2*^ *Movement Disorders Unit, Neuroscience Department, University of Padova, Padova, Italy*

**Background and Aims:** Primary Brain Calcification (PBC) is a neurodegenerative disorder characterized by brain calcium deposition, causing movement disorders, psychiatric/cognitive disturbances. Evidence on symptomatic treatment efficacy is limited. We here assess response to medical therapy in a cohort of PBC patients.

**Methods:** Patients diagnosed with PBC through CT‐scan and blood tests at Padova University underwent clinical evaluation and pharmacological anamnesis; treatment response was rated by neurologists and subjective patients' perception as absent, partial, or satisfying.

**Results:** 35 PBC subjects with movement disorders underwent pharmacological (32) and/or rehabilitation treatment (24), with a multidrug approach in 65.5%. Parkinsonism was the most common phenotype (75%); all patients received Levodopa, dopamine agonists in 13 (54%), MAO/COMT‐inhibitors in 6 (25%). Dystonia was treated with anticholinergics or botulinum toxins in 7 cases (22%), benzodiazepine/antiepileptics were added in resistant cases without further benefit; anticholinergic and carbamazepine were effective in 2 PKD subjects. Essential/dystonic tremor was treated with beta‐blockers or antiepileptics (4 subjects, 12.5%). Levodopa trial was adopted in tremor or dystonia as second line treatment, and in 13 subjects with cerebellar symptoms. Overall, 28% of patients had a satisfying response and 37% a partial response. Cerebellar symptoms, dysarthria, and atypical parkinsonism responded significantly worse (*p* = 0.03) (Figure 1). Psychiatric symptoms required treatment in most patients (33), achieving satisfactory control in 80%; main drugs were SSRIs, atypical antipsychotics, anxiolytics and benzodiazepines.

**Figure:**
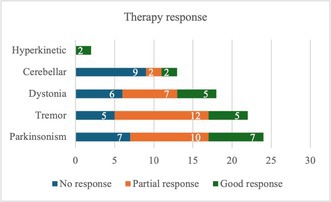
**FIGURE 1** Therapy response according to patients and clinician's survey in different movement disorders.

**Conclusion:** Symptomatic medical treatment provides significant benefit in over half of PBC patients, except for cerebellar features, supporting the need for systematic therapeutic evaluation. Systematic assessment will help in defining therapy guidelines in the future.

**Disclosure:** Data partially submitted for 2026 Italian movement disorders congress. Nothing to disclose.

## OPR‐095

### Continuous subcutaneous foslevodopa/foscarbidopa infusion in advanced Parkinson's disease: results from a prospective real‐world study

#### G. Pinola ^1^; V. Leta^2^; M. Tosi^2^; C. Leuzzi^2^; G. Gaudiano^2^; A. Braccia^2^; F. Pirone^2^; S. Rinaldo^2^; R. Cancilla^2^; N. Golfrè Andreasi^2^; L. Romito^2^; G. Devigili^2^; R. Cilia^2^; A. Elia^2^; R. Eleopra^2^

##### ^*1*^ *Neurology and Stroke Unit, Ospedale di Circolo e Fondazione Macchi, Varese, Italy;* ^*2*^ *Parkinson and Movement Disorders Unit, Department of Clinical Neurosciences, Fondazione IRCCS Istituto Neurologico Carlo Besta, Milan, Italy*

**Background and Aims:** In advanced Parkinson's disease (APD), motor fluctuations and dyskinesias often persist despite optimized oral therapy, supporting the transition toward continuous dopaminergic delivery. Recently, continuous subcutaneous foslevodopa/foscarbidopa (LDp/CDp) became available, but real‐world data remain limited. This study aimed to evaluate the effectiveness and tolerability of continuous subcutaneous LDp/CDp infusion in patients with APD in a real‐world setting.

**Methods:** This prospective observational study included APD patients referred to the outpatient clinics for device‐aided therapies at Fondazione IRCCS Istituto Neurologico “Carlo Besta”, consecutively screened from March 2025 over the following 18 months and followed‐up for 6 months after treatment initiation. Motor complications were assessed using the Movement Disorder Society–Unified Parkinson's Disease Rating Scalepart IV (MDS‐UPDRS IV), the MDS–Unified Dyskinesia Rating Scale (MDS‐UDysRS) and 3‐day Hauser diaries. Non‐motor symptomswere assessed using validated scales. Levodopa equivalent daily dose (LEDD), adverse events and treatment discontinuations were recorded.

**Results:** Out of ninety‐nine patients screened, 45 patients were initiated on LDp/CDp and followed up in outpatient clinics. Treatment discontinuation occurred in 9 (20.0%) patients, mainly due to local infusion‐site reactions. Among patients who continued therapy, MDS‐UPDRS IV, MDS‐UDysRS scores and daily on‐time with dyskinesias significantly decreased, while on‐time without dyskinesias increased. Oral LEDD was reduced, while total LEDD increased, reflecting continuous 24‐h dopaminergic delivery. Non‐motor symptoms showed a tendency toward improvement.

**Figure:**
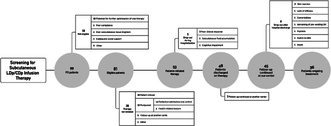
**FIGURE 1** Flowchart of patients screened and initiated on continuous subcutaneous Foslevodopa/Foscarbidopa infusion therapy.

**Conclusion:** Continuous subcutaneous LDp/CDp infusion reduced motor complications and improved on‐time quality with acceptable tolerability in a real‐world cohort of APD, supporting its use in clinical routine with appropriate patient selection and follow‐up.

**Figure:**
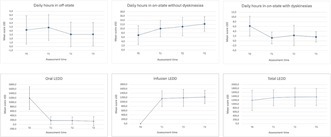
**FIGURE 2** Longitudinal changes in motor‐diary outcomes and LEDD.

**Disclosure:** Nothing to disclose.

## OPR‐096

### Unraveling multiple system atrophy through skin biopsy: Biomarkers of autonomic dysfunction and α‐synuclein aggregation

#### S. Mazzetti^1^; L. Maldera^2^; A. Luppino^1^; G. Gaudiano^1^; A. Elia^1^; G. Simmini^3^; R. Lombardi^4^; V. Leta^1^; A. Ciullini^5^; F. Colucci^1^; D. Cartelli^4^; E. Salvi^2^; R. Cilia^1^; F. Moda^5^; R. Eleopra^1^; G. Devigili ^1^

##### ^*1*^ *Parkinson's and Movement Disorders Unit, Department of Clinical Neurosciences, Fondazione IRCCS Istituto Neurologico Carlo Besta, Milan, Italy;* ^*2*^ *Data Science Center, Fondazione IRCCS Istituto Neurologico “Carlo Besta,” Milan, Italy;* ^*3*^ *Dementia and Degenerative Diseases of CNS Unit, Fondazione IRCCS Istituto Neurologico Carlo Besta, Milan, Italy;* ^*4*^ *Neuroalgology Unit, Fondazione IRCCS Istituto Neurologico “Carlo Besta,” Milan, Italy;* ^*5*^ *SSD Laboratory Medicine, Fondazione IRCSS Istituto Neurologico Carlo Besta, Milan, Italy*

**Background and Aims:** Multiple System Atrophy (MSA) is a progressive neurodegenerative disorder characterized by marked clinical heterogeneity. Identifying accessible biomarkers that mirror underlying neuropathology and differentiate MSA subtypes remains an unmet need. This study explores the skin as an accessible window, aiming to identify peripheral signatures that distinguish MSA phenotypes and capture clinically meaningful differences.

**Methods:** We conducted a cross‐sectional study in 49 patients with clinically established MSA (22 MSA‐P, 27 MSA‐C). Participants underwent comprehensive clinical and instrumental evaluations, including brain magnetic resonance imaging, DaTSCAN, cardiovascular autonomic testing, and sudomotor assessment. Peripheral biomarkers included α‐synuclein seed amplification assay (SAA) performed on olfactory mucosa, and skin biopsy analyses assessing phosphorylated α‐synuclein (p‐syn) deposits and synaptic terminal density. High‐resolution confocal images were analyzed using an automated image software to quantify synaptic markers.

**Results:** MSA‐C was associated with more severe orthostatic hypotension (OH) compared with MSA‐P. A postganglionic cholinergic sudomotor impairment was observed across nearly all patients. Synaptic terminals co‐expressing vasoactive intestinal peptide and tyrosine hydroxylase were significantly denser in MSA‐P than in MSA‐C, suggesting phenotype‐specific compensatory or regenerative mechanisms; p‐syn deposits exhibited a distal‐to‐proximal gradient in both somatic and autonomic fibers and correlated significantly with OH. Integrating p‐syn pathology with SAA results increased sensitivity to 90% in differentiating MSA subtypes, with synaptic loss accounting for most remaining negative cases.

**Conclusion:** By using a multimodal approach that integrates clinical, molecular, and morphological data, this study provides novel insights into MSA heterogeneity and support the skin as a meaningful source of disease‐relevant biomarkers to refine MSA characterization.

**Disclosure:** Nothing to disclose.

## OPR‐097

### Local field aperiodic spectral power modulated by deep brain stimulation in Parkinson's disease

#### M. Bočková

##### Brain and Mind Research Program, Central European Institute of Technology, Masaryk University, Brno, Czech Republic

**Background and Aims:** Aperiodic spectral broadband power has been described recently as reflecting Parkinson's disease (PD) severity. It has therefore become an increasing focus of research interest in the context of the new adaptive deep brain stimulation (aDBS) approach. We aimed to study the influence of DBS on the main parameters of the aperiodic spectral component of local field potentials (LFPs).

**Methods:** LFPs were recorded from the subthalamic nucleus (STN) in patients with PD (*n* = 22) during a simple experimental paradigm that included 5 min of resting state and a short gait task during DBS “off” and “on” conditions (“off” medication). Classical spectral analysis was performed using Fast Fourier Transform (FFT); the analysis of the aperiodic component was performed by fitting oscillations and a one‐over‐F (FOOOF) approach. In a subset of the patients (*n* = 14), it was possible to evaluate the symptom progression over time after 1 year by analyzing the control measurement.

**Results:** The severity of hypokinetic/rigid symptomatology, measured by MDS‐UPDRS, correlated significantly with both of the aperiodic components characteristics of the STN LFPs: the slope and the offset. These parameters were significantly modified by DBS, as evaluated with a Wilcoxon signed‐rank test during the resting state and during the gait task. Aperiodic power remained stable over time during the control measurements.

**Conclusion:** The aperiodic component in LFPs may reflect pathological activity of the neuronal network and might serve as a new potential clinical marker in PD. The variable relation to conventional beta oscillopathy indicates the involvement of multiple neural mechanisms.

**Disclosure:** Nothing to disclose.

## OPR‐098

### Clinical progression and genetic pathways in body‐first and brain‐first Parkinson's disease

#### M. Passaretti ^1^; D. Veréb^1^; M. Mijalkov^1^; Y. Chang^2^; H. Zhao^2^; B. Zufiria‐Gerbolés^1^; J. Sun^1^; G. Volpe^2^; N. Rivera^3^; M. Bologna^4^; J. Pereira^1^

##### ^*1*^ *Department of Clinical Neuroscience, Karolinska Institutet, K8 Klinisk neurovetenskap, K8 Neuro Pereira, 171 77, Stockholm, Sweden;* ^*2*^ *Department of Physics, University of Gothenburg, Gothenburg, Sweden;* ^*3*^ *Immunology and Respiratory Medicine Division, Department of Medicine Solna, Karolinska Institutet, Center for Molecular Medicine, Karolinska University Hospital, Karolinska University Hospital, Solna, Stockholm, Sweden;* ^*4*^ *Department of Human Neurosciences, Sapienza University of Rome, Rome, Italy*

**Background and Aims:** Recently, two main Parkinson's disease (PD) phenotypes have been proposed: “body‐first,” where α‐synuclein pathology begins in the peripheral nervous system and spreads symmetrically from bottom‐up, and “brain‐first,” where pathology starts in the brain and spreads asymmetrically downwards. However, no studies have assessed these phenotypes across both prodromal and clinical PD stages, tracked their pathological progression in vivo or identified potential underlying biological mechanisms.

**Methods:** We analyzed 910 prodromal and 1120 clinical PD cases with comprehensive longitudinal clinical, imaging, and genetic data from the Parkinson Progression Marker Initiative over a 12‐year period.

**Results:** Both prodromal and clinical groups with body‐first symptoms exhibited worse longitudinal motor progression and attention decline compared to brain‐first cases. The body‐first and brain‐first phenotypes were stable over time, identifiable using unsupervised deep learning, and predicted conversion to clinical PD in prodromal cases. Additionally, body‐first cases displayed more pronounced changes in the caudal locus coerelius (LC), symmetrical alterations in the striatum and glymphatic system, consistent with the traditional Braak's bottom‐up progression of α‐synuclein pathology and the more symmetric distribution proposed for body‐first PD. In contrast, brain‐first cases exhibited changes in the rostral LC and asymmetric alterations in the striatum and glymphatic system, suggesting a top‐down progression. Genetic analysis identified new specific single‐nucleotide polymorphisms associated with PD phenotypes (e.g., TRIM40, IP6K2) linked to worse outcomes in prodromal cases.

**Conclusion:** Recognising body‐first and brain‐first PD as distinct entities with unique clinical, imaging, and genetic profiles paves the way for targeted and personalised therapeutic strategies that address the specific pathophysiological mechanisms of PD.

**Disclosure:** Nothing to disclose.

## OPR‐099

### Activity of brainstem nuclei of PD patients during noninvasive vagal nerve stimulation: An fMRI study

#### V. van Midden ^1^; A. Vovk^2^; R. Berlot^1^; Z. Pirtošek^1^; M. Kojović^1^

##### ^*1*^ *Department of Neurology, University Medical Centre Ljubljana, Ljubljana, Slovenia;* ^*2*^ *Centre of Clinical Physiology, Medical Faculty, University of Ljubljana, Ljubljana, Slovenia*

**Background and Aims:** Transcutaneous auricular vagus nerve electrostimulation (taVNS) is under investigation as add‐on therapy for Parkinson's disease (PD). fMRI studies in healthy cohorts show taVNS activates nucleus tractus solitarii (NTS) and locus coeruleus (LC). Whether this applies to PD is unknown. We aimed to quantify taVNS effects on LC and NTS activation in PD, using fMRI.

**Methods:** We enrolled 40 PD patients in our double‐blind, sham‐controlled, within‐subject, fMRI study (NCT05967598). 32 were analyzed. During a single ON‐medication visit, each participant received taVNS at 100 Hz (taVNS100), taVNS at 25 Hz (taVNS25), and sham, in randomized order during which fMRI was obtained. Activity of LC and NTS was analysed using LME models accounting for stimulation type, degeneration‐onset side (Deg_onset_side), and UPDRS‐III‐tertiles. After imaging, we assessed gait using motion sensors across stimulation conditions.

**Results:** NTS activation showed a main effect of taVNS type (*p* = 0.021) and an interaction with Deg_onset_side (*p* = 0.011), with taVNS100 increasing NTS activity versus taVNS25 (*p* = 0.018). In left‐onset PD, taVNS100 exceeded taVNS25 (*p* = 0.005) and sham (*p* = 0.029), while no NTS differences appeared in right‐onset PD. LC activity showed a stimulation‐type effect (*p* = 0.034), driven by reduced LC activity during taVNS100 versus sham (*p* = 0.033). UPDRS‐III‐tertiles did not affect nuclei responses. LC activity was predicted by NTS activity with a Deg_onset_side interaction (*p* = 0.007), showing a negative NTS–LC correlation in left‐onset PD. Stimulation had no effect on gait.

**Figure:**
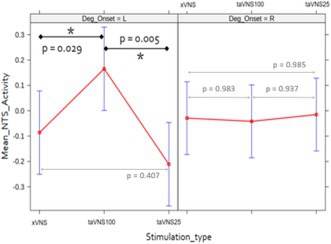
**FIGURE 1** In left‐onset PD, taVNS100 exceeded both taVNS25 (*p* = 0.005) and sham (*p* = 0.029). No NTS differences were detected in right‐onset PD.

**Figure:**
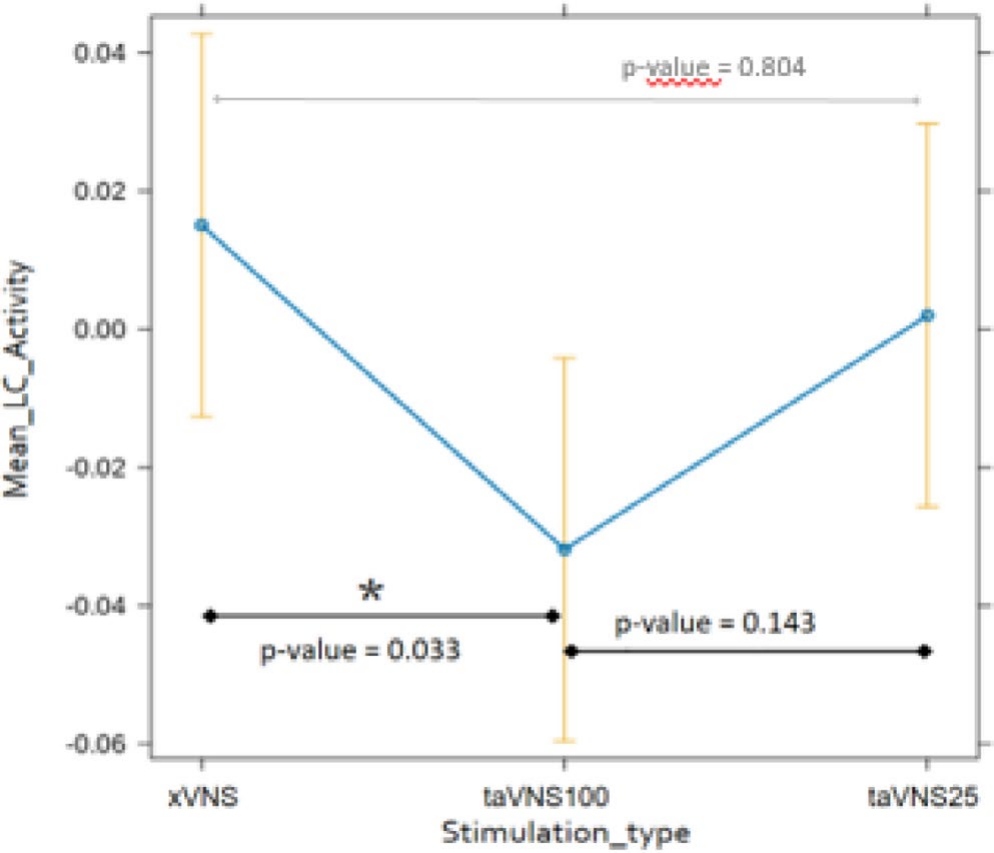
**FIGURE 2** LC activity was decreased during taVNS100 regardless of the side of the participants degeneration onset.

**Figure:**
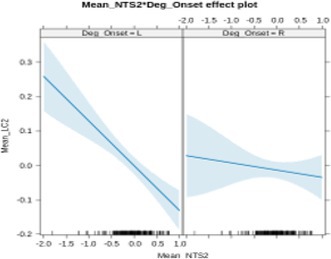
**FIGURE 3** Correlation between the NTS and LC ROI signal.

**Conclusion:** taVNS100 reduced LC activity and elevated NTS activity in patients with left Deg_onset_side. This suggests taVNS has PD specific effects on brainstem‐nuclei activation, which are driven by disease asymmetry.

**Disclosure:** Nothing to disclose.

## Cerebrovascular Diseases 1

## OPR‐100

### Genetic determinants of apixaban plasma exposure and clinical outcomes in ischemic stroke patients

#### A. Olšerová ^1^; T. Šrámková^1^; J. Paulasová Schwabová^1^; S. Kmetonyová^1^; K. Benešová^1^; J. Macek^2^; P. Janský^1^; V. Matoška^3^; A. Tomek^1^

##### ^*1*^ *Department of Neurology, Second Faculty of Medicine, Charles University, Motol University Hospital, Prague, Czech Republic;* ^*2*^ *Pharmakl s.r.o., Prague, Czech Republic;* ^*3*^ *Laboratory of molecular diagnostics, Hospital Na Homolce, Prague, Czech Republic*

**Background and Aims:** Apixaban is a direct factor Xa inhibitor used for stroke prevention in patients with atrial fibrillation. Its absorption and elimination are influenced by drug transporters and metabolic enzymes encoded by the ABCG2, ABCB1, CYP3A5, and SULT1A1 genes. Genetic polymorphisms in these pathways may contribute to variability in apixaban plasma exposure, but data in ischemic stroke patients remain limited. This study evaluated the impact of selected genetic polymorphisms on minimal apixaban plasma concentrations (Cmin) and clinical outcomes.

**Methods:** This retrospective monocentric study included consecutive ischemic stroke patients treated with apixaban. Genotyping for ABCG2 rs2231142, ABCB1 rs4148738, CYP3A5 rs776746, and SULT1A1 rs9282861 was performed, and Cmin was measured 12 h after administration. Clinical follow‐up assessed recurrent ischemic stroke, clinically relevant bleeding, thromboembolic events, and mortality.

**Figure:**
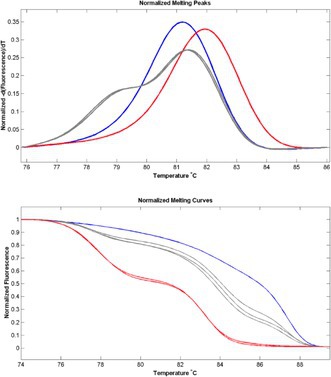
**FIGURE 1** ABCB1 genotyping – graphical visualization of melting curves using HRM analysis with the LightScanner Master Mix system, where red curves represent patients without the ABCB1 rs4148738 allele (wild type), grey curves indicate heterozygotes, and blue curve.

**Results:** Eighty‐three patients were included (53% male, mean age 70.4 years), with a mean follow‐up of 47.2 months (326 patient‐years); 37.3% received a reduced dose. Carriers of ABCG2 rs2231142 more frequently had apixaban plasma levels below the therapeutic range (*p* = 0.016). No significant associations were observed for ABCB1, CYP3A5, or SULT1A1, and genetic variants were not independently associated with clinical outcomes.

**TABLE 1** Distribution of genotypes and apixaban plasma levels in patients treated with apixaban.

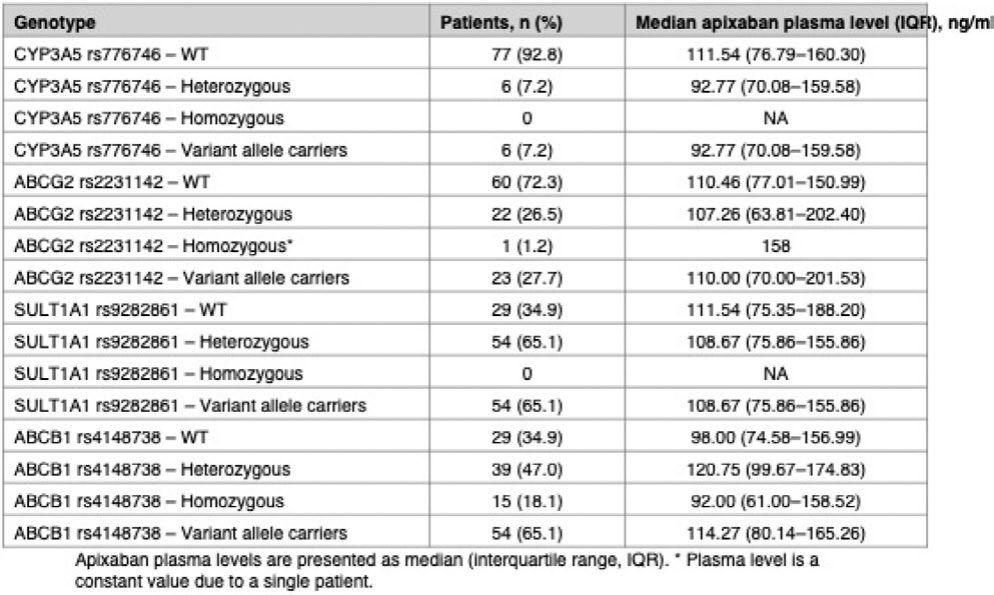

**Figure:**
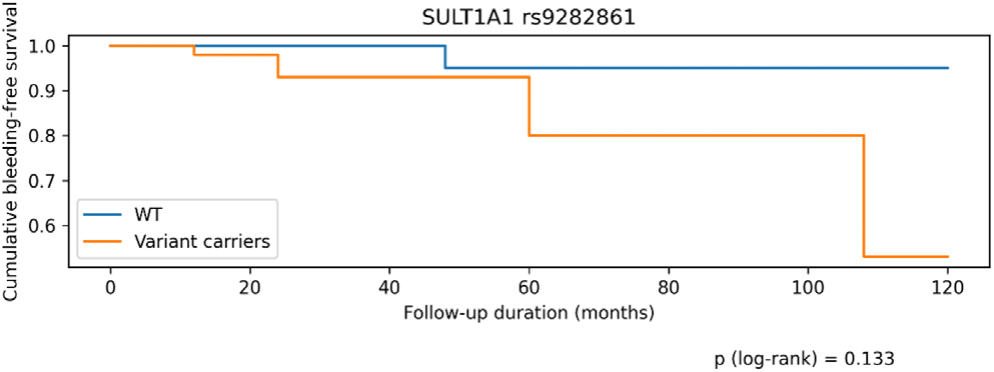
**FIGURE 2** Kaplan–Meier curve for the occurrence of bleeding complications in carriers of SULT1A1 rs9282861.

**Conclusion:** The ABCG2 rs2231142 polymorphism is associated with subtherapeutic apixaban plasma levels in ischemic stroke patients, suggesting a potential role for pharmacogenetic guidance of apixaban dosing.

**Disclosure:** Nothing to disclose.

## OPR‐101

### Streamline‐based lesion–symptom mapping reveals network substrates of post‐stroke disability

#### C. Weiller ^1^; E. Vaidelyte^2^; F. Hertel^1^; M. Reisert^3^; H. Urbach^4^; E. van den Hoven^1^; M. Rijntjes^1^

##### ^*1*^ *Department of Neurology, Freiburg, Baden‐Württemberg, Germany;* ^*2*^ *Department of Neurology & Neurorehabilitation, University Center for Medicine of Aging and Rehabilitation Basel, Felix Platter Hospital, Basel, Switzerland;* ^*3*^ *Department of Radiology, Freiburg, Baden‐Württemberg, Germany,* ^*4*^ *Department of Neurology, Freiburg, BW, Germany*

**Background and Aims:** Although many factors are associated with post‐stroke disability (including initial severity, age, education, co‐morbidity, and social support), the underlying anatomical determinants remain unclear.

**Methods:** We applied a novel method, whole‐brain streamline‐based lesion–symptom mapping (SLSM) (Figure 1) in 493 patients with stroke and a single ischaemic lesion on acute MRI (<1 week). This approach was used to delineate fibre bundles predicting the modified Rankin Scale (mRS) at the chronic stage (>3 months).

**Figure:**
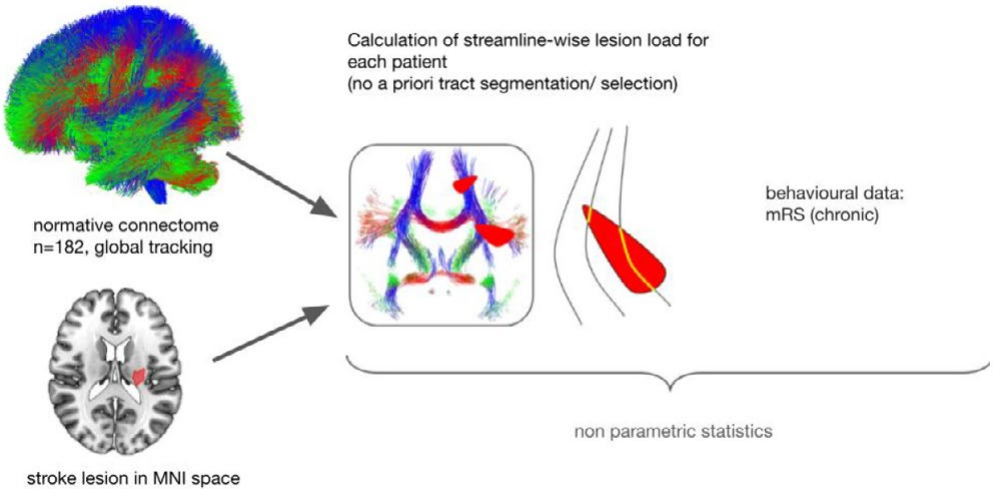
**FIGURE 1** Streamline‐based lesion symptom mapping (SLSM).

**Results:** SLSM identified afferent (spinothalamic) and efferent (corticospinal tract) projection fibres, interhemispheric transcallosal fibres, and three association fibre bundles as predictors of chronic mRS (Figure 2). The association bundles corresponded to dorsal and ventral pathways, as well as streamlines linking the temporal, parietal, and occipital lobes, consistent with the middle longitudinal fasciculus (“caudal pathway”).

**Figure:**
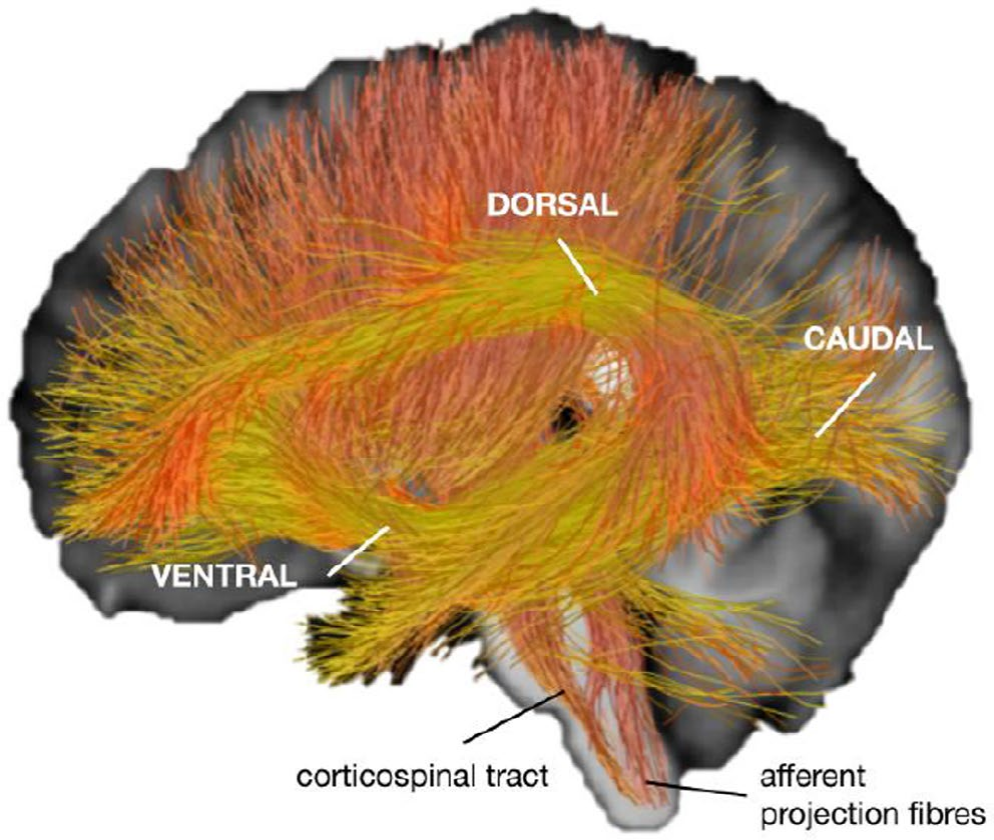
**FIGURE 2** Affected streamlines correlating with mRS (chronic).

**Conclusion:** SLSM is a new technique that constructs whole‐brain connectomes at the single‐streamline level without predefined tract templates. Disability after stroke is not only explained by damage to afferent and efferent projections. Dorsal and ventral pathways converge in frontal and posterior hubs to integrate sequential processing with deeper structural analysis (“syntax”). The caudal pathway supports multimodal integration in the angular gyrus and abstraction in the anterior temporal lobe (“semantics”). This framework enables abstraction and generalisation, crucial abilities when adapting to novel situations during recovery.

**Disclosure:** CW, FH, EvdH, MR are supported by EBRAINS2.0.

## OPR‐102

### Characteristics and causal factors of breakthrough ischemic stroke despite ongoing anticoagulation in atrial fibrillation: The ASPERA‐R study

#### F. De Santis ^1^; M. Foschi^2^; F. Gabriele^1^; L. D'Anna^3^; A. Zini^4^; A. Cascio Rizzo^5^; L. Pantoni^6^; G. Prandin^7^; F. Mele^8^; M. Valente^9^; M. Bagnato^10^; B. Casolla^11^; L. Gonzalez^12^; P. Candelaresi^13^; D. Aguiar de Sousa^14^; P. Caliandro^15^; A. Abdelalim^16^; L. Zhang^17^; A. El Bassiouny^18^; F. Ferrari^19^; M. Guarino^20^; G. Rinaldi^21^; G. Frisullo^22^; M. Mannino^23^; M. Caggiula^24^; A. Fonseca^25^; B. Antunes^26^; H. Budincevic^27^; G. Viticchi^28^; L. Barba^29^; P. Lochner^30^; S. Buddha^31^; M. Piscaglia^2^; M. Zedde^32^; A. Nasreldein^33^; L. Vinciguerra^34^; L. Costa^35^; A. Elsayed^36^; M. AlBanna^37^; L. Tudisco^38^; M. Mosconi^39^; G. Merlino^40^; A. Polymeris^41^; R. Ornello^1^; S. Sacco^1^

##### ^*1*^ *Department of Biotechnological and Applied Clinical Sciences, University of L'Aquila, L'Aquila, Italy;* ^*2*^ *Department of Neurosciences, Stroke Unit, Neurology Unit, S.Maria delle Croci Hospital, AUSL Romagna, Ravenna, Italy;* ^*3*^ *Department of Brain Sciences, Imperial College London, London, UK;* ^*4*^ *IRCCS Istituto delle Scienze Neurologiche di Bologna, Department of Neurology and Stroke Center, Maggiore Hospital, Bologna, Italy;* ^*5*^ *Department of Neurology and Stroke Unit, ASST Grande Ospedale Metropolitano Niguarda, Milan, Italy;* ^*6*^ *Neuroscience Research Center, Department of Biomedical and Clinical Sciences, University of Milan, Milan, Italy;* ^*7*^ *Clinical Unit of Neurology, Department of Medicine, Surgery and Health Sciences, University Hospital and Health Services of Trieste, ASUGI, University of Trieste, Trieste, Italy;* ^*8*^ *Neurology Unit, University Hospital Luigi Sacco, Milan, Italy;* ^*9*^ *Clinical Neurology, DMED, University of Udine, Italy;* ^*10*^ *UOC Stroke Unit e Neurologia, Ospedale Fabrizio Spaziani, Frosinone, Italy;* ^*11*^ *Stroke Unit, CHU Pasteur 2, Université Cote d'Azur, UMR2CA URRIS, Nice, France;* ^*12*^ *Stroke Center and Department of Neurology. Hospital La Paz Institute for Health Research (La Paz University Hospital‐Universidad Autónoma de Madrid). Madrid, Spain;* ^*13*^ *UOC Neurologia e Stroke Unit, AORN Cardarelli, Napoli, Italy;* ^*14*^ *Stroke Center, Department of Neurosciences, Centro Hospitalar Universitário Lisboa Central – ULS São José, Faculty of Medicine, University of Lisbon, Lisbon, Portugal;* ^*15*^ *Department of Neuroscience, Catholic University of the Sacred Hearth, Rome, Italy; UOC Neurology, Department of Neuroscience, Sensory Organs, and Thorax, Fondazione Policlinico Universitario A. Gemelli IRCCS, Rome, Italy;* ^*16*^ *Cairo University Stroke Center, Department of Neurology, Faculty of Medicine, Cairo University, Egypt;* ^*17*^ *Department of Neurology, St George's University Hospital, London, UK;* ^*18*^ *Neurology Department, Faculty of Medicine, Ain Shams University, Cairo, Egypt;* ^*19*^ *Department of Emergency Neurology and Stroke Unit, IRCCS Mondino Foundation, Pavia, Italy;* ^*20*^ *IRCCS Istituto delle Scienze Neurologiche di Bologna, Italy;* ^*21*^ *S.C. Neurologia, Ospedale “Di Venere”, Bari, Italy;* ^*22*^ *Emergency Neurology – Department of Neuroscience, Sensory Organs, and Thorax – Policlinico Universitario Agostino Gemelli, IRCCS Rome, Italy;* ^*23*^ *UOC Neurologia e Stroke Unit, AOOR Villa Sofia‐Cervello, Palermo, Italy;* ^*24*^ *UOC Neurologia e Stroke Unit, PO Vito Fazzi, Lecce, Italy;* ^*25*^ *Neurology Department, Hospital de Santa Maria, Faculty of Medicine, University of Lisbon, Portugal;* ^*26*^ *Department of Neurology, Hospital de Santa Maria, Lisbon, Portugal;* ^*27*^ *Department of Neurology, Sveti Duh University Hospital, Zagreb, Croatia; Department of Neurology, Sveti Duh University Hospital, Zagreb, Croatia;* ^*28*^ *Neurological Clinic, Marche Polytechnic University, Ancona, Italy;* ^*29*^ *Department of Neurology, Martin‐Luther‐University Halle‐Wittenberg, Germany;* ^*30*^ *Department of Neurology, Saarland University Medical Center, Homburg, Germany;* ^*31*^ *Department of Stroke Medicine, Southmead Hospital, North Bristol NHS Trust, Bristol, UK;* ^*32*^ *Neurology Unit, Stroke Unit, Azienda Unità Sanitaria Locale‐IRCCS di Reggio Emilia, Italy;* ^*33*^ *Department of Neurology, Assiut University, Assiut, Egypt;* ^*34*^ *Department of Neurology and Stroke Unit, ASST Crema Hospital, Italy;* ^*35*^ *Department of Neurology, Local Health Unit of Alto Minho, Viana do Castelo, Portugal;* ^*36*^ *Neurology and Neurointervention Department, Kobry Elkoba Medical Complex, Cairo, Egypt;* ^*37*^ *Department of Neurology, National Neuroscience Institute, King Fahad Medical City, Riyadh, Saudi Arabia;* ^*38*^ *Stroke Unit, Careggi University Hospital, Florence, Italy;* ^*39*^ *Department of Internal and Cardiovascular Medicine, Santa Maria della Misericordia Hospital, Perugia, Italy;* ^*40*^ *SOSD Stroke Unit, Udine University Hospital, Italy;* ^*41*^ *Department of Neurology and Stroke Center, University Hospital Basel and University of Basel, Basel, Switzerland*

**Background and Aims:** Oral anticoagulants (OACs), including vitamin K antagonists (VKAs) and direct oral anticoagulants (DOACs), reduce ischemic stroke risk in atrial fibrillation(AF), yet 1%–2% of patients still experience breakthrough strokes annually, not fully explained only by poor adherence. Clarifying underlying mechanisms is essential to improve prevention strategies.

**Methods:** ASPERA (NCT06823466) is a multicenter ambispective study; this analysis includes the retrospective arm of AF patients with neuroimaging‐confirmed ischemic stroke despite ongoing therapeutic OAC between 2020 and 2025, with collection of baseline characteristics, comorbidities, treatments, and 90‐day outcomes.

**Results:** Among 1649 patients (median age 80.4 years, 52.2% females) most were treated with DOACs (77.3%). Causal factors for breakthrough ischemic stroke included drug‐to‐drug interactions potentially affecting OAC efficacy (26.4%), competing etiologies (24.3%), and active cancer (4.4%). Overall, 180 (10.9%) had more than one potential cause and 724 (43.9%) of patients had at least one of those factors. Patients with almost one identifiable factor had a higher prevalence (*p* < 0.01) of arterial hypertension (87.3% vs. 76.3%), diabetes mellitus (30.1% vs. 24.3%), and dyslipidemia (58.1% vs. 45.8%) and a higher rate of minor stroke (NIHSS ≤ 5) presentation (33.8% vs. 26.7%, *p* = 0.002) compared with those without. These patients also showed higher 90‐day mortality (22.7% vs. 18.4%, *p* = 0.032) and stroke recurrence rates (4.8% vs. 2.4%, *p* = 0.007).

**Conclusion:** Approximately half of patients experiencing ischemic stroke despite ongoing oral anticoagulation have an identifiable mechanism that may have contributed to the event. Improved recognition and characterization of these factors may help reduce the risk of breakthrough stroke and warrant investigation in prospective studies.

**Disclosure:** Nothing to disclose.

## OPR‐103

### Maraviroc for stroke recovery (MASTER): Protocol and status of a phase 2 double‐blind placebo‐controlled randomized clinical trial

#### N. Broc; G. Byczynski; E. Dirren; E. Carrera

##### Stroke Research Lab, Division of Neurology, Department of Clinical Neuroscience, Geneva University Hospital, Geneva

**Background and Aims:** Despite advances in acute stroke care, stroke remains the leading cause of acquired disability, and no pharmacological therapy is currently available to enhance recovery beyond the acute phase. Preclinical studies suggest that inhibition of the C‐C chemokine receptor 5 (CCR5) may promote functional recovery by enhancing peri‐infarct cortical plasticity. However, its role in human stroke recovery remains unknown.

**Methods:** MASTER (MAraviroc for STrokE Recovery) is a phase II, single‐centre, randomized, double‐blind, placebo‐controlled trial. Eighty patients with moderate, incomplete upper limb motor impairment will be enrolled within 7 days of acute ischemic stroke and randomized (1:1) to receive oral maraviroc (300 mg twice daily) or placebo for 90 days, in addition to standard rehabilitation. The primary outcome is upper limb motor recovery at day 90, assessed using the Fugl–Meyer Assessment for the Upper Extremity (FMA‐UE). Secondary outcomes include measures of motor skill learning and imaging markers of peri‐infarct plasticity at day 90, evaluated using MRI connectivity and spectroscopy. Additional clinical outcomes will be assessed longitudinally up to 6 months post‐stroke.

**Figure:**
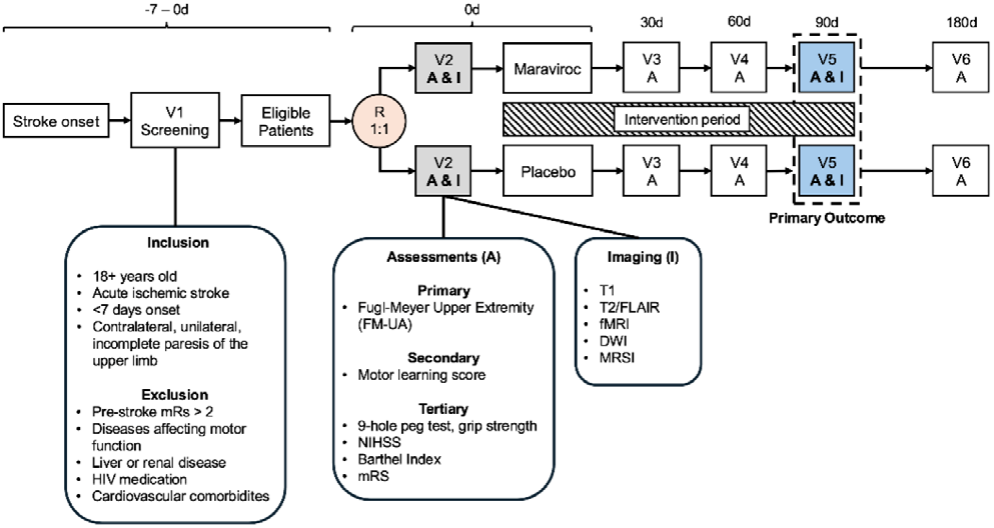
**FIGURE 1** Study schematic for the MASTER trial. Patients are included following verification of inclusion and exclusion criteria. Randomization (R) is 1:1 to placebo (Mannitol) or Maraviroc group.

**Results:** To date, 21 of 80 patients have been enrolled. Treatment compliance ranges from 69% to 100%. The cohort has a median age of 77 years, 43% are women, and median baseline NIHSS and FMA‐UE scores are 5 and 50, respectively. Eight participants have completed the primary outcome assessment.

**Figure:**
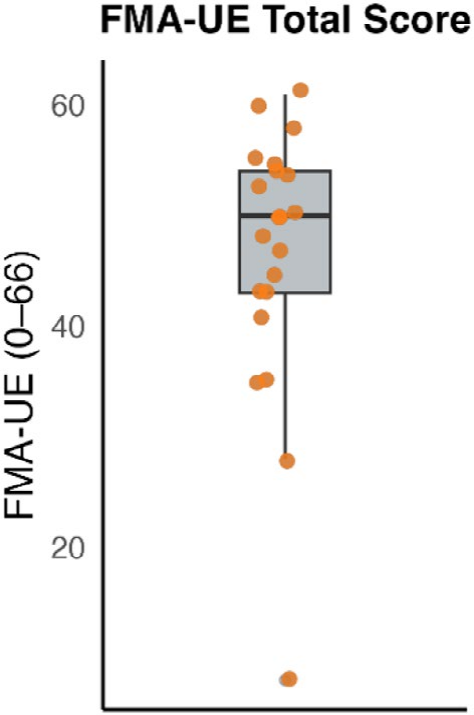
**FIGURE 2** Box‐plot of Fugl‐Meyer scores for the upper extremity (FMA‐UE) at baseline.

**Conclusion:** MASTER explores a novel pharmacological approach to enhance post‐stroke motor recovery and to investigate its underlying mechanisms with the help of advanced MRI.

**Disclosure:** EC is supported by the Swiss National Science Foundation (SNSF; Grant no 215285).

## OPR‐104

### Sex‐based outcomes after breakthrough ischemic stroke on oral anticoagulants for atrial fibrillation: ASPERA‐R inverse probability weighted analysis

#### P. Colantuono ^1^; M. Foschi^2^; L. D'Anna^3^; F. Gabriele^1^; R. Ornello^1^; A. Zini^4^; A. Cascio Rizzo^5,6^; L. Pantoni^7^; M. Bagnato^8^; B. Casolla^9^; B. Fuentes^10^; P. Candelaresi^11^; D. Aguiar De Sousa^12^; P. Caliandro^13^; A. Abdelalim^14^; L. Zhang^15^; A. El Bassiouny^16^; F. Ferrari^17^; M. Guarino^18^; G. Rinaldi^19^; G. Frisullo^20^; M. Mannino^21^; A. Fonseca^22^; H. Budincevic^23,24,25^; G. Viticchi^26^; L. Barba^27^; P. Lochner^28^; S. Buddha^29^; M. Piscaglia^30^; M. Zedde^31^; A. Nasreldein^32^; L. Vinciguerra^33^; A. Elsaid Elsayed^34^; M. Al Banna^35^; G. Merlino^36^; F. De Santis^1^; S. Sacco^1^

##### ^*1*^ *Department of Biotechnological and Applied Clinical Sciences, University of L'Aquila, L'Aquila, Italy;* ^*2*^ *Department of Neurosciences, Stroke Unit, Neurology Unit, S.Maria delle Croci Hospital, AUSL Romagna, Ravenna, Italy;* ^*3*^ *Department of Stroke and Neuroscience, Charing Cross Hospital, Imperial College London NHS Healthcare Trust, London, UK;* ^*4*^ *IRCCS Istituto delle Scienze Neurologiche di Bologna, Department of Neurology and Stroke Center, Maggiore Hospital, Bologna, Italy;* ^*5*^ *Department of Neurology and Stroke Unit, ASST Grande Ospedale Metropolitano Niguarda, Milan, Italy;* ^*7*^ *Neuroscience Research Center, Department of Biomedical and Clinical Sciences, University of Milan, Italy;* ^*8*^ *UOC Stroke Unit e Neurologia, Ospedale Fabrizio Spaziani, Frosinone, Italy;* ^*9*^ *Stroke Unit, CHU Pasteur 2, Université Cote d'Azur, UMR2CA URRIS, Nice, France;* ^*10*^ *Stroke Center and Department of Neurology. Hospital La Paz Institute for Health Research (La Paz University Hospital‐Universidad Autónoma de Madrid), Madrid, Spain;* ^*11*^ *UOC Neurologia e Stroke Unit, AORN Cardarelli, Napoli, Italy;* ^*12*^ *Stroke Center, Department of Neurosciences, Centro Hospitalar Universitário Lisboa Central, ULS São José, Faculty of Medicine, University of Lisbon, Lisbon, Portugal;* ^*13*^ *Department of Neuroscience, Catholic University of the Sacred Hearth, Rome, Italy; UOC Neurology, Department of Neuroscience, Sensory Organs, and Thorax, Fondazione Policlinico Universitario A. Gemelli IRCCS, Rome, Italy;* ^*14*^ *Cairo University Stroke Center, Department of Neurology, Faculty of Medicine, Cairo University, Egypt;* ^*15*^ *Department of Neurology, St George's University Hospital, London, UK;* ^*16*^ *Neurology Department, Faculty of Medicine, Ain Shams University, Cairo, Egypt;* ^*17*^ *Department of Brain and Behavioral Sciences, University of Pavia, Pavia, Italy;* ^*18*^ *IRCCS Istituto delle Scienze Neurologiche di Bologna, Italy;* ^*19*^ *S.C. Neurologia, Ospedale “Di Venere”, Bari, Italy;* ^*20*^ *Emergency Neurology, Department of Neuroscience, Sensory Organs, and Thorax, Policlinico Universitario Agostino Gemelli, IRCCS Rome, Italy;* ^*21*^ *UOC Neurologia e Stroke Unit, AOOR Villa Sofia‐Cervello, Palermo, Italy;* ^*22*^ *Neurology Department, Hospital de Santa Maria, Faculty of Medicine, University of Lisbon, Portugal;* ^*23*^ *Department of Neurology, Sveti Duh University Hospital, Zagreb, Croatia;* ^*26*^ *Neurological Clinic, Marche Polytechnic University, Ancona, Italy;* ^*27*^ *Department of Neurology, Martin‐Luther‐University Halle‐Wittenberg, Germany;* ^*28*^ *Department of Neurology, Saarland University Medical Center, Homburg, Germany;* ^*29*^ *Department of Stroke Medicine, Southmead Hospital, North Bristol NHS Trust, Bristol, UK;* ^*30*^ *Department of Neurosciences, Stroke Unit, Neurology Unit, S.Maria delle Croci Hospital, AUSL Romagna, Ravenna, Italy;* ^*31*^ *Neurology Unit, Stroke Unit, Azienda Unità Sanitaria Locale‐IRCCS di Reggio Emilia, Italy;* ^*32*^ *Department of Neurology, Assiut University, Assiut, Egypt;* ^*33*^ *Department of Neurology and Stroke Unit, ASST Crema Hospital, Italy;* ^*34*^ *Neurology and Neurointervention Department, Kobry El Kobba Medical Complex, Cairo, Egypt;* ^*35*^ *Department of Neurology, National Neuroscience Institute, King Fahad Medical City, Riyadh, Saudi Arabia;* ^*36*^ *SOSD Stroke Unit, Udine University Hospital, Italy*

**Background and Aims:** Sex‐specific outcomes after breakthrough ischemic stroke on oral anticoagulation (OAC) are unexplored. We compared 90‐day outcomes by sex and explored modifiers.

**Methods:** ASPERA‐R (NCT06823466) was an international, multicenter, retrospective study enrolling adults (>18 years) with breakthrough ischemic stroke on OAC for AF. Primary outcome was 90‐day return to baseline neurological (mRS 0–1 maintained if pre‐stroke 0–1; or same/lower mRS if pre‐stroke ≥2). Secondary outcome were 90‐day mRS shift, recurrent ischemic stroke/TIA, myocardial infarction, all‐cause and vascular death. Safety outcomes included 90‐day moderate‐to‐severe bleeding, intracranial hemorrhage (ICH), 24‐h hemorrhagic transformation, and 24‐h symptomatic ICH. We applied inverse probability weighting and regression models to compare outcomes. Prespecified subgroup analysis tested sex‐specific interactions.

**Results:** We included 1649 patients (women 52.2%; mean age 78.0 ± 10.7). Women were older (80.2 ± 9.6 vs. 76.3 ± 10.8; SMDunw = 0.376), had higher baseline NIHSS (13 [IQR 9–19] vs. 9 [IQR 4–17]; SMDunw = 0.227), and worse pre‐stroke mRS (SMDunw=0.237). After weighting, women were less likely to return to baseline neurological function (35.2% versus 42.7%; adjusted risk ratio 0.82, 95% CI 0.71–0.96; *p* = 0.015), had worse mRS distribution (adjusted OR 1.17, 95% CI 1.01–1.37; *p* = 0.043) and higher recurrent ischemic stroke/TIA (4.8% vs. 2.8%; adjusted HR 1.70, 95% CI 1.01–2.86; *p* = 0.045). Women showed a trend toward more moderate‐to‐severe bleeding (4.6% vs. 2.8%; adjusted HR 1.63, 95% CI 0.96–2.72; *p* = 0.070). Subgroup analyses revealed significant sex interactions for OAC type, competing etiology, endovascular treatment, and OAC restart.

**Conclusion:** Women had worse 90‐day outcomes than men after breakthrough ischemic stroke on OAC for AF, highlighting the need for sex‐aware management.

**Disclosure:** Prof Budincevic declares speaker's fees from Bayer, Boehringer Ingelheim, Viatris, Novartis, Pliva‐Teva, Abbott, Medis, Eli Lilly, Berlin Chemie, Pfizer, and Roche. Prof Pantoni declares consultancy fees from Medtronic, PIAM and Amicus. Prof Sacco reports compensation from Novartis for other services; compensation from Novo Nordisk for consultant services; compensation from Boehringer Ingelheim for consultant services; compensation from Teva Pharmaceutical Industries for consultant services; compensation from Allergan for consultant services; employment by Università degli Studi dell'Aquila; compensation from Novartis for consultant services; compensation from Allergan for consultant services; compensation from PFIZER CANADA INC for consultant services; compensation from Abbott Canada for consultant services; compensation from H. Lundbeck A S for consultant services; compensation from AstraZeneca for consultant services; and compensation from Eli Lilly and Company for consultant services. Dr Zini declares consulting and speaker fees from Bayer, Boehringer‐Ingelheim, Alexion, Daiichi Sankyo, Pfizer, PIAM, Amgen, fees for Advisory Board from Boehringer‐Ingelheim, Daiichi Sankyo, Bayer and Astra Zeneca, not related to this study. The other authors report no conflicts.

## OPR‐105

### Impact of cancer on outcomes following breakthrough ischemic stroke on oral anticoagulants for atrial fibrillation: Insights from the ASPERA‐R study

#### V. Cannizzo ^1^; M. Foschi^1^; F. de Santis^1^; F. Gabriele^1^; R. Ornello^1^; L. D'anna^2^; A. Zini^3^; A. Cascio Rizzo^4^; L. Pantoni^5^; M. Bagnato^6^; B. Casolla^7^; B. Fuentes^8^; P. Candelaresi^9^; D. Aguiar de Sousa^10^; P. Caliandro^11^; A. Abdelalim^12^; L. Zhang^13^; A. El Bassiouny^14^; F. Ferrari^15^; M. Guarino^16^; G. Rinaldi^17^; G. Frisullo^18^; M. Mannino^19^; A. Fonseca^20^; H. Budincevic^21^; G. Viticchi^22^; L. Barba^23^; P. Lochner^24^; S. Buddha^25^; M. Piscaglia^26^; M. Zedde^27^; A. Nasreldein^28^; L. Vinciguerra^29^; A. Elsaid Elsayed^30^; M. Al Banna^31^; G. Merlino^32^; S. Sacco^1^

##### ^*1*^ *Department of Biotechnological and Applied Clinical Sciences, University of L'Aquila, L'Aquila, Italy;* ^*2*^ *Department of Stroke and Neuroscience, Charing Cross Hospital, Imperial College London NHS Healthcare Trust, London, UK;* ^*3*^ *IRCCS Istituto delle Scienze Neurologiche di Bologna, Department of Neurology and Stroke Center, Maggiore Hospital, Bologna, Italy;* ^*4*^ *Department of Neurology and Stroke Unit, ASST Grande Ospedale Metropolitano Niguarda, Milan, Italy;* ^*5*^ *Neuroscience Research Center, Department of Biomedical and Clinical Sciences, University of Milan, Italy;* ^*6*^ *UOC Stroke Unit e Neurologia, Ospedale Fabrizio Spaziani, Frosinone, Italy;* ^*7*^ *Stroke Unit, CHU Pasteur 2, Université Cote d Azur, UMR2CA URRIS, Nice, France;* ^*8*^ *Stroke Center and Department of Neurology, Hospital La Paz Institute for Health Research (La Paz University Hospital‐Universidad Autónoma de Madrid), Madrid, Spain;* ^*9*^ *UOC Neurologia e Stroke Unit, AORN Cardarelli, Napoli, Italy;* ^*10*^ *Stroke Center, Department of Neurosciences, Centro Hospitalar Universitário Lisboa Central, ULS São José, and Faculty of Medicine, University of Lisbon, Lisbon, Portugal;* ^*11*^ *Department of Neuroscience, Catholic University of the Sacred Hearth, Rome, Italy; UOC Neurology, Department of Neuroscience, Sensory Organs, and Thorax, Fondazione Policlinico Universitario A. Gemelli IRCCS, Rome, Italy;* ^*12*^ *Cairo University Stroke Center, Department of Neurology, Faculty of Medicine, Cairo University, Egypt;* ^*13*^ *Department of Neurology, St George's University Hospital, London, UK;* ^*14*^ *Neurology Department, Faculty of Medicine, Ain Shams University, Cairo, Egypt;* ^*15*^ *Department of Brain and Behavioral Sciences, University of Pavia, Pavia, Italy;* ^*16*^ *IRCCS Istituto delle Scienze Neurologiche di Bologna, Italy;* ^*17*^ *S.C. Neurologia, Ospedale “Di Venere”, Bari, Italy;* ^*18*^ *Emergency Neurology, Department of Neuroscience, Sensory Organs, and Thorax, Policlinico Universitario Agostino Gemelli, IRCCS Rome, Italy;* ^*19*^ *UOC Neurologia e Stroke Unit, AOOR Villa Sofia‐Cervello, Palermo, Italy;* ^*20*^ *Neurology Department, Hospital de Santa Maria, Faculty of Medicine, University of Lisbon, Portugal;* ^*21*^ *Department of Neurology, Sveti Duh University Hospital, Zagreb, Croatia;* ^*22*^ *Neurological Clinic, Marche Polytechnic University, Ancona, Italy;* ^*23*^ *Department of Neurology, Martin‐Luther‐University Halle‐Wittenberg, Germany;* ^*24*^ *Department of Neurology, Saarland University Medical Center, Homburg, Germany;* ^*25*^ *Department of Stroke Medicine, Southmead Hospital, North Bristol NHS Trust, Bristol, UK;* ^*26*^ *Department of Neurosciences, Stroke Unit, Neurology Unit, S.Maria delle Croci Hospital, AUSL Romagna, Ravenna, Italy;* ^*27*^ *Neurology Unit, Stroke Unit, Azienda Unità Sanitaria Locale‐IRCCS di Reggio Emilia, Italy;* ^*28*^ *Department of Neurology, Assiut University, Assiut, Egypt;* ^*29*^ *Department of Neurology and Stroke Unit, ASST Crema Hospital, Italy;* ^*30*^ *Neurology and Neurointervention Department, Kobry El Kobba Medical Complex, Cairo, Egypt;* ^*31*^ *Department of Neurology, National Neuroscience Institute, King Fahad Medical City, Riyadh, Saudi Arabia;* ^*32*^ *SOSD Stroke Unit, Udine University Hospital, Italy*

**Background and Aims:** Breakthrough ischemic stroke during oral anticoagulation (OAC) for atrial fibrillation (AF) remains a major therapeutic challenge, particularly in patients with cancer, who have competing thrombotic and bleeding risks. We investigated the impact of cancer on 90‐day outcomes after ischemic stroke during OAC.

**Methods:** We analyzed patients with AF experiencing ischemic stroke on continuous OAC enrolled in the international retrospective ASPERA‐R study (35 stroke centers, 9 countries). Inverse probability weighting adjusted baseline imbalances. Weighted Cox, ordinal logistic, and generalized linear models estimated adjusted 90‐day risks for primary (ischemic stroke or TIA), secondary (mRS shift, vascular/all‐cause death), and safety outcomes (moderate‐to‐severe bleeding, intracranial hemorrhage, 24‐h hemorrhagic transformation).

**Results:** Among 1649 patients (mean age 78.0 ± 10.7 years; 45.8% male), 247 (15.0%) had cancer (35.2% active, 64.8% remission). After weighting, cancer was associated with a higher 90‐day risk of new ischemic stroke or TIA (8.2% vs. 2.8%; aHR 2.56, 95% CI 1.59–4.13; *p* < 0.001) and worse mRS distribution (aOR 1.29, 95% CI 1.08–1.54; *p* = 0.005). Active cancer conferred a >4‐fold higher ischemic risk (HR 4.48, 95% CI 2.46–8.13; *p* < 0.001) and higher moderate‐to‐severe bleeding risk (HR 2.77, 95% CI 1.30–5.88; *p* = 0.008). Cancer in remission increased ischemic (HR 2.60, 95% CI 1.59–5.25; *p* = 0.001) but not bleeding risk. Hematological malignancies showed higher risks of ischemic events (HR 3.06, 95% CI 1.69–5.54; *p* = 0.001) and moderate‐to‐severe‐bleeding (HR 3.47, 95% CI 1.57–7.70; *p* = 0.006) than solid tumors.

**Figure:**
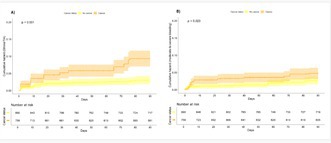
FIGURE 1

**Figure:**
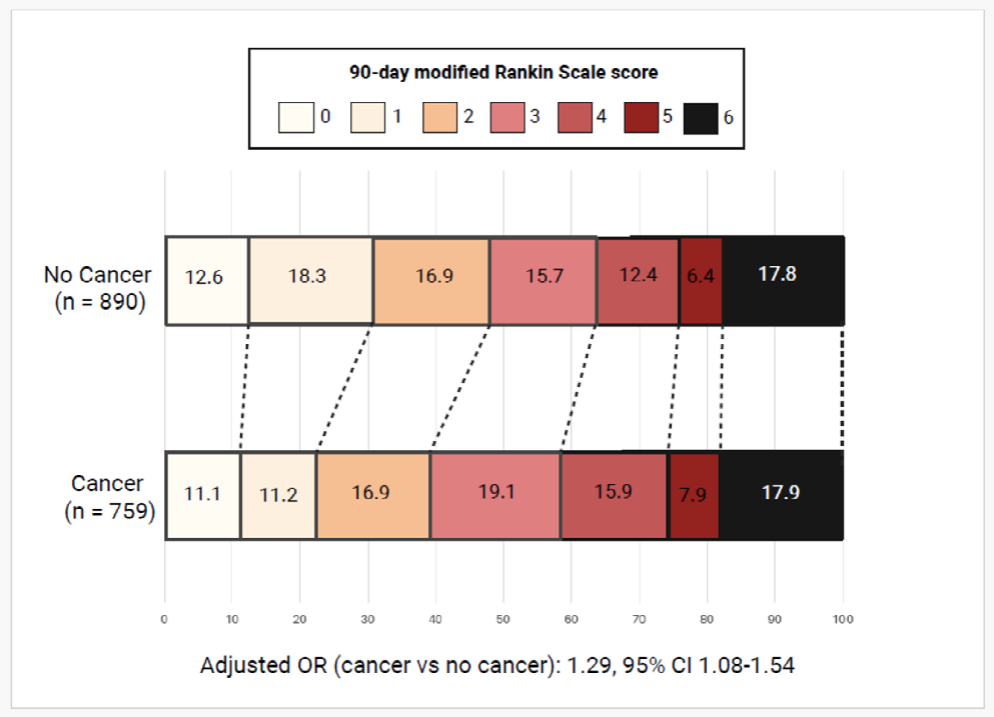
**FIGURE 2** Adjusted OR (cancer vs. no cancer): 1.29, 95% CI 1.08–1.54.

**Figure:**
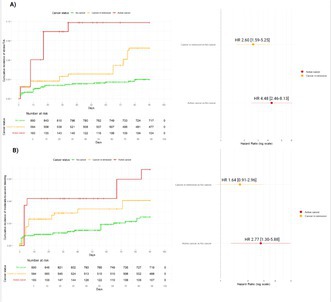
FIGURE 3

**Conclusion:** Cancer, particularly active and hematological malignancies, substantially worsens 90‐day prognosis after breakthrough stroke on OAC, underscoring the need for refined risk stratification and tailored secondary prevention.

**Disclosure:** Nothing to disclose.

## Neuroimmunology 2

## OPR‐106

### Neuronal antibody discovery in unexplained cerebellar ataxia: Preliminary results

#### J. Kerstens ^1^; E. Erdag Turgeon^1^; M. van Duijn^1^; S. Franken^1^; M. Nagtzaam^1^; M. Jin^1^; M. Rouvroye^2^; S. Veenbergen^3^; R. van Steenhoven^1^; J. de Vries^1^; P. Sillevis Smitt^1^; B. van de Warrenburg^4^; M. Titulaer^1^

##### ^*1*^ *Department of Neurology, Erasmus MC University Medical Center, Rotterdam, The Netherlands;* ^*2*^ *Department of Gastroenterology and Hepatology, Amsterdam University Medical Center, Amsterdam, The Netherlands;* ^*3*^ *Department of Immunology, Erasmus MC, University Medical Center Rotterdam, Rotterdam, The Netherlands;* ^*4*^ *Department of Neurology, Radboud University Medical Center, Nijmegen, The Netherlands*

**Background and Aims:** Over 40 autoantibodies associated to autoimmune cerebellar ataxia (ACA) have been discovered and this number is still growing. Antibody identification allows for definitive diagnosis and access to potentially beneficial immunotherapy. This is especially relevant for patients in whom an immune‐mediated cause is not initially suspected, for example because of a chronic rather than subacute disease course.

**Methods:** We retrospectively identified patients with suspected ACA tested at the national referral center for antibody diagnostics and autoimmune encephalitis (cohort A) and patients with unexplained chronic cerebellar ataxia seen at the largest tertiary ataxia referral center in the Netherlands (cohort B). Patients positive for known ACA‐associated antibodies were excluded. Serum or cerebrospinal fluid (CSF) samples were screened for antibodies by immunohistochemistry (IHC). Positive samples were tested on human induced pluripotent stem cell‐derived live neurons (LN). Samples positive with both techniques were grouped according to clinical features and IHC/LN staining patterns and evaluated by immunoprecipitation/mass spectrometry (IP/MS) to identify novel antibody targets.

**Results:** After exclusion of 235 patients who tested positive for known antibodies (all cohort A), we enrolled 351 patients in cohort A (229 serum and CSF, 99 serum, 23 CSF) and 143 in cohort B (all serum). IHC and LN were both positive in 11/351 (3.1%) and 3/143 (2.1%) patients, respectively. Four patients of the IHC/LN double positive subgroup in cohort A had similar staining patterns and were tested by IP/MS (analysis pending).

**Conclusion:** The search for novel ACA‐associated antibodies is not yet exhausted and should also include patients with a chronic disease course.

**Disclosure:** This study was funded by ZonMw (ACT‐MD, 09120232310026). JK was supported by a Research Mobility Fellowship from the European Joint Programme on Rare Diseases (EJP RD) and is currently funded by the Erasmus Trustfonds. PSS holds a patent for the detection of anti‐DNER and received research support from Euroimmun. MT has filed a patent, on behalf of the Erasmus MC, for methods for typing neurological disorders and cancer, and devices for use therein, and has received research funds for serving on a scientific advisory board of Horizon Therapeutics, for consultation at ArgenX, and for consultation at UCB. MT has received an unrestricted research grant from Euroimmun AG, and from CSL Behring. BvdW receives research support from ZonMw, Hersenstichting, Dutch Scientific Organization, Christina Foundation, and FARA; has served on scientific advisory board or did paid consultancy for Biogen, Biohaven, Vico Therapeutics; and receives royalties from BSL – Springer The other authors have nothing to disclose.

## OPR‐107

### Antibody fuctionalities during herpes‐simplex‐virus‐1 infection are associated with disease severity and predict post‐herpes autoimmune encephalitis

#### M. Spatola ^1^; L. Marmolejo^1^; C. Milano^1^; J. Planagumà^1^; E. Aguilar^1^; R. Bello‐Morales^2^; J. Dalmau^1^; T. Armanguè^3^

##### ^*1*^ *Neuroimmunology Program, Fundació de Recerca Clínic Barcelona‐Institut d'Investigacions Biomédiques August Pi i Sunyer (FRCB‐IDIBAPS), University of Barcelona, Spain and Caixa Research Institute (CRI), Barcelona, Spain;* ^*2*^ *Universidad Autonoma de Madrid, Madrid, Spain;* ^*3*^ *Neuroimmunology Program, FRCB‐IDIBAPS, University of Barcelona, Spain; Caixa Research Institute (CRI), Barcelona, Spain; and Pediatric Neuroimmunology Unit, Neurology Service, Sant Joan de Déu (SJD) Children's Hospital, University of Barcelona, Barcelona*

**Background and Aims:** Herpes‐simplex‐virus‐1 (HSV1) can cause recurrent cold sore (periphHSV) or encephalitis (HSE), which may be complicated by autoimmune encephalitis (postHSE‐AE). The immunological mechanisms underlying HSE and postHSE‐AE remain unclear.

**Methods:** We deeply characterized HSV1‐antibody responses in paired serum/CSF of 45 HSE patients (12 who developed postHSE‐AE, 33 who did not; at onset and 1 month), and in serum of 12 periphHSV. Profiles included Ig classes/sublasses, Fc receptor binding, and Antibody‐Dependent Cellular/Neutrophil Phagocytosis (ADCP/ADNP), Complement Deposition (ADCD) and NK‐cells activation (ADNKA). Antibody features were compared across groups and compartments, and correlated with disease severity and development of postHSE‐AE. Neuronal death was quantified in HSV1‐infected cultures treated with IgG from HSE, periphHSV or healthy controls.

**Results:** In HSE, HSV1‐specific responses in CSF were low at onset, but significantly increased at 1 month, while serum responses remained elevated. LASSO/PLSDA analyses identified ADCD in serum and ADCP in CSF as features that differed most across compartments. Higher CSF‐ADCP correlated with HSE severity. Compared to HSE, periphHSV patients showed higher serum ADNKA and FcgR3A binding despite similarly high IgG titers. Patients who developed PostHSE‐AE exhibited higher titers, ADCD, ADNP and FcgR binding. IgG from HSE patients caused increased neuronal death in‐vitro compared to IgG from periphHSV or healthy controls.

**Conclusion:** Our results indicate that, during HSE, HSV1‐responses are compartmentalized and characterized by phagocytosis‐activating antibodies in CSF that correlate with HSE severity and predict PostHSE‐AE. NK‐activating antibodies in periphHSV may protect the brain from HSE. Antibodies from HSE cause increased neuronal death, which likely promotes the development of autoimmune encephalitis.

**Disclosure:** Nothing to disclose.

## OPR‐108

### Frequent early cognitive impairment in both pediatric multiple sclerosis and myelin oligodendrocyte glycoprotein antibody‐associated disease

#### M. Buijze ^1^; S. Molenaar^1^; R. Verkerk^2^; M. Quist^1^; W. van der Vlugt^2^; R. Neuteboom^1^

##### ^*1*^ *Department of Neurology, Division of Pediatric Neurology, MS Center ErasMS, Erasmus Medical Center, Rotterdam, The Netherlands;* ^*2*^ *Department of Neurology, Division of Neuropsychology, Erasmus Medical Center, Rotterdam, The Netherlands*

**Background and Aims:** The characterization of early cognitive impairment (CI) in pediatric multiple sclerosis (MS) remains limited due to methodological heterogeneity across previous studies. Moreover, standardized data for pediatric Myelin Oligodendrocyte Glycoprotein Antibody‐Associated Disease (MOGAD) are scarce. Therefore, we aim to evaluate early cognitive dysfunction in both diseases and comprehensively characterize their neurocognitive profiles.

**Methods:** A cross‐sectional study of children with MS and MOGAD (excluding acute demyelinating encephalomyelitis) assessed neurocognition within 2 years after disease onset using a standardized, comprehensive test battery covering all cognitive domains. Test results were converted to *Z*‐scores. CI was defined as having *Z*‐scores below −1.5 in ≥2 domains. Children were compared according to CI status and domain specific impairment was evaluated using composite domain *Z*‐scores.

**Results:** Fifty‐one participants were analyzed (MS *n* = 31, MOGAD *n* = 20). Full‐scale intelligence was normal in both groups. Poor performance on the Bourdon–Vos Test indicated reduced sustained attention and processing speed. MS patients additionally showed lower scores on tasks of visuospatial processing and visuospatial memory. Overall, 48% of MS children and 45% of MOGAD children met criteria for CI. Domain‐specific impairments were most prominent in complex attention, with significantly reduced composite *Z*‐scores (MS: −1.26 ± 2.45, *p* < 0.001; MOGAD: −1.08 ± 2.13, *p* = 0.006). Only fatigue was significantly associated with the presence of CI (PedsQL fatigue *Z*‐score: −2.55 vs. −0.75, *p* = 0.02).

**Conclusion:** In both pediatric MS and MOGAD, early CI is prevalent with prominent difficulties in complex attention. Visuospatial functioning appears to be an additional vulnerable area for children with MS.

**Disclosure:** Michiel Buijze, Sandy Molenaar, Rosa Verkerk, Maud Quist and Willemijn van der Vlugt report no disclosures. Rinze Neuteboom participated in clinical trials with Roche, Novartis, Sanofi, Teva, Amgen and Livanova.

## OPR‐109

### Pathophysiology of pain in stiff person syndrome: An MRI and autoantibody study

#### S. Seefried ^1^; M. Pham^2^; C. Sommer^1^

##### ^*1*^ *Department of Neurology, University Hospital Würzburg, Würzburg, Germany;* ^*2*^ *Department of Neuroradiology, University Hospital Würzburg, Würzburg, Germany*

**Background and Aims:** Stiff‐person syndrome (SPS) is a rare autoimmune neurological disorder characterized by stiffness, spasms, anxiety, and pain. Pain is frequent but insufficiently understood and treated. Autoantibodies interfering with GABAergic inhibition may contribute to neuromuscular hyperexcitability and nociceptive processing. This study aimed to comprehensively characterize pain in SPS and to investigate its association with autoantibodies, peripheral nociceptor involvement, and central GABAergic dysfunction.

**Methods:** Thirty patients with SPS underwent detailed neurological examination and standardized pain assessment (NRS, GCPS, mRS). Structural brain MRI and proton MR spectroscopy of the insular cortex were performed. Serum autoantibodies were analyzed, peripheral blood mononuclear cells were stimulated to antibody‐producing B cells, and serum binding to rat spinal cord and brain sections was assessed.

**Results:** Patients reported high levels of acute and chronic pain. Greater serum binding to GABAergig interneurons in the spinal cord was associated with higher pain intensity. Burning and electrifying pain, predominantly affecting the back and legs, were frequently reported. SPS patients showed reduced cortical thickness in the bilateral superior frontal gyrus, which correlated negatively with pain severity. Insular GABA levels were reduced compared with healthy controls and showed an association with autoantibody titers.

**Conclusion:** Pain is a prominent and clinically relevant feature of SPS and appears to be linked to autoantibody‐associated peripheral nociceptor involvement and central GABAergic dysfunction. Improved understanding of pain mechanisms may support more targeted therapeutic strategies to reduce pain and disability in SPS.

**Disclosure:** Nothing to disclose.

## OPR‐110

### Long‐term outcomes in anti‐LGI1 encephalitis: A nationwide Dutch cohort study

#### T. Brand ^1^; Y. Crijnen^1^; A. van Sonderen^2^; M. de Bruijn^3^; J. Brenner^1^; T. Bienfait^1^; J. Kerstens^1^; R. van Steenhoven^1^; P. Sillevis Smitt^1^; S. Veenbergen^4^; J. de Vries^1^; M. Titulaer^1^

##### ^*1*^ *Department of Neurology, Erasmus MC, University Medical Centre Rotterdam, Rotterdam, The Netherlands;* ^*2*^ *Department of Neurology, Haaglanden Medical Centre, The Hague, The Netherlands;* ^*3*^ *Department of Neurology, Elisabeth Tweesteden Hospital, Tilburg, The Netherlands;* ^*4*^ *Department of immunology, Erasmus MC, University Medical Centre Rotterdam, Rotterdam, The Netherlands*

**Background and Aims:** Anti‐leucine‐rich glioma‐inactivated 1 (LGI1) encephalitis is one of the most prevalent autoimmune encephalitides, yet data regarding long‐term outcomes and treatment efficacy remain scarce. This study aims to assess long‐term clinical outcomes, characterize relapses and compare the efficacy of different therapeutic approaches.

**Methods:** In this nationwide cohort study, patients diagnosed with anti‐LGI1 encephalitis between August 2010 and January 2026 were included. Retrospective and prospective clinical data were collected. Primary outcomes included the modified Rankin Scale (mRS) and the Clinical Assessment Scale for Autoimmune Encephalitis (CASE).

**Results:** 215/240 patients were eligible for analysis. An interim analysis (*N* = 100) demonstrated a median follow‐up of 60 months (IQR 42–84; range 8–211). At least one relapse occurred in 24%, with significantly lower incidence among those treated with rituximab. Median time to first relapse following immunotherapy was 6 months (IQR 4–37; range 3–83). Relapses typically mirrored the symptoms of the initial episode, albeit with reduced severity: median maximal mRS during the initial disease episode was 3 (IQR 2–3; range 1–5), compared to 2 (IQR 2–3; range 2–3) at the time of first relapse. At 2 years post‐diagnosis, the median mRS score was 1 (IQR 1–2; range 0–3) and symptomatic epilepsy was present in 6% of patients.

**Conclusion:** This study will provide novel insights into long‐term prognosis. Ongoing analyses aim to identify predictors for relapse and functional outcome. Furthermore, we seek to provide guidance on optimal treatment strategies regarding immunotherapy and duration of anti‐seizure medication to improve patient care.

**Disclosure:** Peter A.E. Sillevis Smitt holds a patent for the detection of anti‐DNER and received research support from Euroimmun. Maarten J. Titulaer has filed a patent, on behalf of the Erasmus MC, for methods for typing neurological disorders and cancer, and devices for use therein, and has received research funds for EpilepsieNL (NEF 14‐19 & 19‐08), Dioraphte (20010403), ZonMW (Memorabel fellowship, VIMP scheme and Open Competition [ACT‐MD]), ItsME, Erasmus MC Foundation and Erasmus Trustfonds. MJT has served on a scientific advisory board of Horizon Therapeutics/AmGen, ArgenX and Arialys; for consultation at Guidepoint Global LLC, and at UCB. MT is co‐PI of the EXTINGUISH trial. MT has received an unrestricted research grant from Euroimmun AG, and from CSL Behring. MJT and Juliette Brenner, on behalf of ErasmusMC, have filed a copyright for the PROSE (Patient‐Reported Outcome Scale in Encephalitis). Jeroen Kerstens received a Research Mobility Fellowship from the European Joint Program on Rare Diseases (EJP‐RD) and funding from the Erasmus Trust Foundation. Robin W. Van Steenhoven is funded by ItsME foundation and Erasmus MC foundation. Juna M. de Vries is local investigator for the CIELO Satralizumab in anti‐NMDAR and anti‐LGI1 encephalitis (Roche). JV received the AEA Community seed grant in 2021 for the PROMISE study. The other authors report no relevant disclosures.

## OPR‐111

### Duchenne muscular dystrophy drives T cell remodeling

#### Y. Torrente ^1^; C. Villa^2^; A. Farini^1^

##### ^*1*^ *Neurology Unit, Fondazione IRCCS Ca' Granda Ospedale Maggiore Policlinico, Milan, Italy;* ^*2*^ *Stem Cell Laboratory, Department of Pathophysiology and Transplantation, Dino Ferrari Centre, Università degli Studi di Milano, Milan, Italy*

**Background and Aims:** Duchenne muscular dystrophy (DMD) is characterized by progressive muscle degeneration, yet systemic immunological consequences remain poorly understood. Recent fatalities following AAV‐mediated gene therapy highlight critical gaps in baseline immune dysfunction knowledge. This study aimed to comprehensively characterize systemic immune profiles in glucocorticoid‐free DMD patients using multi‐omic approaches.

**Methods:** We performed single‐cell transcriptomic and epigenomic profiling coupled with systemic cytokine analysis in glucocorticoid‐free DMD patients. Immune cell populations were characterized across bone marrow, dystrophic muscle, and peripheral blood compartments. Cell‐cell communication networks were analyzed, and findings were validated through meta‐analysis across independent cohorts.

**Results:** DMD exhibited distinct immune microenvironments: bone marrow harbored transcriptionally primed yet phenotypically naïve lymphocytes with stress‐responsive signatures (ATF3, FOSB) and metabolic reprogramming. Dystrophic muscle showed chronic T cell exhaustion with epigenetic imprinting. Paradoxically, circulating CD8+ memory T cells maintained TCF1+TIGIT− stem‐like features despite tissue exhaustion. Systemic profiling revealed elevated pro‐inflammatory mediators (IL‐6, TNF receptors, interferons) with impaired chemokine networks. Bone marrow networks centered on monocyte‐mediated T cell priming, while muscle networks involved FAP‐myofiber‐macrophage triads driving remodeling.

**Figure:**
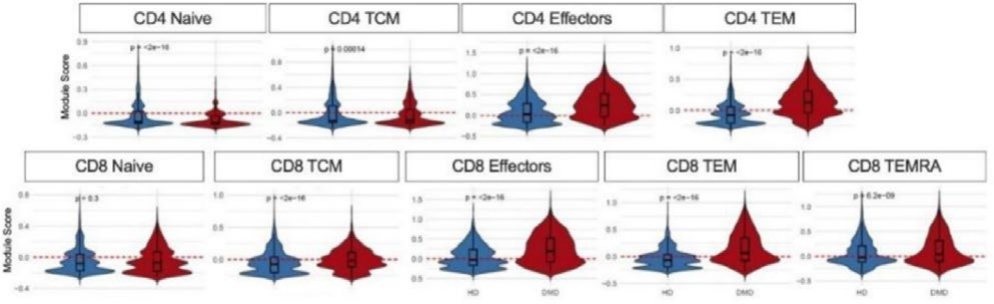
**FIGURE 1** DMD muscles showed marked increases in CD4+ and CD8+ T cells and myeloid dendritic cells, while B cells were notably absent, suggesting selective immune cell recruitment driven by the inflammatory muscle.

**Figure:**
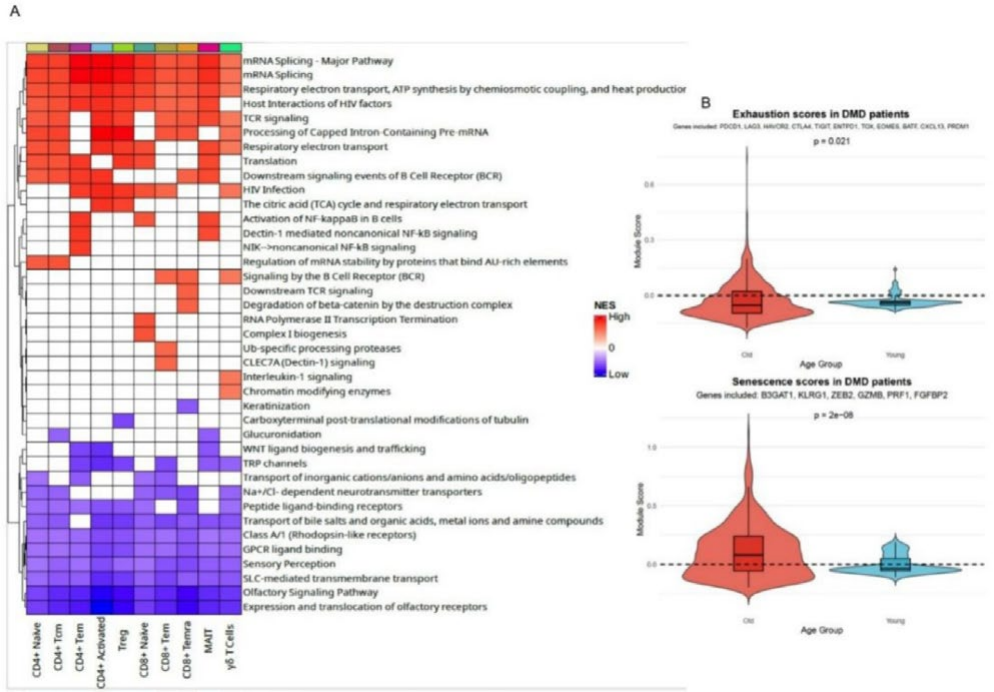
**FIGURE 2** DMD patients exhibited significantly higher T cell exhaustion scores.

**Figure:**
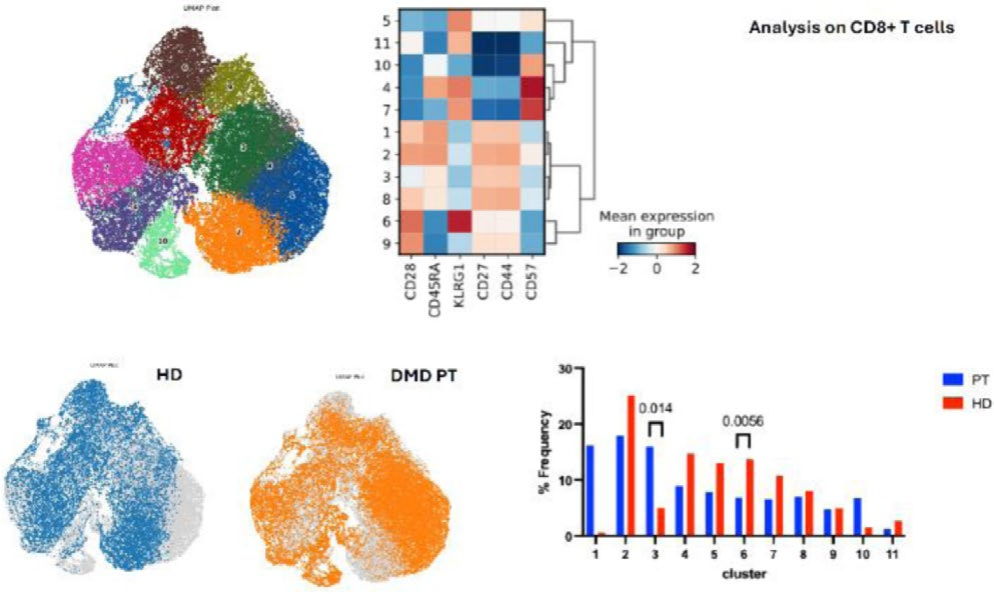
**FIGURE 3** Circulating CD8+ T cells from DMD patients and healthy controls, revealing specific T cell subsets expressing multiple markers of both exhaustion and senescence.

**Conclusion:** Pre‐existing immune priming in DMD—characterized by stress‐responsive transcription, enhanced OXPHOS metabolism, and preserved T cell stemness—may predispose patients to exaggerated therapeutic responses, providing essential groundwork for predicting complications and designing immunomodulatory strategies.

**Disclosure:** Nothing to disclose.

## Epilepsy 1

## OPR‐112

### AutoStim mode versus traditional vagus nerve stimulation in drug‐resistant epilepsy: Long‐term real‐world outcomes from a large vagus nerve stimulation cohort

#### C. Silvestri ^1^; C. Algoet^1^; K. Van Rooy^1^; L. Hogeveen^1^; E. Carrette^1^; S. Gadeyne^1^; A. Mertens^1^; F. Dewaele^2^; J. Vandersteene^2^; A. Meurs^1^; P. Boon^1^; K. Vonck^1^

##### ^*1*^ *4Brain, Department of Neurology, Reference Center for Refractory Epilepsy, Ghent University Hospital, Ghent, Belgium;* ^*2*^ *Department of Neurosurgery, Ghent University Hospital, Ghent, Belgium*

**Background and Aims:** Vagus nerve stimulation (VNS) is an adjunctive therapy for drug‐resistant epilepsy. Newer devices incorporating AutoStim deliver additional stimulation in response to ictal tachycardia (1–3). However, comparative data between AutoStim‐enabled and conventional VNS remain limited. This study compared long‐term clinical outcomes of VNS with AutoStim ON versus AutoStim OFF.

**Methods:** We conducted a retrospective observational cohort study of adults implanted with VNS between 2010 and 2024 at Ghent University Hospital (minimum follow‐up of 12 months) (Table 1). Patients were classified according to exposure to AutoStim during follow‐up. The AutoStim ON group included patients in whom AutoStim was activated at any time, while the AutoStim OFF group comprised patients treated exclusively with conventional VNS (ON: *n* = 142; OFF: *n* = 94). Outcomes included seizure frequency reduction, responder and best‐responder rates (≥50% and ≥90% reduction), seizure freedom, total daily antiseizure medication load (TDL), and mortality. Mixed‐effects negative binomial regression and analysis of covariance were applied.

**TABLE 1** Demographic description of the cohort. *Chi‐square and Mann‐Whitney tests applied. °8/21 (38.1%) patients had SE recurrence after VNS implantation on Open Loop Group, 11/40 (27.5%) in the Closed loop.

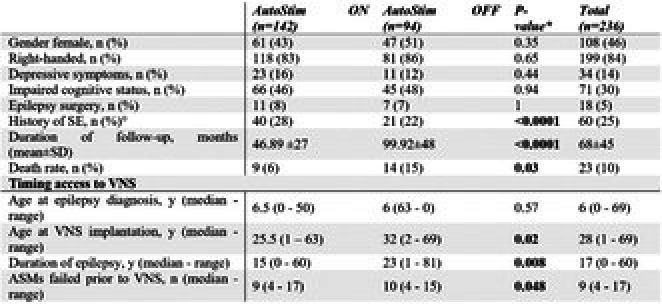

**Results:** Responder rates were higher in the AutoStim ON group (51% vs. 41%, *p* = 0.011), as were best‐responder rates (22% vs. 13%, *p* = 0.002) (Figure 1). Seizure freedom occurred in 17% versus 12% of patients, respectively (*p* > 0.05). Stimulation parameters did not differ significantly between groups (Table 2). Among responders, antiseizure TDL remained stable or decreased in the AutoStim ON group. Mortality was lower in the AutoStim ON group (6% vs. 15%, *p* = 0.03).

**Figure:**
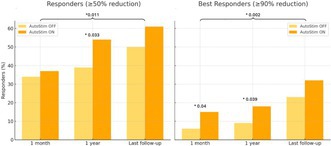
**FIGURE 1** Comparison of the percentage of responders (≥50% seizure reduction) and best responders (≥90% reduction) at 1 month, 1 year, and last follow‐up, with AutoStim ON versus OFF. Across all timepoints, AutoStim ON is associated with higher responder.

**TABLE 2** Comparison of VNS stimulation parameters between AutoStim ON and OFF groups.

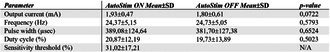

**Conclusion:** In this wide real‐world cohort, VNS with AutoStim ON was associated with better seizure outcomes, stable medication burden, and lower mortality compared with conventional VNS, supporting its use in drug‐resistant epilepsy (4,5).

**Disclosure:** Nothing to disclose.

## OPR‐113

### Medial pulvinar stereo‐electroencephalography biomarkers associated with deep brain stimulation response in focal drug‐resistant epilepsy

#### I. Bratu ^1^; R. Carron^2^; A. Clement^1^; S. Medina Villalon^1^; F. Bartolomei^1^; F. Pizzo^1^

##### ^*1*^ *Department of Epileptology and Cerebral Rhythmology, Timone Hospital, Marseille, France;* ^*2*^ *Medico‐surgical Unit of Epileptology, Functional and stereotactic neurosurgery, Timone Hospital, Marseille, France*

**Background and Aims:** Thalamic deep brain stimulation (DBS) represents an emerging therapeutic option for patients with focal drug‐resistant epilepsy who are ineligible for or have failed resective surgery. To optimize outcomes and guide DBS lead placement, thalamic stereo‐electroencephalography (SEEG) has been proposed.

**Methods:** This monocentric retrospective study aimed to identify interictal and ictal SEEG biomarkers of the medial pulvinar (PuM) associated with favourable PuM‐DBS response. Six patients (four female, two male) underwent SEEG including PuM sampling, were deemed unsuitable for resective surgery, and subsequently received bilateral PuM‐DBS. In total, eight PuM were sampled in SEEG: four bilaterally (two patients) and four unilaterally (four patients). The SEEG exploration covered both the PuM and the ipsilateral epileptogenic‐zone network (EZN) in four patients, whereas in the other two the EZN was bilateral but PuM sampling was unilateral. All SEEG signal analyses were performed on the PuM sampling contacts available in each patient. Interictal SEEG analysis included spike‐rates and non‐linear functional connectivity (h2), while ictal analyses combined visual inspection with quantitative biomarkers: Epileptogenicity Index (EI), Connectivity Epileptogenicity Index (cEI), Permutation Entropy Index (PEI) and Delta Entropy (ΔE).

**Results:** Two patients were responders (≥50% seizure reduction at 1 year). PuM spike‐rates, connectivity, EI and cEI values did not differentiate responders from non‐responders. In contrast, entropy‐based measures were significantly higher in responders: PEI (FDR‐p = 0.024) and ΔE (FDR‐p = 0.034).

**Conclusion:** Ictal PuM complexity disruption, quantified through entropy‐based SEEG metrics (PEI and ΔE), may represent a candidate biomarker of response to medial pulvinar DBS and warrants validation in larger cohorts.

**Disclosure:** Nothing to disclose.

## OPR‐114

### Acute brain injury in cryptogenic new onset refractory status epilepticus

#### S. Meletti^1^; A. Hanin^2^; G. Giovannini^1^; R. Bedin^1^; M. Burani ^1^; L. Taruffi^1^; N. Orlandi^1^; M. Malerba^1^; T. Urbano^1^; F. D'Achille^1^; M. Basha^3^; K. Eschbach^4^; B. Foreman^5^; R. Farias‐Moeller^6^; N. Gaspard^7^; E. Gerard^8^; T. Gofton^9^; M. Gopaul^10^; H. Haider^11^; S. Hantus^12^; S. Herman^13^; P. Kang^14^; G. Day^15^; P. Kandula^16^; C. Steriade^17^; A. Struck^14^; O. Taraschenko^18^; M. Wainwright^19^; J. Yeoun Yoo^20^; D. Zhou^21^; S. Lattanzi^22^; V. Navarro^2^; L. Hirsch^10^

##### ^*1*^ *Department of Biomedical, Metabolic and Neural Sciences, UNIMORE, Modena, Italy;* ^*2*^ *Sorbonne Université, Institut du Cerveau – Paris Brain Institute – ICM, Inserm, CNRS, AP‐HP, Hôpital de la Pitié Salpêtrière, Paris, France;* ^*3*^ *Department of Neurology, Wayne State University, Detroit, MI;* ^*4*^ *Department of Pediatrics, Section of Neurology, University of Colorado School of Medicine, Children's Hospital Colorado, Aurora, USA;* ^*5*^ *Division of Neurocritical Care, Department of Neurology and Rehabilitation Medicine, University of Cincinnati, OH;* ^*6*^ *Department of Neurology, Medical College of Wisconsin, Milwaukee, Wisconsin;* ^*7*^ *Department of Neurology, Hopital Universitaire de Bruxelles–Hopital Erasme, Universite Libre de Bruxelles, Belgium;* ^*8*^ *Department of Neurology, Feinberg School of Medicine, Northwestern University, Chicago, USA;* ^*9*^ *Department of Clinical Neurological Sciences, Schulich School of Medicine and Dentistry, Western University, London, Ontario, Canada;* ^*10*^ *Department of Neurology, Comprehensive Epilepsy Center, Yale University School of Medicine, New Haven, USA;* ^*11*^ *Department of Neurology, University of Chicago, Chicago, USA;* ^*12*^ *Epilepsy Center, Emory University School of Medicine, Atlanta, USA;* ^*13*^ *Epilepsy Center, Cleveland Clinic, Neurological Institute, Ohio, USA;* ^*14*^ *Department of Neurology, Washington University School of Medicine, St. Louis, USA;* ^*15*^ *Department of Neurology, Mayo Clinic, Jacksonville, USA;* ^*16*^ *Department of Neurology, Weill Cornell Medicine, New York City, USA;* ^*17*^ *Department of Neurology, NYU Langone Medical Center, New York City, USA;* ^*18*^ *Department of Neurological Sciences, University of Nebraska Medical Center, Omaha, USA;* ^*19*^ *Division of Pediatric Neurology, Seattle Children's Hospital, University of Washington, Seattle, USA;* ^*20*^ *Icahn School of Medicine at Mount Sinai, New York City, USA;* ^*21*^ *Department of Neurology, University of Pennsylvania, Philadelphia, USA;* ^*22*^ *Department of Experimental and Clinical Medicine, Neurology Clinic, Marche Polytechnic University, Ancona, Italy*

**Background and Aims:** New onset refractory status epilepticus (NORSE) is an extremely severe form of status epilepticus. We aim to assess how extensive is seizure‐induced acute brain damage in patients with cryptogenic NORSE (cNORSE) compared to etiology‐defined status epilepticus (eSE), chronic epilepsy, and healthy controls.

**Methods:** This international cross‐sectional cohort study includes 78 patients with cNORSE, 211 with eSE, 100 subjects with chronic epilepsy and 36 healthy participants. We measured neurofilament light chain (NfL) and S100‐beta (S100B), respectively for neuronal and astroglial damage, in serum and cerebrospinal fluid (CSF).

**Results:** In cNORSE, NfL concentrations were tenfold higher in CSF and fourfold higher in serum compared with eSE (*p* < 0.0001). Serum levels of NfL in cNORSE were twentyfold higher than in epilepsy and healthy controls' groups. Serum and CSF NfL values were strongly correlated (Spearman's *ρ* = 0.75, *p* < 0.001) and NfL levels rose sharply from week 1 to week 3 after cNORSE onset (*p* < 0.001). On the contrary, S100B concentrations in serum and CSF did not differ between cNORSE and eSE. NfL levels discriminated cNORSE from other cohorts with an area under the receiver operating characteristic (ROC) curve between 0.79 (95% CI 0.68–0.90) versus eSE and 0.99 (95% CI 0.78–1.00) versus non‐SE groups.

**Conclusion:** Acute neuroaxonal injury in cNORSE is extraordinarily severe, disproportionate to glial damage, and far greater than in other forms of status epilepticus. The rapid early rise in NfL highlights a narrow therapeutic window, underscoring the need for early, aggressive, and neuroprotective interventions.

**Disclosure:** This work includes data from project SE‐BIO funded by LICE (Lega Italiana Contro l'Epilessia) and PREDICT, project code GR‐2021‐12374594 funded by the Italian Ministry of Health. The NORSE/FIRES biorepository is supported by the Daniel Raymond Wong Neurology fund and the NORSE/FIRES Research Fund at Yale.

## OPR‐115

### Cenobamate in brain tumor‐related epilepsy: A multicenter real‐world experience

#### M. Romozzi ^1^; A. Mazzeo^2^; M. Antonucci^3^; G. Assenza^4^; M. Bassetti^5^; M. Fernandes^3^; C. Liguori^3^; G. Lippa^4^; M. Piccioli^6^; P. Pulitano^2^; P. Tisei^7^; M. Tombini^4^; C. Vollono^1^; P. Calabresi^1^; M. Maschio^8^

##### ^*1*^ *Department of Neuroscience; Università Cattolica del Sacro Cuore; Rome, Italy;* ^*2*^ *Department of Human Neurosciences, Sapienza University, Viale dell'Università, Rome, Italy;* ^*3*^ *Department of Systems Medicine, University of Rome Tor Vergata; Neurology Unit, University Hospital of Rome Tor Vergata, Rome, Italy;* ^*4*^ *UOC Neurologia, Fondazione Policlinico Universitario Campus Bio‐Medico, Via Alvaro del Portillo, Roma, Italy;* ^*5*^ *Neurologia, Azienda Ospedaliera San Giovanni Addolorata, Rome, Italy;* ^*6*^ *Neurologia Unità Operativa Complesse; Presidio Ospedaliero San Filippo Neri Hospital, ASL Roma 1, Rome, Italy;* ^*7*^ *Neurologia, Azienda Ospedaliera Sant'Andrea, Rome, Italy;* ^*8*^ *Neurorehabilitation Department, “Villa delle Querce” Clinic, Nemi, Rome*

**Background and Aims:** Brain tumor–related epilepsy (BTRE) is a disabling complication of brain tumors and often remains poorly controlled despite multiple antiseizure medications (ASMs). Cenobamate (CNB) showed high efficacy in drug‐resistant focal epilepsy, but evidence in BTRE is limited. This study evaluated the effectiveness and safety of CNB as add‐on therapy in a real‐world multicenter cohort of patients with BTRE.

**Methods:** We conducted a multicenter retrospective observational study across tertiary epilepsy centers in Italy. Adult patients with drug‐resistant BTRE treated with adjunctive CNB were included. Monthly seizure frequency was assessed at baseline and follow‐up. Outcomes were seizure frequency changes, responder rates, and safety. Effectiveness was analyzed in the intention‐to‐treat (ITT) population.

**Results:** Twenty patients (10 females; median age 48 years) were included. Tumor histologies were meningioma (35%), astrocytoma (20%), glioblastoma (15%), ganglioglioma (15%), oligodendroglioma (10%), and DNET (5%). Median follow‐up duration was 12 months (IQR: 9.8–12). In the ITT cohort (*n* = 20), monthly seizure frequency significantly decreased from a median of 6 seizures (IQR 18.75) at baseline to 1 seizure (IQR 2.55) at last follow‐up (*p* = 0.006). Median seizure reduction was 49.7%, with 50% of patients achieving a ≥50% reduction, 30% achieving a ≥75% reduction, and 20% becoming seizure‐free. One patient discontinued treatment before the 3‐month follow‐up, and one patient with glioblastoma died before the 6‐month follow‐up. Adverse events were predominantly mild to moderate in severity.

**Conclusion:** In this cohort of patients with drug‐resistant BTRE, CNB add‐on therapy was associated with a substantial reduction in seizure frequency and acceptable tolerability, supporting CNB as a promising option in this challenging population.

**Disclosure:** Nothing to disclose.

## OPR‐116

### Perampanel as only add‐on treatment in people with epilepsy and intellectual disability: Data from a real‐world study

#### R. Caridi ^1^; D. Abelardo^2^; A. Pascarella^1^; R. Cutellè^1^; G. Idone^1^; D. Carrozza^1^; V. Cianci^3^; S. Gasparini^1^; A. Iudice^4^; F. Bisulli^5^; P. Bonanni^6^; E. Caggia^7^; A. D'Aniello^8^; C. Di Bonaventura^9^; S. Beretta^10^; A. Stabile^10^; E. Domina^11^; F. Dono^12^; A. Gambardella^1^; A. Marelli^13^; S. Matricardi^14^; A. Morano^9^; R. Renna^15^; M. Piccioli^16^; P. Striano^17^; A. Orsini^18^; C. Torino^19^; U. Aguglia^1^; E. Ferlazzo^1^

##### ^*1*^ *Department of Medical and Surgical Sciences, Magna Græcia University of Catanzaro, Italy;* ^*2*^ *Regional Epilepsy Centre, Great Metropolitan “Bianchi‐Melacrino‐Morelli” Hospital, Reggio Calabria, Italy;* ^*3*^ *Neurology Unit, Great Metropolitan “Bianchi‐Melacrino‐Morelli Hospital”, Reggio Calabria, Italy;* ^*4*^ *Department of Neurosciences, Section of Neurology, University of Pisa, Pisa, Italy;* ^*5*^ *IRCCS Istituto delle Scienze Neurologiche di Bologna, Full Member of the European Reference Network for Rare and Complex Epilepsies (EpiCARE), Bologna, Italy;* ^*6*^ *Epilepsy and Clinical Neurophysiology Unit, Scientific Institute, IRCCS Eugenio Medea, Conegliano, Treviso, Italy;* ^*7*^ *Neurology Unit, Ospedale Giovanni Paolo II, Ragusa, Italy;* ^*8*^ *IRCCS Neuromed, Pozzilli, Italy;* ^*9*^ *Epilepsy Unit, Department of Human Neurosciences, “Sapienza” University of Rome, Rome, Italy;* ^*10*^ *Department of Neurology, Fondazione IRCCS San Gerardo dei Tintori, Monza, Italy;* ^*11*^ *U.C. Neurology, Ospedale Maggiore di Lodi ASST, Lodi, Italy;* ^*12*^ *Department of Neuroscience, Imaging and Clinical Science, “G. D'Annunzio” University of Chieti‐Pescara, Chieti, Italy;* ^*13*^ *Neurophysiopathology Unit, Epilepsy Center, San Salvatore Hospital, L'Aquila, Italy;* ^*14*^ *Department of Pediatrics, University of Chieti, Italy;* ^*15*^ *Neurological Clinic and Stroke Unit, AORN San Pio, Benevento, Italy;* ^*16*^ *UOC Neurology, PO San Filippo Neri, ASL Roma 1, Rome, Italy;* ^*17*^ *IRCCS Istituto Giannina Gaslini, full member of ERN‐EPICARE, Genova, Italy;* ^*18*^ *Pediatric Neurology, Pediatric Department, AOUP Santa Chiara Univeristy Hospital, Pisa, Italy;* ^*19*^ *Clinical Epidemiology and Physiopathology of Renal Diseases and Hypertension of Reggio Calabria, National Research Council, Institute of Clinical Physiology, Reggio Calabria, Italy*

**Background and Aims:** Epilepsy is markedly more prevalent in individuals with intellectual disability (ID) than in the general population and is often associated with drug resistance and increased adverse effects [1]. This study aimed to evaluate the efficacy and safety of perampanel (PER) as the sole add‐on therapy in patients with epilepsy and ID.

**Methods:** We performed a subanalysis of the PEROC study, an Italian multicenter, retrospective, longitudinal study assessing PER as the only add‐on treatment [2]. Patients were stratified according to ID status into three groups: no ID (no‐ID), mild ID (mID) and moderate/profound ID (mpID). Retention rate, responder rate (≥50% seizure reduction), seizure freedom and adverse events (AEs) were evaluated at 3, 6, and 12 months after PER introduction.

**Results:** The study included 356 patients: 277 no‐ID, 44 mID, 35 mpID. Retention rates were slightly lower in mpID (57.1%) than mID (78.6%) and no‐ID (72.7%), without significant differences (*p* < 0.05). Responder rates were comparable across groups, reaching 69.1%, in no‐ID, 72% in mID, 75% in mpID at 12 months (*p* = 0.848). Seizure‐freedom was higher in no‐ID compared with mID and mpID at 3 (38.1%, 21.4%, 17.4%; *p* = 0.046) and 6 (42.3%, 20.7%, 19.2%; *p* = 0.011) months, but no difference resulted at 12 months (46.3%, 40.5%, and 35%, respectively; *p* = 0.579). AEs (mainly irritability, dizziness, drowsiness) occurred in 28.7% no‐ID, 30.2% mID and 37.1% mpID patients, without significant difference (*p* = 0.585).

**Conclusion:** Perampanel as the only add‐on therapy showed good effectiveness and tolerability in patients with ID, achieving meaningful seizure control and satisfactory retention rates in this challenging population.

**Disclosure:** Nothing to disclose.

## OPR‐117

### Performance of ictal electrical source imaging from routine clinical EEG to define the epileptogenic zone

#### S. Lagarde ^1^; N. Roehri^2^; L. Spinelli^2^; C. Benar^3^; S. Momjian^4^; F. Bartolomei^5^; M. Seeck^2^; S. Vulliemoz^6^

##### ^*1*^ *EEG and Epilepsy Unit, University Hospitals and Faculty of Medicine, University of Geneva, Switzerland; Epileptology Department & Institute of Systems Neurosciences, University Hospitals of Marseille and Aix‐Marseille University, Marseille, France;* ^*2*^ *EEG and Epilepsy Unit, University Hospitals and Faculty of Medicine, University of Geneva, Geneva, Switzerland;* ^*3*^ *Institute of Systems Neurosciences, Aix‐Marseille University, Marseille France;* ^*4*^ *Neurosurgery Department, University Hospitals and Faculty of Medicine, University of Geneva, Switzerland;* ^*5*^ *Epileptology Department and Institute of Systems Neurosciences, University Hospitals of Marseille and Aix‐Marseille University, Marseille, France;* ^*6*^ *EEG and Epilepsy Unit, University Hospitals and Faculty of Medicine, University of Geneva, and CIBM, Centre D'Imagerie Biomédicale, Geneva, Switzerland*

**Background and Aims:** Epilepsy surgery is the treatment of choice for drug‐resistant focal epilepsy, provided the epileptogenic zone (EZ) is accurately identified. Electrical source imaging (ESI) allows three‐dimensional cortical projection of EEG activity and may improve EZ localisation. Ictal analysis is more specific than interictal analysis. This study evaluates the diagnostic performance of ictal ESI using a standard clinical EEG montage in presurgical assessment.

**Methods:** Seventy‐five patients underwent EEG recording with 37 electrodes. Individualised head models (BEM) were generated. Ictal ESI was performed using sLORETA and eLORETA. Seizure onset, time window (1–5 s), and frequency band were visually selected. Ictal power and dynamic functional connectivity (FC) were computed using Granger causality (sPDC). EZ was defined by the resection area or SEEG. Diagnostic performance was assessed using ROC analysis.

**Results:** A total of 213 seizures were analysed (median age 29 years); 73 patients underwent surgery, with 58.6% seizure‐free at follow‐up. MRI was positive in 54 cases, and 37 patients had mesiotemporal epilepsy. sLORETA and eLORETA showed comparable performance (AUROC 0.853 and 0.858, respectively). For eLORETA, sensitivity was 0.752, specificity 0.816, and accuracy 0.813. Performance was higher in seizure‐free patients and in mesiotemporal epilepsy. Diagnostic accuracy varied with discharge morphology, highest for sinusoidal rhythmic patterns. FC metrics showed limited added value (AUROC 0.66). Patients with poor surgical outcome exhibited a more diffuse ictal power distribution.

**Figure:**
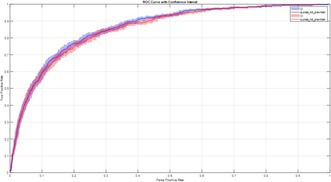
**FIGURE 1** ROC curve eLoreta vs. sLoreta, ictal discharge power.

**Figure:**
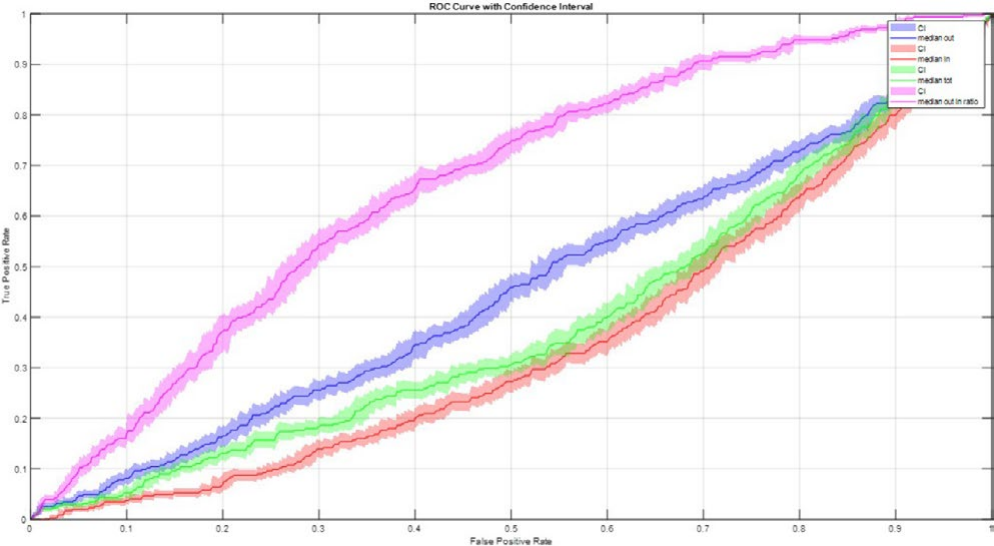
**FIGURE 2** ROC curve for directional ictal connectivity (sPDC) using eLoreta.

**Figure:**
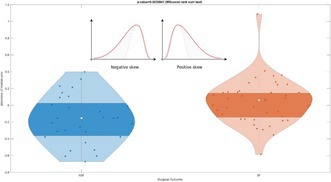
**FIGURE 3** Violin plot, skewness of ictal frequency power distribution in eLoreta, depending on whether the patient is seizure‐free or not.

**Conclusion:** Ictal ESI using standard EEG provides good accuracy for EZ localisation, particularly in seizure‐free patients. Power‐based ESI outperforms FC metrics, supporting its clinical relevance in presurgical evaluation.

**Disclosure:** Nothing to report.

## MS and Related Disorders 3

## OPR‐118

### Discontinuation of ocrelizumab in multiple sclerosis: Re‐occurrence of disease activity

#### F. Konen ^1^; F. Axhausen^2^; S. Wolff^2^; P. Mühlenbrock^2^; S. Gingele^1^; K. Jendretzky^1^; S. Nay^1^; L. Grote‐Levi^1^; P. Schwenkenbecher^1^; S. Meuth^3^; T. Skripuletz^1^; S. Pfeuffer^1^

##### ^*1*^ *Department of Neurology, Hannover Medical School, Hannover, Germany;* ^*2*^ *Department of Neurology, University Hospital of Giessen and Marburg, Justus‐Liebig‐University‐Giessen, Giessen, Germany;* ^*3*^ *Department of Neurology, University of Münster, Münster, Germany*

**Background and Aims:** Discontinuation of B‐cell depleting therapies such as ocrelizumab in people with multiple sclerosis (pwMS) is increasingly discussed due to infection risk and hypogammaglobulinemia. However, prospective data on disease reactivation, disability progression, and optimal treatment duration after cessation are limited.

**Methods:** In this prospective two‐centre observational cohort study, pwMS treated with ocrelizumab for ≥12 months without inflammatory disease activity in the preceding year were enrolled between 2018 and 2023. Patients who discontinued ocrelizumab were compared with continuers using 4:1 propensity score matching. Primary outcomes were time to combined inflammatory activity (CIA; relapse and/or new T2 lesions) and progression independent of relapse activity (PIRA). Receiver operating characteristic (ROC) analyses explored associations between treatment duration, discontinuation length, and disease activity.

**Results:** Among 655 eligible pwMS, 290 patients (58 discontinuers, 232 continuers) were included after matching. Median post‐discontinuation follow‐up was 28.5 months. Discontinuation was not associated with a significantly increased risk of CIA (hazard ratio [HR] 0.91, 95% CI 0.08–10.79) or PIRA (HR 4.8, 95% CI 0.38–60.2; Figure 1) compared with continuation. Disease activity remained largely stable during the first 24 months after discontinuation, with a numerical increase thereafter. ROC analyses suggested a plateau of anti‐inflammatory benefit after approximately 29–30 months of ocrelizumab treatment and a rising risk of disease reactivation beyond 30–32 months off‐treatment.

**Figure:**
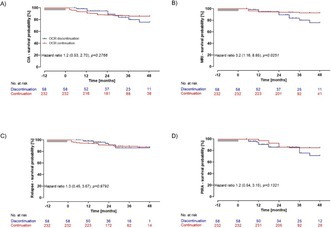
**FIGURE 1** Effectiveness outcomes following ocrelizumab discontinuation. Depicted are propensity score matched patients with multiple sclerosis with discontinuation (blue lines) and with continuation (red lines) of ocrelizumab treatment. Onset of (A) combi.

**Conclusion:** In clinically stable pwMS, ocrelizumab discontinuation after about 30 months appears safe in the short term for 25–43 months. Careful monitoring is warranted beyond 2 years off‐treatment to detect potential delayed disease reactivation.

**Disclosure:** The study received no external commercial or institutional funding. Outside the submitted work, some authors received honoraria for lectures, travel grants, or research grants. FFK received honoraria for lectures and travel compensation from Alexion, Argenx, Merck, Novartis, and Takeda. He received research support as fellow of the German Research Foundation (DFG)‐ and Hannover Medical School (MHH)‐funded Clinician Scientist Program (PRACTIS) at MHH and from Merck, Siemens and Erwin‐Röver‐Foundation. FA has no conflict of interest to report. SW received honoraria for lecturing from Mylan, Roche and Novartis and received research support from Novartis. PM has no conflict of interest to report. SG reports research support from Alnylam Pharmaceuticals, CSL Behring, Else Kröner Fresenius Foundation, Deutsche Forschungsgemeinschaft and Hannover Biomedical Research School (HBRS) and consulting and/or speaker honoraria from Alexion, Alnylam Pharmaceuticals, AstraZeneca, CSL Behring, GSK, Pfizer, Merck and Takeda Pharmaceuticals. KFJ received research support from Else Kröner Fresenius Foundation and travel compensation and congress fee from Neuraxpharm, Merck and Novartis. SN has no conflict of interest to report. LGL received a German Research Foundation (DFG)–funded fellowship as part of the Clinician Scientist Program at Hannover Medical School. PS has no conflict of interest to report. SGM received honoraria for lecturing, consulting, travel expenses for attending meetings, and research support from Academy 2, Argenx, Alexion, Almirall, Amicus Therapeutics Germany, AstraZeneca, Bayer Health Care, Biogen, BioNtech, BMS, Celgene, Datamed, Demecan, Desitin, Diamed, Diaplan, DIU Dresden, DPmed, Gen Medicine and Healthcare products, Genzyme, Hexal AG, IGES, Impulze GmbH, Janssen Cilag, KW Medipoint, MedDay Pharmaceuticals, Medmile, Merck Serono, MICE, Mylan, Neuraxpharm, Neuropoint, Novartis, Novo Nordisk, ONO Pharma, Oxford PharmaGenesis, QuintilesIMS, Roche, Sanofi, Springer Medizin Verlag, STADA, Chugai Pharma, Teva, UCB, Viatris, Wings for Life international, and Xcenda. TS reports research support from Alnylam, CSL Behring, Merck, Novartis, Siemens; honoraria for lectures, travel support for meeting attendance, and/or consultancy fees from Alexion, Alnylam, argenx, Bayer, Biogen, Bristol Myers Squibb, Centogene, CSL Behring, Grifols, Hexal AG, Horizon, Janssen, Merck, Novartis, Pfizer, Purpose Pharma, Roche, Sanofi, Siemens, SOBI, Teva, Viatris. SP received honoraria for lecturing and for serving on advisory boards from argenx, Alexion, Biogen, Hexal, Merck Healthcare, Novartis, Roche, and Sanofi Aventis; travel reimbursements from argenx, Alexion, Biogen, Merck Healthcare, neuraxpharm, and Roche; research support from Biogen, Merck Healthcare, and Novartis.

## OPR‐119

### Proinflammatory Epstein–barr virus antibody functions track with disease activity in multiple sclerosis

#### M. Behrens ^1^; P. Terroba‐Navajas^1^; E. Willemse^2^; S. Schädelin^2^; M. Comabella^3^; C. Münz^4^; G. Lauc^5^; Y. Bartsch^6^; J. Kuhle^2^; J. Lünemann^1^

##### ^*1*^ *Department of Neurology, University Hospital Münster, Germany;* ^*2*^ *Depa rtment of Neurology, University Hospital and University of Basel, Switzerland;* ^*3*^ *Servei de Neurologia, Centre d'Esclerosi Múltiple de Catalunya (Cemcat), Institut de Recerca Vall d'Hebron (VHIR), Hospital Universitari Vall d'Hebron, Universitat Autònoma de Barcelona, Spain;* ^*4*^ *Viral Immunobiology, Institute of Experimental Immunology, University of Zürich, Switzerland;* ^*5*^ *Faculty of Pharmacy and Biochemistry, University of Zagreb, Zagreb, Croatia;* ^*6*^ *Laboratory of Anti‐viral antibody‐omics, TWINCORE—Institute for Experimental Infection Research, Helmholtz Center for Infection Research (HZI) and Medical School Hannover (MHH), Germany*

**Background and Aims:** Epstein–Barr virus (EBV) has been implicated in the pathogenesis of multiple sclerosis (MS). Elevated immunoglobulin G (IgG) titers against EBV nuclear antigen 1 (EBNA1) represent the most consistent serological marker of MS risk, with levels remaining persistently elevated following disease onset.

**Figure:**
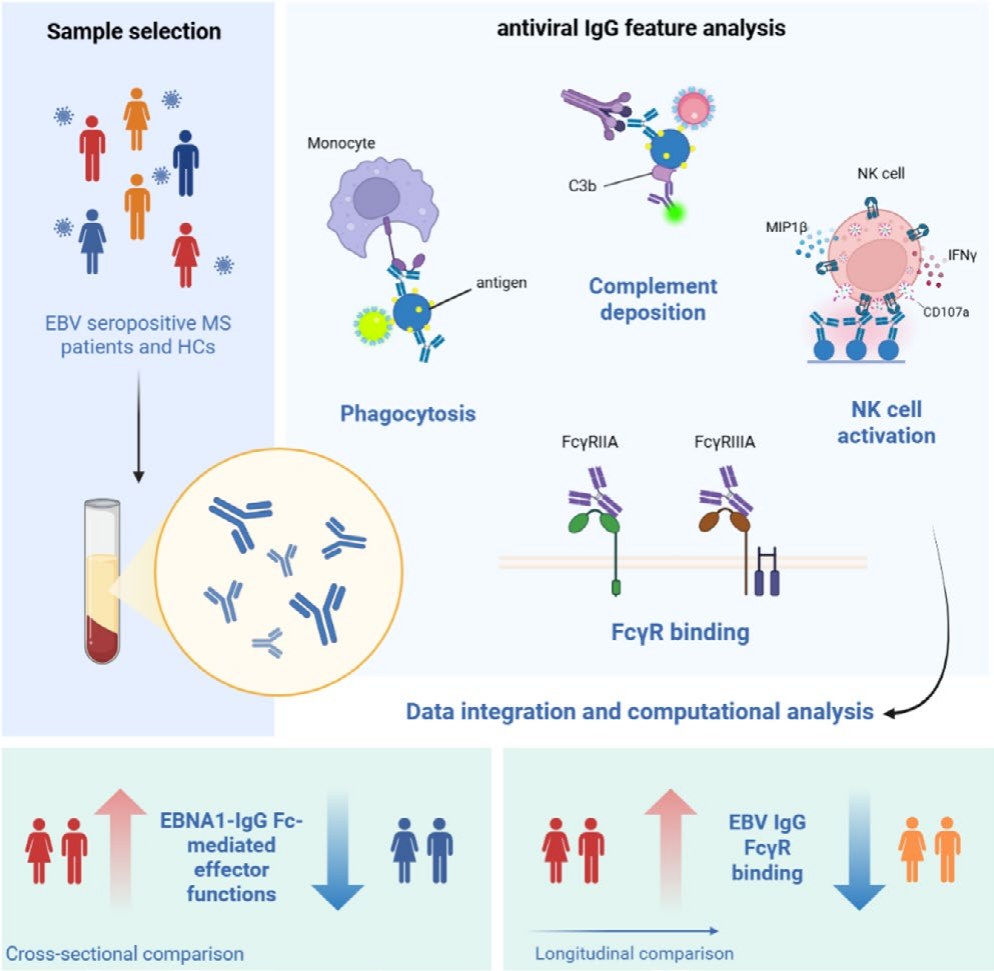
**FIGURE 1** Project Overview.

**Methods:** We conducted high‐dimensional profiling of virus‐specific antibody‐associated immune features in 60 individuals with relapsing‐onset MS followed over time and in 54 age‐ and sex‐matched EBV‐seropositive healthy controls.

**Results:** EBNA1‐specific IgG in MS patients exhibited a distinct proinflammatory Fc signature, characterized by increased affinity for Fcγ receptors (FcγRs) and enhanced Fc‐mediated effector functions, including antibody‐dependent phagocytosis, complement activation, and natural killer cell engagement. This signature was not observed for antibodies against other common viral antigens and showed a strong association with MS. Increased FcγR binding by IgG specific for both EBNA1 and the lytic EBV glycoprotein 350 correlated with disease activity, defined as clinical relapse and/or presence of contrast‐enhancing brain lesions.

**Conclusion:** Both Fab‐ and pro‐inflammatory functional Fc‐mediated features contribute to the association between EBNA1‐specific antibody responses and MS. Fc effector functions of EBV‐specific antibodies may actively participate in promoting focal disease activity in MS.

**Disclosure:** Nothing to disclose.

## OPR‐120

### T‐cell receptor excision circles and Kappa‐deleting recombination excision circles as biomarkers of immune aging in multiple sclerosis

#### M. Surano ^1^; A. Giordano^1^; S. Moylett^2^; P. Uibel^3^; F. Trogu^4^; M. Khademi^4^; M. Needhamsen^4^; R. Ouellette^4^; M. Flaskamp^3^; C. Gasperi^3^; M. Mühlhau^3^; A. Goris^2^; B. Dubois^2^; M. Sorosina^1^; M. Rocca^1^; M. Filippi^1^; S. D'Alfonso^5^; N. Barizzone^5^; M. Tosi^5^; A. Gajofatto^6^; C. Bosa^7^; D. Vecchio^8^; A. Gallo^9^; S. Gustavsen^10^; A. Olsson^10^; H. Søndergaard^10^; A. Gyllenberg^4^; H. Harbo^11^; T. Berge^12^; F. Martinelli‐Boneschi^13^; S. Llufriu^14^; P. Villoslada^14^; J. Saarela^15^; T. Granberg^4^; F. Piehl^4^; F. Esposito^1^; M. Jagodic^4^; B. Hemmer^16^; I. Kockum^4^

##### ^*1*^ *Department of Neurology, Università Vita‐Salute San Raffaele, Italy;* ^*2*^ *Department of Neurology, KU Leuven, Leuven, Belgium;* ^*3*^ *Department of Neurology, Technical University of Munich, Munich, Germany;* ^*4*^ *Department of Clinical Neuroscience, Neuroimmunology Unit, Karolinska Institutet, Stockholm, Sweden, and Karolinska University Hospital/Academic Specialist Clinic, SLSO, Stockholm, Sweden;* ^*5*^ *Department of Health Sciences, UPO University, Novara, Italy;* ^*6*^ *Dipartimento di neuroscienze, biomedicina, e movimento, Università di Verona, Verona, Italy;* ^*7*^ *MS Center, Department of Neuroscience and Mental Health, City of Health and Science University Hospital of Torino, Turin, Italy;* ^*8*^ *MS Center, AOU Maggiore della Carità, Novara Italy;* ^*9*^ *MS Center, I Division of Neurology, Department of Advanced Medical and Surgical Sciences (DAMSS), University of Campania Luigi Vanvitelli, Naples, Italy;* ^*10*^ *Department of Neurology, Neurocentret Rigshospitalet, Denmark;* ^*11*^ *Department of Neurology, Oslo University Hospital and University of Oslo, Oslo, Norway;* ^*12*^ *Department of Research, Innovation and Education, Oslo University Hospital and Department of Mechanical, Electronic and Chemical Engineering, Oslo Metropolitan University, Oslo, Norway;* ^*13*^ *Neurology Unit and Multiple Sclerosis Center, ASST Santi Paolo e Carlo, University of Milan, Italy;* ^*14*^ *Department of Neurology, Hospital Clínic de Barcelona, IDIBAPS, Spain;* ^*15*^ *Center for Molecular Medicine Norway (NCMM), Oslo, Norway;* ^*16*^ *Department of Neurology, Technical University of Munich and Munich Cluster for Systems Neurology (SyNergy), Munich, Germany*

**Background and Aims:** Kappa‐deleting recombination excision circles (KRECs) and T‐cell receptor excision circles (TRECs) are measures of newly generated B‐ and T‐cells respectively. Given their potential as immune aging biomarkers, we evaluated their association with disease activity and disability in people with Multiple Sclerosis and assessed their relationship with Disease‐Modifying Therapies (DMTs).

**Methods:** We included treatment‐naive relapsing–remitting MS patients from a 24‐month prospective multicenter study involving 9 European centers (MultipleMS). TRECs (*n* = 471) and KRECs (*n* = 472) levels were determined by qPCR at baseline and at 1‐year. Associations with age, baseline Expanded Disability Status Scale (EDSS), and time to first relapse in the 24 months before and after baseline were assessed through linear models and Cox regression. Longitudinal TRECs and KRECs changes under different DMTs and in untreated patients were assessed by Wilcoxon tests on cell‐count–adjusted values.

**Results:** Both TRECs (*p* = 4.65 × 10^−13^) and KRECs (*p* = 0.004) significantly decreased with age, with a stronger effect observed for TRECs. Neither TRECs nor KRECs correlated with baseline EDSS, although a negative trend was observed in patients with EDSS < 3.0. No associations were found with time to first relapse. DMTs had distinct effects on immune output: TRECs significantly decreased under fingolimod, remained stable with alemtuzumab and cladribine, and increased with other therapies at 1 year. KRECs declined markedly under fingolimod and anti‐CD20 therapies, more mildly under dimethyl fumarate and teriflunomide, and increased under natalizumab.

**Figure:**
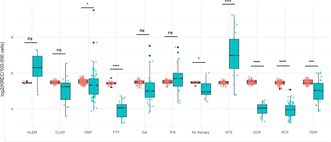
**FIGURE 1** Longitudinal KRECs changes under different DMTs at baseline and after 12 months.

**Figure:**
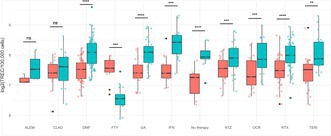
**FIGURE 2** Longitudinal TRECs changes under different DMTs at baseline and after 12 months.

**Conclusion:** TRECs and KRECs primarily reflect immune ageing in MS and are differentially modulated by DMTs. This study prompts future investigations for their role as markers of DMT response.

**Disclosure:** MS, AG, SM, PU, MK, MN, RO, MF, AG, BD, MS, DAS, NB, MT, AG, CB, DV, AG, SG, AGO, HBS, GA, TB, HFH, PV, JS, MJ and IK nothing to disclose FE received speaking honoraria from Merck and Genzyme. TG recipient of the Grant for Multiple Sclerosis Innovation (GMSI) funded by Merck, Darmstadt, Germany. FP has received research grants from Denka, Janssen, Merck KGaA, Novartis, Pfizer and UCB, and fees for serving on data monitoring committees in clinical trials with Lundbeck and Roche. FT is supported by Karolinska Institutet's doctoral education programme and has received a scholarship from the Blanceflor Foundation. FT has received compensation from Novartis for serving as a meeting moderator. MR received speaker honoraria from Bayer, Biogen, Roche, and Teva and research support from the Canadian MS Society and Fondazione Italiana Sclerosi Multipla. MF is Editor‐in‐Chief of the Journal of Neurology and received compensation for consulting services and/or speaking activities from Alexion, Merck‐Serono, Novartis, Roche and Teva Pharmaceutical Industries and receives research support from Biogen Idec, Merck‐Serono, the Italian Ministry of Health, Fondazione Italiana Sclerosi Multipla, 770 and ARiSLA (Fondazione Italiana di Ricerca per la SLA). BH has served on advisory boards for Novartis, Polpharma, and Hoffmann‐La Roche, as well as on DMSC boards for AllergyCare, Polpharma, Sandoz, Biocom, and TG Therapeutics. He has received honoraria for consulting services provided to clients of the Gerson Lehrman Group and AlphaSights, expert advice for Wuesthoff&Wuesthoff and for educational activities organized by neuro.today and patients.today. His organization has received funding for research projects by Polpharma and Hoffmann‐La Roche. All conflicts are not relevant to the topic of the study. He is associated with DIFUTURE (Data Integration for Future Medicine). He received funding for the study by the European Union's Horizon 2020 Research and Innovation Program and the Deutsche Forschungsgemeinschaft (DFG, German Research Foundation) under Germany's Excellence Strategy within the framework of the Munich Cluster for Systems Neurology. Chiristiane Gasperi received funding from the BMBF, DFG (German Research Foundation), the Hertie Foundation, and the Hans and Klementia Langmatz Stiftung. CG received funding from the BMBF and the DFG (German Research Foundation). SL received compensation for consulting services and speaker honoraria from Biogen Idec, Novartis, Johnson and Johnson, Sanofi, Merck, and Bristol‐Myers Squibb, and holds grants from the Instituto de Salud Carlos III and AGAUR. MM has received research support from the German Research Foundation, the Bavarian State Ministry for Science and Art, the German Federal Ministry of Education and Research, and the National Institutes of Health; he has received honoraria for lecturing from Merck Serono. FMB received travel support from Roche, Novartis and Biogen.

## OPR‐121

### Treatment specific infection risk in multiple sclerosis patients: A Danish nationwide registry study

#### R. Holm ^1^; F. Elberling^1^; S. Poulsen^2^; F. Sellebjerg^3^; M. Magyari^1^

##### ^*1*^ *Danish Multiple Sclerosis Registry, Department of Neurology, Copenhagen University Hospital Rigshospitalet, Glostrup, Denmark;* ^*2*^ *Department of Clinical Medicine, Faculty of Health and Medical Sciences, University of Copenhagen, Copenhagen, Denmark;* ^*3*^ *Danish Multiple Sclerosis Center, Department of Neurology, Copenhagen University Hospital Rigshospitalet, Glostrup, Denmark*

**Background and Aims:** Disease‐modifying therapies (DMTs) for multiple sclerosis (MS) vary in immunomodulatory and immunosuppressive effects. We compared infection risks across DMTs using nationwide registry data.

**Methods:** Patients starting DMTs (2017–2024) were identified in the Danish Multiple Sclerosis Registry. ICD‐10 codes captured hospital‐treated infections, and antimicrobial (AM) prescriptions by ATC codes served as infection proxies. Risk windows covered treatment duration plus a treatment‐specific period—infections within 6 months before treatment were excluded. Cox models estimated the risks of infection and AM use, accounting for repeated measures and adjusting for demographic and clinical covariates.

**Results:** The cohort included 11,140 patients and 24,403 treatment episodes. All DMTs, especially anti‐CD20 agents (HR: 3.0, 95% CI: 2.7–3.5), were associated with higher infection risks than teriflunomide, except alemtuzumab. Most DMTs increased antiviral use, with alemtuzumab conferring the greatest risk (HR: 9.8, 95% CI: 7.1–13.3). Alemtuzumab and ocrelizumab raised antimycotic use, and alemtuzumab had the highest total AM use.

**Conclusion:** This nationwide registry study found that anti‐CD20 DMTs conferred the highest infection risk, while alemtuzumab was associated with the greatest AM use. These findings underscore the heterogeneity of infection profiles and emphasize the need for tailored clinical management strategies across DMTs.

**Disclosure:** Rolf Pringler Holm has received speaker honoraria from Sanofi and Novartis, has served on an advisory board for Novartis, and has received a travel grant from The Danish Multiple Sclerosis Society. Frederik Elberling has received speaker honoraria from Sanofi and Roche. Susanne Dam Poulsen has received grants from the Novo Nordisk Foundation, the Independent Research Fund Denmark, and Svend Andersen Fonden, and reports advisory board activity for Gilead, Takeda, and GSK. Finn Sellebjerg has served on scientific advisory boards, served as a consultant, received support for congress participation, or received speaker honoraria from Biogen, Bristol Myers Squibb, H. Lundbeck A/S, Merck, Novartis, Roche, and Sanofi. His laboratory has received research support from Biogen, Merck, Novartis, Roche, and Sanofi. Additionally, FS serves as the section editor of the journal Multiple Sclerosis and Related Disorders, is the chairman of the research board of the Danish Multiple Sclerosis Society, and is a member of the scientific steering committee of the International Progressive MS Alliance. Melinda Magyari has served on scientific advisory boards, served as a consultant, received support for congress participation, or received speaker honoraria from Roche, Sanofi, Biogen, Merck, Novartis, Bristol Myers Squibb, Medscape, Alexion, and Moderna. Her research group has contracts with Biogen, Merck, Novartis, Roche, Sanofi, and Bristol Myers Squibb.

## OPR‐122

### The T cell repertoire of persons with multiple sclerosis is characterized by CD8+ T cells specifically targeting astrocytes

#### S. Jones ^1^; S. Perriot^1^; A. Meringa^1^; A. Mathias^1^; R. Genolet^2^; H. Lindsay^3^; M. Canales^1^; L. Queiroz^2^; C. Sauvage^2^; L. Oberholster^1^; R. Bernard‐Valnet^4^; M. Theaudin^4^; C. Pot^1^; R. Gottardo^3^; A. Harari^2^; R. Du Pasquier^1^

##### ^*1*^ *Laboratories of Neuroimmunology, Neuroscience Research Centre, Department of Clinical Neurosciences, Lausanne University Hospital and University of Lausanne, Lausanne, Switzerland;* ^*2*^ *Ludwig Institute for Cancer Research, Lausanne Branch, Department of Oncology, Lausanne University Hospital and University of Lausanne, Agora Cancer Research Center, Lausanne, Switzerland;* ^*3*^ *Biomedical Data Science Center, University of Lausanne and Lausanne University Hospital, Swiss Institute of Bioinformatics, Lausanne;* ^*4*^ *Service of Neurology, Department of clinical neurosciences, University Hospital of Lausanne and Lausanne University Hospital, Lausanne, Switzerland*

**Background and Aims:** Clonally expanded CD8+ T cells are prevalent in CNS lesions of persons with MS (pwMS), suggesting antigen‐driven proliferation. Beyond myelin, studies suggest that astrocytes and neurons may also be targeted. Since astrocytes and neurons upregulate HLA class I in active lesions, we sought to assess if pwMS harbor CD8+ T cells targeting these cells.

**Methods:** We generated human‐induced pluripotent stem cell (hiPSC) derived astrocytes and neurons from 8 pwMS and 6 age‐matched healthy donors (HD). We then cultured peripheral blood mononuclear cells (PBMC) with autologous hiPSC‐derived astrocytes or neurons for 14 days. To identify proliferating clonotypes, CD8+ T cell TCR repertoires were compared before and after coculture. The TCRs of these clonotypes were then transfected into NFAT‐luciferase Jurkat cells to validate for CNS autoreactivity. Public TCR databases from the blood/CSF of pwMS were screened for identical/similar TCR‐β CDR3 sequences. Finally, scRNAseq was performed on expanded CD8+ T cells.

**Results:** We demonstrate that CD8+ T cell repertoires of pwMS are restricted after coculture with astrocytes only. Astrocyte‐reactive clonotypes are exclusively found in pwMS (5/7 pwMS vs. 0/5 HD) with most clonotypes non‐reactive against PBMC, indicating CNS specificity. Identified astrocyte‐specific CD8+ T cell clonotypes share similar CDR3 sequences to blood/CSF clonotypes from pwMS from public databases. scRNAseq reveals that these clonotypes display a highly proliferative activated cytotoxic phenotype.

**Conclusion:** Our autologous CNS antigen presentation system demonstrates that astrocyte‐specific CD8+ T cells are a frequent finding in pwMS whilst mostly absent in HD, reinforcing a potential pathogenic role for astrocyte‐specific CD8+ T cells in MS.

**Disclosure:** S.J. is supported by the Swiss National Science Foundation (323630_214544). C.P. is supported by the Panacée Foundation, the Swiss MS Society and the Swiss National Science Foundation. R.D.P. is supported by a generous donator advised by Carigest SA and a part of this study was supported by the Swiss National Science Foundation (320030‐179531 and 320030‐231948). S.P., A.Me, A.Ma., R.Ge., H.L., M.C., L.Q., C.S., L.O., R.B.V., M.T., R.Go., A.H. have nothing to disclose.

## Cerebrovascular Diseases 2

## OPR‐123

### Competing stroke etiologies and outcomes after breakthrough ischemic stroke on oral anticoagulants in patients with atrial fibrillation

#### C. Di Cristofaro ^1^; M. Foschi^1^; F. De Santis^1^; F. Gabriele^1^; R. Ornello^1^; L. D'Anna^2^; A. Zini^3^; A. Cascio Rizzo^4^; L. Pantoni^5^; M. Bagnato^6^; B. Casolla^7^; B. Fuentes^8^; D. Aguiar de Sousa^9^; P. Caliandro^10^; A. Abdelalim^11^; L. Zhang^12^; A. El Bassiouny^13^; F. Ferrari^14^; M. Guarino^15^; G. Rinaldi^16^; G. Frisullo^17^; M. Mannino^18^; A. Fonseca^19^; H. Budincevic^20^; G. Viticchi^21^; L. Barba^22^; P. Lochner^23^; S. Buddha^24^; M. Piscaglia^25^; M. Zedde^26^; A. Nasreldein^27^; L. Vinciguerra^28^; A. Elsaid Elsayed^29^; M. Al Banna^30^; G. Merlino^31^; P. Candelaresi^32^; S. Sacco^1^

##### ^*1*^ *Department of Biotechnological and Applied Clinical Sciences, University of L'Aquila, L'Aquila, Italy;* ^*2*^ *Department of Stroke and Neuroscience, Charing Cross Hospital, Imperial College London NHS Healthcare Trust, London, UK;* ^*3*^ *IRCCS Istituto delle Scienze Neurologiche di Bologna, Department of Neurology and Stroke Center, Maggiore Hospital, Bologna, Italy;* ^*4*^ *Department of Neurology and Stroke Unit, ASST Grande Ospedale Metropolitano Niguarda, Milan, Italy;* ^*5*^ *Neuroscience Research Center, Department of Biomedical and Clinical Sciences, University of Milan, Italy;* ^*6*^ *UOC Stroke Unit e Neurologia, Ospedale Fabrizio Spaziani, Frosinone, Italy;* ^*7*^ *Stroke Unit, CHU Pasteur 2, Université Cote d'Azur, UMR2CA URRIS, Nice, France;* ^*8*^ *Stroke Center and Department of Neurology. Hospital La Paz Institute for Health Research (La Paz University Hospital‐Universidad Autónoma de Madrid). Madrid, Spain;* ^*9*^ *Stroke Center, Department of Neurosciences, Centro Hospitalar Universitário Lisboa Central, ULS São José, Faculty of Medicine, University of Lisbon, Lisbon, Portugal;* ^*10*^ *Department of Neuroscience, Catholic University of the Sacred Hearth, Rome, Italy; UOC Neurology, Department of Neuroscience, Sensory Organs, and Thorax, Fondazione Policlinico Universitario A. Gemelli IRCCS, Rome, Italy;* ^*11*^ *Cairo University Stroke Center, Department of Neurology, Faculty of Medicine, Cairo University, Egypt;* ^*12*^ *Department of Neurology, St George's University Hospital, London, UK;* ^*13*^ *Neurology Department, Faculty of Medicine, Ain Shams University, Cairo, Egypt;* ^*14*^ *Department of Brain and Behavioral Sciences, University of Pavia, Pavia, Italy;* ^*15*^ *IRCCS Istituto delle Scienze Neurologiche di Bologna, Italy;* ^*16*^ *S.C. Neurologia, Ospedale “Di Venere”, Bari, Italy;* ^*17*^ *Emergency Neurology – Department of Neuroscience, Sensory Organs, and Thorax, Policlinico Universitario Agostino Gemelli, IRCCS Rome, Italy;* ^*18*^ *UOC Neurologia e Stroke Unit, AOOR Villa Sofia‐Cervello, Palermo, Italy;* ^*19*^ *Neurology Department, Hospital de Santa Maria, Faculty of Medicine, University of Lisbon, Portugal;* ^*20*^ *Department of Neurology, Sveti Duh University Hospital, Zagreb, Croatia;* ^*21*^ *Neurological Clinic, Marche Polytechnic University, Ancona, Italy;* ^*22*^ *Department of Neurology, Martin‐Luther‐University Halle‐Wittenberg, Germany;* ^*23*^ *Department of Neurology, Saarland University Medical Center, Homburg, Germany;* ^*24*^ *Department of Stroke Medicine, Southmead Hospital, North Bristol NHS Trust, Bristol, UK;* ^*25*^ *Department of Neurosciences, Stroke Unit, Neurology Unit, S.Maria delle Croci Hospital, AUSL Romagna, Ravenna, Italy;* ^*26*^ *Neurology Unit, Stroke Unit, Azienda Unità Sanitaria Locale‐IRCCS di Reggio Emilia, Italy;* ^*27*^ *Department of Neurology, Assiut University, Assiut, Egypt;* ^*28*^ *Department of Neurology and Stroke Unit, ASST Crema Hospital, Italy;* ^*29*^ *Neurology and Neurointervention Department, Kobry El Kobba Medical Complex, Cairo, Egypt;* ^*30*^ *Department of Neurology, National Neuroscience Institute, King Fahad Medical City, Riyadh, Saudi Arabia;* ^*31*^ *SOSD Stroke Unit, Udine University Hospital, Italy,* ^*32*^ *UOC Neurologia e Stroke Unit, AORN Cardarelli, Napoli, Italy*

**Background and Aims:** Patients with atrial fibrillation (AF) experiencing ischemic stroke despite oral anticoagulation (OAC) face high risk of recurrence and bleeding. We compared 90‐day outcomes between patients with and without competing stroke etiologies after breakthrough ischemic stroke on OAC for AF.

**Methods:** ASPERA‐R retrospectively enrolled adults (>18 years) with AF experiencing breakthrough ischemic stroke on continuous OAC (≤48 h) across 35 centers (Feb 2020–Feb 2025). Eligibility required imaging‐confirmed stroke, vessel imaging, and 90‐day follow‐up; inadequate dosing or poor adherence were excluded. Competing etiologies coexisting with cardioembolism were assessed. Primary outcome: 90‐day recurrent stroke; secondary: mRS shift, myocardial infarction, vascular and all‐cause death, and bleeding. IPW balanced baseline covariates; Cox/regression models compared outcomes.

**Results:** Among 1649 patients (mean age 78, 47.8% male), 24.3% had competing etiologies, most commonly large‐artery atherosclerosis (59.9%). Weighted analyses showed higher 90‐day recurrent ischemic stroke in patients with competing etiologies (6.0% vs. 2.7%; aHR 2.62), particularly large‐artery atherosclerosis and other determined causes. They also had worse disability (aOR 1.30), higher all‐cause mortality (aHR 1.58), and increased moderate‐to‐severe bleeding (aHR 1.82), while 24‐h hemorrhagic transformation was less frequent (aRD −4.3%). Other outcomes did not differ

**Figure:**
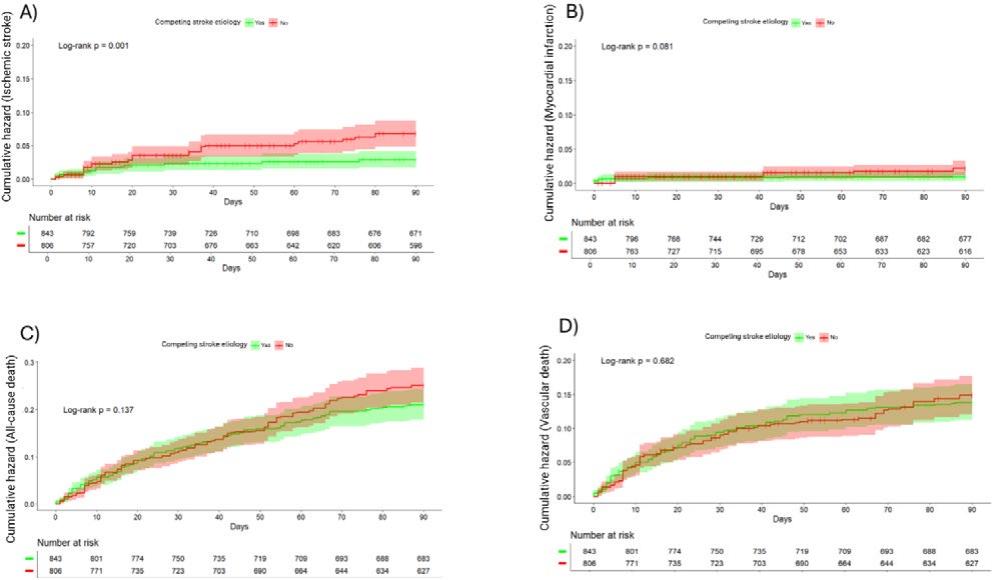
FIGURE 1

**Figure:**
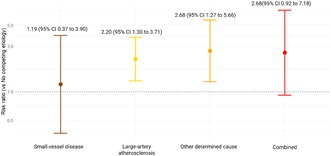
FIGURE 2

**Figure:**
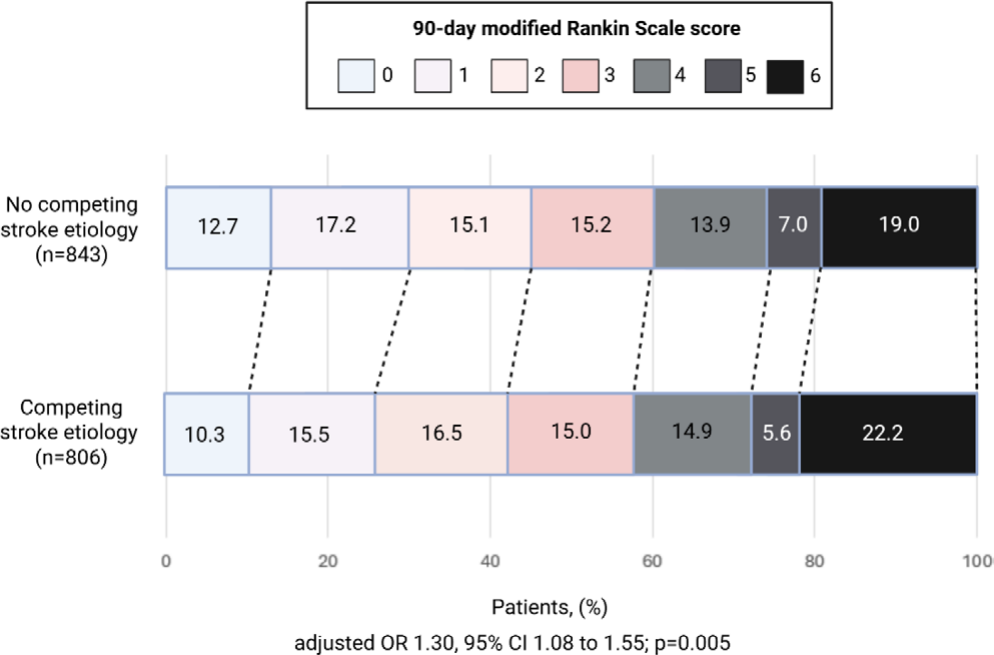
FIGURE 3

**Conclusion:** One in four patients with AF and breakthrough ischemic stroke on OAC had competing etiologies, most often large‐artery atherosclerosis. These patients showed higher risk of recurrent stroke, functional dependence, mortality, and bleeding, highlighting the need for development of individualized secondary prevention. Main study limits include observational retrospective design and incomplete data on on‐treatment anticoagulation quality.

**Disclosure:** Nothing to disclose.

## OPR‐124

### Continuation versus switching direct oral anticoagulant after breakthrough stroke: A non‐inferiority target trial analysis from the ASPERA‐R Study

#### F. De Santis ^1^; L. D'Anna^2^; F. Gabriele^1^; R. Ornello^1^; A. Zini^3^; A. Cascio Rizzo^4^; L. Pantoni^5^; M. Bagnato^6^; B. Casolla^7^; B. Fuentes^8^; P. Candelaresi^9^; D. Aguiar de Sousa^10^; P. Caliandro^11^; A. Abdelalim^12^; F. Ferrari^13^; M. Guarino^14^; G. Rinaldi^15^; G. Frisullo^16^; M. Mannino^17^; A. Catarina Fonseca^18^; H. Budincevic^19^; G. Viticchi^20^; L. Barba^21^; P. Lochner^22^; S. Buddha^23^; M. Piscaglia^24^; M. Zedde^25^; A. Nasreldein^26^; L. Vinciguerra^27^; A. Elsaid Elsayed^28^; M. Al Banna^29^; G. Merlino^30^; S. Sacco^1^

##### ^*1*^ *Department of Biotechnological and Applied Clinical Sciences, University of L'Aquila, L'Aquila, Italy;* ^*2*^ *Department of Stroke and Neuroscience, Charing Cross Hospital, Imperial College London NHS Healthcare Trust, London, UK;* ^*3*^ *IRCCS Istituto delle Scienze Neurologiche di Bologna, Department of Neurology and Stroke Center, Maggiore Hospital, Bologna, Italy;* ^*4*^ *Department of Neurology and Stroke Unit, ASST Grande Ospedale Metropolitano Niguarda, Milan, Italy;* ^*5*^ *Department of Neurology and Stroke Unit, ASST Grande Ospedale Metropolitano Niguarda, Milan, Italy;* ^*6*^ *UOC Stroke Unit e Neurologia, Ospedale Fabrizio Spaziani, Frosinone, Italy;* ^*7*^ *Stroke Unit, CHU Pasteur 2, Université Cote d'Azur, UMR2CA URRIS, Nice, France;* ^*8*^ *Stroke Center and Department of Neurology, Hospital La Paz Institute for Health Research (La Paz University Hospital‐Universidad Autónoma de Madrid), Madrid, Spain;* ^*9*^ *UOC Neurologia e Stroke Unit, AORN Cardarelli, Napoli, Italy;* ^*10*^ *Stroke Center, Department of Neurosciences, Centro Hospitalar Universitário Lisboa Central – ULS São José, Faculty of Medicine, University of Lisbon, Lisbon, Portugal;* ^*11*^ *Department of Neuroscience, Catholic University of the Sacred Hearth, Rome, Italy; UOC Neurology, Department of Neuroscience, Sensory Organs, and Thorax, Fondazione Policlinico Universitario A. Gemelli IRCCS, Rome, Italy;* ^*12*^ *Cairo University Stroke Center, Department of Neurology, Faculty of Medicine, Cairo University, Egypt;* ^*13*^ *Department of Brain and Behavioral Sciences, University of Pavia, Pavia, Italy;* ^*14*^ *IRCCS Istituto delle Scienze Neurologiche di Bologna, Italy;* ^*15*^ *S.C. Neurologia, Ospedale “Di Venere”, Bari, Italy;* ^*16*^ *Emergency Neurology, Department of Neuroscience, Sensory Organs, and Thorax – Policlinico Universitario Agostino Gemelli, IRCCS Rome, Italy;* ^*17*^ *UOC Neurologia e Stroke Unit, AOOR Villa Sofia‐Cervello, Palermo, Italy;* ^*18*^ *Neurology Department, Hospital de Santa Maria, Faculty of Medicine, University of Lisbon, Portugal;* ^*19*^ *Department of Neurology, Sveti Duh University Hospital, Zagreb, Croatia;* ^*20*^ *Neurological Clinic, Marche Polytechnic University, Ancona, Italy;* ^*21*^ *Department of Neurology, Martin‐Luther‐University Halle‐Wittenberg, Germany;* ^*22*^ *Department of Neurology, Saarland University Medical Center, Homburg, Germany;* ^*23*^ *Department of Stroke Medicine, Southmead Hospital, North Bristol NHS Trust, Bristol, UK;* ^*24*^ *Department of Neurosciences, Stroke Unit, Neurology Unit, S.Maria delle Croci Hospital, AUSL Romagna, Ravenna, Italy;* ^*25*^ *Neurology Unit, Stroke Unit, Azienda Unità Sanitaria Locale‐IRCCS di Reggio Emilia, Italy;* ^*26*^ *Department of Neurology, Assiut University, Assiut, Egypt;* ^*27*^ *Department of Neurology and Stroke Unit, ASST Crema Hospital, Italy;* ^*28*^ *Neurology and Neurointervention Department, Kobry El Kobba Medical Complex, Cairo, Egypt;* ^*29*^ *Department of Neurology, National Neuroscience Institute, King Fahad Medical City, Riyadh, Saudi Arabia;* ^*30*^ *SOSD Stroke Unit, Udine University Hospital, Italy*

**Background and Aims:** Management of ischemic stroke occurring despite direct oral anticoagulant (DOAC) therapy for atrial fibrillation remains inconsistent due to limited evidence regarding the benefits of switching treatments. This study aims to evaluate the effectiveness and safety of continuing the current DOAC versus switching to an alternative oral anticoagulant, utilizing a predefined non‐inferiority framework.

**Methods:** This multicenter, retrospective study involved 35 stroke centers across Europe and North Africa. We emulated a target trial employing a non‐inferiority comparison of continuation versus switching to an alternative oral anticoagulant (different DOAC or VKA) after a breakthrough stroke. The primary outcome was 90‐day net clinical benefit (recurrent ischemic stroke and moderate‐to‐severe bleeding). Secondary endpoints included all‐cause mortality, vascular death, and specific recurrent ischemic or hemorrhagic events.

**Results:** We included 1006 consecutive adults (median age 80.4 years; 50% women). After IPTW adjustment, the 90‐day net clinical benefit was similar between strategies (Figure 1): 5.1% in continuation vs. 4.9% in switching (Figure 2), with a risk difference of −0.3% (90% CI, −2.7% to 2.1%), satisfying non‐inferiority. Recurrent ischemic events and bleeding outcomes were likewise comparable, with absolute differences within prespecified margins. Non‐inferiority was not demonstrated for all‐cause or vascular mortality.

**Figure:**
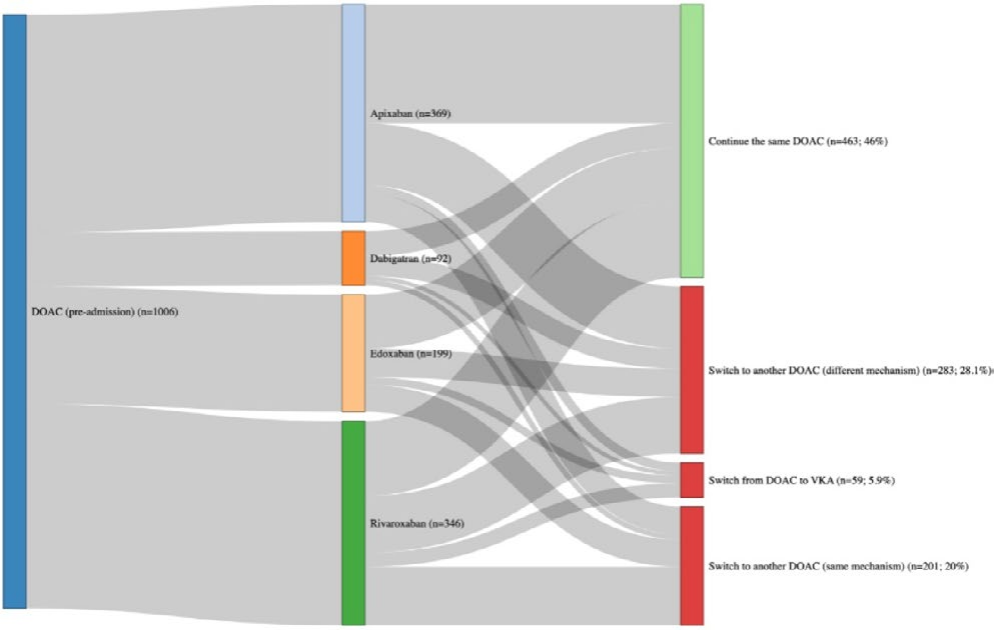
**FIGURE 1** Sankey flow of pre‐stroke DOAC agent and post‐stroke anticoagulation strategy.

**Figure:**
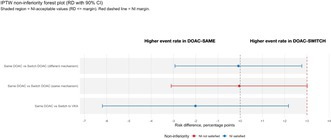
**FIGURE 2** Strategy‐specific comparison for net clinical benefit (IPTW, Risk Difference scale).

**Conclusion:** Continuing the same DOAC after a breakthrough ischemic stroke resulted in clinical outcomes comparable to switching anticoagulant therapy, with absolute differences within clinically acceptable margins for most outcomes. Our findings do not support routine switching to improve secondary prevention after a breakthrough event. Randomized controlled trials are needed to identify strategies to improve secondary prevention after a breakthrough ischemic stroke.

**Disclosure:** Nothing to disclose.

## OPR‐125

### Clinical outcome of MRI‐negative acute ischemic stroke treated with and without intravenous thrombolysis

#### F. Fenter ^1^; V. Dunet^2^; P. Michel^1^; D. Strambo^1^

##### ^*1*^ *Stroke Center, Neurology Service, Department of Clinical Neurosciences, Lausanne, Switzerland;* ^*2*^ *Neuroradiology Unit, Service of Diagnostic and Interventional Radiology, Department of Medical Radiology, both at the Lausanne University Hospital and University of Lausanne, Lausanne, Switzerland*

**Background and Aims:** Intravenous thrombolysis (IVT) for acute ischemic stroke (AIS) is guided by clinical assessment and exclusion of radiological contraindications. While MRI protocols reduce mistreatment of stroke mimics, infra‐radiologic strokes (IRS) on admission MRI remain diagnostically challenging. We aimed to characterize MRI‐based IRS and evaluate their clinical and functional outcomes.

**Methods:** From our local prospectively‐constructed stroke registry we identified all AIS undergoing acute MRI, without intracranial occlusion. IRS were defined as clinically diagnosed AIS with negative DWI and perfusion imaging. IRS were compared with radiologically confirmed (RC)‐AIS. Within IRS‐patients, IVT‐treated patients were compared with those receiving conservative treatment. Outcomes included 3‐month modified‐Rankin‐Scale (mRS) shift, 24‐h‐NIHSS‐shift, lesion evidence on follow‐up imaging (LFI). Analyses were adjusted using propensity score weighting.

**Results:** Among 1315 consecutive eligible AIS between 2018 and 2024, 58 (4.4%) had an IRS. Compared with RC‐AIS in univariate analysis, IRS patients had similar baseline NIHSS, shorter onset‐to‐door times (96 vs. 246 min, *p* < 0.001), less paresis (44.8% vs. 63.4%, *p* = 0.006), more infra‐tentorial localization (48.3% vs. 22.5%, *p* < 0.001), received more IVT (63.8% vs. 32.1%, *p* < 0.001), and had less LFI (17.8% vs. 73.3%, *p* < 0.001). After adjustment, IRS patients had greater 24‐h‐NIHSS improvement (beta = 1.68, 95% CI = 0.29–3.07), less LFI (pswOR = 0.05, 95% CI = 0.02–0.12), and similar 3‐month mRS. Within the IRS group, IVT was associated with greater 24‐h‐NIHSS improvement (beta = 2.97, 95% CI = 1.17–4.78) without significant 3‐month mRS changes.

**Figure:**
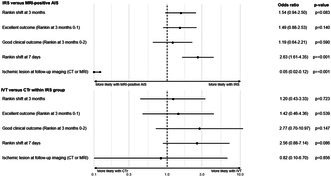
**FIGURE 1** Forest plot of binary/categorial outcomes.

**Conclusion:** IRS represent an uncommon AIS radiotype and overall similar medium‐term outcomes as MRI‐positive AIS. In IRS, IVT was associated with improved short but not medium‐term outcomes.

**Disclosure:** Nothing to disclose.

## OPR‐126

### Exploring the contribution of cerebrovascular risk factors to glymphatic system impairment in multiple sclerosis

#### M. Rubin ^1^; P. Preziosa^1^; E. Pagani^2^; A. Meani^2^; M. Margoni^3^; L. Storelli^2^; M. Albergoni^2^; M. Filippi^4^; M. Rocca^1^

##### ^*1*^ *Neuroimaging Research Unit, Division of Neuroscience, and Neurology Unit, IRCCS San Raffaele Scientific Institute, Milan, Italy; and Vita‐Salute San Raffaele University, Milan, Italy;* ^*2*^ *Neuroimaging Research Unit, Division of Neuroscience, IRCCS San Raffaele Scientific Institute, Milan, Italy;* ^*3*^ *Neuroimaging Research Unit, Division of Neuroscience, Neurology Unit, and Neurorehabilitation Unit, IRCCS San Raffaele Scientific Institute, Milan, Italy;* ^*4*^ *Neuroimaging Research Unit, Division of Neuroscience, Neurology Unit, Neurorehabilitation Unit, and Neurophysiology Service, IRCCS San Raffaele Scientific Institute, Milan, Italy; and Vita‐Salute San Raffaele University, Milan, Italy*

**Background and Aims:** Cerebrovascular risk factors (cVRFs) associate with greater brain damage and disability in multiple sclerosis (MS) patients. The glymphatic system, contributing to brain waste clearance, is impaired in both MS and cerebrovascular diseases. We investigated whether choroid plexus volume (CPV) and the diffusion tensor imaging analysis along the perivascular spaces (DTI‐ALPS) are influenced by cVRFs in MS.

**Methods:** Two‐hundred nine MS patients and 123 age‐ and sex‐matched healthy controls (HC) underwent cVRF, neurological evaluation, and brain 3T MRI acquisition. CP volume was quantified using a deep learning‐based approach on 3D T1‐weighted MRI scans; DTI‐ALPS was used to assess glymphatic system function.

**Results:** At least one cVRF was present in 64% of MS patients and 41% of HC. MS patients with and without cVRFs showed no differences for disease duration, disability, clinical phenotype and treatment (*p* ≥ 0.738). Compared to HC, MS patients had significantly higher T2‐hyperintense white matter (WM) lesion volume, lower global and regional brain volumes, and larger CPV (FDR‐p ≤ 0.003), regardless of cVRFs (FDR‐p ≥ 0.553). Both MS groups with and without cVRFs exhibited significantly lower DTI‐ALPS index compared to their respective HC counterparts (FDR‐p ≤ 0.002), with the coexistence of MS and cVRFs resulting in a significantly greater DTI‐ALPS index reduction (FDR‐p = 0.013). Lower DTI‐ALPS and higher CPV correlated with higher T2‐hyperintense WM lesion volume and lower brain volumes (FDR‐p ≤ 0.007), independently from cVRF presence (FDRp ≥ 0.122).

**Conclusion:** cVRFs may worsen glymphatic system dysfunction in MS, possibly contributing to aggravate neuroinflammation and neurodegeneration and supporting the importance of proactive cVRF management.

**Disclosure:** M. Rubin has nothing to disclose. P. Preziosa received speaker honoraria from Roche, Biogen, Novartis, Merck, Bristol Myers Squibb, Genzyme, Horizon and Sanofi. E. Pagani and A. Meani have nothing to disclose. M. Margoni reports grants and personal fees from Sanofi Genzyme, Merck Serono, Roche, Biogen, Amgen and Novartis. L. Storelli and M. Albergoni have nothing to disclose. M.A. Rocca received consulting fees from Biogen, Bristol Myers Squibb, Roche; and speaker honoraria from Alexion, Biogen, Bristol Myers Squibb, Celgene, Horizon Therapeutics Italy, Merck Serono SpA, Mitsubishi‐Tanabe Pharma, Neuraxpharm, Novartis, Roche, Sandoz, and Sanofi. She receives research support from the MS Society of Canada, the Italian Ministry of Health, the Italian Ministry of University and Research, and Fondazione Italiana Sclerosi Multipla. She is Associate Editor for Multiple Sclerosis and Related Disorders; and Associate Co‐Editor for Europe and Africa for Multiple Sclerosis Journal. M. Filippi is Editor‐in‐Chief of the Journal of Neurology, Associate Editor of Human Brain Mapping, Neurological Sciences, and Radiology; received compensation for consulting services from Almirall, Biogen, Bristol‐Myers Squibb, Eli Lilly, Merck, Novartis, Roche, Sanofi; speaking activities from Amgen, Bayer, Biogen, Bristol‐Myers Squibb, Celgene, Chiesi Italia SpA, Eisai, Eli Lilly, Fujirebio, Genzyme, Janssen, Merck, Neopharmed Gentili, Neuraxpharm, Novartis, Novo Nordisk, Roche, Sanofi, Takeda; participation in Advisory Boards for Alexion, Biogen, Bristol‐Myers Squibb, Eli Lilly, GE Healthcare Ltd, Merck, Neuraxpharm, Novartis, Roche, Sandoz, Sanofi, Takeda; scientific direction of educational events for Biogen, Merck, Roche, Celgene, Bristol‐Myers Squibb, Lilly, Novartis, Sanofi‐Genzyme; he receives research support from Biogen Idec, Merck‐Serono, Novartis, Roche, the Italian Ministry of Health, the Italian Ministry of University and Research, and Fondazione Italiana Sclerosi Multipla.

## OPR‐127

### Endovascular therapy improves clinical outcomes in acute ischemic stroke due to isolated M2 occlusion: Real‐world experience from the RES‐Q registry

#### R. Herzig ^1^; S. Szabó^2^; G. Neto^3^; R. Mikulík^3^

##### ^*1*^ *Department of Neurology, Charles University Faculty of Medicine and University Hospital Hradec Králové, Hradec Králové, Czech Republic;* ^*2*^ *Department of Neurology, Charles University Faculty of Medicine in Hradec Králové, Hradec Králové, Czech Republic;* ^*3*^ *International Clinical Research Center, St. Anne's University Hospital in Brno, Brno, Czech Republic*

**Background and Aims:** Indication for endovascular therapy (EVT) in acute ischemic stroke (AIS) caused by middle cerebral artery M2 segment (MCA/M2) occlusion remains controversial. The aim was to compare clinical outcomes in these patients treated with intravenous thrombolysis (IVT), EVT, and IVT + EVT.

**Methods:** In an observational multicenter cohort study, we analyzed data from AIS with causative MCA/M2 occlusion treated with IVT, EVT, and IVT+EVT, prospectively collected in the RES‐Q registry in the Czech Republic (2022–2024). Clinical outcomes were assessed using utility‐weighted 3‐month modified Rankin Scale (mRS) and 3‐month mortality. Baseline differences were adjusted using multivariable ordinal regression for utility‐weighted 3‐month mRS and multivariable logistic regression for 3‐month mortality, including age, sex, National Institutes of Health Stroke Scale, pre‐stroke mRS, and onset‐to‐treatment time. Missing 3‐month outcomes were handled using multiple imputation.

**Results:** The IVT group consisted of 520 (248 males; median age 74 [65–82] years), the EVT group of 223 (117 males; median age 73 [61–81] years), and the IVT+EVT group of 321 patients (163 males; median age 73 [64–79] years). Adjusted utility‐weighted 3‐month mRS was significantly better in the IVT+EVT versus IVT group (OR 1.26, 95% CI 1.05–1.51), and in the IVT+EVT versus EVT group (OR 1.31, 95% CI 1.07–1.59); there was no difference between the EVT and IVT groups. There were no differences in 3‐month mortality between the groups.

**Conclusion:** The addition of EVT to IVT was associated with better 3‐month clinical outcomes compared with IVT alone in patients with AIS due to MCA/M2 occlusion.

**Disclosure:** Roman Herzig was supported by STROCZECH within the CZECRIN Large Research Infrastructure (No. LM2023049) funded by the state budget of the Czech Republic, Ministry of Health of the Czech Republic (Grant No. DRO‐UHHK 00179906), and Charles University, Czech Republic (Cooperatio Program, research area NEUR). Szabolcs Szabó was supported by Charles University, Czech Republic (Cooperatio Program, research area NEUR). Geraldo Neto and Robert Mikulík were supported by STROCZECH within the CZECRIN Large Research Infrastructure (No. LM2023049) funded by the state budget of the Czech Republic.

## Movement Disorders 3

## OPR‐128

### The EJS ACT‐PD multi‐arm, multi‐stage platform trial: Assessing potential disease‐modifying therapies for Parkinson's disease

#### G. Mills^1^; M. Zeissler^2^; C. Gonzalez‐Robles^1^; O. Bandmann^3^; R. Barker^4^; L. Brennan^5^; J. Carpenter^5^; S. Collins^2^; J. Duffen^6^; S. Gandhi^1^; C. Girges^1^; R. Gurney^1^; K. McFarthing^7^; C. Murphy^5^; C. Pugh^5^; A. Schapira^1^; A. Schrag^1^; T. Foltynie^1^; C. Carroll ^2^

##### ^*1*^ *University College London;* ^*2*^ *Newcastle University;* ^*3*^ *University of Sheffield;* ^*4*^ *University of Cambridge;* ^*5*^ *Medical Research Council Clinical Trials Unit;* ^*6*^ *Cure Parkinson's;* ^*7*^ *Expert by Experience*

**Background and Aims:** The Edmond J. Safra Accelerating Clinical Trials in Parkinson's disease (EJS ACT‐PD) trial uses a multi‐arm, multi‐stage (MAMS) design to accelerate the evaluation of potential disease‐modifying therapies (DMTs) for Parkinson's disease (PD). MAMS designs enable simultaneous testing of multiple therapies; early identification of ineffective therapies for discontinuation; and efficient incorporation of new candidates. The primary objective is to detect a 30% reduction in the rate of increase in Movement Disorder Society‐Unified PD Rating Scale (MDS‐UPDRS) parts I and II combined within a representative UK population of people with PD.

**Methods:** Initially 1600 participants will be recruited across 40 UK sites using a self‐referral online registration of interest process. Participants will be randomised to telmisartan, terazosin, or placebo, with a third treatment arm (ursodeoxycholic acid) opening in 2026. A hybrid delivery model enables 6‐monthly visits to be conducted either in person or remotely. Trial medication will be delivered to participants homes to be taken for up to 3 years. Early‐stage analyses will use the MDS‐UPDRS parts I, II and remote III to inform discontinuation of ineffective treatments. Sub‐studies include passive digital monitoring of progression and optional collection of blood and cerebrospinal fluid for biomarker research.

**Results:** Recruitment opened in September 2025, with site activation continuing until March 2026. Over 1300 individuals have already registered interest.

**Conclusion:** EJS ACT‐PD is set to become the world's largest Parkinson's disease trial, providing an efficient platform to evaluate multiple DMTs while generating an extensive dataset to advance understanding of disease progression.

**Disclosure:** Nothing to disclose.

## OPR‐129

### PD vs. PSP‐P: superior diagnostic biomarker performance for combining Tau‐PET and MRI

#### T. van Eimeren^1^; J. Gnörich^2^; P. Alster^3^; G. Bischof^4^; N. Franzmeier^5^; N. Madetko‐Alster^3^; C. Palleis^6^; M. Brendel^7^; H. Theis ^1^; G. Höglinger^6^

##### ^*1*^ *Department of Nuclear Medicine and Department of Neurology, Faculty of Medicine and University Hospital Cologne, University of Cologne, Cologne, Germany;* ^*2*^ *Department of Nuclear Medicine, University Hospital, LMU Munich, Munich, Germany;* ^*3*^ *Department of Neurology, Medical University of Warsaw, Warsaw, Poland;* ^*4*^ *Department of Nuclear Medicine, Faculty of Medicine and University Hospital Cologne, University of Cologne, Cologne, Germany. Life Molecular Imaging, a Lantheus Company;* ^*5*^ *German Center of Neurodegenerative Diseases (DZNE), Munich, Germany. Institute of Stroke and Dementia Research (ISD), University Hospital, LMU Munich, Munich, Germany. The Sahlgrenska Academy, Institute of Neuroscience and Physiology;* ^*6*^ *German Center of Neurodegenerative Diseases (DZNE), Munich, Germany. Munich Cluster for Systems Neurology (SyNergy), Munich, Germany. Department of Neurology, University Hospital, LMU Munich, Munich, Germany;* ^*7*^ *Department of Nuclear Medicine, University Hospital, LMU Munich, Munich, Germany. German Center of Neurodegenerative Diseases (DZNE), Munich, Germany. Munich Cluster for Systems Neurology (SyNergy), Munich, Germany*

**Background and Aims:** Differentiating the parkinsonian variant of progressive supranuclear palsy (PSP‐P) from Parkinson's disease (PD) remains clinically challenging due to substantial phenotypic overlap. Reliable imaging biomarkers are therefore essential to improve early diagnostic accuracy. This study evaluated whether structural MRI (midbrain‐to‐pons ratio) or Tau‐PET ([18F]PI‐2620) provides superior discriminatory power, and whether a multimodal approach enhances diagnostic performance.

**Methods:** Fourteen patients with PD and fifteen with PSP‐P were diagnosed according to recent consensus criteria. All subjects underwent Tau‐PET imaging (20–40 min p.i.). Midbrain‐to‐pons (M/P) ratios were derived from high‐resolution T1‐weighted MRI. ROC analyses were performed for M/P ratio, globus pallidus (GP) SUVRs, and a combined logistic‐regression model including both biomarkers. AUCs were compared using DeLong tests. Binary logistic regression models assessed explained variance (Nagelkerke R^2^) and improvements in model fit via –2LL chi‐square comparisons.

**Figure:**
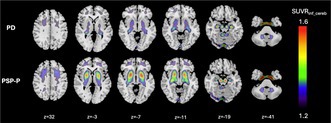
**FIGURE 1** Mean [18F]PI‐2620 SUVR images of the PD and PSP‐P group.

**Results:** AUCs were 0.79 for M/P ratio, 0.83 for GP SUVRs, and 0.91 for the combined model. DeLong testing showed no significant difference between M/P ratio and GP SUVRs (*p* = 0.73), but the combined model outperformed M/P ratio alone (*p* < 0.05). Logistic regression revealed explained variance of 38% for M/P ratio, 50% for GP SUVRs, and 72% for the combined model. Adding PET to MRI and adding MRI to PET improved model fit significantly (*p* < 0.005) in both directions.

**Figure:**
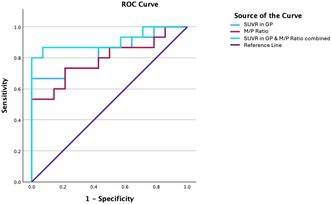
**FIGURE 2** ROC curves for M/P ratio, globus pallidus (GP) SUVRs, and a combined logistic‐regression model including both biomarkers.

**Conclusion:** Both MRI and Tau‐PET serve as informative biomarkers for differentiating PD from PSP‐P, with PET showing higher individual discriminatory power. However, combining structural MRI and Tau‐PET provides the strongest diagnostic accuracy, supporting multimodal biomarker strategy for early and reliable differentiation of PD and PSP.

**Disclosure:** #Contributed equally GNB is a full time employee at Life Molecular Imaging, a Lantheus Company. N.F. has received speaker or consulting honoraria from Life Molecular Imaging, GE Healthcare, MSD, EISAI and Biogen. C.P. is inventor in a patent “Oral Phenylbutyrate for Treatment of Human 4‐Repeat Tauopathies” (PCT/EP2024/053388) filed by LMU Munich. M.B. is a member of the Neuroimaging Committee of the EANM. M.B. has received speaker honoraria from Roche, GE Healthcare, Iba, Miltenyi, and Life Molecular Imaging; has advised Life Molecular Imaging, MIAC, Cenos, and GE healthcare; and is currently on the advisory or imaging review boards of AC Immune and ZRO Imaging. H.T. was supported by the Cologne Clinician Scientist Program (CCSP)/Faculty of Medicine/University of Cologne, funded by the DFG—Project ID 413543196.

## OPR‐130

### Digital biomarkers in normal pressure hydrocephalus: Instrumented timed up and go for assessing and predicting shunt response

#### I. D'Ascanio ^1^; D. Milletti^2^; E. Magelli^2^; A. D'Apolito^3^; L. Sambati^2^; L. Albini‐Riccioli^2^; D. Mascarella^2^; P. Cortelli^3^; G. Calandra‐Buonaura^2^; S. Cevoli^2^; L. Palmerini^1^; G. Palandri^2^; G. Giannini^2^

##### ^*1*^ *Department of Electrical, Electronic and Information Engineering, Alma Mater Studiorum – University of Bologna, Bologna, Italy;* ^*2*^ *IRCCS Istituto delle Scienze Neurologiche di Bologna, Bologna, Italy;* ^*3*^ *Department of Biomedical and Neuromotor Sciences (DIBINEM), University of Bologna, Bologna, Italy*

**Background and Aims:** Normal pressure hydrocephalus (NPH) is characterized by early manifestations of gait apraxia [1]. Wearable sensors have emerged as a promising approach to quantitatively capture motor impairments, with sensor‐derived motor parameters demonstrating moderate to strong correlations with clinical measures [3]. This study aims to evaluate sensor‐derived motor parameters by assessing both their responsiveness to shunt surgery and their ability to predict shunt response.

**Methods:** A total of 254 NPH individuals from the Bologna PRO‐Hydro cohort performed instrumented Timed Up and Go using a sensor worn on the lower back, at baseline (preTT), 3 days after (postTT) the cerebrospinal fluid tap test (CSF‐TT), and 6 months following shunt surgery (postShunt) [4]. Responsiveness to shunt surgery was assessed by examining changes between preTT and postShunt. Two predictive models were developed using preTT values alone or combined with postTT–preTT changes. The prognostic value of CSF‐TT for surgery outcomes was assessed via likelihood ratio testing.

**Results:** Most instrumental variables showed statistically significant improvements after surgery, including reduced duration (*p* < 0.001), more controlled sit‐to‐walk transitions (lower jerk, *p* < 0.001), and faster turns (higher velocity, *p* < 0.01). Most parameters showed a significant improvement in the predictive model when ∆(postTT–preTT) was included as a predictor. Motor strategies during sit‐to‐walk were found to be key determinants in post‐shunt prediction (adjusted *R* ^2^ > 0.65).

**Conclusion:** Sensor–derived parameters capture clinically meaningful motor changes provide significant predictive value for surgery outcomes. These findings support the use of instrumented motor tests as objective measures to complement traditional clinical assessments and guide candidate selection for shunt surgery.

**Disclosure:** Nothing to disclose.

## OPR‐131

### Tourette syndrome symptomatology associated with hyper‐glutamatergic activity in the anterior cingulate cortex

#### M. Boussac ^1^; A. Salabert^2^; E. Harroch^3^; C. Thalamas^4^; P. Payoux^5^; C. Brefel‐Courbon^3^

##### ^*1*^ *Toulouse NeuroImaging Center (ToNIC), Inserm, University of Toulouse III (UPS), France;* ^*2*^ *Radiopharmacy Department, Toulouse University Hospital, Toulouse, France;* ^*3*^ *Clinical Pharmacology and Neurosciences Department, Parkinson Expert Center, Toulouse University Hospital, NeuroToul COEN (Center of Excellence in Neurodegeneration), Toulouse, NS‐PARK/FCRIN Network, France;* ^*4*^ *Clinical Investigation Center, Clinical Pharmacology Department, Toulouse University Hospital, Toulouse, France;* ^*5*^ *Nuclear Medicine Department, Toulouse University Hospital, Place du Dr Baylac, Toulouse Cedex, France*

**Background and Aims:** In Tourette syndrome (TS), tics would result from a dysfunction in the cortico‐basal ganglia‐thalamo‐cortical loops (Atkinson‐Clement et al. 2020), related to an hyper‐dopaminergic activity. Nonetheless, treatment by dopamine antagonists is not sufficient for every patients, and topiramate, a glutamatergic antagonist, is sometimes more efficient to reduce tics (Cs et al. 2013). Therefore, our objective was to study the glutamatergic system in vivo in TS patients by evaluating NMDA receptors (NMDAR) activity using PET (Positron emission tomography) imaging.

**Methods:** TS patients (*n* = 12) and matched healthy volunteers (*n* = 12) were included in the GlutaTour study (NCT03681795). PET was done using the [18F]‐FNM (Fluoroethylnormemantine) radiotracer to study activated NMDAR. Tics severity was measured using the YGTSS (Yale Global Tic Severity Scale). Normalized PET activity was compared between TS patients and healthy volunteers (*t*‐tests), and correlations were done between YGTSS scores and normalized PET activity in patients.

**Results:** Activated NMDAR were significantly higher in TS patients compared to healthy volunteers, mainly in the right anterior cingulate cortex (rACC) (*p*.value = 0.03), and their activity in the rACC correlated significantly and positively with the YGTSS scores (*p*.value = 0.005 and coefficient = 0.75).

**Figure:**
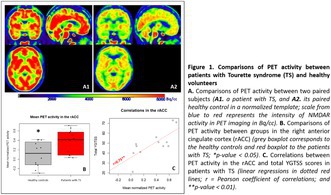
**FIGURE 1** Comparisons of PET activity between patients with Tourette syndrome (TS) and healthy volunteers.

**Conclusion:** This study is the first to have evaluated in vivo part of the glutamatergic system in TS patients, showing more activity within the NMDAR compared to controls. This supposed “hyper‐glutamatergic activity” in the ACC of TS patients could highlight a disinhibition of direct striatal pathway in TS which would result in tics emergence.

**Disclosure:** I declare no conflict of interest for this study. This study was funded by the AFSGT (Association Française du Syndrome de Gilles de La Tourette/French Tourette Syndrome Association) and HoPeS (Institut des Handicaps Neurologiques, Psychiatriques et Sensoriels/Neurological, Psychiatric and Sensorial Disabilities Institute).

## OPR‐132

### Remote deep brain stimulation programming for Parkinson's disease: A prospective, randomized, controlled, multicentre, non‐inferiority trial

#### T. Köglsperger ^1^; J. Shaik^1^; W. Zhang^1^; V. Zentsch^2^; C. Buhmann^3^; U. Hidding^3^; T. Kinfe^4^; M. Regensburger^5^; J. Rumpf^6^; A. Schnitzler^7^; J. Dong^1^; M. Scherer^1^; E. Kaufmann^1^; G. Höglinger^1^; J. Mehrkens^8^

##### ^*1*^ *Department of Neurology, LMU University Hospital, Ludwig Maximilian University of Munich, Germany;* ^*2*^ *Department of Neurology, University of Münster, Germany;* ^*3*^ *Department of Neurology, University Medical Center Hamburg‐Eppendorf, Germany;* ^*4*^ *Dr. Rolf M. Schwiete Endowed Chair for Neuromodulation and Neuroprosthetics, Medical Faculty Mannheim, Ruprecht‐Karls‐University Heidelberg, Germany;* ^*5*^ *Department of Molecular Neurology, Friedrich‐Alexander Universität (FAU) of Erlangen‐Nürnberg, Erlangen, Germany;* ^*6*^ *Department of Neurology, University of Leipzig, Leipzig, Germany;* ^*7*^ *Institute of Clinical Neuroscience and Medical Psychology, Medical Faculty, Heinrich Heine University, Düsseldorf, Germany; Department of Neurology, Medical Faculty, Heinrich Heine University, Düsseldorf, Germany;* ^*8*^ *Department of Neurosurgery, Ludwig Maximilian University of Munich, Germany*

**Background and Aims:** Deep brain stimulation (DBS) is an effective therapy for advanced Parkinson's disease (PD), but outcomes depend on expert programming, usually requiring in‐person visits to specialised centres. Remote DBS programming could improve access, yet controlled prospective evidence is limited.

**Methods:** We conducted a prospective, multicentre, randomised, open‐label, non‐inferiority trial at DBS centres in Germany. Patients with idiopathic PD requiring DBS programming were assigned (1:1) to standard in‐person programming (standard of care [SoC]) or remote programming via a secure telemedicine platform. The primary endpoint was the MDS‐UPDRS Part III score (medication‐on/stimulation‐on) at 3 months, with a non‐inferiority margin of 5 points. Secondary outcomes included motor and non‐motor symptoms, quality of life, cognition, levodopa equivalent daily dose (LEDD), and safety. Analyses were per protocol (ClinicalTrials.gov NCT05193825).

**Results:** Between March 2022 and December 2024, 52 patients were randomised; 48 were analysed (SoC *n* = 26; remote *n* = 22). Median MDS‐UPDRS Part III improvement at 3 months was −23.0 points in the SoC group and −20.5 in the remote group. The median between‐group difference was −2.5 (95% CI −9.0 to 2.0), meeting non‐inferiority criteria. Improvements in total MDS‐UPDRS, Hoehn and Yahr stage, and PDQ‐39 were similar. MoCA scores improved more with remote programming, while LEDD reduction was greater with SoC. Adverse events and serious adverse events were comparable between groups.

**Conclusion:** Remote DBS programming was non‐inferior to in‐person programming for motor outcomes at 3 months, with similar safety and patient‐reported benefits, supporting its integration into routine DBS care.

**Disclosure:** Abbott Medical Inc.

## OPR‐133

### Does DaTScan have a prognostic value for motor decline in Parkinson's disease patients? A multimodal deep learning study

#### F. Ragni^1^; W. Endrizzi ^1^; S. Bovo^1^; M. Malaguti^2^; C. Longo^2^; N. Campese^2^; M. Moroni^1^; G. Jurman^1^

##### ^*1*^ *Fondazione Bruno Kessler, Trento, Italy;* ^*2*^ *U.O. Neurologia, Azienda Sanitaria Universitaria Integrata del Trentino (ASUIT), Trento, Italy*

**Background and Aims:** Parkinson's disease (PD) trajectories vary widely among individuals, making it difficult to predict disease progression and to identify patients at risk of rapid decline. Among routinely collected data from PD patients there is DaTscan, whose prognostic value for motor complications remains debated. This study implements state‐of‐the‐art Deep Learning (DL) architectures to investigate the prognostic value of DaTscan volumes and electronic health records (EHRs) data, separately and in multimodal combination, in predicting future motor decline.

**Methods:** A longitudinal cohort of 513 patients at different PD stages from the Parkinson's Progression Markers Initiative dataset with EHRs data, DaTscan volumes at baseline and UPDRS Part III evaluation at 3‐year follow‐up was used to develop a dual‐branch DL architecture. The first branch employs a tabular foundation model for EHRs data, while the second extracts compressed embeddings from DaTscan volumes using a 3D Convolutional Neural Network. The embeddings are then merged for regression of the UPDRS Part III evaluation at 3‐year follow‐up. This modular design facilitated an ablation study to quantify the contribution of each modality.

**Results:** Model performances were evaluated using Mean Absolute Error (MAE) and R2 on a three‐fold cross validation scheme (Table 1). The unimodal EHRs‐based model achieved a MAE of 6.51 ± 0.03 (mean ± SD), comparable to the complex multimodal architecture.

**TABLE 1** Mean Performance Metrics. Averaged scores across the three folds for the uni‐ and multimodal architectures.

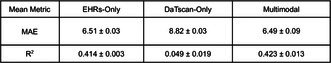

**Conclusion:** Despite its diagnostic relevance, DaTscan provides limited prognostic benefits beyond standard clinical features. The EHRs‐only model offers comparable performance while reducing model complexity and computational demands. Future prognostic modeling should prioritize richer clinical characterization over striatal imaging features.

**Disclosure:** Nothing to disclose.

## Epilepsy 2

## OPR‐134

### Dysregulated CX3CR1 signaling in mesial temporal lobe epilepsy: Evidence from human brain tissue and monocyte‐derived microglia

#### B. Guerra Leal ^1^; C. Carvalho^2^; P. Beça^2^; C. Santos^3^; R. Samões^4^; C. Teixeira^5^; P. Pinho e Costa^1^; M. Lobo^6^; P. Correia de Sá^6^; J. Chaves^4^

##### ^*1*^ *Unit for Multidisciplinary Research in Biomedicine (UMIB,ICBAS‐UPorto); Immunogenetics Laboratory, School of Medicine and Biomedical Sciences, University of; Laboratory for Integrative and Translational Research in Population Health (ITR), Porto, Portugal;* ^*2*^ *Immunogenetics Laboratory, Department of Molecular Pathology and Immunology, School of Medicine and Biomedical Sciences, University of Porto, Porto, Portugal;* ^*3*^ *Unit for Multidisciplinary Research in Biomedicine (UMIB,ICBAS‐UPorto); Immunogenetics Laboratory, Department of Molecular Pathology and Immunology, School of Medicine and Biomedical Sciences, University of Porto, Porto, Portugal;* ^*4*^ *Unit for Multidisciplinary Research in Biomedicine; Santo António Hospital, Santo António Local Health Unit, Porto, Portugal;* ^*5*^ *Santo António Hospital, Santo António Local Health Unit, Porto, Portugal;* ^*6*^ *Pharmacology and Neurobiology Laboratory Neurobiology, Center for Drug Discovery and Innovative Medicines (MedInUP), ICBAS‐UP, Porto, Portugal*

**Background and Aims:** CX3CR1, the fractalkine (CX3CL1) receptor, is expressed by CNS‐resident microglia and mediates microglia–neuron communication. Chronic microglial activation is a hallmark of mesial temporal lobe epilepsy with hippocampal sclerosis (MTLE‐HS) and contributes to neuroinflammation and neuronal hyperexcitability. Seizure‐induced release of CX3CL1 may activate microglial CX3CR1, amplifying inflammatory signaling. We studied this pathway by analyzing CX3CR1 expression in human epileptic brain tissue and in an in vitro model of monocyte‐derived microglia‐like cells (MMG).

**Methods:** MMG cells were differentiated from peripheral blood monocytes of 15 MTLE‐HS patients and 5 healthy donors. CX3CR1 mRNA levels were assessed under basal conditions and following lipopolysaccharide (LPS) stimulation. The findings were validated in hippocampal and temporal neocortex samples from 19 MTLE‐HS (8 males, 43.5 ± 10.0 years) patients and 10 controls (8 males, 67.0 ± 10.0 years).

**Results:** MMG cells derived from MTLE‐HS patients showed higher basal CX3CR1 expression compared to controls, with further upregulation after inflammatory stimulation. In parallel, CX3CR1 transcripts were significantly increased in both the hippocampus and the temporal neocortex of MTLE‐HS patients (1.96‐fold, *p* = 0.004; 2.23‐fold, *p* < 0.01, respectively).

**Conclusion:** Together, these concordant in vitro and in vivo findings suggest a primed, maladaptive microglial activation state in MTLE‐HS. Although CX3CR1 is typically associated with microglial homeostasis, its sustained upregulation in chronic epilepsy may contribute to persistent neuroinflammation. These results highlight the CX3CL1/CX3CR1 axis as a potential therapeutic target to modulate microglia–neuron interactions and reduce seizure susceptibility in drug‐resistant epilepsy.

**Disclosure:** UMIB is funded by FCT Portugal (UID/215/2025; UID/PRR/00215/2025). Work funded by a FCT grant 2022.10372.PTDC. No other disclosures.

## OPR‐135

### Pharmacokinetics of cenobamate in focal epilepsy: Influence of sex and concomitant antiseizure medications

#### F. Operto ^1^; B. Charlier^2^; V. Izzo^2^; G. Assenza^3^; A. Balsamo^2^; F. Cirillo^4^; A. Coglianese^2^; C. Di Bonaventura^5^; M. Fernandes^4^; A. Gambardella^1^; E. Cerulli Irelli^5^; C. Liguori^4^; G. Pastorino^1^; S. Rufolo^2^; I. Sammara^1^; A. Filippelli^2^

##### ^*1*^ *University of Catanzaro, Italy;* ^*2*^ *University of Salerno;* ^*3*^ *Campus Bio‐Medico University of Rome, Italy;* ^*4*^ *Tor Vergata University of Rome;* ^*5*^ *Sapienza University of Rome, Rome, Italy*

**Background and Aims:** Cenobamate (CNB) is a recently approved antiseizure medication (ASM) for drug‐resistant focal epilepsy. Despite its proven efficacy, real‐world pharmacokinetic data, particularly regarding sex‐based variations and interactions with concomitant ASMs, remain limited. This study aimed to investigate the pharmacokinetic profile of CNB in clinical practice and evaluate its relationship with demographic and clinical variables.

**Methods:** We conducted an exploratory study on 17 adult responder patients receiving add‐on CNB. Plasma concentrations were measured at each dose titration step using a validated method. Pharmacokinetic parameters, including concentration‐to‐dose ratio, incremental slope (delta C/delta D), and area under the dose‐concentration curve (AUC), were calculated. Patients were stratified into three exposure clusters (low, medium, and high) based on AUC distribution.

**Results:** A significant association was observed between sex and AUC exposure clusters (*p* = 0.026), with females predominating in the high‐exposure group. The analysis revealed a nonlinear dose‐concentration relationship, characterized by steeper incremental slopes at lower doses (12.5–50 mg) and increased variability at higher dosages (100–200 mg). Regarding drug interactions, higher CNB concentrations were observed in patients co‐treated with lacosamide (*p* = 0.0335), while concomitant topiramate was associated with lower exposure (*p* = 0.0044).

**Conclusion:** The pharmacokinetics of cenobamate appear highly variable and are influenced by sex and concomitant ASMs. These findings highlight the importance of therapeutic drug monitoring and individualized titration strategies to optimize efficacy and safety in real‐world clinical practice.

**Disclosure:** Nothing to disclose.

## OPR‐136

### The effect of subacute neocortical stimulation during intracranial EEG on seizure frequency

#### I. Stavropoulos ^1^; D. Jiménez‐Jiménez^2^; S. Freigang^3^; J. Díaz‐Díaz^1^; F. Kazi^1^; M. Pina^4^; B. Diehl^2^; R. Selway^5^; G. Alarcón^1^; A. Valentin^1^

##### ^*1*^ *Department of Basic and Clinical Neuroscience, Institute of Psychiatry, Psychology and Neuroscience. King's College London, UK;* ^*2*^ *Department of Clinical and Experimental Epilepsy, UCL Queen Square Institute of Neurology, London, UK;* ^*3*^ *Department of Neurosurgery, Medical University Graz, Austria;* ^*4*^ *Department of Clinical Neurophysiology, King's College Hospital NHS Trust, London, UK;* ^*5*^ *Department of Neurosurgery, King's College Hospital, NHS Trust London, UK*

**Background and Aims:** Neurostimulation is an alternative therapy for patients with drug‐resistant epilepsy (DRE) who are not candidates for resective surgery. This study evaluates the impact of Subacute Neocortical Stimulation (SNCS) during intracranial presurgical assessment on seizure frequency.

**Methods:** This study was approved by the HRA ethical committee (IRAS 187072). Patients undergoing intracranial EEG with subdural or depth electrodes for epilepsy surgery evaluation were recruited. SNCS was initiated after sufficient seizures were captured for clinical purposes and reinstatement of all antiseizure medications. Following a 24‐h baseline, SNCS was applied for approximately 48 h using different stimulation parameters (varying electrode pairs and cortical locations), separated by periods without stimulation. Each stimulation period involved one electrode pair. Stimulation targeted electrode pairs located within MRI‐visible lesions, the seizure onset zone and/or areas with high interictal epileptiform discharge rates, or delayed responses to single‐pulse electrical stimulation (450 μs pulse width, up to 2 V or 2.5 mA, 130 Hz). Seizure rate ratio per hour (SRR/h) was calculated as the seizure rate during SNCS divided by the baseline rate.

**Results:** Twenty‐eight patients were included (11 females; mean age 25 ± 44). Median SRR/h during baseline was 0.296 (±3.57) and decreased to 0.0947 (±4.27) during SNCS (*p* = 0.0179). Twenty‐three patients showed a reduction in SRR/h (median reduction 77%), with 17 demonstrating reductions greater than 50%.

**Conclusion:** SNCS is safe and significantly reduces seizure frequency. It may represent a therapeutic alternative for patients unsuitable for resective surgery and could serve as an additional marker of epileptogenic tissue, warranting further investigation.

**Disclosure:** This study was funded by Epilepsy Research UK and by a jointly grant from both Action Medical Research and Great Ormond Street Hospital Children's Charity.

## OPR‐137

### Integrative approach to transient loss of consciousness: Guided protocol of differential diagnosis

#### G. Nadejda ^1^; C. Carolina^2^; D. Diana^1^; S. Siluan^1^; M. Serghei^3^; B. Corneliu^4^; G. Stanislav^5^

##### ^*1*^ *Epileptology Department, Institute of Emergency Medicine, Chisinau, Republic of Moldova;* ^*2*^ *Department of Neurosciences Cliniques, Centre Hauspitalier Universitaire Vuadois (CHUV), Lausanne, Switzerland;* ^*3*^ *Cardiology Department, “Holy Trinity” Municipal Clinical Hospital, Chisinau, Republic of Moldova;* ^*4*^ *Nuffield Department of Primary Care Health Sciences, Oxford University, Oxford, UK;* ^*5*^ *Department of Neurology nr. 2, State University of Medicine and Pharmacy “Nicolae Testemițanu”, Chisinau, Republic of Moldova*

**Background and Aims:** Transient loss of consciousness (TLOC) represents a spontaneous loss of consciousness with complete resolution being a common and serious presentation in the emergency department (ED). Despite the existence of evidence‐based recommendations, the diagnostic process is frequently fragmented, discipline‐specific and sequential. This leads to a significant proportion of cases remaining either unexplained or misclassified. The objective of this study is to develop an integrated neuro‐cardiology protocol to reduce “unexplained” TLOC after initial ED assessment.

**Methods:** We conducted a structured review of the literature (PubMed/Embase/Cochrane) from 2000 to 2025 to identify existing diagnostic frameworks approaches for TLOC and their limitations in practice. In parallel, we performed a prospective, single‐centre pilot study including adult patients, presenting with unexplained TLOC after an initial standard assessment. We applied a standardised diagnostic pathway involving the targeted use of neuroimaging, prolonged simultaneous ECG, arterial tension and EEG monitoring, echocardiography and tilt‐table testing. Final diagnoses were adjudicated using ESC syncope guidelines and ILAE seizure definitions, with clinical follow‐up.

**Results:** No fully operationalised, integrated diagnostic protocol for TLOC applicable to the neurological and cardiological settings was identified in the literature revising 1470 publications. Implementation of the structured multidisciplinary pathway in our cohort substantially reduced the proportion of unexplained TLOC cases and clarified the diagnostic yield of individual investigations when applied within a coordinated framework.

**Conclusion:** The evaluation of TLOC should evolve from isolated neurological or cardiological reasoning towards structured, synchronised diagnostic pathways. Translating the existing guidance in the form of a standardised protocol improves diagnostic accuracy, reduces uncertainty and enhances patient safety.

**Disclosure:** The authors declare no conflicts of interest.

## OPR‐138

### Mortality and SUDEP in developmental and epileptic encephalopathies: A bayesian meta‐analysis of rcts and open‐label extensions

#### P. Moro; M. Borioni; A. Mazzeo; C. Di Bonaventura; E. Cerulli Irelli

##### Department of Human Neurosciences, Sapienza Università di Roma, Rome, Italy

**Background and Aims:** Mortality and Sudden Unexpected Death in Epilepsy (SUDEP) are major concerns in Developmental and Epileptic Encephalopathies (DEEs), particularly Lennox–Gastaut (LGS) and Dravet syndromes (DS). Accurate incidence estimates from prospectively monitored populations are limited. We aimed to estimate long‐term rates of all‐cause mortality and SUDEP by pooling data from RCTs and their corresponding long‐term open‐label extensions (OLEs) using a Bayesian framework.

**Methods:** We systematically reviewed pharmacological trials in DEEs and performed a Bayesian random‐effects Poisson meta‐analysis with informative priors derived from published literature. Sensitivity analyses were conducted using weakly informative priors and frequentist generalized linear mixed models. Results are expressed as incidence per 1000 person‐years (PY) with 95% Credible Intervals (CrI).

**Figure:**
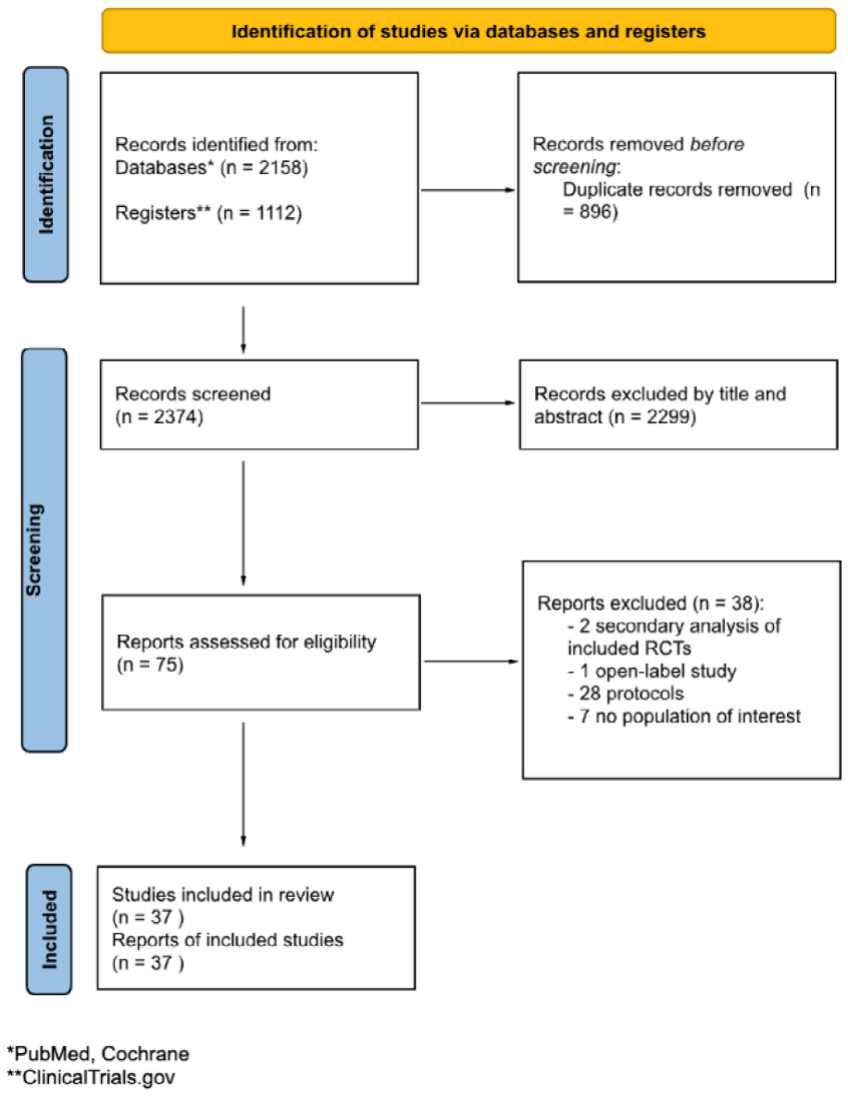
**FIGURE 1** PRISMA 2020 flow diagram for new systematic reviews which included searches of databases and registers only.

**Results:** The analysis included 37 RCTs (3757 patients) and 16 OLE studies (2210 patients). In the overall population, all‐cause mortality was 8.17 per 1000 PY (95% CrI 4.77–11.84), and SUDEP was 3.96 (2.24–6.12). These estimates were consistent across sensitivity analyses. In LGS, all‐cause mortality was 7.77 (3.58–13.31) and SUDEP 3.73 (1.73–6.49) per 1000 PY. In DS, all‐cause mortality was comparable (8.97; 3.84–16.99), whereas SUDEP incidence appeared higher at 7.29 (2.88–14.42).

**Conclusion:** This study provides robust long‐term estimates of mortality and SUDEP in DEEs by leveraging pooled trial data. The consistency between Bayesian and frequentist approaches validates these findings, highlighting a substantial mortality burden, with SUDEP particularly elevated in DS. These results emphasize the need for proactive risk management and counseling in this vulnerable population.

**Disclosure:** Nothing to disclose.

## Sleep‐Wake Disorders

## OPR‐139

### Cognitive impairment and cardiovascular autonomic failure as independent and combined predictors of progression and phenoconversion in iRBD

#### L. Baldelli^1^; F. Di Laudo^1^; L. Sambati^2^; D. Cimatti ^1^; P. Guaraldi^2^; G. Giannini^1^; A. Cecere^1^; G. Loddo^3^; G. Mainieri^2^; F. Mignani^1^; G. Barletta^1^; P. Cortelli^1^; G. Calandra‐Buonaura^1^; F. Provini^1^

##### ^*1*^ *Department of Biomedical and NeuroMotor Sciences, University of Bologna, Bologna, Italy;* ^*2*^ *IRCCS Istituto delle Scienze Neurologiche di Bologna, Bologna, Italy,* ^*3*^ *Dipartimento delle Cure Primarie, Azienda AUSL di Bologna, Bologna, Italy*

**Background and Aims:** REM sleep behaviour disorder (RBD) is a parasomnia caused by loss of physiological muscle atonia during REM, resulting in dream‐enactment behaviours. Isolated RBD (iRBD) is recognized as a prodromal manifestation of α‐synucleinopathies, including Parkinson's disease (PD), dementia with Lewy bodies (DLB), and multiple system atrophy (MSA). Although cardiovascular autonomic failure (cAF) and cognitive impairment (CI) are established prodromal biomarkers, their combined prognostic significance remains unclear. This study investigated the interaction between cAF and CI in iRBD at baseline and longitudinally, evaluating their individual and combined phenoconversion predictive value.

**Methods:** Seventy‐four iRBD (73% male; age 68.61 ± 7.23 years) were prospectively enrolled and followed‐up for a mean of 3.17±1.88 years. Cardiovascular autonomic function was assessed using a standardized reflex testing battery alongside a comprehensive neuropsychological battery. Cross‐sectional and longitudinal cognitive and autonomic changes were analyzed, conversion risk was assessed using survival analyses.

**Results:** At baseline, 17 patients (25.4%) had mild CI (MCI) and 12 (17.9%) had neurogenic orthostatic hypotension (nOH), with no significant association between them (*p* = 0.484). Longitudinally, multidomain MCI increased significantly (*p* = 0.038), driven by visuospatial (*p* = 0.001) and memory decline (*p* = 0.050), and nOH prevalence also rose (*p* = 0.026). Fifteen patients (20.3%) converted to an α‐synucleinopathy (6 DLB, 5 PD, 2 MSA, 2 parkinsonism). Both MCI (HR = 3.71; 95% CI: 1.22–11.22; *p* = 0.020) and nOH (HR = 3.64; 95% CI: 1.20–10.98; *p* = 0.022) independently predicted conversion, while their coexistence markedly increased risk (HR = 12.12; 95% CI: 2.76–53.29; *p* = 0.001).

**Conclusion:** In iRBD, CI and cAF evolve largely independently, but their coexistence strongly increases the risk of phenoconversion, supporting multidimensional biomarker‐based risk stratification.

**Disclosure:** Nothing to disclose.

## OPR‐140

### Lateralisation of dream‐enacting gestures during rapid eye movement sleep behaviour disorder

#### D. Hoxhaj ^1^; J. Maranci^2^; S. Leu‐Semenescu^2^; P. Dodet^2^; A. Pascazio^2^; E. Bonanni^1^; M. Maestri Tassoni^1^; I. Arnulf^2^

##### ^*1*^ *Department of Clinical and Experimental Medicine, Neurology Unit, University of Pisa, Italy;* ^*2*^ *Paris Brain Institute (ICM), DreamTeam, Paris, France*

**Background and Aims:** Are gestures in dreams produced symmetrically or as an inverted mirror image of real‐life gestures? Evidence from actigraphy and analyses of nocturnal movements in sexual parasomnia during NREM sleep suggests that motor activity during sleep may shift toward the non‐dominant hand. We aimed to investigate whether a similar state‐dependent inversion of motor lateralisation occurs in REM sleep behaviour disorder (RBD), a condition in which dream‐enacting movements can be directly observed.

**Methods:** Consecutive patients with RBD underwent a structured interview to assess handedness, and their video‐polysomnography recordings were visually analysed. Upper limb movements during wakefulness and REM sleep were annotated according to laterality (right, left, bilateral), movement type (simple, complex, jerk‐like), and duration. Linear mixed‐effects models were used to examine the interaction between behavioural state (wakefulness vs. REM sleep) and motor lateralisation.

**Results:** Seventy‐one patients were included: 33 with isolated RBD and 38 with RBD associated with parkinsonism (13 Parkinson's disease, 25 multiple system atrophy). Most participants were right‐handed (65/71, 91.5%). A total of 22,066 movements were analysed. During wakefulness, right‐handed individuals showed longer right‐arm movements, consistent with normal motor dominance. In contrast, during REM sleep this pattern was reversed: right‐sided movements became shorter and less frequent, while left‐sided movements predominated, particularly for complex gestures. Jerks were largely symmetrical. Among right‐handed patients, 72% exhibited leftward motor predominance during REM sleep. Importantly, the side affected by parkinsonism did not abolish this inversion.

**Figure:**
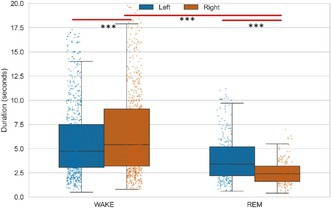
**FIGURE 1** Boxplot of mean simple and complex movement duration per subject(s) across wakefulness and REM sleep. (*): *p* < 0.05, (***): *p* < 0.001 Each box represents the distribution of subject‐level mean durations for movements performed with the left (blue) or righ.

**Figure:**
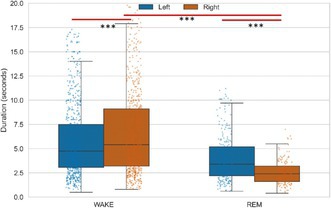
**FIGURE 2** Distribution of individual complex movement durations during wakefulness and REM sleep. Each dot represents a single movement performed with the left (blue) or right (orange) arm, while boxes indicate the interquartile range (IQR) and whiskers extend to 1.

**Conclusion:** Dream‐enacting movements in RBD are preferentially expressed through the non‐dominant hand, suggesting a transient reversal of motor lateralisation during REM sleep.

**Disclosure:** Nothing to disclose.

## OPR‐141

### Mass spectrometry‐based quantification of hypocretin‐1 and its fragment_1‐16 following immunoprecipitation and solid‐phase extraction

#### E. Wenz ^1^; J. Prost^2^; S. Braga‐Lagache^3^; G. Mäder^2^; J. Warncke^1^; M. Luginbühl^4^; P. Wüthrich^4^; F. Lersch^4^; C. Schankin^1^; I. Filchenko^1^; L. Fregolente^1^; K. Zub^1^; S. Nishino^5^; C. Largiadèr^2^; M. Heller^3^; M. Tafti^6^; M. Schmidt^1^; C. Bassetti^1^

##### ^*1*^ *Department of Neurology, Inselspital, Bern University Hospital, University of Bern, Bern, Switzerland;* ^*2*^ *Department of Clinical Chemistry, Inselspital, Bern University Hospital and University of Bern, Switzerland;* ^*3*^ *Department for Biomedical research, Core Facility Proteomics & Mass Spectrometry, University of Bern, Switzerland;* ^*4*^ *Department of Anaesthesiology and Pain Medicine, Inselspital, Bern University Hospital, University of Bern, Bern, Switzerland;* ^*5*^ *Sleep and circadian neurobiology laboratory, School of Medicine, Stanford University, Stanford, USA;* ^*6*^ *Department of Biomedical Sciences, University of Lausanne, Lausanne, Switzerland*

**Background and Aims:** Loss of hypocretin‐1/orexin‐A (Hcrt‐1) immunoreactivity in cerebrospinal fluid is a biomarker for type 1 narcolepsy (NT1). However, the current radioimmunoassay (RIA) does not allow for precise quantification, which limits the assessment of disease severity and progression. Three previous mass‐spectrometry studies showed mixed results. Following the identification of RIA antibody targets by immunoprecipitation (IP), this study aimed to compare the ability of two purification methods (IP and solid phase extraction (SPE)) followed by nano‐liquid chromatography coupled to high‐resolution mass spectrometry (nLC‐HRMS) to identify patients with NT1.

**Methods:** IP with magnetic beads coupled to the RIA antibody (Phoenix Pharmaceuticals) was performed, and the resulting peptides were identified by MS using data‐dependent acquisition. Full‐length Hcrt‐1 and Hcrt‐1 fragment_1–16 levels were analyzed in 4 NT1 patients and 4 controls after both IP and SPE by nLC‐HRMS.

**Results:** The discovery experiment with IP most reliably revealed Hcrt‐1 fragment_1‐16. SPE generally revealed much higher concentrations than IP. Hcrt‐1_1‐16 levels enabled the distinction between patients and controls after both IP (controls 0.94 pg/mL [0.71–1.21] NT1 0.15 pg/mL [0.04–0.30], *p* = 0.029) and SPE (controls 29.19 pg/mL [21.67–37.55], NT1 1.66 pg/mL [0.49–2.57], *p* = 0.029), whereas full‐length Hcrt‐1 levels after IP (controls 1.48 pg/mL [0.89–2.33], NT1 2.26 pg/mL [1.86–2.89], *p* = 0.2) did not.

**Conclusion:** Hcrt‐1_1‐16 appears stable and abundant in the CSF of controls and allows the identification of patients with NT1, making it a promising surrogate marker and confirming results of a recent study. SPE seems to be a more suitable method for further validation in clinical practice.

**Disclosure:** The authors declare no conflict of interest. The international Swiss Primary Hypersomnolence and Narcolepsy Cohort study is supported by the Swiss National Science Foundation (SNF 320030_185362; SNF 32003B_215721).

## OPR‐142

### Brain and heart cross‐talk: Cortical topography of slow‐wave–heart rhythm coupling is associated with cognition

#### I. Filchenko ^1^; M. Schmidt^1^; C. Bassetti^2^

##### ^*1*^ *Department of Neurology, Bern University Hospital (Inselspital) and University of Bern, Bern, Switzerland;* ^*2*^ *Medical Faculty, University of Bern, Bern, Switzerland*

**Background and Aims:** Cognitive dysfunction is associated with both cardiac autonomic dysfunction and reduced slow‐wave (SW) activity. Slow‐wave‐heart rhythm (SW‐HR) coupling reflects the interaction between SW and autonomic activity and is largely underexplored. We investigated its association with cognition.

**Methods:** “Sleep and cognitive functioning” (*n* = 63) included volunteers in good or excellent health condition. Demographics, medical history, cognition (11 tests), sleep architecture by polysomnography were assessed at study inclusion (Figure 1). SW‐HR coupling was quantified using previously described (Fabus et al., 2024) and newly conceptualized metrics in terms of temporal coherence, SW amplitude, and SW globality (Figure 2). Associations between SW–HR coupling (dependent variables) and cognition (independent variables) were explored using multivariate linear regression, adjusted for age and arousal index at the level of individual electrodes for all parameters except globality. The significance level was set at α = 0.05.

**Figure:**
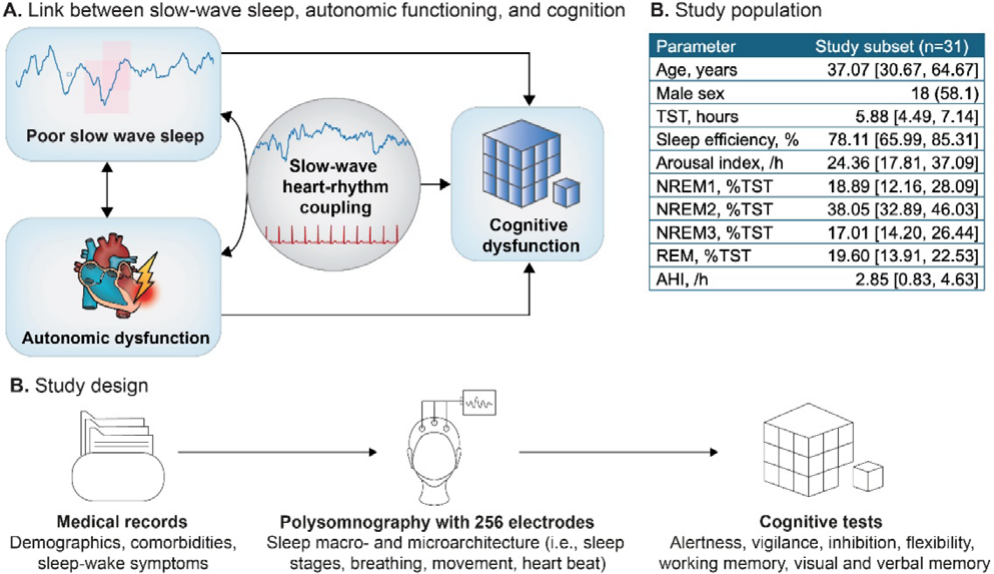
FIGURE 1

**Figure:**
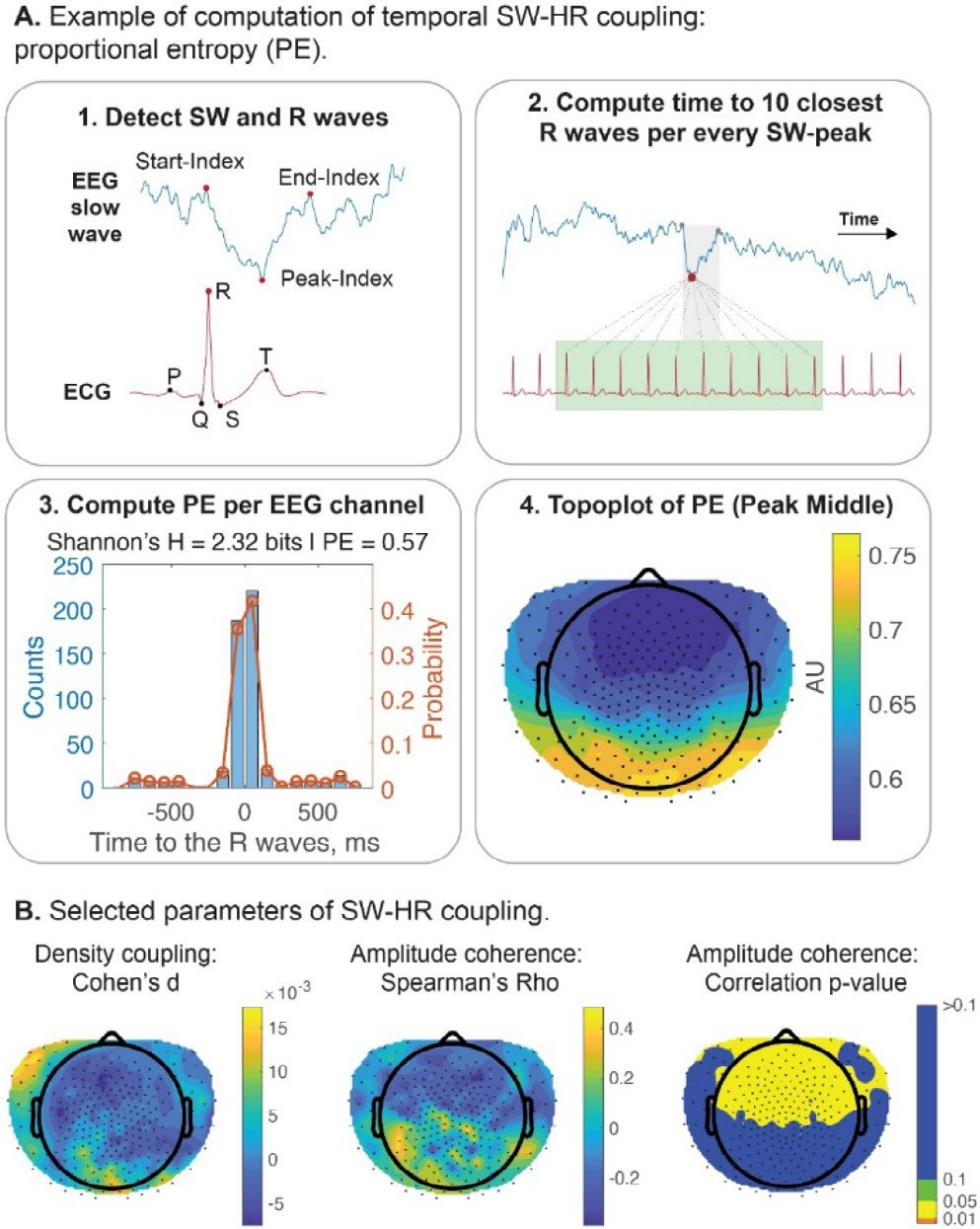
FIGURE 2

**Results:** A subset of 31 participants with 256‐electrode electroencephalography was analyzed. SW‐HR coupling was bidirectional and mostly involved fronto‐central cortical areas. Stronger temporal and amplitude coupling was associated with alertness (frontal, left‐lateralized), response inhibition (frontal and occipital, left‐lateralized), verbal memory (frontal and occipital), and visual memory (central regions; Figure 3). Stronger globality coupling was associated with the same domains (e.g., significance with visuospatial memory recall: beta = 5.84 SD, *p* < 0.008). No associations were found for density coupling.

**Figure:**
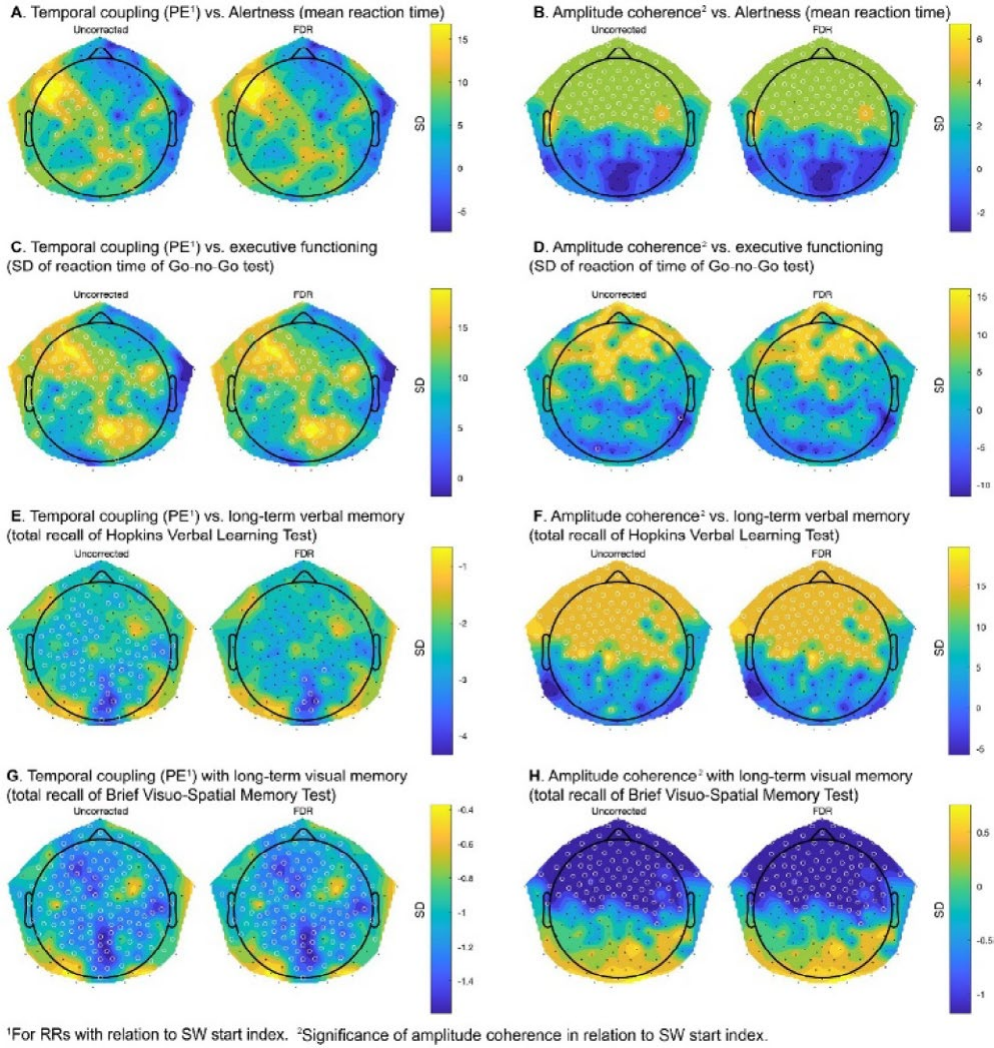
FIGURE 3

**Conclusion:** This is the first description of the cortical topography of SW‐HR coupling and it association with multiple cognitive domains. SW‐HR coupling may represent a promising marker of brain health.

**Disclosure:** Nothing to disclose.

## OPR‐143

### Behind the mirror: Clinical and neuroradiological significance of mirror movements in idiopathic REM sleep behavior disorder

#### R. Balestrino ^1^; E. Sarasso^2^; S. Basaia^3^; A. Gardoni^4^; S. Sangermano^5^; S. Marelli^5^; A. Castelnuovo^6^; A. Grassi^4^; E. Canu^4^; L. Ferini Strambi^5^; M. Filippi^7^; F. Agosta^8^

##### ^*1*^ *Neurology Unit, and Neurorehabilitation Unit, IRCCS San Raffaele Scientific Institute, Milan, Italy; and Neurotech Hub, Vita‐Salute San Raffaele University, Milan, Italy;* ^*2*^ *Neuroimaging Research Unit, Division of Neuroscience, IRCCS San Raffaele Scientific Institute, Milan, Italy; DINOGMI, University of Genoa, Genoa, Italy; Neurotech Hub, Vita‐Salute San Raffaele University, Milan, Italy;* ^*3*^ *Neuroimaging Research Unit, Division of Neuroscience, IRCCS San Raffaele Scientific Institute, Milan, Italy;* ^*4*^ *Neuroimaging Research Unit, Division of Neuroscience, IRCCS San Raffaele Scientific Institute, Milan, Italy; Neurotech Hub, Vita‐Salute San Raffaele University, Milan, Italy;* ^*5*^ *Sleep Disorders Center, Division of Neuroscience, IRCCS San Raffaele Scientific Institute, Milan, Italy; Vita‐Salute San Raffaele University, Milan, Italy;* ^*6*^ *Sleep Disorders Center, Division of Neuroscience, IRCCS San Raffaele Scientific Institute, Milan, Italy;* ^*7*^ *Neuroimaging Research Unit, Division of Neuroscience, Neurology Unit, Neurorehabilitation Unit, and Neurophysiology Service, IRCCS San Raffaele Scientific Institute, Milan, Italy; Neurotech Hub, Vita‐Salute San Raffaele University, Milan, Italy;* ^*8*^ *Neuroimaging Research Unit, Division of Neuroscience, and Neurology Unit, IRCCS San Raffaele Scientific Institute, Milan, Italy; Neurotech Hub, Vita‐Salute San Raffaele University, Milan, Italy*

**Background and Aims:** Idiopathic REM Sleep Behavior Disorder (iRBD) is a prodromal stage of synucleinopathies such as Parkinson's disease (PD). Mirror movements (MM) are involuntary contralateral mirroring of voluntary actions, considered para‐physiological in childhood and described in stroke, multiple sclerosis, and movement disorders, including PD. Their presence and significance in iRBD remain unexplored.

**Methods:** We enrolled 49 polysomnography‐confirmed iRBD patients, divided into MM‐positive (*n* = 33) and MM‐negative (*n* = 16) groups. MM were further classified as monolateral (*n* = 10) or bilateral (*n* = 23). All underwent neurological, cognitive (MMSE, FAB, Rey and Benson figures, Rey Word List, Corsi, Token Test), motor (UPDRS I–III, 9HPT, 5TSTS, 10MWT), and MRI evaluations. FLAIR, 3DT1, and 3DT2 sequences quantified juxtacortical, deep grey matter (DGM), periventricular, and infratentorial hyperintensities.

**Results:** MM‐positive patients showed lower upper limb dexterity (9HPT, *p* = 0.029) and reduced visuospatial‐executive function (Corsi, *p* = 0.004, *p* = 0.01). Monolateral MM were associated with younger age and worse visuospatial, memory, and language performance. Bilateral MM showed higher DGM hyperintensity volume (*p* = 0.049) and number (*p* = 0.029), with a trend toward higher periventricular burden (*p* = 0.071). Periventricular lesion volume and number correlated with worse visuospatial performance (Rey copy: *r* = −0.668, *p* = 0.002), mood symptoms (BDI‐II: *r* = 0.572, *p* = 0.013), and gait impairment (10MWT steps: *r* = 0.555, *p* = 0.014).

**Conclusion:** Mirror movements are common in iRBD and show distinct clinical and radiological patterns. Monolateral MM may indicate early neurodegenerative changes, while bilateral MM may reflect a less specific pattern. MM assessment could help stratify RBD patients.

**Disclosure:** R. Balestrino received travel reimbursement from Lusofarmaco, Angelini Pharma, Roche, Bial, Biogen, and speaking fees from Bayer and Editree; and research support from the Italian Ministry of Health. S. Basaia, E. Sarasso, and E. Canu received research support from the Italian Ministry of Health. A Gardoni, S Sangermano, S Marelli, A Castelnuovo, A Grassi report no competing interests. Luigi Ferini‐Strambi is field editor of the journal Sleep Medicine journal, associate editor of the Journal of Alzheimer's Disease, member of the editorial board of the European Journal of Neurology, and Behavioral Neurology, has received speaker honoraria from Biprojet, Idorsia, Italfarmaco, and Takeda, and receives or has received research support from the Italian Ministry of Health and the Italian Ministry of University and Research. M. Filippi is Editor‐in‐Chief of the Journal of Neurology, Associate Editor of Human Brain Mapping, Neurological Sciences, and Radiology; received compensation for consulting services from Almirall, Biogen, Bristol‐Myers Squibb, Eli Lilly, Merck, Novartis, Roche, Sanofi; speaking activities from Amgen, Bayer, Biogen, Bristol‐Myers Squibb, Celgene, Chiesi Italia SpA, Eisai, Eli Lilly, Fujirebio, Genzyme, Janssen, Merck, Neopharmed Gentili, Neuraxpharm, Novartis, Novo Nordisk, Roche, Sanofi, Takeda; participation in Advisory Boards for Alexion, Biogen, Bristol‐Myers Squibb, Eli Lilly, GE Healthcare Ltd, Merck, Neuraxpharm, Novartis, Roche, Sandoz, Sanofi, Takeda; scientific direction of educational events for Biogen, Merck, Roche, Celgene, Bristol‐Myers Squibb, Lilly, Novartis, Sanofi‐Genzyme; he receives research support from Biogen Idec, Merck‐Serono, Novartis, Roche, the Italian Ministry of Health, the Italian Ministry of University and Research, and Fondazione Italiana Sclerosi Multipla. F. Agosta is Associate Editor of NeuroImage: Clinical and the European Journal of Neurology; has received speaker honoraria from Biogen Idec, Bristol Myers Squibb, Eisai, Eli Lilly, GE Healthcare, Neuraxpharm, and Roche; and receives or has received research supports from the Italian Ministry of Health, the Italian Ministry of University and Research, AriSLA (Fondazione Italiana di Ricerca per la SLA), the European Research Council (ERC), the EU Joint Programme – Neurodegenerative Disease Research (JPND), and Foundation Research on Alzheimer Disease (France).

## Headache 2

## OPR‐144

### CSF proteomic alterations in spontaneous intracranial hypotension

#### A. El Rahal ^1^; K. Joseph^1^; K. Wolf^1^; B. Haupt^2^; F. Volz^1^; S. Behringer^1^; N. Neidert^1^; S. Tholen^3^; O. Schilling^3^; M. Overstijns^1^; N. Lützen^4^; H. Urbach^4^; C. Zander^4^; V. Ravi^1^; J. Beck^1^

##### ^*1*^ *Department of Neurosurgery, Medical Center University of Freiburg, Freiburg, Germany;* ^*2*^ *Department of Neurological Surgery, Feinberg School of Medicine, Northwestern University, Chicago, USA;* ^*3*^ *Proteomics Platform – Core Facility (ProtCF), Medical Center – University of Freiburg/Medical Faculty – University of Freiburg, Freiburg, Germany;* ^*4*^ *Department of Neuroradiology, Medical Center University of Freiburg, Freiburg, Germany*

**Background and Aims:** Spontaneous intracranial hypotension (SIH) results from spinal CSF leaks, but there is sparse knowledge about metabolic sequelae, and no fluid biomarkers exist. We investigated the CSF proteome of SIH patients, aiming to identify disease signatures and potential biomarkers.

**Methods:** We analyzed CSF from 26 patients (9 with type I ventral dural leaks and 17 controls). Samples were analyzed by label‐free liquid chromatography‐tandem mass spectrometry after depletion and SP3‐based preparation. Data were evaluated using differential expression, weighted correlation network analysis (WGCNA), gene set variation analysis (GSVA) with 50 Hallmark pathways, and protein–protein interaction network centrality. Classification performance was assessed with cross‐validation and permutation testing.

**Results:** A total of 918 proteins were quantified, 311 were included in the final analysis, of which 31 were significantly dysregulated (FDR < 0.05). A 31‐protein panel discriminated leaks from controls with an AUC of 0.93 (*p* < 0.001). LYVE1, a regulator of extracellular matrix and fluid balance, was the most stable biomarker, potentially implicating meningeal lymphatic dysfunction in patients with CSF leaks. Network analysis highlighted ENPP2, GOT1, CTSD, and RELN as central hubs within the disease network. Functional clustering pointed out the downregulation of extracellular matrix remodeling (e.g., FAT2, CD44) and aberrant tissue repair proteins, coupled with the downregulation of metabolic and neuroprotective pathways (CYST C, ENPP2, GOT1, ALDOA).

**Figure:**
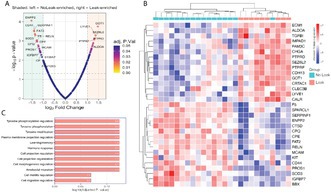
**FIGURE 1** CSF‐leak proteomic signature showing a leak‐associated proteomic fingerprint.

**Figure:**
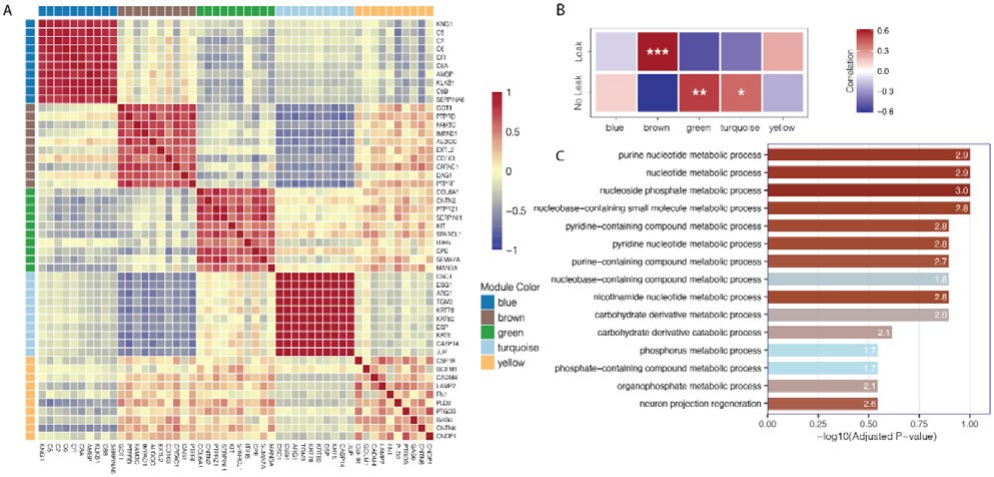
**FIGURE 2** Protein co‐expression network analysis of ventral CSF leaks reveals a specific leak‐associated protein module.

**Figure:**
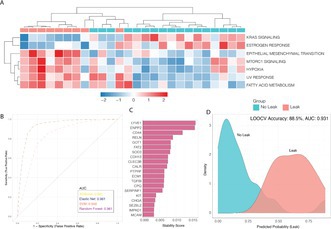
**FIGURE 3** Integrated stratification of leak and control samples, enabling group stratification with high accuracy.

**Conclusion:** CSF proteomics revealed that CSF leaks induced a distinct proteomic biological signature. Interestingly, we identified proteomic patterns commonly associated with tissue remodeling, lymphatic dysfunction, and cognitive disturbance. CSF proteomics may identify biomarkers for CSF leaks.

**Disclosure:** Nothing to disclose.

## OPR‐145

### MRI biomarker predicts headache burden in spontaneous intracranial hypotension

#### F. Volz^1^; A. El Rahal^1^; N. Lützen^2^; C. Zander^2^; M. Overstijns^1^; H. Urbach^2^; J. Beck^1^; K. Wolf ^1^

##### ^*1*^ *Department of Neurosurgery, Medical Center, University of Freiburg, Germany;* ^*2*^ *Department of Neuroradiology, Medical Center, University of Freiburg, Germany*

**Background and Aims:** Spontaneous intracranial hypotension (SIH) is caused by spinal cerebrospinal fluid (CSF) leaks and is classically characterized by orthostatic headache. Objective: To evaluate whether headache intensity is associated with clinical, anatomical, or dynamic MRI parameters.

**Methods:** Patients with SIH and a confirmed spinal CSF leak (12/2021–07/2024) were analyzed. Data included demographics, symptom duration, leak type, Bern score, HIT‐6, and phase‐contrast MRI–derived measures of CSF and spinal cord velocities at C2/C3. From these, derivatives as intradural volume displacements and accelerations as compliance surrogates (C = ΔV/ΔP) were calculated.

**Figure:**
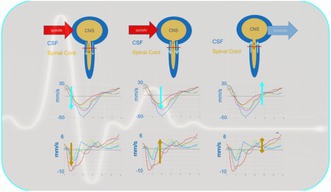
**FIGURE 1** Example of cardiac dynamic phase‐contrast MRI measurements of CSF and spinal cord velocities.

**Results:** 150 patients were included (57% female; mean age 46.6 ± 12 years). Leak types were ventral (60%), lateral (17.3%), CSF–venous fistula (21.4%), and sacral (1.3%). Median symptom duration was 8 months (IQR 3–21). Substantial to severe headache burden was reported by 81%, and 75% had predominantly orthostatic headaches. Conventional MRI findings and clinical variables did not predict headache severity. However, in patients with dural tears (ventral&lateral leaks), increased cranially directed CSF acceleration was strongly associated with orthostatic headache burden within the first 12 months (*R* = 0.574, adj. *R* ^2^ = 0.268, *p* = 0.012), particularly in early disease (<3 months: *R* = 0.909, adj. *R* ^2^ = 0.786, *p* < 0.001). These associations became diffuse in chronic disease, and in CSF–venous fistulas.

**Figure:**
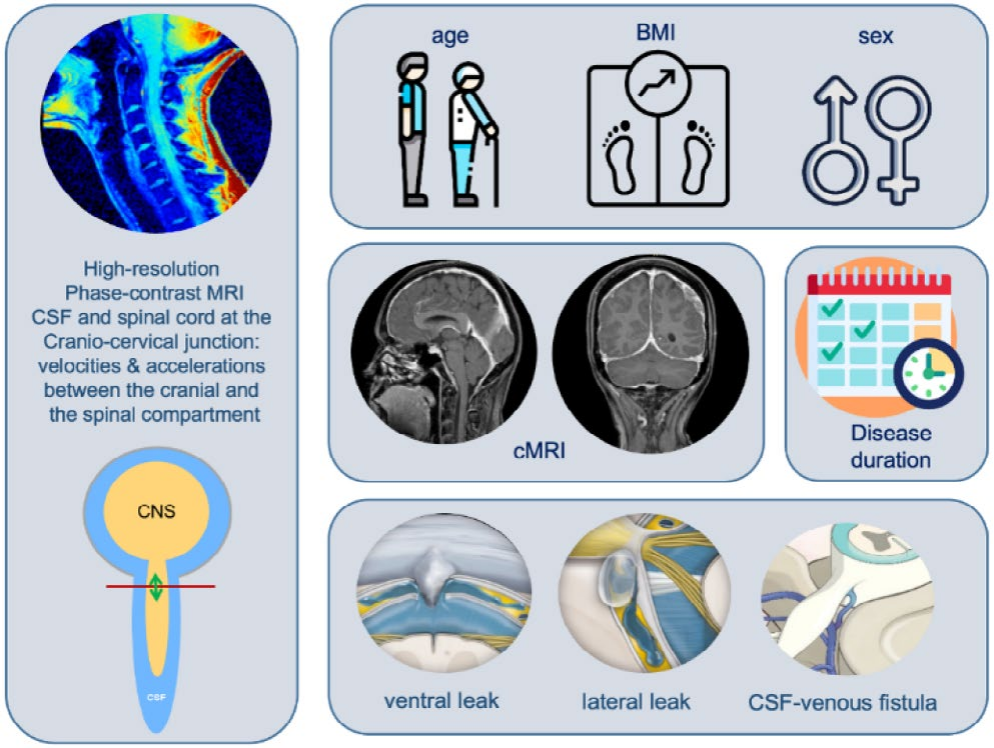
**FIGURE 1** Data assessed per patient.

**Figure:**
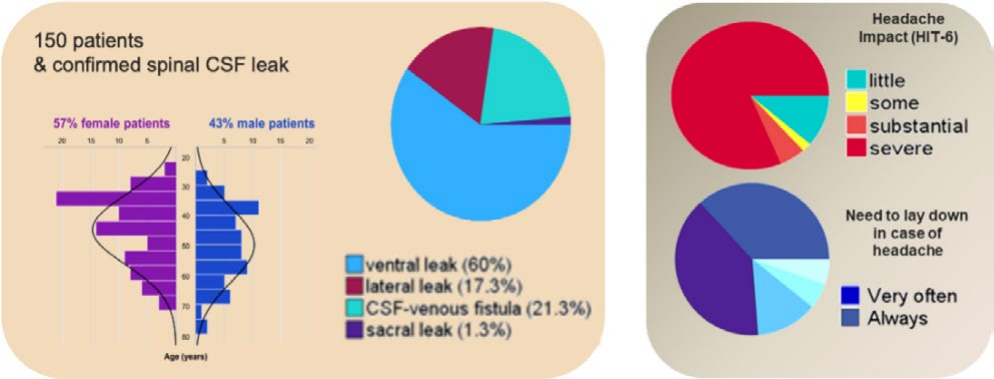
**FIGURE 2** Results on demographics, leak types and headache intensity.

**Conclusion:** SIH is associated with a high headache burden not explained by standard imaging or demographics. In early and intermediate disease, dynamic MRI biomarkers identify patients with greater orthostatic headache burden, whereas chronic SIH shows increasing heterogeneity, suggesting evolving pathophysiology.

**Disclosure:** none in relation to this study. KW is supported by the Margarete‐Von‐Wrangell‐Fellowship, Ministry of Science, Research and Arts, Baden‐Württemberg, Germany.

## OPR‐146

### Machine learning‐based statistical interpretation of headache pattern data: Insights from the myclusters application

#### L. Jabbarian ^1^; D. Ramanagoudra^2^

##### ^*1*^ *Department of Psychiatry, Erasmus Medical Center Rotterdam, The Netherlands;* ^*2*^ *MyCluste rs, Leiden, The Netherlands*

**Background and Aims:** Management of cluster headache (CH) remains a challenge due to its variable presentation patterns, idiosyncratic nature of attacks and the rarity of the disease. Most patients with CH experience a diagnostic delay and effective treatment strategies are limited. The MyClusters application collects real‐world global patient‐reported data on, among others, attack frequency, timing and patient characteristics. The aim of the app is to aid pattern recognition, both on an individual as well as population level.

**Methods:** As of now, 2060 patients with (cluster) headaches have input 21,048 data points on attack timing, duration of the attack, intensity, symptoms, medications and trigger factors. An unsupervised machine learning model was trained for automated statistical analyses, to identify and classify clinically relevant headache attack patterns, predict bout periods, and identify personalised risk factors with trigger fingerprinting.

**Results:** The model successfully classified five distinct attack pattern subtypes with significant differences in treatment response. Personalised trigger identification showed 82% concordance with patient‐reported associations, revealing temporal relationships between lifestyle factors and attack occurrence.

**Conclusion:** This machine learning‐based tool provides objective, automated statistical analysis of MyClusters data, enabling early bout detection, pattern classification and personalised trigger identification. Integration of artificial intelligence with patient‐reported outcomes offers promising applications for improving management of CH, potentially informing treatment decisions. This shows promise of further analysis by adding more data points by integrations with wearables and weather data to establish more pattern based sub‐types.

**Disclosure:** Nothing to disclose.

## OPR‐147

### Beyond the aura: Is burden measurable in migraine?

#### Ö. Soylu; F. Bachhuber; P. Klassen; H. Tumani

##### Department of Neurology, Ulm University Hospital, Ulm, Germany

**Background and Aims:** Migraine is traditionally considered a benign disorder; however, it can be sorely disabling. Its pathogenesis includes multiple mechanisms, notably neurogenic inflammation and neuroaxonal involvement. Therefore, a measurable biomarker to demonstrate subclinical disease burden is needed.

**Methods:** We included 86 patients with migraine (62 with aura [MWA]; 24 without aura [MWOA]) (classified according to ICH 3rd beta Version), and 126 (age‐ and sex‐matched) control subjects (tension‐type headache, dizziness, functional disorder [Controls]). Individuals with comorbidities associated with neuroaxonal damage, obesity, or renal insufficiency were excluded. By the selection of Controls, we aimed to strengthen the clinical relevance of the study. Serum neurofilament light chain (sNfL) and glial fibrillary acidic protein (sGFAP) were analyzed on a sensitive immunoassayplatform. Age‐ and sex‐adjusted biomarker levels were derived using linear regression residuals. Comprehensive demographic and clinical characteristics were considered.

**Results:** sNfL levels differed significantly between patients with migraine groups and Controls (Kruskal–Wallis, *p* = 0.004). In contrast, sGFAP levels did not differ between the groups. The subgroup analysis revealed higher sNfL levels in patients with MWOA (median 0.58; IQR 4.22) compared to Controls (median −0.40; IQR 2.16) (*p* = 0.001), whereas no statistically significant difference was found between MWA (median −0.26; IQR 2.00) and Controls (*p* = 0.307).

**Conclusion:** These findings point to neuroaxonal involvement rather than astroglial injury and suggest that axonal burden is mainly dominant in MWOA. Furthermore, this study highlights the need to search for neurobiological differences between migraine phenotypes, to enable diagnostic and therapeutic stratification.

**Disclosure:** H.T.: Received honoraria as a consultant/speaker and/or for events sponsored by Alexion, Bayer, Biogen, Bristol–Myers Squibb, Celgene, Diamed, Fresenius, Fujirebio, GlaxoSmithKline, Horizon, Janssen–Cilag, Merck, Novartis, Roche, Sanofi–Genzyme, Siemens, Teva and Viatris. No conflicts are relevant to the present study. F.B.: Nothing to disclose. P.K.: Nothing to disclose. Ö.S.: Nothing to disclose.

## Neuroimaging 2

## OPR‐148

### Functional classification of thalamic nuclei in myotrophic lateral sclerosis: Contribution of limbic and associative nuclei to behavioural symptoms and disease progression

#### A. Maranzano ^1^; G. Scacciatella^2^; A. Cocuzza^3^; V. Patisso^1^; S. Pierro^2^; M. passaretti^3^; S. Torre^1^; E. Aiello^1^; B. Poletti^1^; A. Doretti^1^; C. Morelli^1^; C. Cinnante^4^; J. Pereira^5^; V. Silani^6^; F. Verde^1^; N. Ticozzi^6^

##### ^*1*^ *Department of Neurology and Laboratory of Neuroscience, IRCCS Istituto Auxologico Italiano, Milan, Italy;* ^*2*^ *Neurology Residency Program, Università degli Studi di Milano, Milan, Italy;* ^*3*^ *IUSS Cognitive Neuroscience (ICON) Center, Institute for Advaced Studies, Pavia, Italy;* ^*4*^ *Department of Radiology and Diagnostic Imaging, IRCCS Istituto Auxologico Italiano, Milan, Italy;* ^*5*^ *Department of Clinical Neuroscience, Karolinska Institutet, Stockholm, Sweden;* ^*6*^ *Department of Pathophysiology and Transplantation Dino Ferrari Centre, Università degli Studi di Milano, Milan, Italy*

**Background and Aims:** Although amyotrophic lateral sclerosis (ALS) has traditionally been regarded as a disease primarily affecting cortical structures, growing evidence highlights the significant involvement of subcortical regions in the emergence and progression of clinical symptoms. In this study, we investigated whether thalamic subnuclei, grouped according to their functional specialization, contribute to the motor and neuropsychological phenotype of ALS patients.

**Methods:** We retrospectively analyzed 289 ALS patients who underwent 3‐Tesla MRI with a 3D T1‐weighted acquisition. Thalamic subnuclei volumes were estimated using FreeSurfer based on the probabilistic atlas of human thalamic nuclei (Iglesias et al., 2018) and subsequently grouped according to their functional classification into sensory, motor, limbic, associative, and diffuse projection nuclei.

**TABLE 1** Classification of thalamic nuclei according to their functional specialization

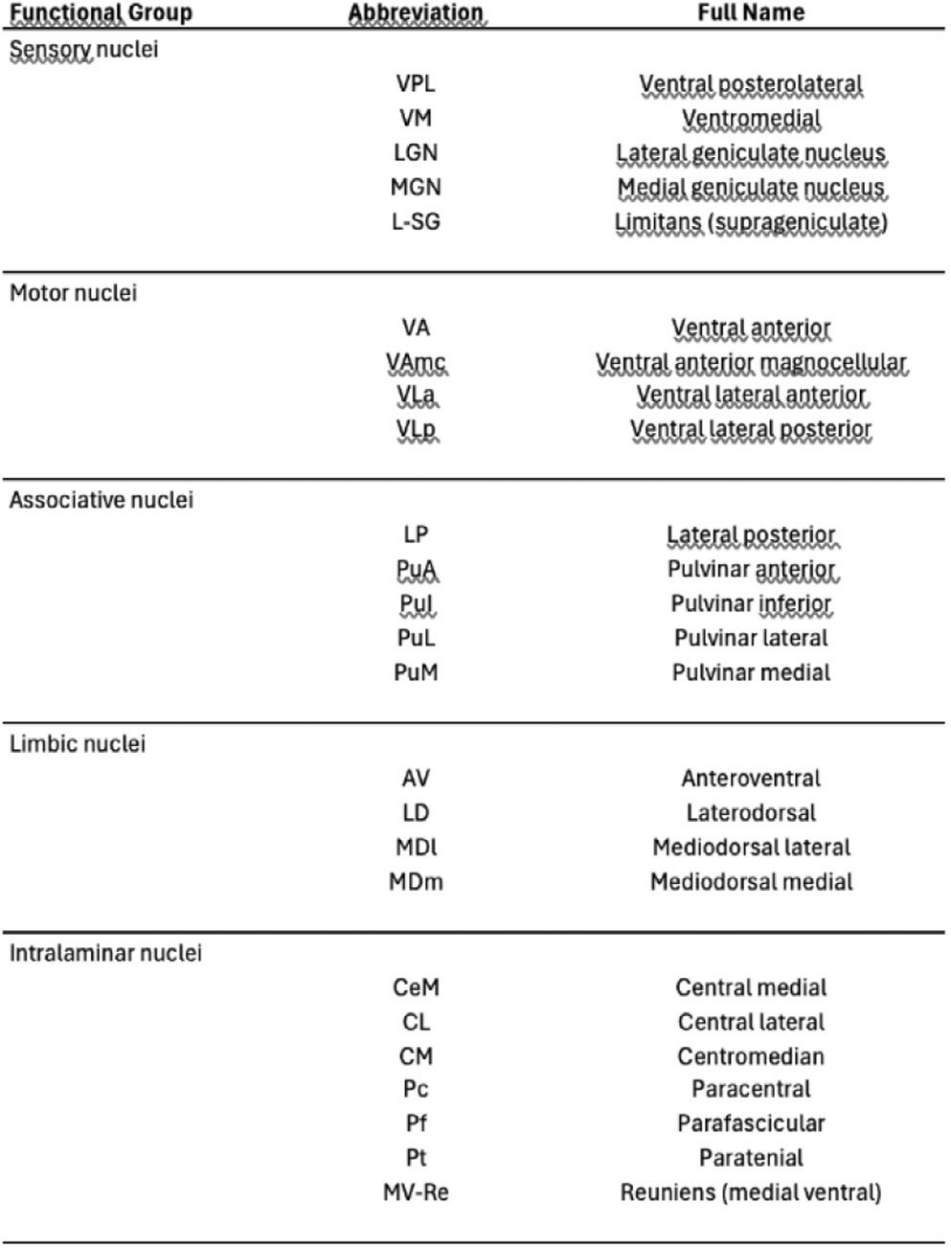

**Results:** Thalamic subnuclei volumes were not significantly associated with the motor or cognitive profiles of the cohort. In contrast, limbic nuclei volumes (specifically mediodorsal medial and lateral) were inversely associated with behavioral symptom burden (β = −0.28; *p* < 0.001) and plasma neurofilament light chain (NfL) levels (β = −0.26; *p* = 0.009). Notably, volumes of associative (specifically pulvinar and lateral posterior) thalamic nuclei were positively correlated with disease progression rate (β = 0.17; *p* = 0.001) and plasma NfL levels (β = 0.31; *p* < 0.001)

**Figure:**
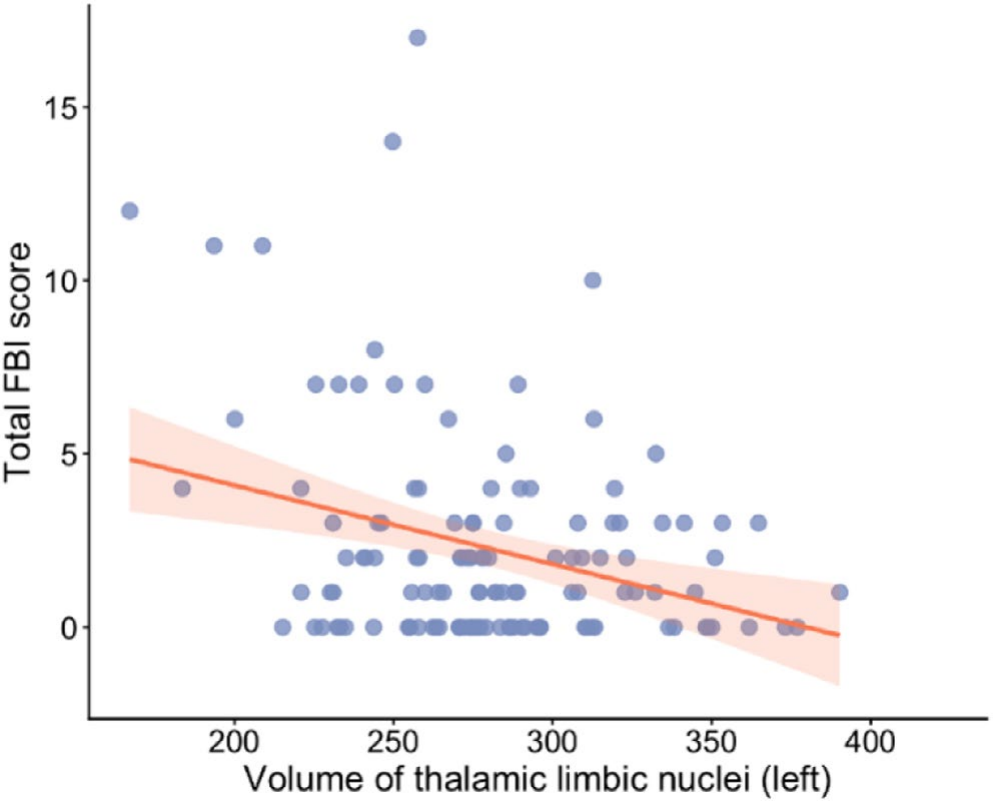
**FIGURE 1** Scatter plots with regression lines showing inverse association between FBI total score and volume of limbi thalamic nuclei. FBI, frontal behaviour inventory.

**Figure:**
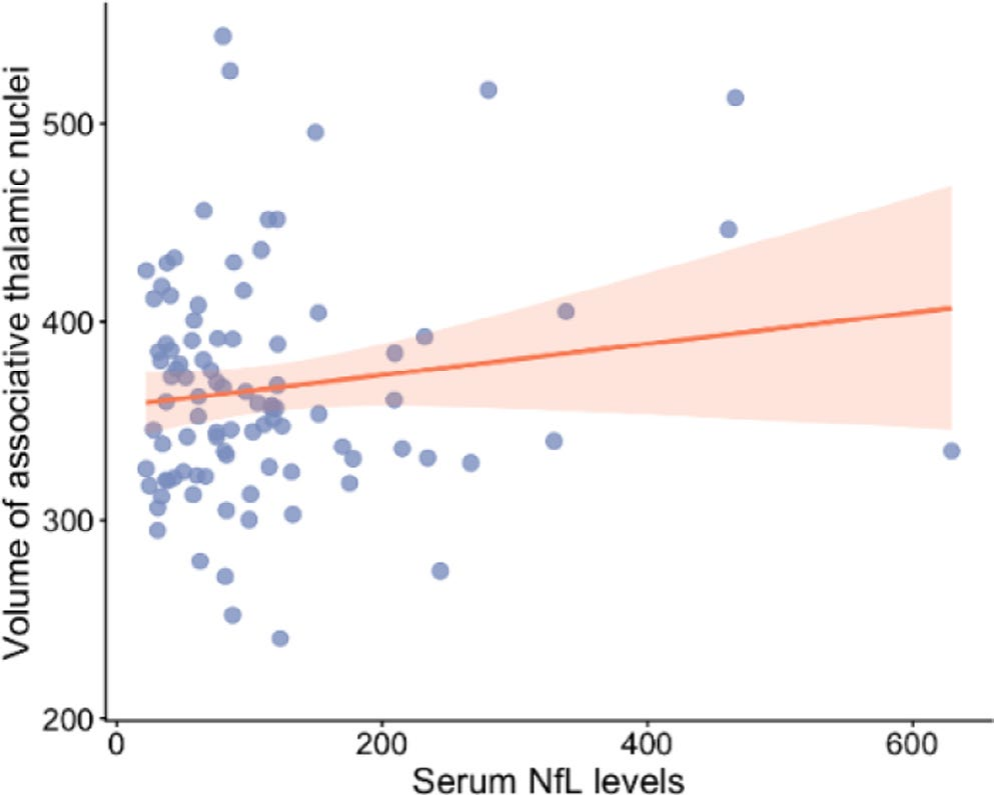
**FIGURE 2** Scatter plots with regression lines showing positive association between serum NFL levels and volume of associative thalamic nuclei. NFL, neurofilament light chain.

**Conclusion:** Findings from our work suggest that limbic thalamic nuclei may contribute to the emergence of behavioural symptoms and to the rate of neurodegeneration. In contrast, associative nuclei appeared to be relatively spared in patients with rapid disease progression, supporting a putative protective or compensatory role of the pulvinar–lateral posterior nuclei in the progression of ALS.

**Disclosure:** A. Maranzano, G. Scacciatella, A. Cocuzza, V. Patisso, S. Pierro, M. Passaretti, S. Torre, E.N. Aiello, A. Doretti, C. Morelli, C.M. Cinnante, J.B. Pereira, F. Verde report no disclosure. Barabara Poletti. received compensation for consulting services and/or speaking activities from Liquidweb S.r.l. and she is associated Editor for Frontiers in Neuroscience Vincenzo Silani received compensation for consulting services and/or speaking activities from AveXis, Cytokinetics, Italfarmaco, LiquidWeb, Srl and Novartis Pharma AG. He receives or he has received research support from the Italian Ministry of Health, AriSla, and E‐Rare Joint Translational Call. He is on. the Editorial Board of Amyotrophic Lateral Sclerosis and Frontotemproal Degeneration, European Neurology, American Journal of Neurodegenerative Disease and Frontiers in Neurology. Nicola Ticozzi received compensation for consulting services from Amylyx Pharmaceutical, Biogen, Italfarmaco and Zambon Biotech SA. He received research funding from the Italian Ministry of Health, the Italian Ministry of University and Research and AriSLA. He is associate editor of Frontiers in Aging Neuroscience.

## OPR‐149

### Structural brain changes in Alzheimer's disease: Relationship with cerebrospinal fluid pTAU181 and clinical implications

#### C. Manco ^1^; D. Van Rossem^2^; S. De Witte^2^; D. Righi^1^; F. Galasso^1^; B. Pucci^3^; I. Chiarotti^4^; A. Cerase^3^; N. De Stefano^1^; S. Engelborghs^2^; D. Plantone^1^

##### ^*1*^ *Department of Medicine, Surgery and Neuroscience, University of Siena, Siena, Italy;* ^*2*^ *NEUR Research Group, Center for Neurosciences (C4N), Vrije Universiteit Brussel (VUB) and Department of Neurology, UZ Brussel, Brussels, Belgium;* ^*3*^ *Azienda Ospedaliera Universitaria Senese, Siena, Italy;* ^*4*^ *Neuroradiologia, AOU Meyer, Florence, Italy*

**Background and Aims:** Alzheimer's disease (AD) is a neurodegenerative disorder characterized by cognitive decline and brain atrophy. Neuroimaging biomarkers reflecting underlying neuropathological processes are essential for diagnosis and prognosis. This study aims to investigate the relationship between cerebrospinal fluid (CSF) biomarkers, atrophy of relevant brain regions and cognitive decline in patients with AD.

**Methods:** Ninety‐four patients with mild cognitive impairment and mild dementia due to AD, diagnosed according to the 2024 NIA‐AA criteria, were enrolled [1]. Available data included CSF biomarkers, MRI‐derived brain volumes, demographic information at diagnosis, and Mini‐Mental State Examination (MMSE) scores at 1‐year follow‐up (56/94 patients). Patients were classified according to: pTAU181 levels above or below 78 pg/mL [2] (54/94 vs. 40/94); MMSE outcome at 1‐year (stable vs. worsened). Brain volumes were adjusted for age, sex, magnetic field strength, and total intracranial volume. Analyses of covariance and binomial regression models were applied.

**Results:** Forty‐six patients were recruited in Vrije Universiteit Brussels (mean age 72.17; 21 males/25 females; education 12.75) and forty‐eight in University of Siena (mean age 74.14; 25males/23females; education 9.34). Higher pTAU181 levels were associated with lower volumes of the hippocampal head (*p* = 0.005), body (*p* = 0.04), and tail (*p* = 0.03), the CA1 hippocampal subfield (*p* = 0.004), the medial‐mediodorsal thalamic nucleus (*p* = 0.02), total amygdala volume (*p* = 0.003), and basal (*p* < 0.001) and cortical (*p* = 0.02) amygdala nuclei. Only hippocampal head (*p* = 0.02) and CA1 volumes (*p* = 0.03) predicted cognitive performance at 1‐year follow‐up.

**Figure:**
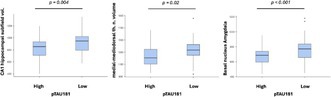
**FIGURE 1** Boxplots showing the comparison of regional brain volumes in subjects with high and low pTAU181 levels. Subjects with high pTAU181 levels exhibit significantly reduced volumes in all the brain regions shown.

**Conclusion:** Elevated CSF pTAU181 levels are associated with selective atrophy of hippocampal, thalamic, and amygdala regions. Hippocampal head and CA1 subfield volumes are linked to cognitive outcome.

**Disclosure:** Nothing to disclose.

## OPR‐150

### The developmental dynamics of cortical grey matter in children with self‐limiting epilepsy and centrotemporal spikes

#### C. Ciumas ^1^; K. Ostrowsky‐Coste^2^; E. Panagiotakaki^2^; P. Ryvlin^1^

##### ^*1*^ *Deparment of Clinical Neurosciences, CHUV, Lausanne, Switzerland;* ^*2*^ *ESEFNP Department – Clinical Epileptology, Sleep Disorders and Pediatric Functional Neurology, Femme Mère Enfant Hospital, Hospices Civils de Lyon (HCL), France*

**Background and Aims:** Self‐limited epilepsy with centrotemporal spikes (SeLECTS) has long being considered to be a benign condition that resolves without lasting consequences. However, evidence has emerged linking epileptiform activity in SeLECTS to impairments in language, attention, working memory and socio‐emotional processing, challenging this assumption. Recurrent centrotemporal spikes may disrupt critical phases of cortical maturation. In this context, the longitudinal impact of epileptiform activity on grey matter development in SeLECTS remains insufficiently explored.

**Methods:** We conducted a 5‐year longitudinal 1.5 T MRI study (Siemens) in children with SeLECTS and matched controls. 23 patients and 28 healthy participants (mean age: ~9–14 years) were included in the analyses. Cortical thickness trajectories were analysed using CAT12/SPM12, with vertex‐wise non‐parametric permutation testing of linear and quadratic effects adjusted for age, age^2^, and sex with family‐wise error correction, used for quantitation of thickness and thickness change.

**Results:** Vertex‐wise longitudinal analyses revealed the expected progressive cortical thinning in the control group across the frontal, cingulate, parietal, occipital and insular cortices. In the SeLECTS group cortical maturation followed distinct trajectories, with delayed linear thinning observed in the fronto‐central and paracentral regions alongside steeper thinning in the posterior associative cortices. Quadratic effects further distinguished between the two groups, indicating nonlinear deviations in cortical thickness trajectories over time, particularly within the medial frontal, cingulate and temporo‐parietal regions.

**Figure:**
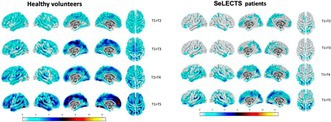
**FIGURE 1** Within group difference from T1 to T5.

**Figure:**
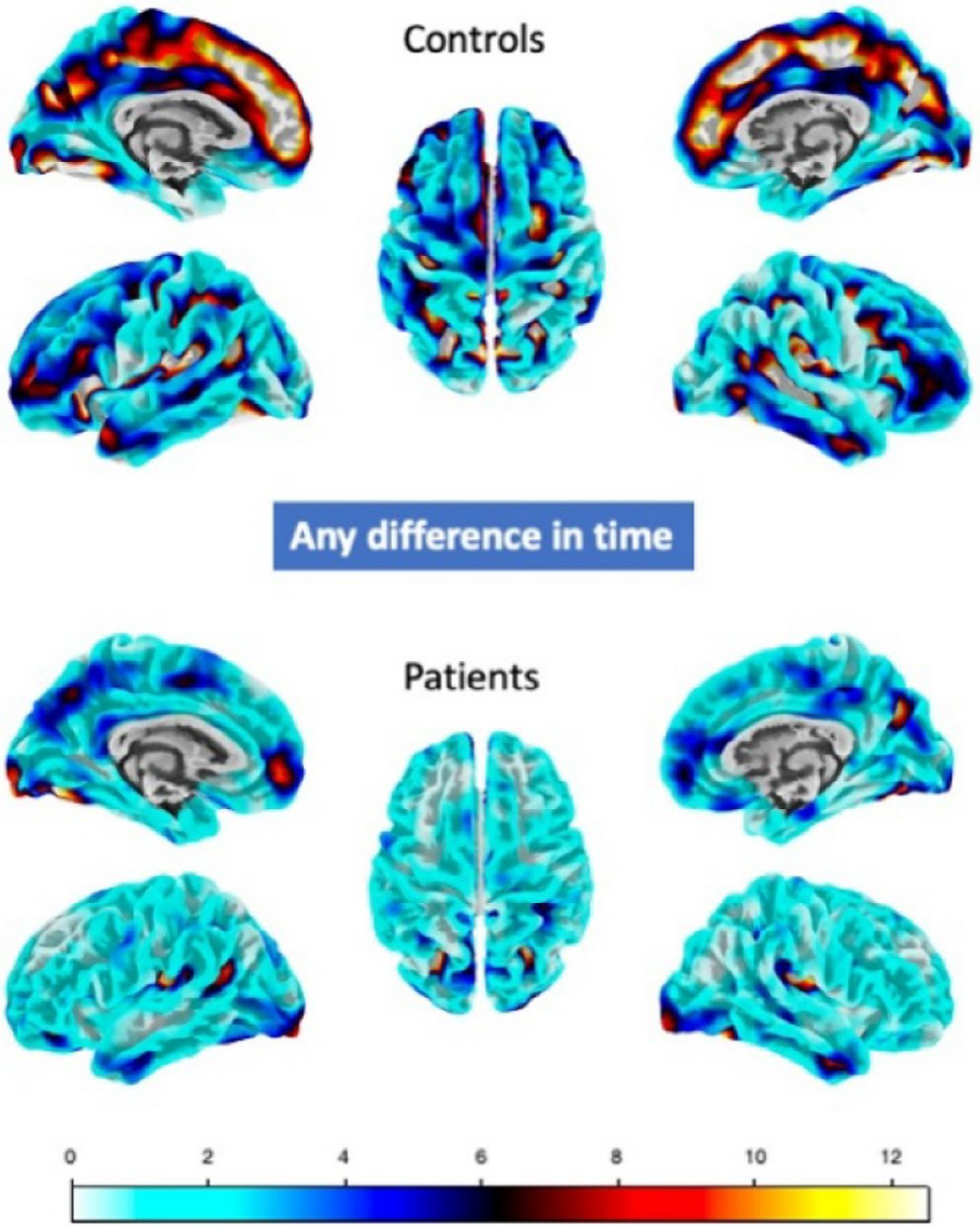
**FIGURE 2** Differences in each group over time.

**Figure:**
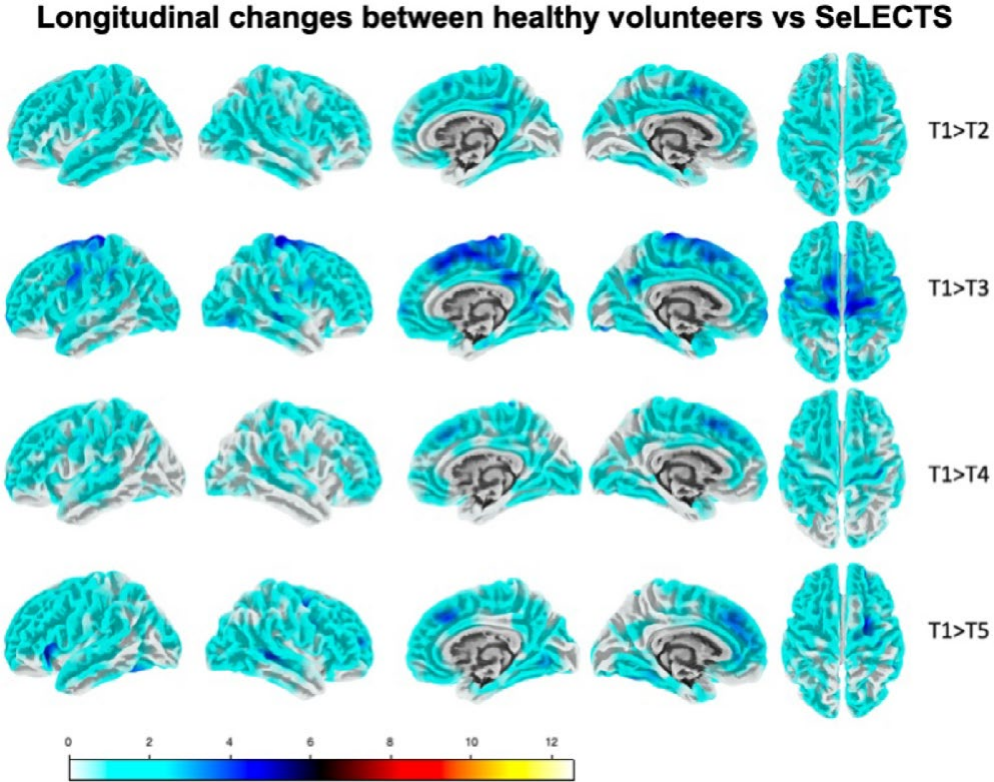
**FIGURE 3** Statistically significant differences between groups over 5 years period.

**Conclusion:** SeLECTS shows subtle, region‐specific alterations in cortical thinning trajectories, suggesting a neurodevelopmental impact that challenges its traditional characterization as a benign condition.

**Disclosure:** Nothing to disclose.

## OPR‐151

### MRI‐based brain aging models reveal specific atrophy patterns in multiple sclerosis according to age, sex, and age at onset

#### P. Preziosa ^1^; L. Storelli^2^; A. Meani^2^; A. Gallo^3^; A. d'Ambrosio^3^; P. Pantano^4^; C. Piervincenzi^5^; N. De Stefano^6^; R. Cortese^6^; M. Rocca^1^; M. Filippi^7^

##### ^*1*^ *Neuroimaging Research Unit, Division of Neuroscience, and Neurology Unit, IRCCS San Raffaele Scientific Institute, Milan, Italy; and Vita‐Salute San Raffaele University, Milan, Italy;* ^*2*^ *Neuroimaging Research Unit, Division of Neuroscience, IRCCS San Raffaele Scientific Institute, Milan, Italy;* ^*3*^ *Department of Advanced Medical and Surgical Sciences, and 3T MRI‐Center, University of Campania “Luigi Vanvitelli”, Naples, Italy; First Division of Neurology and Neurophysiopathology, AOU Luigi Vanvitelli, Naples, Italy;* ^*4*^ *Department of Human Neurosciences, Sapienza University of Rome, Rome, Italy; and IRCCS Neuromed, Pozzilli, Italy;* ^*5*^ *Department of Human Neurosciences, Sapienza University of Rome, Rome, Italy;* ^*6*^ *Department of Medicine, Surgery and Neuroscience, University of Siena, Siena, Italy;* ^*7*^ *Neuroimaging Research Unit, Division of Neuroscience, Neurology Unit, Neurorehabilitation Unit, and Neurophysiology Service, IRCCS San Raffaele Scientific Institute, Milan, Italy; and Vita‐Salute San Raffaele University, Milan, Italy*

**Background and Aims:** Physiological aging involves brain structural changes. Understanding these changes is essential to distinguish normal aging from disease‐related alterations in multiple sclerosis (MS). This study modeled normative age‐related trajectories of brain and grey matter (GM) volumes in healthy controls (HC) and applied them to a large multicenter MS cohort to identify disease‐specific atrophy patterns.

**Methods:** We analyzed 3D T1‐ and T2‐weighted MRI scans from 995 HC and 2016 MS patients from the Italian Neuroimaging Network Initiative (INNI). After image pre‐processing, normalized brain, cortical, and deep GM volumes were estimated using FSL‐SIENAx and FSL‐FIRST. Age‐related volumetric trajectories were modeled in HC using regression analyses accounting for linear and non‐linear age effects, sex, scanner, and interactions. These models were applied to MS patients to compute *z*‐scores reflecting disease‐related atrophy beyond normal aging, with analyses stratified by sex and age at disease onset.

**Results:** In HC, brain volume declined non‐linearly with age, while cortical and deep GM decreased linearly. In MS patients, *z*‐scores declined linearly with age (*p* < 0.01), indicating progressively greater MS‐related brain and GM atrophy. Cortical atrophy was more pronounced in men after age 40 (*p* = 0.03), while deep GM atrophy was greater in women after age 30 (*p* < 0.001). Pediatric‐onset MS showed faster brain and cortical atrophy than adult‐onset MS, whereas late‐onset MS exhibited greater brain and cortical atrophy than adult‐onset MS with aging.

**Conclusion:** In conclusion, these results highlight the need to account for both physiological aging and patient‐specific factors, such as sex and age at onset, when evaluating brain atrophy in individuals with MS.

**Disclosure:** Submitted on behalf of the INNI Network (Paola Valsasina, Nicolò Tedone, Stefania Sala, Manuela Altieri, Alvino Bisecco, Fabrizio Esposito, Alessandro De Rosa, Serena Ruggieri, Silvia Tommasin, Nikolaos Petsas, Costanza Gianni, Maria Laura Stromillo, Marco Battaglini). Supported by Fondazione Italiana Sclerosi Multipla (FISM2025/S/02). P. Preziosa received speaker honoraria from Roche, Biogen, Novartis, Merck, Bristol Myers Squibb, Genzyme, Horizon and Sanofi. L. Storelli has nothing to disclose. A. Meani has nothing to disclose. A. Gallo received speaker and consulting fees from Biogen, Genzyme, Merck Serono, Mylan, Novartis, Roche, and Teva, and receives research support from FISM. A. d'Ambrosio has nothing to disclose. P. Pantano has received funding for travel from Novartis, Genzyme, and Bracco and speaker honoraria from Biogen. She receives research support from the Italian Ministry of Health, the Italian Ministry of University and Research, and FISM. C. Piervincenzi has nothing to disclose. N. De Stefano has received honoraria from Schering, Biogen‐Idec, Teva, Novartis, Genzyme, and Merck Serono S.A. for consulting services, and speaking and travel support. He serves on advisory boards for Biogen‐Idec Merck Serono S.A. and Novartis. R. Cortese received speaker honoraria from Roche, Merck Serono and Sanofi and travel support for conferences by Novartis. M. Filippi is Editor‐in‐Chief of the Journal of Neurology, Associate Editor of Human Brain Mapping, Neurological Sciences, and Radiology; received compensation for consulting services from Almirall, Biogen, Bristol‐Myers Squibb, Eli Lilly, Merck, Novartis, Roche, Sanofi; speaking activities from Amgen, Bayer, Biogen, Bristol‐Myers Squibb, Celgene, Chiesi Italia SpA, Eisai, Eli Lilly, Fujirebio, Genzyme, Janssen, Merck, Neopharmed Gentili, Neuraxpharm, Novartis, Novo Nordisk, Roche, Sanofi, Takeda; participation in Advisory Boards for Alexion, Biogen, Bristol‐Myers Squibb, Eli Lilly, GE Healthcare Ltd, Merck, Neuraxpharm, Novartis, Roche, Sandoz, Sanofi, Takeda; scientific direction of educational events for Biogen, Merck, Roche, Celgene, Bristol‐Myers Squibb, Lilly, Novartis, Sanofi‐Genzyme; he receives research support from Biogen Idec, Merck‐Serono, Novartis, Roche, the Italian Ministry of Health, the Italian Ministry of University and Research, and FISM. M.A. Rocca received consulting fees from Biogen, Bristol Myers Squibb, Roche; and speaker honoraria from Alexion, Biogen, Bristol Myers Squibb, Celgene, Horizon Therapeutics Italy, Merck Serono SpA, Mitsubishi‐Tanabe Pharma, Neuraxpharm, Novartis, Roche, Sandoz, and Sanofi. She receives research support from the MS Society of Canada, the Italian Ministry of Health, the Italian Ministry of University and Research, and FISM. She is Associate Editor for Multiple Sclerosis and Related Disorders; and Associate Co‐Editor for Europe and Africa for Multiple Sclerosis Journal.

